# Programmable Functionalizations of Tetramethylpiperidine N‑Oxyl (TEMPO)–Acrolein Linchpins Derived from Alkoxyallenes

**DOI:** 10.1021/jacs.5c21371

**Published:** 2026-06-08

**Authors:** Ken S. Lee, Colin S. Crawford, George T. Cheng, Jensen J. Zerban, Emanuele Casali, Subir Panja, Daniel S. Rampon, Jennifer M. Schomaker

**Affiliations:** † Department of Chemistry, 5228 University of Wisconsin, 1101 University Avenue, Madison, Wisconsin 53706, United States; ‡ Department of Chemistry, University of Pavia, Viale Taramelli 12, 27100 Pavia, PV, Italy

## Abstract

Allenes are versatile precursors for constructing complex molecular frameworks, yet oxidative transformations to install C–C or C–heteroatom bonds across the C1–C3 framework rely on inconvenient reagents and yield overoxidized byproducts and unstable intermediates that limit downstream diversification. Herein, we report a general strategy to access multifunctional C­(sp^2^)-TEMPO-acrolein linchpins from simple alkoxyallenes. These TEMPO-acrolein intermediates enable orthogonal transformations of all three carbons of the initial allene framework into heterocyclic scaffolds that include pharmaceutically relevant quinoxalines, imidazoles, oxazoles, and thiazoles. Finally, the utility of this new strategic paradigm is demonstrated through a concise, metal-free synthesis of the natural product clathrodin.

## Graphical abstract

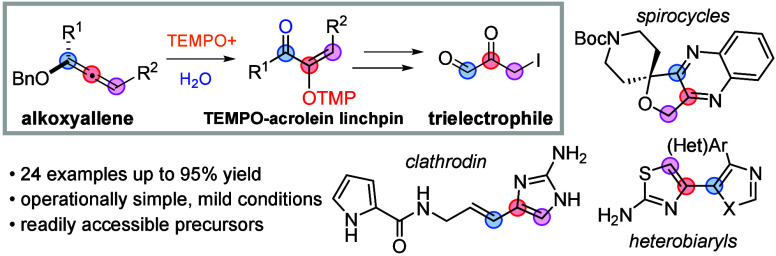

## Introduction

Allenes are the simplest cumulenes, comprised of three contiguous unsaturated carbon atoms in the form of two orthogonal alkenes.^1^ Their distinctive electronic and geometric features enable the installation of functionality across the entire C1–C3 framework of the allene, positioning these precursors as unique three-carbon synthons in synthesis.^2^ Allenes can be prepared from inexpensive, commercially available precursors and have emerged as valuable building blocks for complex molecules, as exemplified in the total syntheses of epoxomicin and rocaglamide (Scheme 1 **A**).^3^ Despite these advances, general strategies able to fully exploit the synthetic potential of allenes, particularly through programmed, multisite functionalizations, remain an ongoing challenge. In this context, oxidative allene trifunctionalizations that furnish chemically distinct sites of reactivity (Scheme 1 **B**) are attractive, but are often limited by harsh or inconvenient reagents, overoxidation, poor functional group tolerance, and the generation of unstable intermediates.^4,5^ Once the oxidation step is initiated, intermediates are not isolable, hampering the ability to program subsequent reactivity in a divergent and tunable manner.

**Scheme 1:**
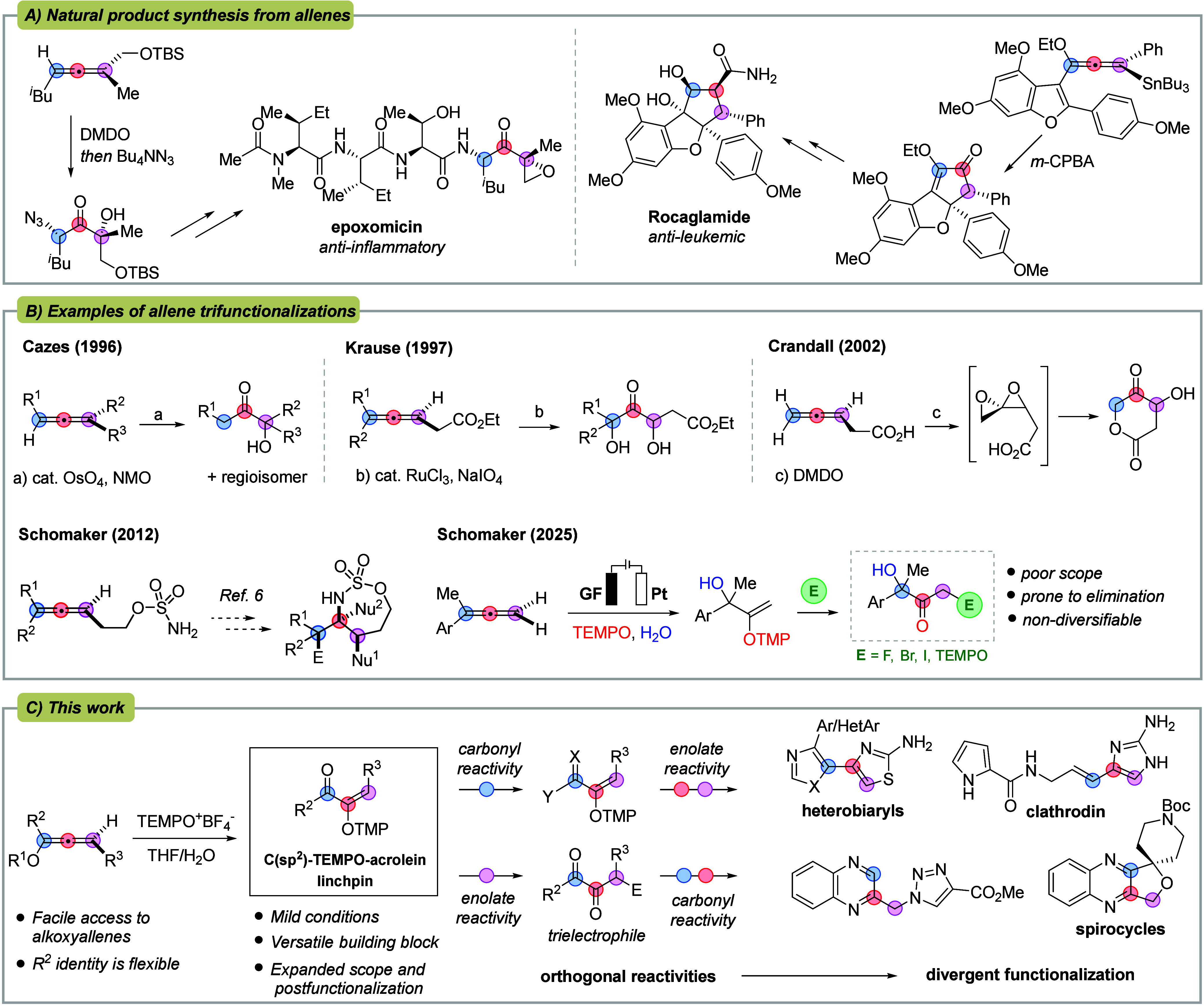
Oxidative Allene Functionalization

(A) Allenes as building blocks for natural product synthesis. (B) Previous examples of allene trifunctionalization via oxidative allene functionalization. (C) Oxidation of alkoxyallenes to form the TEMPO-acrolein linchpin and selected postfunctionalization examples.

Our group has a long-standing interest in methods for allene trifunctionalization (Scheme 1 **B**). Early work leveraged intramolecular nitrene transfer as a key step to ultimately form amine-containing stereotriads, but an intermolecular version of this chemistry was unsuccessful.^6,7^ More recently, we described an intermolecular electrooxidation of aryl-disubstituted allenes capable of functionalizing all three allene carbons.^8^ Unfortunately, the scope was restricted to 1,1′-Ar/Me substituted allenes, furnished poor yields and offered limited opportunities for diversification.

We envisioned leveraging electron-rich and readily synthesized alkoxyallenes^9^ as precursors to multifunctional, isolable intermediates that act as versatile platforms for downstream diversifications. We were especially interested in underexplored C­(sp^2^)-TEMPO adducts^10^ that combine the stability of a TEMPO-protected enol with the reactivity of a conjugated carbonyl system (Scheme 1 **C**). Herein, we report selective alkoxyallene oxidation to C­(sp^2^)-TEMPO-acroleins that function as versatile synthetic linchpins. In contrast to our previous work with aryl allenes, this chemistry gives rise to downstream orthogonal reactivity at the vinyl-TEMPO and carbonyl sites for elaboration into heterocycles and bioactive molecules. The mild reaction conditions, facile access to the alkoxyallene precursors, broad functional group compatibility, and versatility of this method provide a practical, programmable platform to transform alkoxyallenes into complex molecular architectures, as demonstrated by the synthesis of the pyrrole-imidazole alkaloid clathrodin.

## Results and Discussion

The TEMPO group is widely employed in organic synthesis, most commonly as a persistent radical trap in mechanistic studies.^11^ Beyond this role, well-established methods to install C­(sp^3^)-TEMPO groups, particularly those adjacent to carbonyl groups, are known. These include electrochemical and photoredox strategies, as well as enantioselective α-oxyamination reactions mediated by organocatalysis.^12^ Furthermore, alkene difunctionalizations, as exemplified by azidooxygenations reported from the Lin and Studer groups, provide access to C­(sp^3^)-TEMPO adducts that can be transformed into alcohols and ketones.^13,14^

In contrast to C­(sp^3^)-TEMPO compounds, C­(sp^2^)-TEMPO adducts are comparatively rare and have not been well-explored.^8,15^ We envisioned regioselective reaction of an alkoxyallene with TEMPO^+^, followed by hydrolysis (Scheme 1 **C**) would furnish a product with a unique combination of a stable enol ether and a conjugated carbonyl group. These C­(sp^2^)-TEMPO-acroleins have the potential to function as programmable linchpins, where the carbonyl and enol handles can be diversified in an orthogonal manner to deliver new heterocycles without the need for transition metal catalysis.

Allene **1** was selected as a nonvolatile model substrate; while other alkoxyallenes (e.g., methoxyallene) can be used, their low boiling points and relative instability made determining overall mass balance in our initial studies challenging. Optimized conditions combine **1** with TEMPO^+^ in 1:1 THF:H_2_O at 0 °C for 15 min (Figure 1A, for full screening results, see **Tables S2–S5** in the Supporting Information). Increasing the temperature to 25 °C or the time to 30 min resulted in decreased yields of **2**, suggesting it may be prone to decomposition (Figure 1A). In addition to benzyl alkoxyallenes, tetrahydropyran (THP)-protection was tolerated. Reaction in either acetonitrile or acetone gave lower yields, while a 10:1 THF:H_2_O solvent mixture did not substantially affect the outcome. A 1:10 THF:H_2_O mixture or H_2_O only gave low conversion of **1** to **2** due to poor substrate solubility.

**Figure 1:**
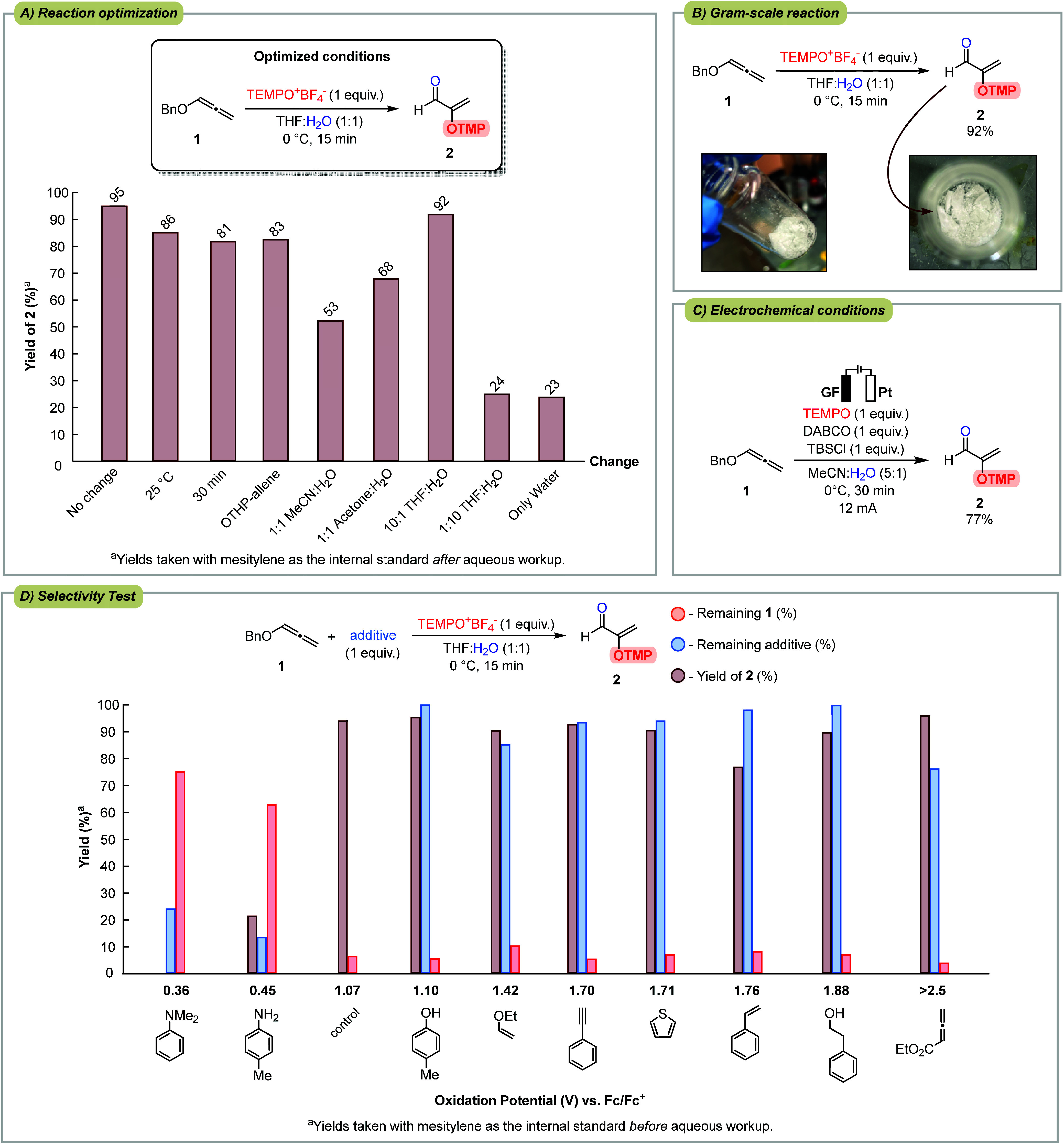
**Reaction optimization and selectivity**. (A) Impact of changes made to the optimized conditions on the yield of **2**. (B) Gram-scale reaction of **1** to **2** and visual representations of **2**. (C) Optimized electrochemical conditions. (D) Selectivity test with various additives of varying oxidation potentials and their impact on the formation of **2**.

The scalability of the reaction was demonstrated on a 1-gram scale of **1** (6.84 mmol), affording TEMPO-acrolein **2** in 92% isolated yield as a crystalline white solid (Figure 1B). Electrochemical oxidation could also be employed using TEMPO instead of TEMPO+, delivering **2** in a comparable 77% yield (Figure 1C, Supporting Information for details).

The reaction exhibits good chemoselectivity to form **2** even in the presence of other oxidizable functional groups. Additives, including alkenes, alkynes, alcohols, and heterocycles, gave good conversion of **1** to **2** (72–93% yields) and could be recovered in most cases (Figure 1D). As **1** has a lower oxidation potential than the alkene, alkyne, alcohol, and thiophene additives, it engages TEMPO^+^BF_4_^–^ in a productive manner,^16^ while the anilines oxidize in preference to **1**. Both radical and polar mechanisms were considered in a brief computational study (**Figures S6**, **S7** in the Supporting Information) and appear plausible. The alkoxyallene may directly attack TEMPO+ to form an oxocarbenium ion, followed by hydrolysis;^17^ alternatively, oxidation of the allene to a radical cation, followed by trapping with TEMPO, could form the TEMPO-acrolein adduct.

### Reaction Scope

With a reliable method in hand to access **2**, the generality of the transformation was examined. We prioritized substrates that introduce both structural complexity and useful handles for further elaboration to highlight the utility of C­(sp^2^)-TEMPO-acroleins as programmable synthetic linchpins. Three key features were of interest in the exploration of scope: (i) the ability of the method to tolerate complex and strained precursors; (ii) the potential for diastereoselective reactions to furnish stereochemically rich scaffolds, and (iii) versatility in the installation of functionality at C1 and C3 of the original allene.

C1-monosubstituted alkoxyallenes contain an acidic proton; treatment of **1** with *n*BuLi and various electrophiles furnishes disubstituted allenes of the general structure **3** (Figure 2).^18^ Ketones (**3a**–**n**), aldehydes (**3p**–**u**), and an alkyl halide (**3o**) readily furnished more elaborate alkoxyallenes, while a 1,3-disubstituted allene **7** was accessed via prefunctionalization at C3 of the alkyne precursor **6**, followed by base-mediated isomerization (Figure 2, bottom left).^19^

**Figure 2:**
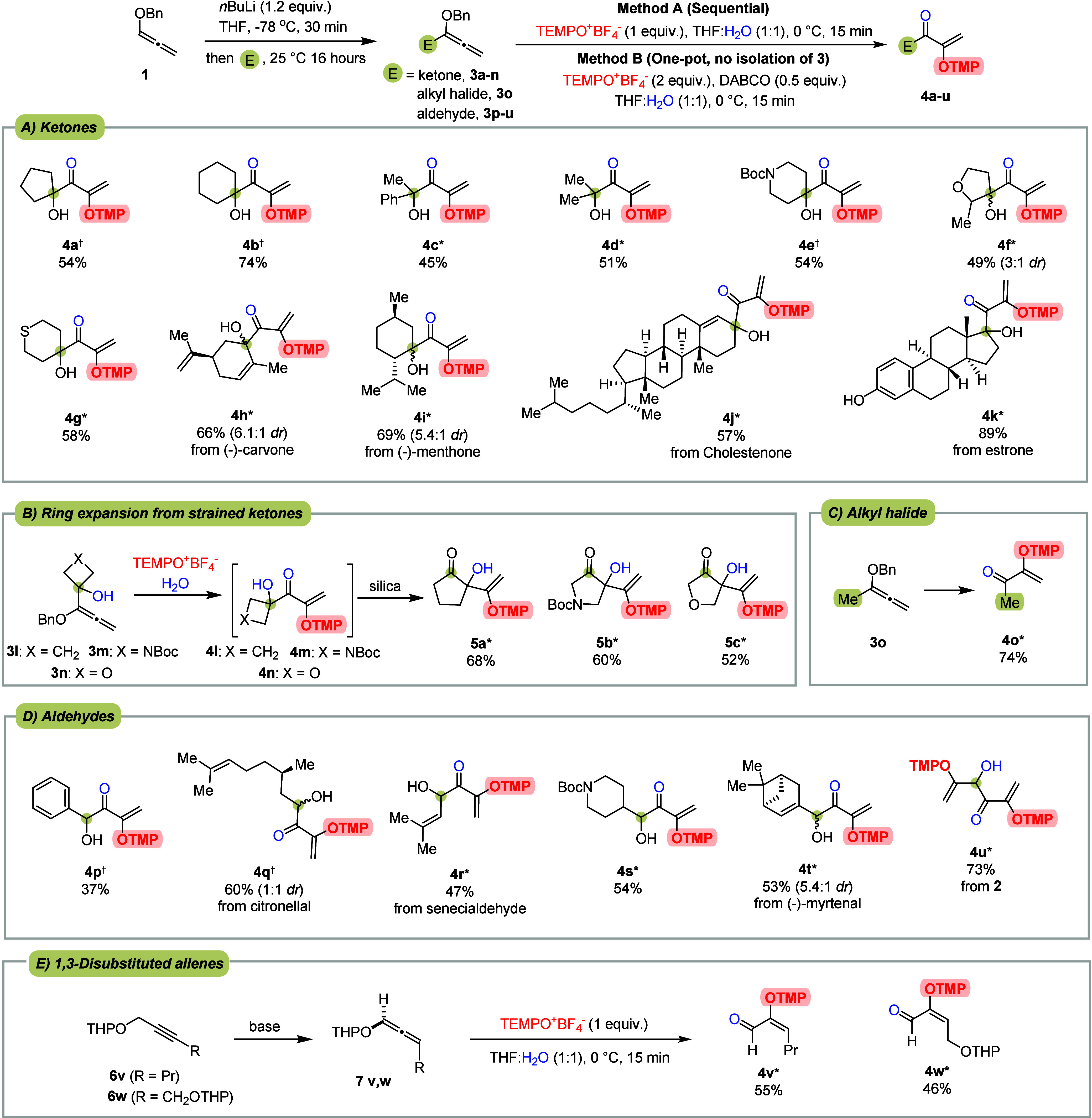
**Oxidation of alkoxyallene derivatives**. (A) Alkoxyallene derivatives from ketones. * Method A (Sequential) † Method B (One-pot). (B) Alkoxyallene derivatives from α-ketol rearrangements of strained ketones. (C) Alkoxyallene derivative from alkyl halide. (D) Alkoxyallene derivatives from aldehydes. (E) Products from reaction of 1,3-disubstituted allene derivatives.

Optimized conditions from Figure 1A were employed to evaluate the scope of the oxidation (Figure 2). While most alkoxyallenes could be prefunctionalized and isolated prior to oxidation, the instability of certain substrates necessitated a one-pot protocol, where crude intermediates were directly subjected to oxidation without isolation. In these cases, complete conversion of the allene required increased TEMPO^+^ loading to quench residual base from the preceding step. Under these conditions, **1** was converted to cyclic TEMPO-acroleins **4a** and **4b** in good yields.

Acyclic alkoxyallenes **3c**–**d** and heterocycle-derived ketones **3e–g** were transformed to the corresponding TEMPO-acroleins **4c**–**d** and **4e**–**g**, respectively, in moderate yields and modest *dr* for **4f**. Precursors derived from natural products bearing ketones, including (−)-carvone (**3h**), (−)-menthone (**3i**), cholestenone (**3j**), and estrone (**3k**), were competent substrates, affording **4h**–**k** in moderate yields (Figure 2A). Steric interactions between the TEMPO and the ketone framework are likely responsible for reduced yields, but the ability to incorporate both functional group and stereochemical complexity into the products underscores the versatility of this method. Finally, alkoxyallenes derived from strained cyclic ketones, such as cyclobutanone (**3l**), *N*-Boc azetidinone (**3m**), and oxetanone (**3n**), underwent oxidation to **4l–n**, followed by a Wagner-Meerwein shift to deliver ring-expanded products **5a**–**c**. Note these rearranged products reverse the position of the carbonyl and alcohol groups as compared to **4a**–**k** (Figure 2B).^20^

Treatment of the anion of **1** with MeI gave the 1,1-disubstituted allene **3o**, which was converted to the corresponding TEMPO-acrolein **4o** in good yield (Figure 2C). A series of alkoxyallenes derived from aldehydes **3p**–**u**, including citronellal, senescialdehyde and (−)-myrtenal, were smoothly converted to the TEMPO-acroleins **4p**–**u** (Figure 2D). Notably, TEMPO-acrolein **2** itself could be employed as an electrophile to furnish **4u**, introducing additional sites for diversification. Finally, 1,3-disubstituted alkoxyallenes **7a** and **7b**, derived from alkynes **6v** and **6w**, successfully underwent oxidation to yield the TEMPO-acroleins **4v** and **4w** respectively (Figure 2E). Collectively, these results demonstrate that a broad array of alkoxyallenes can be converted into structurally diverse TEMPO-acroleins under mild conditions, enabling convergent access to a unified class of multifunctional TEMPO-acroleins as useful entry points for downstream synthetic elaborations.

### Synthetic Applications

With mild conditions in hand to generate diverse TEMPO-acroleins, we next evaluated the potential of **2** as a diversifiable building block for the synthesis of pharmaceutically relevant scaffolds. A defining feature of these intermediates is the presence of two orthogonal reactive sites: a conjugated aldehyde and a vinyl-TEMPO moiety. The vinyl-TEMPO moiety acts as a protected enolate that may react with various electrophiles (**E**) to reveal an α-functionalized ketone (Scheme 2 **A**, right). Meanwhile, the aldehyde group in **2** offers a versatile entry point to classical aldehyde transformations to access a wide range of other useful functional groups and heterocycles (Scheme 2 **A**, left).^21^

**Scheme 2:**
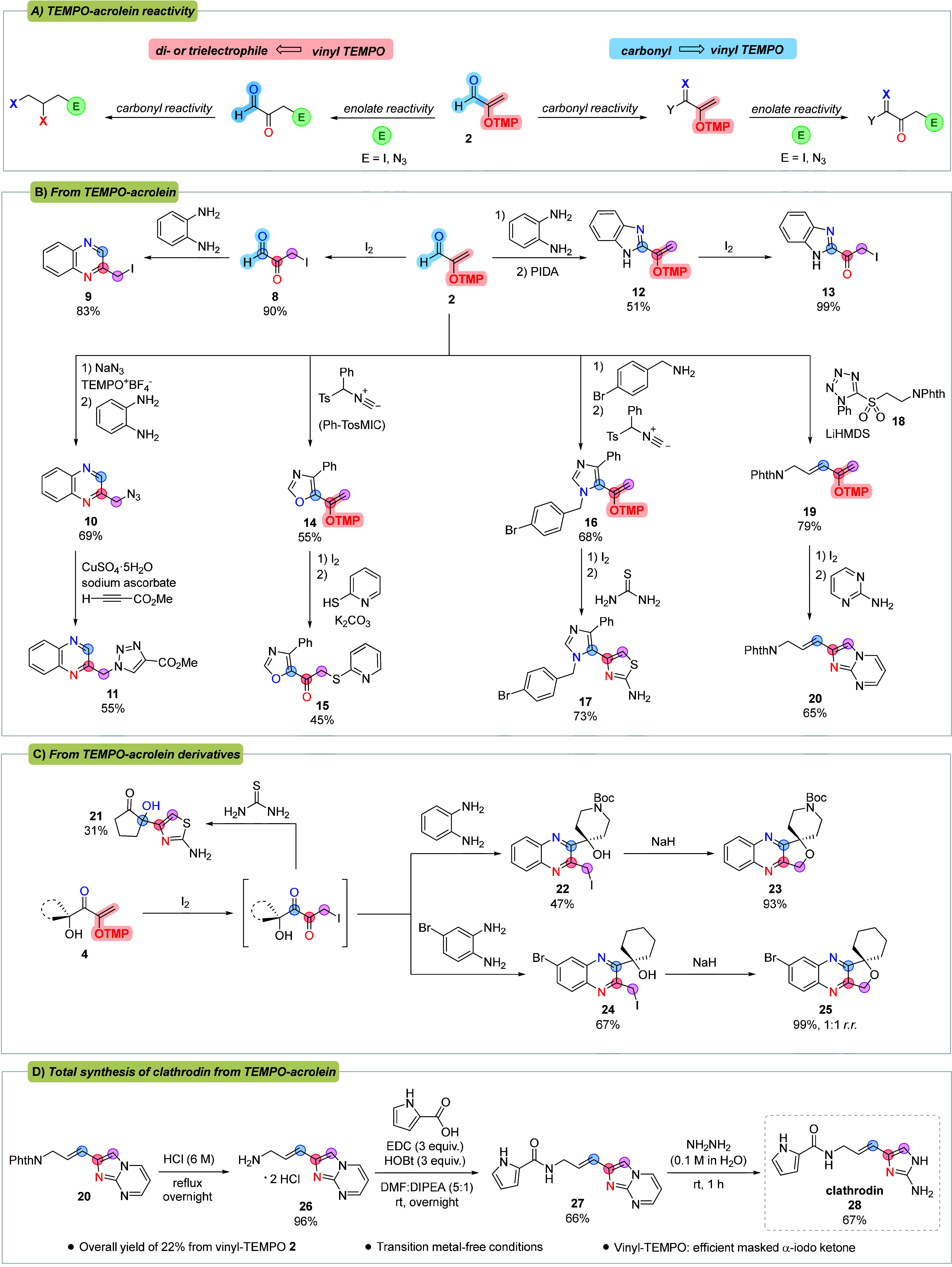
Postfunctionalization of TEMPO-Acrolein and Its Derivatives^a^

a (A) Potential transformations of the vinyl-TEMPO and aldehyde moieties on TEMPO-acrolein. (B) Postfunctionalization of TEMPO-acrolein. (C) Postfunctionalization of selected TEMPO-acrolein derivatives. (D) Total synthesis of clathrodin from TEMPO-acrolein.

Representative examples that showcase the synthetic utility of **2** are highlighted in Schemes 2 **B-D**. Iodination of **2** furnished trielectrophile **8** in 90% yield (Scheme 2 **B**), which underwent a one-pot condensation with 1,2-phenyldiamine to deliver the quinoxaline **9** in good yield.^22^ Quinoxaline derivatives are of particular interest in medicinal chemistry due to their broad range of physiochemical and biological properties; the modularity of **2** provides a straightforward entry into this valuable scaffold.^23^

In a similar fashion, azidation of **2**, followed by condensation with 1,2-phenyldiamine, afforded the azido-quinoxaline **10** in 69% yield. Subsequent Cu-catalyzed 1,3-dipolar cycloaddition delivered triazole **9** in 55% yield.^14,24^ These complementary pathways illustrate the potential for either divergent polar (iodination) or radical (azidation) reactivities of the TEMPO-acrolein motif.^13,17^

Engaging the carbonyl reactivity of **2** (Scheme 2 **B**) via condensation with 1,2-phenyldiamine, followed by oxidation, delivered the benzimidazole **12**, which was converted to α-iodo ketone **13** in quantitative yield.^25^ Isocyanate condensation with phenyl toluenesulfonylmethyl isocyanide (Ph-TosMIC) and **2** afforded oxazole **14** in moderate yield, which underwent iodination and S_N_2 displacement with 2-mercaptopyridine to afford imidazole **15**.^26^ A similar isocyanate condensation was conducted on a precondensed variant of **2** with 4-bromobenzylamine to afford imidazole **16**; sequential treatment with I_2_ and thiourea afforded the heterobisaryl **17**.^27^ Oxazoles, imidazoles, and thiazoles are naturally occurring secondary metabolites derived from amino acids and show significant bioactivities.^28^

Julia-Kocienski olefination of **2** with tetrazole **18** and lithium bis­(trimethylsilyl)­amide (LiHMDS) gave **19**; iodination and condensation with 2-aminopyrimidine gave imidazo­[1,2-α]­pyrimidine **20**, a key precursor to pyrrole-imidazole alkaloid (PIA) natural products.^29^ Finally, TEMPO-acrolein **4** proved amenable to an iodination–condensation sequence to access more elaborate heterocycles (Scheme 2 **C**). For example, iodination of the α-ketol rearrangement product **5a**, followed by condensation with thiourea, yielded thiazole **21**. Similarly, ketone adducts **4e** and **4b** were transformed into quinoxalines **22** and **24** via iodination and condensation with 1,2-phenylenediamine and 4-bromo-1,2-diaminobenzene, respectively. Subsequent NaH-mediated cyclization furnished heterospirocycles **23** and **25**, providing rapid entry to structurally complex scaffolds in only three steps from benzoxyallene **1** and avoiding the use of precious metal catalysis. Collectively, these transformations demonstrate that TEMPO-acroleins function as three-carbon synthons with programmable, site-selective reactivity at each position.

To further highlight the potential of **2** as a useful building block, it was applied to the total synthesis of clathrodin (Scheme 2 **D**).^30^ Clathrodin is a pyrrole-imidazole alkaloid isolated from the Caribbean sponge *Agelas clathrodes* and is structurally related to the brominated congeners oroidin and hymenidin. The compound exhibits diverse biological activities that include antimicrobial, antihistaminic, and cytotoxic properties.^31^ We envisioned a route in which the imidazole fragment would arise from an imidazo­[1,2-α]­pyrimidine, itself accessible from condensation of an α-halo ketones.^29^ Leveraging the vinyl-TEMPO motif as a protected α-iodo ketone, the remaining fragment was installed through Julia-Kocienski olefination of **2**, followed by iodination and condensation to yield **20** (Scheme 2B, right). From the imidazo­[1,2-α]­pyrimidine **20**, acid-mediated phthalimide deprotection afforded amine **26** in 96% yield. Subsequent amide coupling with the pyrrole fragment gave **27**, and the last hydrazine-mediated deprotection delivered clathrodin **28** in five steps from TEMPO-acrolein **2** with an overall yield of 22% with no precious metal catalysis.

## Conclusion

In summary, we have developed a new strategy for versatile and programmable allene reactivity through the use of C­(sp^2^)-TEMPO-acroleins as a unifying intermediate. This mild and operationally simple strategy converts readily accessible alkoxyallenes into stable, isolable C­(sp^2^)-TEMPO-acroleins as multifunctional intermediates. The unique combination of a conjugated carbonyl moiety and a vinyl-TEMPO motif enables orthogonal and sequential reactivity, supporting both polar and radical transformations. This dual reactivity allows modular access to structurally diverse heterocycles, including quinoxalines, imidazoles, oxazoles, and thiazoles. Finally, the power of this multifunctional platform is illustrated by a concise synthesis of clathrodin.

## Supplementary Material



## Notes

Characterization data, optimization tables, additional substrates/catalysts, and details of computational methods are included in Supporting Information. This material includes experimental details, analytical data, ^1^H, ^13^C, ^19^F NMR spectra and computational data (PDF)
