# Supplementary material for: Programmable Functionalizations of Tetramethylpiperidine N‑Oxyl (TEMPO)–Acrolein Linchpins Derived from Alkoxyallenes
Source: J Am Chem Soc. 2026 Jun 8;148(23):23568–76. doi: 10.1021/jacs.5c21371 (PMC13281524; doi:10.1021/jacs.5c21371)

Programmable Functionalizations of Tetramethylpiperidine N-Oxyl (TEMPO)-Acrolein Linchpins Derived from Alkoxyallenes

Ken S. Lee[1]†, Colin S. Crawford[1]†, George T. Cheng[1], Jensen J. Zerban[1], Emanuele Casali[2], Subir Panja[1], Daniel S. Rampon[1], Jennifer M. Schomaker[1]*

[1]Department of Chemistry, University of Wisconsin-Madison, Wisconsin 53706, United States

[2]Department of Chemistry, Viale Taramelli 12, University of Pavia, 27100 Pavia, PV, Italy

*Corresponding author. Email: schomakerj@chem.wisc.edu

Supporting Information

Table of contents

I. General Information.. S2
II. Reaction Optimization and Additional Experiments.. S3
III. Starting Material Synthesis.. S9
IV. Allene Dioxygenation Reactions.. S15
V. Cyclic Voltammograms.. S25
VI. Proposed Mechanism.. S26
VII. Postfunctionalization Reactions.. S26
VIII. Computational details and Cartesian coordinates.. S34
IX. References.. S62
X. NMR Spectra.. S65

I. General Information.

Batch dioxygenations were performed in 2- and 4-dram vials as well as round-bottom flasks, while electrochemical diooxygenations were performed in Schlenk tubes unless otherwise noted. Unless otherwise specified, reagents were used as obtained from Sigma-Aldrich, Oakwood Products, Alfa Aesar, Combi-Blocks, Acros Organics, or Chem-Impex and used directly without further purification. The 2,2,6,6-Tetramethyl-1-oxo-piperidinium tetrafluoroborate (TEMPO+) was synthesized according to the literature.[1] 1-Benzyloxyallene or allene **1** was synthesized according to the literature.[2] Platinum wire (diameter: 0.254 mm × length: 14 mm, 99.99+%, obtained from Strem) and graphite felt (length: 20 mm × width: 10 mm × height: 5 mm, Fuel Cell Earth) were connected using a house-made stainless steel electrode holder. Electrolysis was conducted using a house-made power supply in constant current mode. For detailed information, see the discussion below. Cyclic voltammetry studies were performed using a WaveNow^{xv} Potentiostat. Acetonitrile and tetrahydrofuran were dispensed from an Inert PureSolv PS-MD-5 solvent purification system. Analytical thin layer chromatography (TLC) was performed utilizing pre-coated silica gel 60 F254 plates containing a fluorescent indicator. Hexanes/ethyl acetate (EtOAc) was used as the mobile phase for column chromatography. All flash column chromatography was typically performed using a gradient method, beginning with 100% of the less polar eluent and gradually increasing the polarity with the other solvent. For reactions yielding products without a UV signature, phosphomolybdic acid (PMA) was employed to visualize the reaction progress. In all cases where TEMPO+ is mentioned, the TEMPO+ BF_4^- salt is used. ^{1}H NMR and ^{13}C NMR spectra were obtained using Bruker Avance III 500, Bruker Avance III 400, and Bruker Avance Neo 500 spectrometers. For ^{1}H NMR, chemical shifts are reported relative to residual protiated solvent peaks (d 7.26 and 1.94 ppm for $CDCl_3$ and CD_3CN, respectively). ^{13}C NMR spectra were measured at 125 MHz on the same instruments noted above for recording ^{1}H NMR spectra. Chemical shifts were reported according to the ^{13}C solvent peaks (d 77.1 ppm for $CDCl_3$). Accurate mass measurements were acquired at the University of Wisconsin, Madison using a Thermo Q Exactive™ Plus (electrospray ionization or atmospheric solids analysis probe (ASAP-MS) methods). The NMR facilities are funded by the NSF (CHE-1048642 and CHE-2017891). The purchase of the Thermo Q Exactive™ Plus in 2015 was funded by NIH Award 1S10 OD020022-1 to the Department of Chemistry.

II. Reaction Optimization and Additional Experiments.

Table S1. Formation of **2** initial screen.

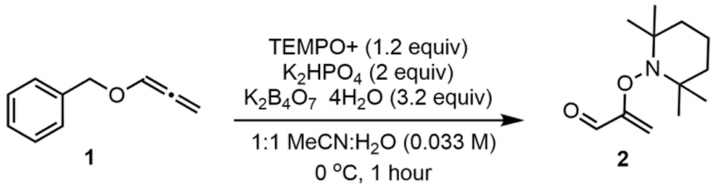

Entry	*Change*	**2** (%)[a]
1	No Change	48
2	2 equiv $K_2B_4O_7$	43
3	1 equiv $K_2B_4O_7$	53
**4**	**0 equiv $K_2B_4O_7$**	**64**
5	3 equiv K_2HPO_4	9
**6**	**1 equiv of K_2HPO_4**	**47**
7	2 equiv of TEMPO+	19
8	3 equiv of TEMPO+	13
9	2:1 Solvent Ratio	35
10	10:1 Solvent ratio	50
**11**	**0.05 M**	**49**
12	0.1 M	37

[a]Yields determined by 1H NMR using mesitylene as the internal standard

Table S2. Formation of **2** base screen.

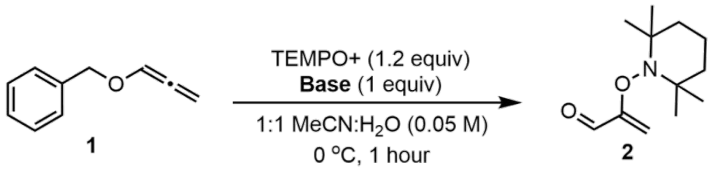

Entry	*Base*	**2** (%)[a]
1	K_2HPO_4	64
2	2,6-lutidine	63
3	pyridine	58
4	2,4,6-collidine	44
5	NMI	45
6	NEt_3	0
7	DBU	0
8	Imidazole	30
9	KH_2PO_4	21
10	K_3PO_4	58
11	$CaHPO_4$	59
12	$KHSO_4$	0
13	KOtBu	41
**14**	**DABCO**	**73**
15	Cs_2CO_3	24
16	DIPEA	0

[a]Yields determined by 1H NMR using mesitylene as the internal standard

Table S3. Formation of **2** solvent screen.

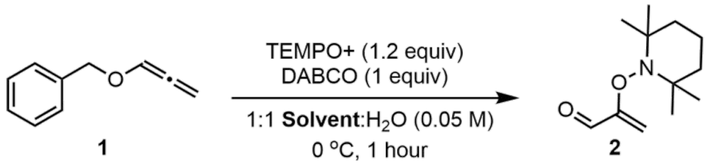

Entry	*Solvent*	**2** (%)[a]
1	HFIP	40
2	TFE	2
3	Acetone	62
4	DCM	29
**5**	**THF**	**76**
6	Hexanes	8
7	DME	51
8	MTBE	28
9	THP	28
10	2-MeTHF	36
11	CPME	19

[a]Yields determined by 1H NMR using mesitylene as the internal standard

Table S4. Formation of **2** initial control experiments.

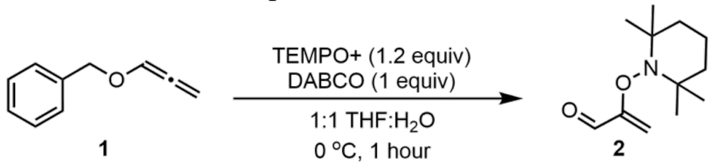

Entry	*Change*	**2** (%)[a]
1	No DABCO	77
2	No DABCO, 1.0 equiv TEMPO+	79

[a]Yields determined by 1H NMR using mesitylene as the internal standard

Table S5. Formation of **2** over time screen.

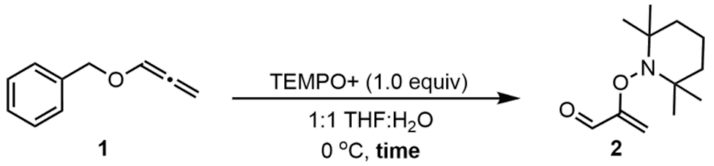

Entry	*Change*	**2** (%)[a]
1	60 min.	79
2	45 min.	78
3	30 min.	81
**4**	**15 min.**	**95**

[a]Yields determined by 1H NMR using mesitylene as the internal standard

Table S6. Electrochemical formation of **2 s**olvent screen.

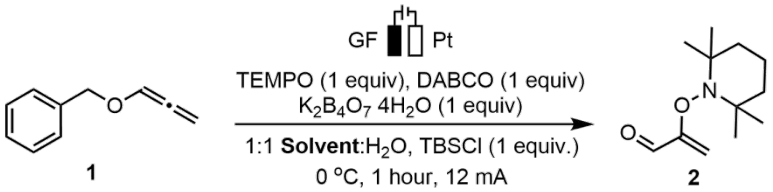

Entry	*Solvent*	**2** (%)[a]
1	DCE	0
2	PhMe	0
3	DMF	8
**4**	**MeCN**	**52**
5	1,2 dimethoxy ethane	0
6	DMA	13
7	PhCl	0
8	PhCF3	0
9	Acetone	0
10	DMSO	1
11	1,1,2,2-tetrachloro ethane	3
12	1,1,1-trichloro ethane	0
13	1,3-dibromo propane	2
14	1,1- dibromo methane	0
15	TFA	1
16	NMP	36

[a]Yields determined by 1H NMR using mesitylene as the internal standard

Table S7. Electrochemical formation of **2** time screen.

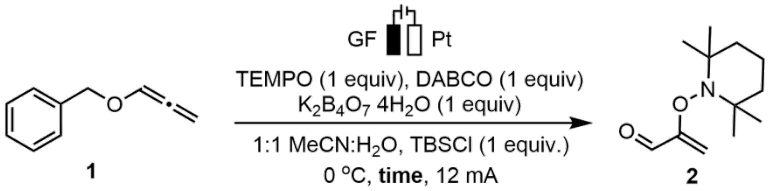

Entry	*Time (min.)*	**2** (%)[a]
1	10	18
2	20	17
**3**	**30**	**57**
4	40	52
5	50	13
6	60	52
7	75	24
8	90	40
9	120	49
10	180	55
11	240	25
12	300	24

[a]Yields determined by 1H NMR using mesitylene as the internal standard

Table S8. Electrochemical formation of **2** current screen.

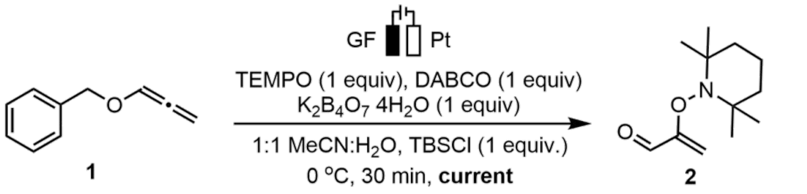

Entry	*Current (mA)*	**2** (%)[a]
1	1	4
2	2	5
3	3	5
4	4	6
5	5	18
6	6	6
7	7	19
8	8	1
9	9	18
10	10	20
11	11	12
12	**12**	**57**
**13**	13	16
14	14	24
15	15	30
16	16	15
17	17	25
18	18	13
19	19	24
20	20	20

[a]Yields determined by 1H NMR using mesitylene as the internal standard

Table S9. Electrochemical formation of **2** TEMPO equivalents screen.

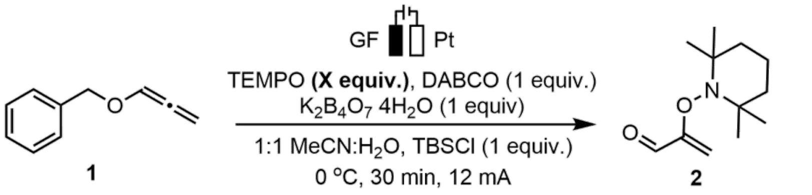

Entry	*TEMPO Equivalents*	**2** (%)[a]
**1**	**1.0**	**57**
2	1.2	48
3	1.5	21
4	1.7	28
5	2.0	27

[a]Yields determined by 1H NMR using mesitylene as the internal standard

Table S10. Electrochemical formation of **2** DABCO equivalents screen.

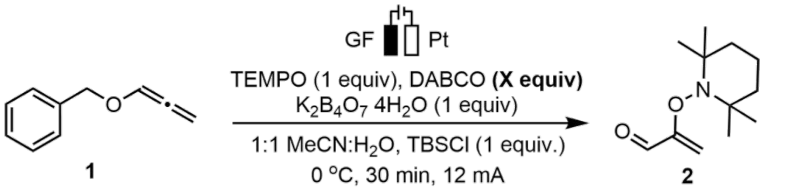

Entry	*DABCO Equivalents*	**2** (%)[a]
1	0	35
2	0.5	26
**3**	**1.0**	**57**
4	1.5	42
5	2.0	31
6	2.5	32
7	3.0	11
8	3.5	24
9	4.0	25

[a]Yields determined by 1H NMR using mesitylene as the internal standard

Table S11. Electrochemical formation of **2** electrolyte equivalents screen.

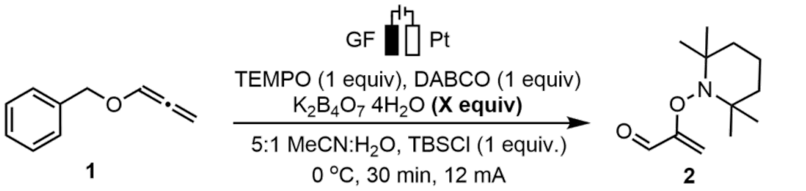

Entry	*Electrolyte Equivalents*	**2** (%)[a]
**1**	**0**	**70**
2	0.5	20
3	1.0	57
4	1.5	32
5	2.0	33
6	2.5	24
7	3.0	24
8	3.5	16
9	4.0	10

[a]Yields determined by 1H NMR using mesitylene as the internal standard

Table S12. Electrochemical formation of **2** solvent equivalents screen.

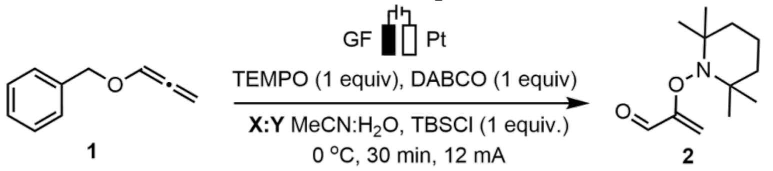

Entry	*Solvent Ratio*	**2** (%)[a]
1	100:0	22
2	0:100	7
3	1:1	57
4	2:1	34
5	1:2	23
**6**	**5:1**	**77**
7	1:5	8
8	9:1	66
9	19:1	39
10	29:1	28

[a]Yields determined by 1H NMR using mesitylene as the internal standard

Table S13. Reproducibility test.

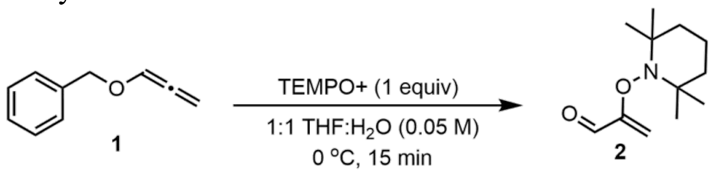

Entry	**2** (%)[a]
1	95
2	93
3	93

[a]Yields determined by 1H NMR using mesitylene as the internal standard

III. Starting Material Synthesis.

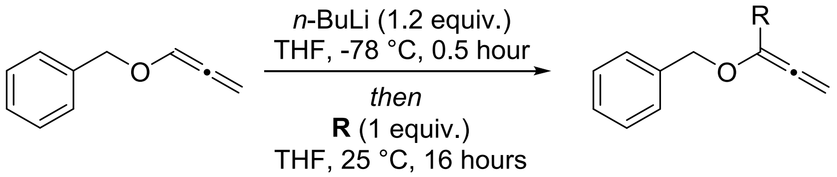

General Procedure for the Synthesis of 1,1-disubstituted allenes

A flame-dried round-bottom flask under nitrogen was charged with allene **1** (0.40 mmol) in anhydrous THF (0.1 M). The solution was cooled to –78 °C, and n-BuLi (1.2 equiv) was added dropwise. The mixture was stirred at –78 °C for 30 min, then treated with the electrophile (1.0 equiv). The reaction was allowed to warm to room temperature and stirred for 16 hours. The mixture was quenched with water and extracted with DCM (3×). The combined organic layers were dried over $MgSO_4$, filtered, and concentrated under reduced pressure. The crude residue was purified by column chromatography (Hex/EtOAc), using silica neutralized with 2% NEt_3 in hexanes, to afford allene **3**.

3-(benzyloxy)-2-phenylpenta-3,4-dien-2-ol (3c).

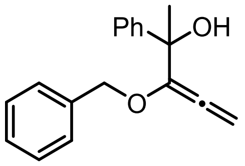

Prepared from the above procedure from benzoxyallene in 78% yield.
^{1}H NMR (500 MHz, CDCl$_3$) δ 7.55 – 7.49 (m, 2H), 7.39 – 7.32 (m, 2H), 7.31 – 7.26 (m, 5H), 7.20 (dd, *J* = 7.6, 2.1 Hz, 2H), 5.65 (d, *J* = 1.6 Hz, 2H), 4.73 – 4.63 (m, 2H), 2.95 (s, 1H), 1.61 (s, 3H).
^{13}C NMR (126 MHz, CDCl$_3$) δ 197.2, 148.1, 146.3, 137.2, 136.8, 128.3, 128.1, 128.0, 127.8, 127.6, 127.0, 126.5, 125.3, 124.8, 93.1, 77.3, 77.0, 76.8, 74.9, 74.7, 70.9, 44.0, 30.2, 28.5, 26.2, 23.0, 14.0.
HRMS $[C_{18}H_{18}O_2 + H]^+$ calculated, 249.1274; measured, 249.1270.

3-(benzyloxy)-2-methylpenta-3,4-dien-2-ol (3d).

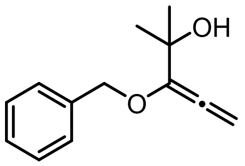

Prepared from the above procedure from benzoxyallene in 67% yield.
^{1}H NMR (500 MHz, CDCl$_3$) δ 7.39 – 7.28 (m, 5H), 5.55 (s, 2H), 4.65 (s, 2H), 2.26 (s, 1H), 1.40 (s, 6H).
^{13}C NMR (126 MHz, CDCl$_3$) δ 195.9, 138.3, 137.5, 128.4, 127.8, 127.7, 92.8, 77.3, 77.0, 76.8, 70.8, 70.4, 27.8.
HRMS $[C_{13}H_{16}O_2\text{-}(H_2O)+H]^+$ calculated, 187.1117; measured, 187.1118.z

3-(1-(benzyloxy)propa-1,2-dien-1-yl)-2-methyltetrahydrofuran-3-ol (3f).

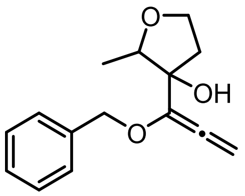

Prepared from the above procedure from benzoxyallene in 87% yield.
^{1}H NMR (500 MHz, $CDCl_3$) δ 7.40 – 7.27 (m, 5H), 5.67 – 5.59 (m, 2H), 4.65 (d, *J* = 3.7 Hz, 0H), 4.05 (q, *J* = 7.8 Hz, 1H), 3.92 (q, *J* = 6.3 Hz, 1H), 3.84 – 3.75 (m, 1H), 2.48 – 2.38 (m, 1H), 2.22 (d, *J* = 0.9 Hz, 1H), 2.09 (ddd, *J* = 13.1, 7.8, 5.2 Hz, 1H), 1.21 (d, *J* = 6.3 Hz, 3H).
^{13}C NMR (126 MHz, $CDCl_3$) δ 196.5, 137.1, 134.4, 128.5, 128.0, 127.7, 93.9, 81.4, 80.5, 79.2, 77.3, 77.0, 76.8, 71.0, 65.7, 38.9, 26.7, 23.3, 14.0, 13.5, 13.1.
HRMS $[C_{15}H_{18}O_3+H]^+$ calculated, 247.1334; measured, 247.1328.

The NMR data shows a mixture of diastereomers.

4-(1-(benzyloxy)propan-1,2-dien-1-yl)tetrahydro-2H-thiopyran-4-ol (3g).

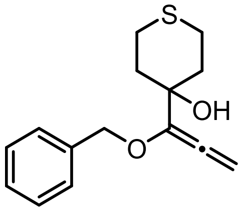

Prepared from the above procedure from benzoxyallene in 63% yield.
^{1}H NMR (500 MHz, $CDCl_3$) δ 7.39 – 7.29 (m, 6H), 5.59 (s, 1H), 4.63 (s, 1H), 2.98 (tt, *J* = 15.9, 9.0 Hz, 3H), 2.44 (tt, *J* = 14.0, 7.0 Hz, 3H), 2.01 (t, *J* = 7.1 Hz, 4H), 1.87 – 1.80 (m, 1H), 1.75 (td, *J* = 14.0, 3.6 Hz, 1H), 1.54 (s, 1H), 1.37 – 1.28 (m, 2H).
^{13}C NMR (126 MHz, $CDCl_3$) δ 196.5, 137.6, 137.2, 128.4, 128.4, 128.0, 127.9, 127.8, 127.7, 93.5, 77.3, 77.0, 76.8, 70.8, 70.2, 70.0, 44.1, 43.5, 38.2, 36.6, 30.1, 24.8, 24.4, 24.2, 23.2, 14.1.
HRMS $[C_{15}H_{18}O_2S+H]^+$ calculated, 263.1106; measured, 263.1099.

The NMR data shows minor inseparable impurities.

(5*R*)-1-(1-(benzyloxy)propa-1,2-dien-1-yl)-2-methyl-5-(prop-1-en-2-yl)cyclohex-2-en-1-ol (3h).

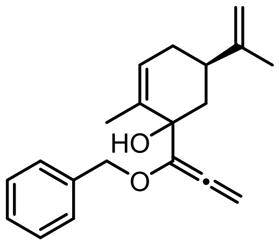

Prepared from the above procedure from benzoxyallene in 81% yield.
^{1}H NMR (500 MHz, $CDCl_3$) δ 7.40 – 7.28 (m, 5H), 5.61 – 5.55 (m, 1H), 5.53 (d, *J* = 7.9 Hz, 1H), 5.45 (d, *J* = 7.9 Hz, 1H), 4.76 – 4.61 (m, 4H), 2.68 (s, 1H), 2.37 (tq, *J* = 12.8, 4.9 Hz, 1H), 2.30 (dt, *J* = 12.4, 2.2 Hz, 1H), 2.07 (dtt, *J* = 17.4, 5.2, 1.7 Hz, 1H), 1.93 (ddp, *J* = 18.5, 10.7, 2.5 Hz, 1H), 1.73 (q, *J* = 2.7 Hz, 3H), 1.70 (s, 3H).
^{13}C NMR (126 MHz, $CDCl_3$) δ 197.7, 148.9, 137.4, 134.8, 134.6, 128.4, 127.8, 127.7, 126.5, 108.9, 92.1, 77.3, 77.0, 76.8, 75.3, 70.9, 40.9, 39.1, 31.0, 20.6, 17.7.

HRMS $[C_{20}H_{24}O_2\text{-}H_2O+H]^+$ calculated, 279.1776; measured, 279.1742.

The NMR data shows a mixture of diasteromers.

(2*S*,5*R*)-1-(1-(benzyloxy)propa-1,2-dien-1-yl)-2-isopropyl-5-methylcyclohexan-1-ol (3i).

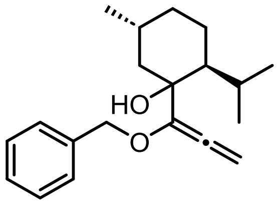

Prepared from the above procedure from benzoxyallene in 97% yield.
^{1}H NMR (500 MHz, CDCl$_3$) δ 7.42 – 7.28 (m, 5H), 5.68 – 5.57 (m, 2H), 4.62 (d, *J* = 4.2 Hz, 2H), 1.94 (p, *J* = 7.2 Hz, 1H), 1.75 (dt, *J* = 11.6, 2.5 Hz, 1H), 1.64 – 1.60 (m, 1H), 1.58 (d, *J* = 10.6 Hz, 1H), 1.55 – 1.44 (m, 3H), 1.11 (d, *J* = 7.2 Hz, 1H), 0.94 (dd, *J* = 16.1, 6.9 Hz, 3H), 0.90 – 0.85 (m, 6H).
^{13}C NMR (126 MHz, CDCl$_3$) δ 197.5, 197.0, 139.7, 139.5, 137.6, 137.5, 128.3, 127.8, 127.7, 127.7, 127.6, 94.1, 93.6, 77.3, 77.0, 76.8, 75.5, 71.0, 70.9, 47.3, 46.3, 46.1, 42.6, 35.0, 31.5, 28.0, 28.0, 27.4, 27.2, 24.2, 23.7, 22.3, 21.1, 21.0, 19.5, 18.7.
HRMS $[C_{20}H_{28}O_2+H]^+$ calculated, 301.2168; measured, 301.2157.

The NMR data shows a mixture of diasteromers.

(8*S*,9*S*,10*R*,13*R*,14*S*,17*R*)-3-(1-(benzyloxy)propa-1,2-dien-1-yl)-10,13-dimethyl-17-((*R*)-6-methylheptan-2-yl)-2,3,6,7,8,9,10,11,12,13,14,15,16,17-tetradecahydro-1H-cyclopenta[a]-phenanthren-3-ol (3j).

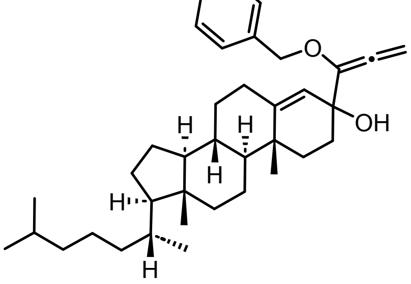

Prepared from the above procedure from benzoxyallene in 75% yield.
^{1}H NMR (500 MHz, CDCl$_3$) δ 7.39 – 7.29 (m, 5H), 5.48 (d, *J* = 7.7 Hz, 1H), 5.43 (d, *J* = 7.8 Hz, 1H), 5.25 (s, 1H), 2.56 (s, 1H), 2.25 – 2.15 (m, 1H), 2.11 – 1.93 (m, 3H), 1.86 – 1.77 (m, 1H), 1.70 (td, *J* = 13.6, 3.1 Hz, 2H), 1.59 (t, *J* = 3.8 Hz, 1H), 1.51 (dd, *J* = 13.3, 6.8 Hz, 1H), 1.44 – 1.20 (m, 12H), 1.12 (dtd, *J* = 19.5, 10.3, 4.1 Hz, 6H), 1.04 (s, 3H), 1.01 – 0.93 (m, 2H), 0.91 (d, *J* = 6.5 Hz, 3H), 0.87 (dd, *J* = 6.6, 2.2 Hz, 9H).
^{13}C NMR (126 MHz, CDCl$_3$) δ 197.9, 148.2, 137.5, 135.5, 128.4, 127.8, 127.7, 122.2, 91.8, 77.3, 77.0, 76.8, 72.2, 70.9, 56.3, 56.2, 54.3, 42.5, 39.9, 39.5, 37.4, 36.2, 36.0, 35.8, 34.8, 33.1, 32.4, 31.1, 28.2, 28.0, 24.2, 23.8, 22.8, 22.6, 21.1, 18.7, 18.6, 12.0.
HRMS $[C_{37}H_{54}O_2 + Na]^+$ calculated, 553.4016; measured, 553.4012.

(8*R*,9*S*,13*S*,14*S*)-17-(1-(benzyloxy)propa-1,2-dien-1-yl)-13-methyl-7,8,9,11,12,13,14,15,16,17-decahydro-6H-cyclopenta[a]phenanthrene-3,17-diol (3k).

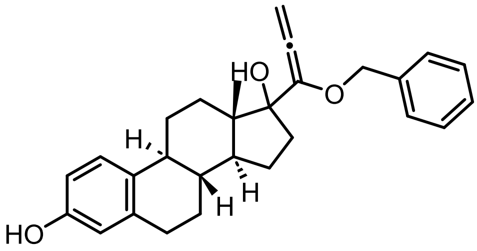

Prepared from the above procedure from benzoxyallene in 15% yield.

^{1}H NMR (500 MHz, CDCl$_3$) δ 7.44 – 7.32 (m, 7H), 7.14 (d, *J* = 8.4 Hz, 2H), 6.62 (dd, *J* = 8.4, 2.8 Hz, 1H), 6.56 (d, *J* = 2.8 Hz, 1H), 5.63 (d, *J* = 7.8 Hz, 1H), 5.56 (d, *J* = 7.8 Hz, 1H), 4.63 (q, *J* = 11.7 Hz, 2H), 2.91 – 2.76 (m, 3H), 2.28 (dt, *J* = 13.5, 3.5 Hz, 1H), 2.14 (td, *J* = 10.8, 4.4 Hz, 1H), 2.02 (qdd, *J* = 14.4, 9.9, 5.0 Hz, 3H), 1.88 (ddt, *J* = 12.4, 5.6, 2.5 Hz, 1H), 1.81 – 1.75 (m, 1H), 1.63 – 1.42 (m, 6H), 1.40 – 1.23 (m, 4H), 1.11 (d, *J* = 7.3 Hz, 1H), 0.94 (s, 3H).

^{13}C NMR (126 MHz, CDCl$_3$) δ 198.4, 153.5, 153.4, 138.3, 138.3, 137.1, 136.3, 132.8, 132.7, 128.5, 128.0, 127.0, 126.5, 115.3, 115.3, 112.7, 112.7, 92.1, 84.6, 84.5, 77.3, 77.0, 76.8, 71.0, 49.2, 48.3, 43.7, 39.5, 35.1, 34.0, 29.7, 27.6, 26.6, 22.9, 14.6

HRMS $[C_{28}H_{32}O_3+H]^+$ calculated, 417.2430; measured, 417.2421.

1-(1-(benzyloxy)propa-1,2-dien-1-yl)cyclobutan-1-ol (3l).

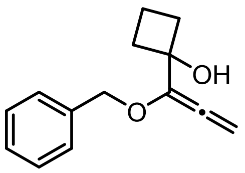

Prepared from the above procedure from benzoxyallene in 71% yield.

^{1}H NMR (500 MHz, CDCl$_3$) δ 7.36 – 7.20 (m, 5H), 5.59 (s, 2H), 4.69 (s, 2H), 2.61 (dt, *J* = 4.3, 1.4 Hz, 1H), 2.35 (dddt, *J* = 11.3, 9.1, 4.9, 2.5 Hz, 2H), 2.25 – 2.14 (m, 2H), 1.79 (qt, *J* = 11.0, 3.9 Hz, 1H), 1.57 (dp, *J* = 11.0, 8.7 Hz, 1H).

^{13}C NMR (126 MHz, CDCl$_3$) δ 196.2, 137.4, 135.8, 128.4, 127.9, 127.7, 92.5, 77.3, 77.0, 76.8, 73.8, 71.0, 34.6, 12.7.

HRMS $[C_{14}H_{16}O_2+H]^+$ calculated, 217.1229; measured, 217.1222.

The spectra are consistent with the reported literature values.[3]

***tert*-butyl 3-(1-(benzyloxy)propa-1,2-dien-1-yl)-3-hydroxyazetidine-1-carboxylate (3m).**

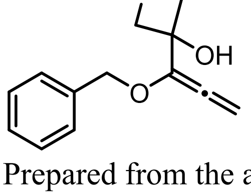

Prepared from the above procedure from benzoxyallene in 94% yield.

^{1}H NMR (500 MHz, CDCl$_3$) δ 7.42 – 7.28 (m, 5H), 5.68 (s, 2H), 4.69 (s, 2H), 4.12 (d, *J* = 9.2 Hz, 2H), 3.95 (dd, *J* = 9.3, 1.1 Hz, 2H), 2.74 (s, 1H), 1.43 (s, 9H).

^{13}C NMR (126 MHz, CDCl$_3$) δ 195.6, 156.4, 136.8, 133.7, 128.5, 128.1, 127.8, 94.7, 79.6, 77.3, 77.0, 76.8, 71.3, 69.0, 28.4.

HRMS $[C_{18}H_{23}NO_4 + Na]^+$ calculated, 340.1519; measured, 340.1513.

3-(1-(benzyloxy)propa-1,2-dien-1-yl)oxetan-3-ol (3n).

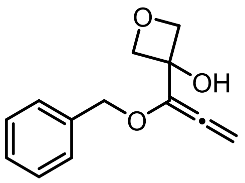

Prepared from the above procedure from benzoxyallene in 80% yield.
^{1}H NMR (500 MHz, CDCl$_3$) δ 7.39 – 7.29 (m, 6H), 5.71 (s, 2H), 4.75 (d, *J* = 6.7 Hz, 2H), 4.72 (s, 2H), 4.70 (d, *J* = 6.7 Hz, 2H), 2.80 (s, 1H).
^{13}C NMR (126 MHz, CDCl$_3$) δ 195.9, 136.8, 133.3, 128.5, 128.2, 127.9, 94.6, 82.0, 77.3, 77.0, 76.8, 73.1, 71.3.
HRMS $[C_{13}H_{14}O_3+H]^+$ calculated, 219.1021; measured, 219.1013.

((buta-2,3-dien-2-yloxy)methyl)benzene (3o).

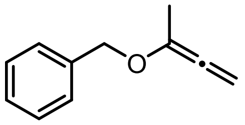

Prepared from the above procedure from benzoxyallene in 99% yield.
^{1}H NMR (500 MHz, CDCl$_3$) δ 7.36 – 7.27 (m, 6H), 5.35 (q, *J* = 2.9 Hz, 2H), 4.59 (s, 2H), 1.95 (t, *J* = 2.8 Hz, 3H).
^{13}C NMR (126 MHz, CDCl$_3$) δ 199.6, 137.8, 129.8, 128.4, 127.8, 127.7, 89.1, 77.3, 77.0, 76.8, 70.6, 18.5.
HRMS $[C_{11}H_{12}O + H]^+$ calculated, 161.0961; measured, 161.0961.

The spectra are consistent with the reported literature values.[4]

3-(benzyloxy)-6-methylhepta-1,2,5-trien-4-ol (3r).

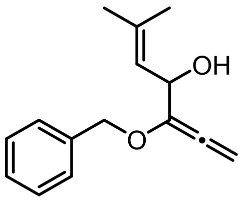

Prepared from the above procedure from benzoxyallene in 49% yield.
^{1}H NMR (500 MHz, CDCl$_3$) δ 7.39 – 7.28 (m, 5H), 5.53 (d, *J* = 1.8 Hz, 2H), 5.36 (dp, *J* = 8.6, 1.4 Hz, 1H), 4.95 (ddt, *J* = 8.7, 5.0, 2.0 Hz, 1H), 4.66 (s, 2H), 2.07 (d, *J* = 5.1 Hz, 1H), 1.76 (d, *J* = 1.5 Hz, 3H), 1.73 (s, 3H).
^{13}C NMR (126 MHz, CDCl$_3$) δ 197.2, 137.4, 137.3, 134.5, 128.4, 127.8, 127.7, 123.8, 92.3, 77.3, 77.0, 76.8, 70.8, 67.9, 25.8, 18.4.
HRMS $[C_{15}H_{18}O_2+H]^+$ calculated, 231.1385; measured, 231.1376.

***tert*-butyl 4-(2-(benzyloxy)-1-hydroxybuta-2,3-dien-1-yl)piperidine-1-carboxylate (3s).**

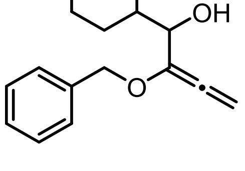

Prepared from the above procedure from benzoxyallene in 26% yield.
^{1}H NMR (500 MHz, CDCl$_3$) δ 7.38 – 7.29 (m, 5H), 5.57 – 5.50 (m, 2H), 4.64 (d, *J* = 1.4 Hz, 2H), 4.10 (s, 1H), 3.91 (t, *J* = 7.0 Hz, 1H), 2.66 (s, 2H), 1.91 – 1.86 (m, 1H), 1.86 – 1.74 (m, 2H), 1.59 – 1.57 (m, 1H), 1.54 (dt, *J* = 13.5, 2.7 Hz, 1H), 1.45 (s, 10H).
^{13}C NMR (126 MHz, CDCl$_3$) δ 197.7, 154.9, 137.2, 132.6, 128.4, 128.0, 127.9, 92.2, 79.3, 77.3, 77.0, 76.8, 75.2, 70.8, 60.4, 40.0, 28.5, 21.0, 14.2.
HRMS [$C_{21}H_{29}NO_4$+H]$^+$ calculated, 360.2175; measured, 360.2167.

2-(benzyloxy)-1-((1*S*,5*R*)-6,6-dimethylbicyclo[3.1.1]hept-2-en-3-yl)buta-2,3-dien-1-ol (3t).

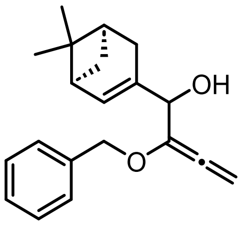

Prepared from the above procedure from benzoxyallene in 83% yield.
^{1}H NMR (500 MHz, CDCl$_3$) δ 7.36 – 7.27 (m, 5H), 5.60 (ddt, *J* = 6.1, 3.1, 1.5 Hz, 1H), 5.57 (d, *J* = 2.1 Hz, 2H), 4.64 – 4.55 (m, 3H), 2.38 (dq, *J* = 8.6, 5.9 Hz, 1H), 2.32 (dt, *J* = 18.0, 3.5 Hz, 1H), 2.29 – 2.22 (m, 2H), 2.11 – 2.06 (m, 1H), 2.00 (dd, *J* = 19.5, 6.3 Hz, 1H), 1.27 (d, *J* = 3.8 Hz, 3H), 1.18 (dd, *J* = 18.4, 8.6 Hz, 1H), 0.82 (d, *J* = 15.3 Hz, 3H).
^{13}C NMR (126 MHz, CDCl$_3$) δ 197.6, 146.8, 137.3, 133.3, 128.3, 127.8, 127.8, 120.1, 92.9, 77.3, 77.0, 76.8, 73.4, 70.8, 42.3, 42.3, 40.9, 37.8, 32.0, 31.3, 26.2, 21.2.
HRMS [$C_{20}H_{24}O_2$+H]$^+$ calculated, 297.1855; measured, 297.1842.

4-(benzyloxy)-2-((2,2,6,6-tetramethylpiperidin-1-yl)oxy)hexa-1,4,5-trien-3-ol (3u).

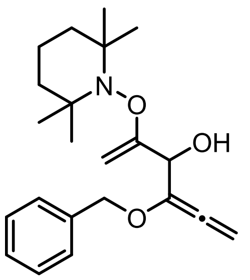

Prepared from the above procedure from benzoxyallene in 71% yield.
^{1}H NMR (500 MHz, CDCl$_3$) δ 7.34 – 7.26 (m, 5H), 5.62 (t, *J* = 1.8 Hz, 2H), 4.79 (s, 1H), 4.64 (s, 2H), 4.60 (dt, *J* = 9.3, 1.9 Hz, 1H), 4.28 (s, 1H), 2.43 (d, *J* = 9.4 Hz, 1H), 1.62 – 1.47 (m, 6H), 1.35 (dq, *J* = 12.2, 3.4 Hz, 1H), 1.11 (d, *J* = 6.3 Hz, 6H), 1.05 (d, *J* = 14.3 Hz, 7H).
^{13}C NMR (126 MHz, CDCl$_3$) δ 197.8, 162.5, 137.2, 133.2, 128.3, 127.9, 127.8, 92.9, 85.8, 77.3, 77.0, 76.8, 71.4, 70.9, 60.5, 60.4, 39.7, 39.7, 32.4, 32.3, 20.4, 20.3, 17.0.
HRMS [$C_{22}H_{31}NO_3$+H]$^+$ calculated, 358.2382; measured, 358.2369.

Synthesis of 2-(hexa-1,2-dien-1-yloxy)tetrahydro-2H-pyran (7a).

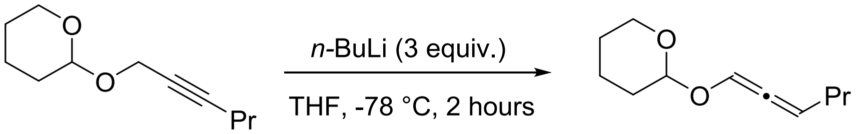

In a round-bottom flask, 2-(hex-2-yn-1-yloxy)tetrahydro-2H-pyran **6v** (2.00 mmol) was dissolved in anhydrous THF (4 mL) and cooled to -78 °C. *n*-BuLi (6.00 mmol, 3 equiv.) was added and the

reaction mixture was stirred for 2 hours at the same temperature and quenched with addition of H_2O. The mixture was extracted with DCM (3x), dried over $MgSO_4$, filtered, and evaporated under reduced pressure. The mixture was then purified via column chromatography (Hex:EA 9:1) to yield 2-(hexa-1,2-dien-1-yloxy)tetrahydro-2H-pyran **7v** in 11% yield.
^{1}H NMR (500 MHz, CDCl$_3$) δ 6.57 (dq, J = 6.0, 2.1 Hz, 1H), 5.78 (dq, J = 31.6, 6.3 Hz, 1H), 4.92 (q, J = 3.2 Hz, 1H), 3.92 – 3.86 (m, 1H), 3.56 (dddd, J = 15.6, 10.0, 4.2, 1.7 Hz, 1H), 2.11 – 2.03 (m, 2H), 1.93 – 1.84 (m, 1H), 1.78 – 1.68 (m, 2H), 1.64 – 1.53 (m, 3H), 1.51 – 1.45 (m, 2H), 0.94 (td, J = 7.4, 3.1 Hz, 3H).
^{13}C NMR (126 MHz, CDCl$_3$) δ 194.0, 193.7, 117.5, 117.4, 105.5, 105.4, 97.5, 97.3, 77.3, 77.0, 76.8, 62.1, 62.0, 33.0, 32.9, 29.9, 25.2, 25.2, 21.8, 21.7, 18.9, 18.8, 13.7, 13.7.
HRMS $[C_{11}H_{18}O_2+H]^+$ calculated, 183.1380; measured, 183.1381.
The spectra are consistent with the reported literature values.[5]

Synthesis of 2,2'-(buta-1,2-diene-1,4-diylbis(oxy))bis(tetrahydro-2H-pyran) (7b).

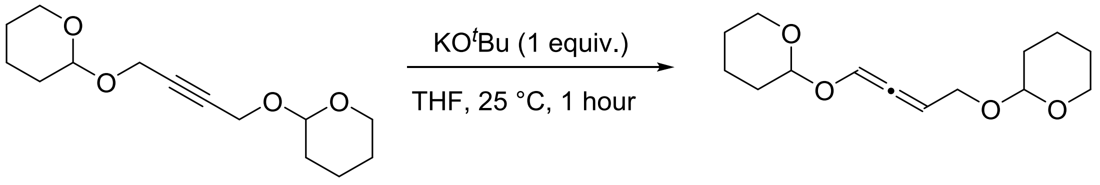

In a round-bottom flask, 1,4-bis((tetrahydro-2H-pyran-2-yl)oxy)but-2-yne **6w** (2.00 mmol) was dissolved in THF (4 mL) and KO^{t}Bu (2.00 mmol, 1 equiv.) was added and the reaction mixture was stirred for 1 hour. The mixture was passed through a silica plug with Et_2O, evaporated under reduced pressure, and the mixture was purified via column chromatography (Hex:EA 4:1) to yield 2,2'-(buta-1,2-diene-1,4-diylbis(oxy))bis(tetrahydro-2H-pyran) **7w** in 20% yield.
^{1}H NMR (500 MHz, CDCl$_3$) δ 6.73 – 6.67 (m, 2H), 5.94 – 5.88 (m, 1H), 5.83 (dt, J = 7.0, 5.3 Hz, 1H), 4.94 (dt, J = 10.1, 3.3 Hz, 2H), 4.81 (t, J = 3.5 Hz, 1H), 4.70 (p, J = 3.7 Hz, 2H), 4.37 – 4.32 (m, 1H), 4.30 – 4.23 (m, 3H), 4.12 – 4.05 (m, 2H), 3.86 (dddd, J = 19.7, 11.6, 7.7, 3.2 Hz, 5H), 3.60 – 3.49 (m, 5H), 1.84 (dp, J = 15.3, 4.3 Hz, 5H), 1.77 – 1.69 (m, 7H), 1.63 (dd, J = 9.0, 4.3 Hz, 5H), 1.58 – 1.50 (m, 12H).
^{13}C NMR (126 MHz, CDCl$_3$) δ 195.5, 195.4, 195.4, 118.8, 118.7, 102.6, 102.6, 102.6, 97.8, 97.8, 97.6, 97.5, 97.5, 96.9, 96.8, 82.0, 77.3, 77.0, 76.8, 65.9, 65.8, 62.2, 62.1, 62.0, 61.9, 54.4, 30.6, 30.5, 30.3, 29.8, 29.7, 25.5, 25.4, 25.2, 19.4, 19.1, 18.7, 18.6.
HRMS $[C_{14}H_{22}O_4 + Na]^+$ calculated, 277.1410; measured, 277.1405.
The NMR data shows minor inseparable impurities.

IV. Allene Dioxygenation Reactions.

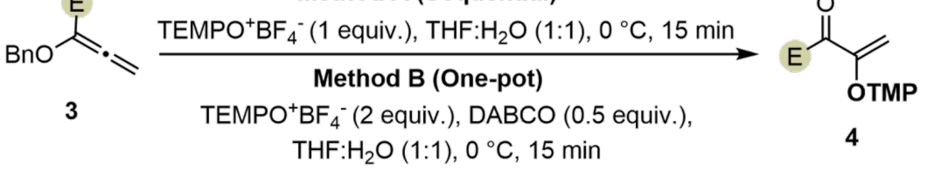

General Procedures for Allene dioxygenation

Method A: To a round-bottom flask with allene **3** in 1:1 THF:H_2O (0.025 M) was added $TEMPO^+BF_4^-$ (1 equiv.) in one portion at 0 °C and the reaction mixture was allowed to stir at 0 °C for 15 minutes. The solution was diluted with both DCM and sat. NH_4Cl*, then transferred to a separatory funnel. The aqueous layer was washed with DCM (3x), dried over $MgSO_4$, filtered, and

concentrated via rotary evaporation. The crude mixture was purified via column chromatography (Hex:EA) to yield the desired dioxygenation product.

Method B: To a round-bottom flask with allene **3** in THF (0.1 M) that was stirring with *n*-BuLi (1.2 equiv.) over 16 hours was added THF and water to achieve a 1:1 ratio of THF:H_2O (0.025 M). The reaction vessel was cooled to 0 °C, and DABCO (0.5 equiv.) followed by $TEMPO^+BF_4^-$ (2 equiv.) was added and stirred at this temperature for 15 minutes. The solution was diluted with both DCM and 1M HCl*, then transferred to a separatory funnel. The aqueous layer was washed with DCM (3x), dried over $MgSO_4$, filtered, and concentrated via rotary evaporation. The crude mixture was purified via column chromatography (Hex:EA) to yield the desired dioxygenation product.

*Acid workup necessary to convert epoxide intermediates to desired products for aldehyde and ketone adducts.

TEMPO-acrolein (2).

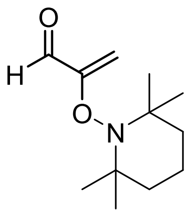

To a solution of allene **1** (0.4 mmol) in 1:1 H_2O:THF (0.025 M) in a round-bottom flask was added $TEMPO^+BF_4^-$ (1 equiv.) in one portion at 0 °C and the reaction mixture was allowed to stir at 0 °C for 15 minutes. The solution was diluted with both DCM and H_2O, then transferred to a separatory funnel. The aqueous layer was washed with DCM (3x), dried over $MgSO_4$, filtered, and concentrated via rotary evaporation. The crude mixture was purified via column chromatography (98:2 Hex:EA) to yield the desired dioxygenation product **2** in 95% yield.

Note: Product is an amorphous/viscous oil containing residual solvent which spontaneously crystallizes on standing, the process is accelerated by agitation and lower temperatures. To remove excess solvent, the reaction vessel with the compound was frozen (either ice bath or dry ice) and concentrated via rotary evaporation while frozen (2x).

^{1}H NMR (500 MHz, CDCl$_3$) δ 9.24 (s, 1H), 5.89 (s, 1H), 5.10 (s, 1H), 1.70 – 1.58 (m, 1H), 1.56 – 1.52 (m, 4H), 1.38 (dp, *J* = 12.5, 3.2 Hz, 1H), 1.21 (s, 6H), 1.01 (s, 6H).

^{13}C NMR (126 MHz, CDCl$_3$) δ 187.1, 162.2, 108.3, 60.8, 39.7, 32.4, 20.5, 16.9.

HRMS $[C_{12}H_{21}NO_2 + H]^+$ calculated, 212.1645; measured, 212.1645.

1-(1-hydroxycyclopentyl)-2-((2,2,6,6-tetramethylpiperidin-1-yl)oxy)prop-2-en-1-one (4a).

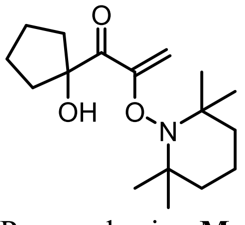

Prepared using **Method B** from allene **1** in 54% yield.

^{1}H NMR (500 MHz, CDCl$_3$) δ 5.50 (s, 1H), 5.44 (s, 1H), 2.39 (dtd, *J* = 13.1, 7.2, 1.9 Hz, 2H), 1.95 (dtd, *J* = 11.9, 7.5, 3.0 Hz, 2H), 1.87 – 1.79 (m, 2H), 1.62 – 1.51 (m, 5H), 1.47 – 1.38 (m, 1H), 1.20 (s, 6H), 1.07 (s, 7H).

^{13}C NMR (126 MHz, CDCl$_3$) δ 198.3, 160.6, 99.3, 86.5, 77.9, 77.8, 77.7, 77.5, 77.4, 77.3, 60.8, 40.0, 38.5, 32.4, 25.4, 21.4, 16.9.

HRMS [$C_{17}H_{29}NO_3$+H]$^+$ calculated, 296.2226; measured, 296.2217.

1-(1-hydroxycyclohexyl)-2-((2,2,6,6-tetramethylpiperidin-1-yl)oxy)prop-2-en-1-one (4b).

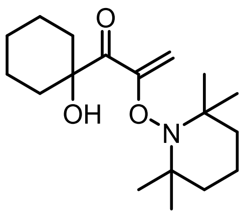

Prepared using **Method B** from allene **1** in 74% yield.
^{1}H NMR (500 MHz, CDCl$_3$) δ 5.40 (d, *J* = 4.2 Hz, 2H), 3.87 (s, 1H), 2.17 (td, *J* = 12.9, 4.5 Hz, 2H), 1.78 – 1.70 (m, 3H), 1.67 – 1.61 (m, 3H), 1.58 (s, 6H), 1.45 – 1.38 (m, 1H), 1.35 – 1.27 (m, 1H), 1.24 (s, 6H), 1.08 (s, 6H).
^{13}C NMR (126 MHz, CDCl$_3$) δ 199.5, 161.1, 99.0, 77.9, 77.3, 77.0, 76.8, 60.8, 39.9, 33.0, 32.4, 25.5, 21.5, 21.2, 16.9.
HRMS [$C_{18}H_{31}NO_3$+H]$^+$ calculated, 310.2382; measured, 310.2371.

4-hydroxy-4-phenyl-2-((2,2,6,6-tetramethylpiperidin-1-yl)oxy)pent-1-en-3-one (4c).

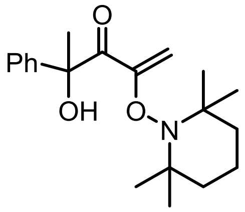

Prepared using **Method A** from allene **3c** in 45% yield.
^{1}H NMR (500 MHz, CDCl$_3$) δ 7.28 (d, *J* = 4.3 Hz, 4H), 7.17 (dd, *J* = 6.2, 2.6 Hz, 1H), 5.46 (s, 1H), 5.31 (s, 1H), 4.45 (s, 1H), 1.70 (s, 3H), 1.46 – 1.32 (m, 6H), 1.28 – 1.22 (m, 1H), 0.75 – 0.66 (m, 13H).
^{13}C NMR (126 MHz, CDCl$_3$) δ 195.6, 160.0, 142.4, 128.8, 127.7, 124.8, 99.8, 79.5, 77.3, 77.0, 76.8, 60.5, 60.5, 39.8, 39.8, 31.9, 31.9, 24.9, 20.9, 20.7, 16.8.
HRMS [$C_{20}H_{29}NO_3$+H]$^+$ calculated, 332.2220; measured, 332.2218.

4-hydroxy-4-methyl-2-((2,2,6,6-tetramethylpiperidin-1-yl)oxy)pent-1-en-3-one (4d).

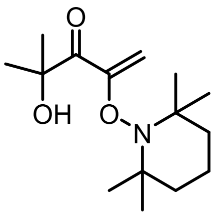

Prepared using **Method A** from allene **3d** in 51% yield.
^{1}H NMR (500 MHz, CDCl$_3$) δ 5.47 (s, 1H), 5.44 (s, 1H), 4.14 (s, 1H), 1.63 – 1.57 (m, 5H), 1.44 – 1.40 (m, 1H), 1.22 (s, 7H), 1.07 (s, 6H).
^{13}C NMR (126 MHz, CDCl$_3$) δ 199.5, 160.3, 99.3, 77.3, 77.0, 76.8, 75.8, 60.8, 39.9, 32.3, 26.2, 25.7, 21.5, 16.9.
HRMS [$C_{15}H_{27}NO_3$+H]$^+$ calculated, 270.2069; measured, 270.2060.

tert-butyl 4-hydroxy-4-(2-((2,2,6,6-tetramethylpiperidin-1-yl)oxy)acryloyl)piperidine-1-carboxylate (4e).

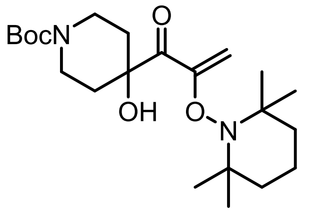

Prepared using **Method B** from allene **1** in 54% yield.
^{1}H NMR (500 MHz, CDCl$_3$) δ 5.46 (d, *J* = 11.1 Hz, 2H), 4.13 (s, 1H), 4.04 (s, 2H), 3.21 (s, 2H), 2.38 (s, 2H), 1.62 – 1.54 (m, 5H), 1.45 (s, 11H), 1.43 – 1.38 (m, 1H), 1.20 (s, 6H), 1.05 (s, 6H).
^{13}C NMR (126 MHz, CDCl$_3$) δ 197.7, 160.5, 154.4, 99.9, 79.3, 77.3, 77.0, 76.8, 76.3, 60.9, 39.9, 32.8, 32.4, 28.5, 21.6, 16.8.
HRMS $[C_{22}H_{38}N_2O_5+H]^+$ calculated, 411.2859; measured, 411.2846.

1-(3-hydroxy-2-methyltetrahydrofuran-3-yl)-2-((2,2,6,6-tetramethylpiperidin-1-yl)oxy)prop-2-en-1-one (4f).

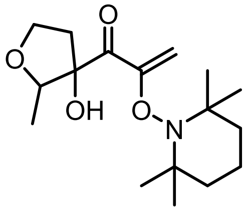

Prepared using **Method A** from allene **3f** in 49% yield.
^{1}H NMR (500 MHz, CDCl$_3$) δ 5.52 (s, 1H), 5.48 (s, 1H), 4.51 (q, *J* = 6.3 Hz, 1H), 4.19 (td, *J* = 8.1, 6.6 Hz, 1H), 4.07 (s, 1H), 4.02 (td, *J* = 8.7, 5.4 Hz, 1H), 2.94 (ddd, *J* = 13.0, 9.1, 6.6 Hz, 1H), 2.09 (ddd, *J* = 13.2, 7.9, 5.4 Hz, 1H), 1.60 (pd, *J* = 8.6, 3.1 Hz, 5H), 1.42 (dq, *J* = 10.2, 3.2 Hz, 1H), 1.22 (d, *J* = 4.9 Hz, 6H), 1.16 (d, *J* = 6.3 Hz, 3H), 1.09 (s, 3H), 1.04 (s, 3H).
^{13}C NMR (126 MHz, CDCl$_3$) δ 196.5, 160.2, 100.1, 86.0, 79.6, 77.3, 77.0, 76.8, 66.6, 61.0, 60.8, 40.0, 39.1, 32.4, 21.5, 21.5, 16.8, 13.4.
HRMS $[C_{17}H_{29}NO_4+H]^+$ calculated, 312.2175; measured, 312.2166.

1-(4-hydroxytetrahydro-2H-thiopyran-4-yl)-2-((2,2,6,6-tetramethylpiperidin-1-yl)oxy)prop-2-en-1-one (4g).

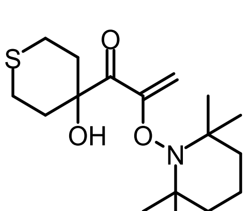

Prepared using **Method A** from allene **3g** in 58% yield.
^{1}H NMR (500 MHz, CDCl$_3$) δ 5.46 (s, 2H), 4.00 (s, 1H), 3.19 (ddd, *J* = 13.6, 12.6, 2.6 Hz, 2H), 2.60 (td, *J* = 13.0, 3.8 Hz, 2H), 2.48 – 2.43 (m, 2H), 1.79 – 1.73 (m, 2H), 1.70 – 1.63 (m, 1H), 1.60 (ddd, *J* = 6.9, 4.8, 3.3 Hz, 4H), 1.42 (dq, *J* = 12.2, 3.0 Hz, 1H), 1.29 (s, 6H), 1.07 (s, 6H).
^{13}C NMR (126 MHz, CDCl$_3$) δ 198.1, 160.4, 99.9, 77.3, 77.0, 76.8, 76.4, 60.9, 39.9, 33.6, 32.4, 23.4, 21.7, 16.9.
HRMS $[C_{17}H_{29}NO_3S +H]^+$ calculated, 328.1947; measured, 328.1938.

1-((5R)-1-hydroxy-2-methyl-5-(prop-1-en-2-yl)cyclohex-2-en-1-yl)-2-((2,2,6,6-tetramethylpiperidin-1-yl)oxy)prop-2-en-1-one (4h).

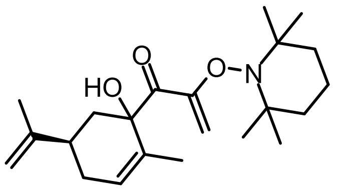

Prepared using **Method A** from allene **3h** in 66% yield.
^{1}H NMR (500 MHz, CDCl$_3$) δ 5.78 (dtt, *J* = 5.6, 4.0, 2.0 Hz, 1H), 5.46 (dd, *J* = 7.2, 0.9 Hz, 2H), 4.75 (q, *J* = 1.7 Hz, 1H), 4.71 – 4.68 (m, 1H), 2.43 – 2.36 (m, 2H), 2.24 (dtt, *J* = 17.4, 5.0, 1.7 Hz, 1H), 2.09 (ddt, *J* = 17.4, 9.1, 2.7 Hz, 1H), 1.89 – 1.82 (m, 1H), 1.72 (d, *J* = 1.0 Hz, 3H), 1.65 (t, *J* = 1.9 Hz, 3H), 1.64 – 1.60 (m, 1H), 1.58 (dt, *J* = 9.8, 4.0 Hz, 4H), 1.41 (dq, *J* = 10.9, 3.1 Hz, 1H), 1.21 (d, *J* = 13.4 Hz, 6H), 1.14 (s, 1H), 1.11 (s, 3H), 1.07 – 1.01 (m, 4H).
^{13}C NMR (126 MHz, CDCl$_3$) δ 197.0, 160.9, 148.0, 133.3, 126.7, 126.1, 109.4, 109.3, 99.9, 99.4, 80.1, 77.3, 77.0, 76.8, 60.9, 60.9, 40.0, 39.9, 39.9, 38.6, 38.1, 37.6, 36.6, 32.4, 32.4, 32.3, 30.9, 30.0, 21.3, 21.1, 21.0, 21.0, 20.9, 18.9, 18.3, 16.9, 16.8.
HRMS $[C_{22}H_{35}NO_3+H]^+$ calculated, 362.2695; measured, 362.2685.

The NMR data shows a mixture of diasteromers.

1-((2S,5R)-1-hydroxy-2-isopropyl-5-methylcyclohexyl)-2-((2,2,6,6-tetramethylpiperidin-1-yl)oxy)prop-2-en-1-one (4i).

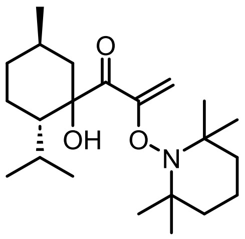

Prepared using **Method A** from allene **3i** in 69% yield.
^{1}H NMR (500 MHz, CDCl$_3$) δ 5.49 (s, 1H), 5.44 (s, 1H), 3.96 (s, 1H), 2.23 (dq, *J* = 12.4, 3.8 Hz, 1H), 2.09 – 1.97 (m, 2H), 1.94 – 1.80 (m, 2H), 1.69 – 1.57 (m, 9H), 1.38 (dddd, *J* = 24.9, 12.8, 9.4, 6.5 Hz, 4H), 1.25 (s, 9H), 1.08 (d, *J* = 11.3 Hz, 9H), 0.96 – 0.82 (m, 17H).
^{13}C NMR (126 MHz, CDCl$_3$) δ 201.1, 161.1, 99.5, 82.7, 77.3, 77.0, 76.8, 75.2, 60.8, 60.7, 47.7, 46.9, 45.8, 45.8, 43.8, 41.1, 40.1, 40.0, 35.2, 35.0, 32. 3, 32.3, 29.7, 29.4, 29.3, 28.1, 27.6, 26.2, 25.5, 23.6, 23.5, 22.9, 22.3, 21.8, 21.6, 21.5, 21.4, 20.5, 19.1, 18.2, 16.8.
HRMS $[C_{22}H_{39}NO_3+H]^+$ calculated, 366.3008; measured, 366.3000.

The NMR data shows a mixture of diasteromers.

1-((8S,9S,10R,13R,14S,17R)-3-hydroxy-10,13-dimethyl-17-((R)-6-methylheptan-2-yl)-2,3,6,7,8,9,10,11,12,13,14,15,16,17-tetradecahydro-1H-cyclopenta[a]phenanthren-3-yl)-2-((2,2,6,6-tetramethylpiperidin-1-yl)oxy)prop-2-en-1-one (4j).

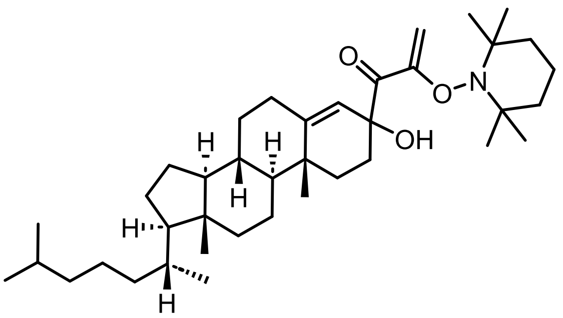

Prepared using **Method A** from allene **3j** in 57% yield.
^{1}H NMR (500 MHz, $CDCl_3$) δ 5.50 (d, *J* = 1.6 Hz, 1H), 5.47 (s, 1H), 5.45 (s, 1H), 3.79 (s, 1H), 2.33 – 2.21 (m, 2H), 2.06 (ddd, *J* = 13.8, 4.3, 2.4 Hz, 1H), 1.97 (dt, *J* = 12.7, 3.5 Hz, 1H), 1.81 (dddd, *J* = 13.0, 9.3, 7.2, 2.4 Hz, 1H), 1.71 (dddd, *J* = 27.8, 14.7, 6.7, 4.4 Hz, 3H), 1.64 – 1.30 (m, 18H), 1.26 (s, 5H), 1.21 (s, 4H), 1.15 – 1.03 (m, 16H), 0.92 – 0.78 (m, 13H), 0.67 (s, 3H).
^{13}C NMR (126 MHz, $CDCl_3$) δ 194.5, 160.9, 152.2, 119.5, 99.1, 77.3, 77.0, 76.9, 76.8, 60.9, 60.8, 56.2, 56.0, 53.1, 42.5, 40.0, 39.9, 39.8, 39.5, 37.3, 36.2, 36.0, 35.8, 33.7, 33.0, 32.8, 32.4, 32.3, 29.4, 28.2, 28.0, 24.2, 23.9, 22.8, 22.6, 21.5, 21.4, 21.2, 19.1, 18.6, 16.9, 12.0.
HRMS $[C_{39}H_{65}NO_3+H]^+$ calculated, 596.5043; measured, 596.5034.

1-((8R,9S,13S,14S)-3,17-dihydroxy-13-methyl-7,8,9,11,12,13,14,15,16,17-decahydro-6H-cyclopenta[a]phenanthren-17-yl)-2-((2,2,6,6-tetramethylpiperidin-1-yl)oxy)prop-2-en-1-one (4k).

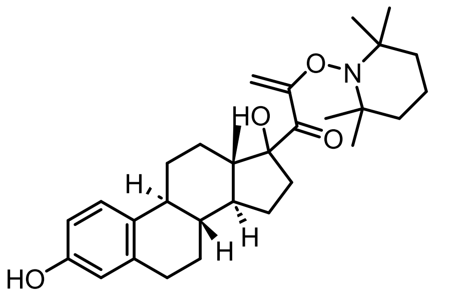

Prepared using **Method A** from allene **3k** in 89% yield.
^{1}H NMR (500 MHz, $CDCl_3$) δ 7.10 (d, *J* = 8.3 Hz, 1H), 6.62 – 6.53 (m, 2H), 5.40 (d, *J* = 8.4 Hz, 2H), 4.40 (s, 1H), 2.80 (h, *J* = 6.9 Hz, 2H), 2.37 (ddd, *J* = 13.9, 9.3, 6.5 Hz, 1H), 2.31 – 2.23 (m, 1H), 2.11 (td, *J* = 11.3, 4.3 Hz, 1H), 2.03 (dt, *J* = 12.3, 3.2 Hz, 1H), 1.60 (s, 14H), 1.47 (dtd, *J* = 24.3, 12.4, 5.0 Hz, 6H), 1.38 – 1.29 (m, 3H), 1.25 (d, *J* = 12.4 Hz, 8H), 1.14 (d, *J* = 14.0 Hz, 6H), 1.01 (s, 3H).
^{13}C NMR (126 MHz, $CDCl_3$) δ 200.1, 163.0, 153.4, 138.4, 132.6, 126.5, 115.3, 112.6, 98.9, 92.1, 77.3, 77.0, 76.8, 68.0, 61.2, 61.0, 49.3, 48.9, 43.1, 40.3, 40.2, 39.3, 35.7, 34.0, 32.3, 32.2, 29.7, 29.7, 27.3, 26.5, 25.6, 23.8, 21.5, 21.4, 16.8, 14.5.
HRMS $[C_{30}H_{43}NO_4-H]^-$ calculated, 480.3114; measured, 480.3116.

2-hydroxy-2-(1-((2,2,6,6-tetramethylpiperidin-1-yl)oxy)vinyl)cyclopentan-1-one (5a).

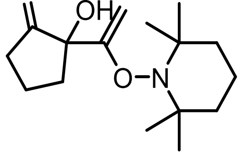

Prepared using **Method A** from allene **3l** and obtained via column chromatography of **4l** with silica in 68% yield.

^{1}H NMR (500 MHz, CDCl$_3$) δ 4.91 (d, *J* = 1.3 Hz, 1H), 4.35 (d, *J* = 1.4 Hz, 1H), 2.70 (s, 1H), 2.54 – 2.34 (m, 3H), 2.09 – 1.95 (m, 3H), 1.63 – 1.58 (m, 1H), 1.56 – 1.47 (m, 5H), 1.36 (ddt, *J* = 10.6, 5.2, 2.2 Hz, 1H), 1.15 (s, 3H), 1.06 (d, *J* = 6.5 Hz, 6H), 1.01 (s, 3H).
^{13}C NMR (126 MHz, CDCl$_3$) δ 217.5, 164.2, 85.0, 78.9, 77.3, 77.0, 76.8, 60.7, 60.6, 39.8, 39.8, 36.2, 35.2, 32.4, 32.1, 20.8, 20.7, 17.9, 16.9.
HRMS [$C_{16}H_{27}NO_3$+H]$^+$ calculated, 282.2069; measured, 282.2060.

tert-butyl 3-hydroxy-4-oxo-3-(1-((2,2,6,6-tetramethylpiperidin-1-yl)oxy)vinyl)pyrrolidine-1-carboxylate (5b).

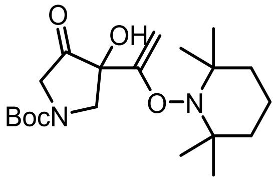

Prepared using **Method A** from allene **3m** obtained via column chromatography of **4m** with silica in 60% yield.
^{1}H NMR (500 MHz, CDCl$_3$) δ 4.94 (d, *J* = 1.7 Hz, 1H), 4.45 – 4.28 (m, 2H), 4.12 (dd, *J* = 36.9, 19.8 Hz, 1H), 3.85 (d, *J* = 19.6 Hz, 1H), 3.53 (d, *J* = 12.0 Hz, 1H), 2.93 (s, 1H), 1.63 – 1.50 (m, 6H), 1.48 (d, *J* = 3.9 Hz, 9H), 1.36 (dq, *J* = 10.7, 3.1 Hz, 1H), 1.12 (s, 3H), 1.07 (s, 3H), 1.04 (s, 3H), 0.97 (s, 3H).
^{13}C NMR (126 MHz, CDCl$_3$) δ 210.3, 163.0, 153.8, 85.3, 80.6, 77.3, 77.0, 76.8, 60.8, 60.8, 54.1, 53.3, 52.0, 51.6, 39.8, 39.7, 32.2, 32.1, 28.4, 20.9, 20.7, 16.9.
HRMS [$C_{20}H_{34}N_2O_5$+H]$^+$ calculated, 383.2546; measured, 383.2537.

4-hydroxy-4-(1-((2,2,6,6-tetramethylpiperidin-1-yl)oxy)vinyl)dihydrofuran-3(2H)-one (5c).

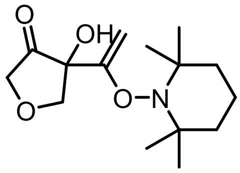

Prepared using **Method A** from allene **3n** obtained via column chromatography of **4n** with silica in 52% yield.
^{1}H NMR (500 MHz, CDCl$_3$) δ 4.99 (d, *J* = 1.5 Hz, 1H), 4.50 (d, *J* = 10.1 Hz, 1H), 4.47 (d, *J* = 1.5 Hz, 1H), 4.19 (dd, *J* = 17.4, 0.9 Hz, 1H), 4.12 (dd, *J* = 17.4, 0.9 Hz, 1H), 4.07 (dd, *J* = 10.1, 0.8 Hz, 1H), 2.74 (s, 1H), 1.53 (s, 6H), 1.39 – 1.34 (m, 1H), 1.16 (s, 3H), 1.07 (s, 3H), 1.03 (d, *J* = 8.0 Hz, 6H).
^{13}C NMR (126 MHz, CDCl$_3$) δ 213.1, 161.8, 86.1, 77.3, 77.0, 76.8, 75.9, 70.3, 60.8, 60.8, 39.8, 39.7, 32.4, 32.1, 20.7, 20.6, 16.9.
HRMS [$C_{15}H_{25}NO_4$+H]$^+$ calculated, 284.1862; measured, 284.1851.

3-((2,2,6,6-tetramethylpiperidin-1-yl)oxy)but-3-en-2-one (4o).

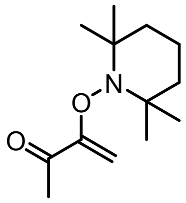

Prepared using **Method A** from allene **3o** in 74% yield.

^{1}H NMR (500 MHz, $CDCl_3$) δ 5.24 (s, 1H), 5.16 (s, 1H), 2.33 (s, 3H), 1.59 – 1.50 (m, 6H), 1.21 (s, 6H), 1.03 (s, 6H).
^{13}C NMR (126 MHz, $CDCl_3$) δ 195.0, 161.0, 95.0, 77.3, 77.0, 76.8, 60.7, 39.6, 32.5, 26.7, 20.7, 16.9.
HRMS $[C_{13}H_{23}NO_2+H]^+$ calculated, 226.1807; measured, 226.1799.

1-hydroxy-1-phenyl-3-((2,2,6,6-tetramethylpiperidin-1-yl)oxy)but-3-en-2-one (4p).

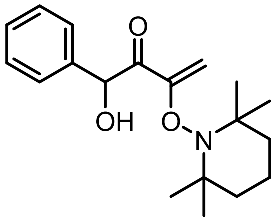

Prepared using **Method B** from allene **1** in 37% yield.
^{1}H NMR (500 MHz, $CDCl_3$) δ 7.38 – 7.33 (m, 2H), 7.32 – 7.29 (m, 1H), 7.29 – 7.26 (m, 2H), 5.57 (d, J = 6.1 Hz, 1H), 5.48 (s, 1H), 4.11 (d, J = 6.9 Hz, 1H), 1.59 – 1.50 (m, 1H), 1.47 – 1.25 (m, 4H), 1.15 (s, 3H), 0.96 (s, 3H), 0.68 (s, 3H), 0.58 (s, 3H).
^{13}C NMR (126 MHz, $CDCl_3$) δ 195.7, 158.9, 138.1, 129.0, 128.6, 128.1, 98.5, 77.3, 77.1, 76.8, 60.8, 60.4, 39.7, 39.6, 32.5, 31.5, 20.9, 20.5, 16.8.
HRMS $[C_{19}H_{27}NO_3+H]^+$ calculated, 318.2069; measured, 318.2058.

4-hydroxy-6,10-dimethyl-2-((2,2,6,6-tetramethylpiperidin-1-yl)oxy)undeca-1,9-dien-3-one (4q).

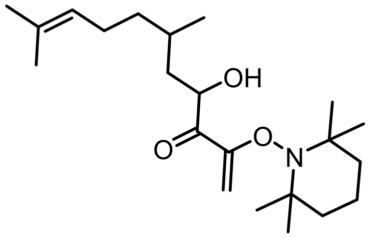

Prepared using **Method B** from allene **1** in 60% yield.
^{1}H NMR (500 MHz, $CDCl_3$) δ 5.42 (d, J = 9.5 Hz, 1H), 5.35 (d, J = 5.8 Hz, 1H), 5.12 – 5.08 (m, 2H), 4.75 (dtd, J = 9.3, 7.0, 2.3 Hz, 1H), 3.69 (tt, J = 5.7, 3.0 Hz, 1H), 3.27 (dd, J = 29.5, 7.3 Hz, 1H), 1.98 (dh, J = 15.1, 6.8 Hz, 6H), 1.88 – 1.84 (m, 1H), 1.68 (d, J = 2.9 Hz, 9H), 1.64 – 1.52 (m, 15H), 1.48 – 1.28 (m, 13H), 1.27 – 1.18 (m, 11H), 1.07 (s, 3H), 1.04 (d, J = 6.6 Hz, 2H), 1.00 (d, J = 1.3 Hz, 3H), 0.97 (d, J = 6.3 Hz, 2H), 0.91 (td, J = 7.0, 1.9 Hz, 7H).
^{13}C NMR (126 MHz, $CDCl_3$) δ 199.1, 199.0, 159.1, 159.1, 131.3, 131.3, 131.3, 131.2, 124.8, 124.6, 124.6, 98.0, 98.0, 77.3, 77.0, 76.8, 72.6, 72.1, 70.0, 69.7, 60.9, 60.7, 45.2, 45.0, 42.1, 41.5, 39.8, 39.6, 39.6, 38.1, 38.0, 37.5, 36.7, 36.3, 32.5, 32.4, 32.4, 29.9, 29.7, 29.3, 29.2, 28.9, 27.9, 27.8, 25.7, 25.6, 25.5, 25.4, 25.4, 22.8, 21.1, 21.1, 20.8, 20.4, 20.3, 19.2, 18.4, 17.7, 17.7, 17.7, 16.9, 14.1.
HRMS $[C_{22}H_{39}NO_3+H]^+$ calculated, 366.3008; measured, 366.2996.

The NMR data shows a mixture of diastereomers.

4-hydroxy-6-methyl-2-((2,2,6,6-tetramethylpiperidin-1-yl)oxy)hepta-1,5-dien-3-one (4r).

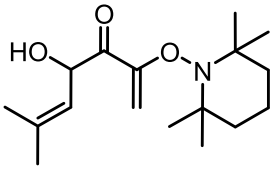

Prepared using **Method A** from allene **3t** in 47% yield.
^{1}H NMR (500 MHz, $CDCl_3$) δ 5.39 (s, 1H), 5.34 (s, 1H), 5.24 (dd, *J* = 9.4, 5.8 Hz, 1H), 5.16 (dq, *J* = 9.3, 1.6 Hz, 1H), 3.68 (d, *J* = 6.0 Hz, 1H), 1.86 (s, 3H), 1.77 (d, *J* = 1.6 Hz, 3H), 1.66 – 1.50 (m, 6H), 1.39 (dq, *J* = 12.0, 3.2 Hz, 1H), 1.17 (s, 3H), 1.13 (s, 3H), 1.00 (s, 6H).
^{13}C NMR (126 MHz, $CDCl_3$) δ 197.0, 159.1, 140.8, 121.2, 97.9, 77.3, 77.0, 76.8, 71.0, 60.8, 60.7, 39.7, 39.6, 32.5, 32.0, 25.9, 20.7, 20.4, 18.6, 16.9.
HRMS $[C_{17}H_{29}NO_3+H]^+$ calculated, 296.2226; measured, 296.2216.

***tert*-butyl 4-(1-hydroxy-2-oxo-3-((2,2,6,6-tetramethylpiperidin-1-yl)oxy)but-3-en-1-yl)piperidine-1-carboxylate (4s).**

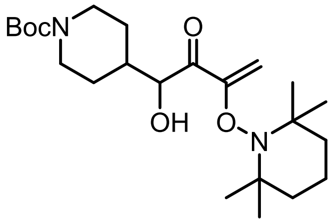

Prepared using **Method A** from allene **3u** in 54% yield.
^{1}H NMR (400 MHz, $CDCl_3$) δ 5.35 (d, *J* = 0.9 Hz, 1H), 5.28 (d, *J* = 0.9 Hz, 1H), 4.56 (s, 1H), 4.12 (s, 2H), 3.23 (s, 1H), 2.55 (d, *J* = 44.3 Hz, 2H), 2.14 (td, *J* = 11.2, 4.8 Hz, 1H), 1.62 (s, 3H), 1.55 – 1.46 (m, 5H), 1.38 (s, 10H), 1.36 – 1.27 (m, 2H), 1.15 (s, 3H), 1.11 (s, 3H), 1.00 (s, 3H), 0.93 (s, 3H).
^{13}C NMR (126 MHz, $CDCl_3$) δ 197.5, 159.2, 154.7, 97.9, 79.4, 77.3, 77.0, 76.9, 76.8, 61.0, 60.8, 39.8, 39.6, 39.3, 32.5, 32.5, 29.4, 28.4, 24.1, 21.0, 20.7, 16.8.
HRMS $[C_{23}H_{40}N_2O_5+H]^+$ calculated, 425.3010; measured, 425.3005.

1-((1*S*,5*R*)-6,6-dimethylbicyclo[3.1.1]hept-2-en-3-yl)-1-hydroxy-3-((2,2,6,6-tetramethylpiperidin-1-yl)oxy)but-3-en-2-one (4t).

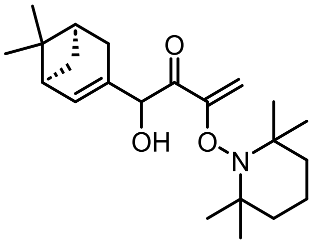

Prepared using **Method A** from allene **3v** in 53% yield.
^{1}H NMR (500 MHz, $CDCl_3$) δ 5.78 (tt, *J* = 2.9, 1.4 Hz, 1H), 5.40 (d, *J* = 0.8 Hz, 1H), 5.36 (d, *J* = 0.9 Hz, 1H), 5.13 (d, *J* = 6.9 Hz, 1H), 3.59 (d, *J* = 7.0 Hz, 1H), 2.33 – 2.28 (m, 3H), 2.07 (dddq, *J* = 5.8, 4.2, 2.9, 1.4 Hz, 1H), 1.97 (td, *J* = 5.6, 1.6 Hz, 1H), 1.54 (s, 7H), 1.42 – 1.35 (m, 2H), 1.26 (s, 4H), 1.21 (d, *J* = 3.9 Hz, 7H), 1.08 (d, *J* = 8.8 Hz, 1H), 1.02 (s, 3H), 0.99 (s, 3H), 0.86 (s, 3H).
^{13}C NMR (126 MHz, $CDCl_3$) δ 196.0, 159.5, 143.8, 124.9, 98.5, 77.3, 77.0, 76.8, 60.9, 60.8, 40.8, 40.6, 39.8, 39.7, 38.1, 32.5, 32.2, 31.6, 31.2, 26.0, 21.2, 21.0, 20.8, 16.9.
HRMS $[C_{22}H_{35}NO_3+H]^+$ calculated, 362.2695; measured, 362.2686.

4-hydroxy-2,5-bis((2,2,6,6-tetramethylpiperidin-1-yl)oxy)hexa-1,5-dien-3-one (4u).

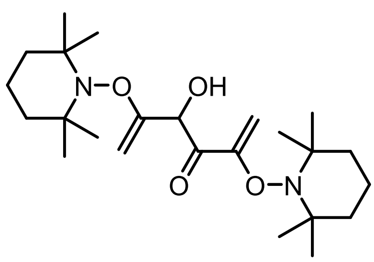

Prepared using **Method A** from allene **3w** in 73% yield.
^{1}H NMR (500 MHz, CDCl$_3$) δ 5.49 (d, *J* = 1.2 Hz, 1H), 5.46 (d, *J* = 1.1 Hz, 1H), 5.11 (d, *J* = 7.8 Hz, 1H), 4.91 (s, 1H), 4.35 (s, 1H), 3.92 (d, *J* = 8.1 Hz, 1H), 1.57 – 1.43 (m, 9H), 1.35 (ddq, *J* = 22.5, 12.0, 3.2 Hz, 2H), 1.20 (d, *J* = 1.5 Hz, 6H), 1.11 (d, *J* = 17.2 Hz, 6H), 1.00 (dd, *J* = 17.2, 12.0 Hz, 13H).
^{13}C NMR (126 MHz, CDCl$_3$) δ 193.7, 160.7, 159.1, 99.4, 89.7, 77.3, 77.0, 76.8, 74.8, 60.8, 60.8, 60.7, 60.5, 39.9, 39.8, 39.8, 39.7, 32.7, 32.6, 32.2, 32.2, 20.9, 20.7, 20.4, 20.3, 16.91, 16.9.
HRMS [$C_{24}H_{42}N_2O_4$+H]$^+$ calculated, 423.3223; measured, 423.3208.

(*E*)-2-((2,2,6,6-tetramethylpiperidin-1-yl)oxy)hex-2-enal (4v).

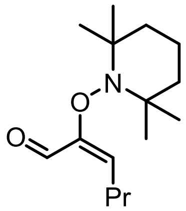

Prepared using **Method A** from allene **7a** in 55% yield.
^{1}H NMR (500 MHz, CDCl$_3$) δ 9.78 (s, 1H), 6.41 (t, *J* = 8.8 Hz, 1H), 2.44 (dt, *J* = 8.8, 7.2 Hz, 2H), 1.56 – 1.45 (m, 8H), 1.37 (dp, *J* = 12.7, 3.3 Hz, 1H), 1.20 (s, 6H), 0.98 (s, 6H), 0.94 (t, *J* = 7.4 Hz, 4H).
^{13}C NMR (126 MHz, CDCl$_3$) δ 184.6, 154.2, 124.2, 77.3, 77.0, 76.8, 60.5, 39.7, 32.3, 26.8, 23.7, 20.6, 17.0, 13.5.
HRMS [$C_{15}H_{27}NO_2$+H]$^+$ calculated, 254.2120; measured, 254.2110.

(*E*)-4-((tetrahydro-2H-pyran-2-yl)oxy)-2-((2,2,6,6-tetramethylpiperidin-1-yl)oxy)but-2-enal (4w).

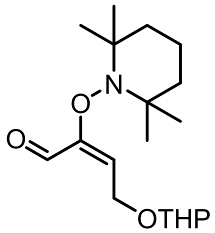

Prepared using **Method A** from allene **7b** in 46% yield.
^{1}H NMR (500 MHz, CDCl$_3$) δ 9.79 (s, 1H), 6.41 (t, *J* = 7.6 Hz, 1H), 4.63 (dd, *J* = 4.5, 2.9 Hz, 1H), 4.60 – 4.49 (m, 2H), 3.86 (ddd, *J* = 11.2, 7.5, 3.1 Hz, 1H), 3.49 (dd, *J* = 10.9, 5.2 Hz, 1H), 1.87 – 1.77 (m, 1H), 1.71 (ddt, *J* = 12.1, 8.4, 3.2 Hz, 1H), 1.65 – 1.46 (m, 11H), 1.38 (dq, *J* = 12.7, 3.4 Hz, 1H), 1.20 (d, *J* = 1.4 Hz, 6H), 1.01 (s, 6H).
^{13}C NMR (126 MHz, CDCl$_3$) δ 185.8, 155.3, 117.4, 97.6, 77.3, 77.0, 76.8, 62.6, 60.8, 60.8, 60.7, 39.7, 32.3, 32.3, 30.6, 25.7, 25.4, 20.6, 19.6, 16.9.
HRMS [$C_{18}H_{31}NO_4$+H]$^+$ calculated, 326.2331; measured, 326.2321.

V. Cyclic Voltammograms.

All cyclic voltammetry (CV) was carried out in a glass cell with Pine WaveNow potentiostat. A glassy carbon disk electrode (diameter is 3.0 mm, PCTFE shroud) was used as a working electrode. Platinum wire was used as a counter electrode. $AgNO_2$ electrode was used as a reference electrode. Electrolyte: 0.1 M $TBAPF_6$ in MeCN. The scan rate was 100 mV s^{-1}.

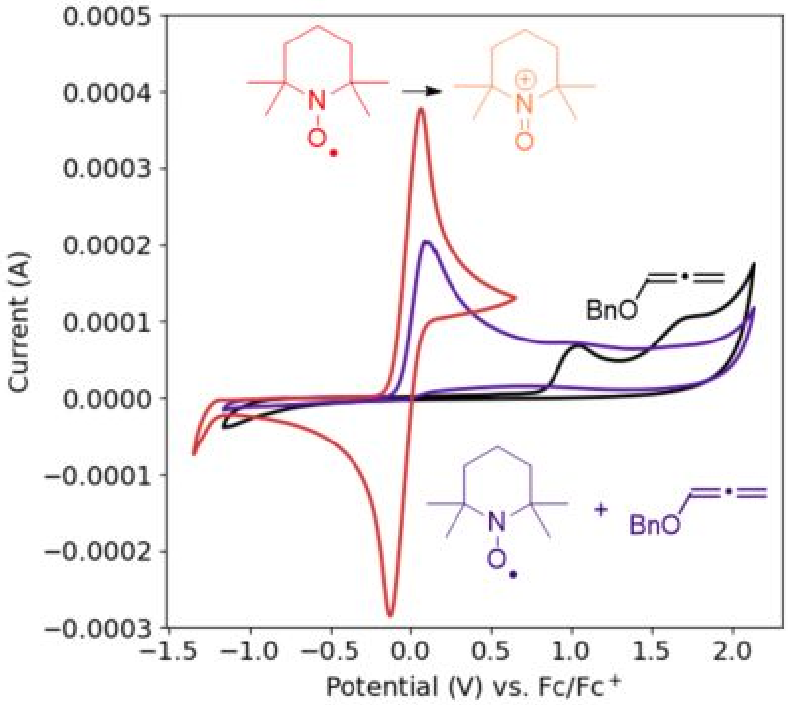

Figure S1. CV of allene **1**, TEMPO, and allene **1** + TEMPO (0.05 mmol of substrates).

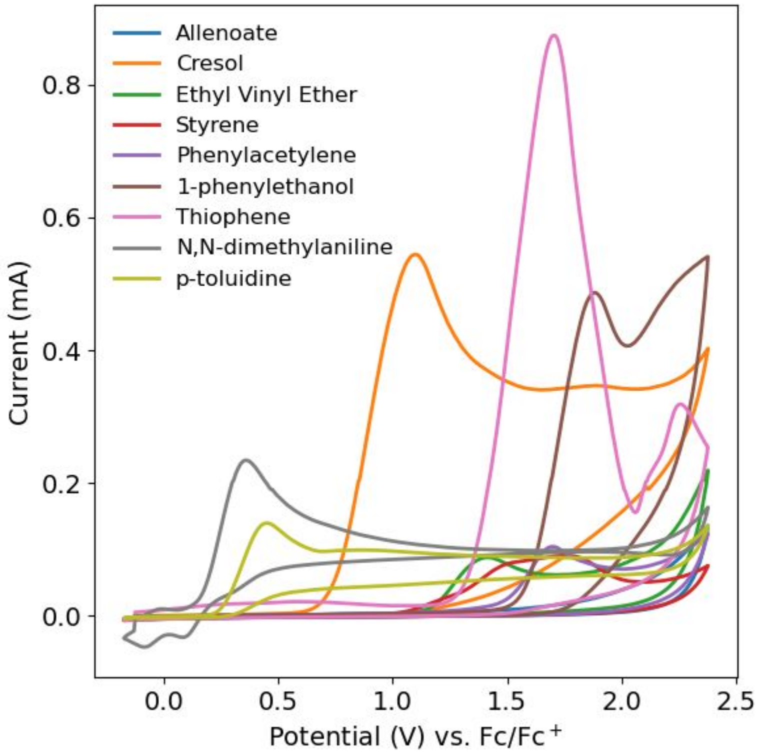

Figure S2. CV of additives (0.05 mmol of substrates).

VI. Proposed Mechanisms.

We propose either a radical mechanism building on the insights from our previous paper, or a polar mechanism based on established electrophilic nature of oxoammonium salts (Figure S3). In the radical pathway, allene **1** undergoes single electron transfer (SET) oxidation by TEMPO$^+$BF$_4^-$ to generate radical cation **INT1**, followed by rapid radical recombination with TEMPO to afford **INT2**. In the polar pathway, the lone pairs from the oxygen of alkoxyallene **1** promote nucleophilic attack at the oxygen center of TEMPO$^+$BF$_4^-$ to form **INT2**. This intermediate is resonance-stabilized with the lone pairs from the adjacent oxygen as an oxocarbenium ion. Water insertion followed by proton transfer results in the formation of **INT3**, where an elimination of benzyl alcohol reveals the TEMPO-acrolein **2**. Computational studies were also conducted to generate a reaction coordinate diagram for the proposed mechanisms (See **Figure S6-7** below).

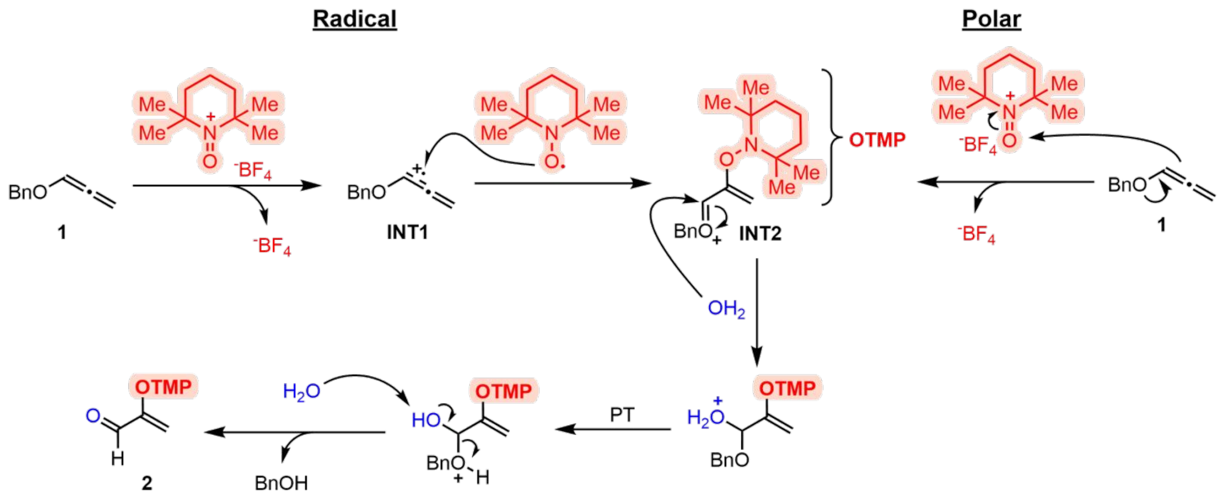

Figure S3. Two Proposed Mechanisms of TEMPO-mediated dioxygenation.

VII. Postfunctionalization reactions.

3-iodo-2-oxopropanal (8).

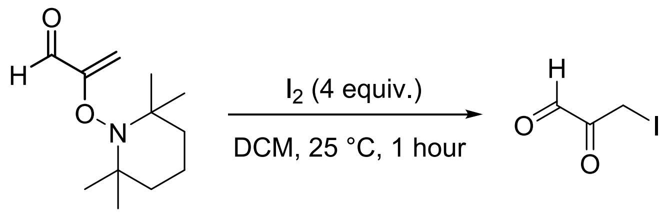

To a covered vial of **2** (0.1 mmol) in DCM (0.1 M) was added I$_2$ (4 equiv.) and stirred at 25 °C for one hour. The reaction mixture was concentrated via rotary evaporation, mesitylene was added as an internal NMR standard, and the yield of **8** was determined to be 90% yield. Although the crude product can be dried down alongside excess iodine, the product rapidly decomposes in solution.

^{1}H NMR (500 MHz, CDCl$_3$) δ 9.32 (s, 1H), 4.13 (s, 2H).

^{13}C NMR (126 MHz, CDCl$_3$) δ 189.7, 186.3, -3.5.

Crude mixture with TMP+ reported due to instability of product.

2-(iodomethyl)quinoxaline (9).

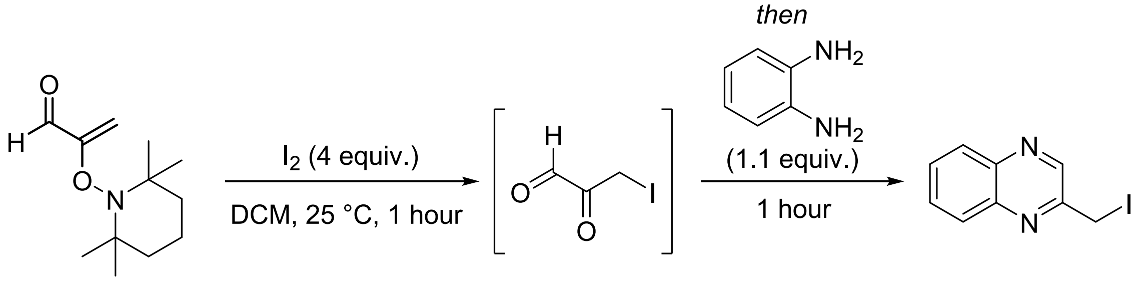

To a covered vial of **8** (0.1 mmol) prior to reaction work up was added 1,2-phenyldiamine (1.1 equiv.) and stirred at 25 °C for one hour. The reaction was quenched by addition of sat. $Na_2S_2O_3$ and washed with DCM (3x), dried over $MgSO_4$, and concentrated via rotary evaporation. The crude mixture was purified via column chromatography (9:1 Hex:EtOAc) to yield **9** in 83% yield.
Note: Product is unstable in polar solvent, decomposing by release of iodine to afford the free methyl.
^{1}H NMR (500 MHz, CDCl$_3$) δ 8.96 (s, 1H), 8.09 (dd, J = 7.1, 2.8 Hz, 1H), 8.04 (dd, J = 7.1, 2.8 Hz, 1H), 7.81 – 7.72 (m, 2H), 4.69 (s, 2H).
^{13}C NMR (126 MHz, CDCl$_3$) δ 153.6, 145.3, 141.6, 141.4, 130.5, 130.1, 129.2, 129.1, 2.3.
HRMS $[C_9H_7IN_2+H]^+$ calculated, 270.9732; measured, 270.9720.

2-(azidomethyl)quinoxaline (10).

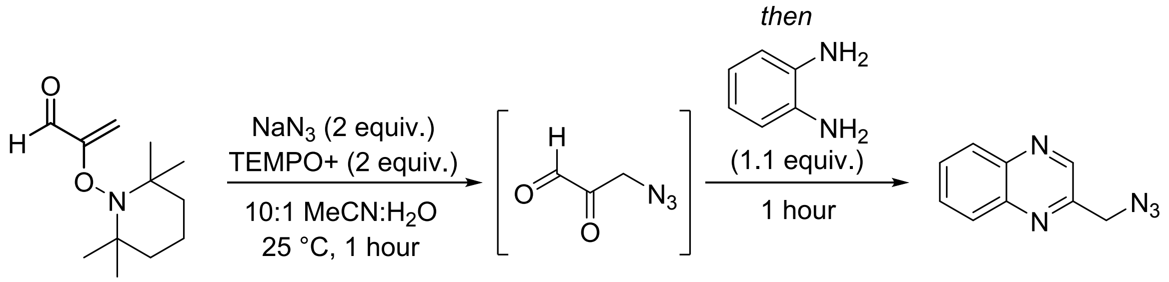

To a round-bottom flask with **2** (0.4 mmol) in 10:1 MeCN:H_2O (0.2 M) was added NaN_3 (2 equiv.), followed by $TEMPO^+BF_4^-$ (2 equiv.). The solution turned black the moment the oxidant was added and cleared up over time as the reaction progressed. After stirring at 25 °C for one hour, 1,2-phenyldiamine (1.1 equiv.) was added and stirred for an additional hour. The crude mixture was diluted with water, rinsed with EtOAc (3x), dried over $MgSO_4$, and concentrated via rotary evaporation. The crude mixture was purified via column chromatography (4:1 Hex:EtOAc) to yield **10** in 69% yield.
^{1}H NMR (500 MHz, CDCl$_3$) δ 8.91 (s, 1H), 8.18 – 8.06 (m, 2H), 7.85 – 7.75 (m, 2H), 4.73 (s, 2H).

^{13}C NMR (126 MHz, CDCl$_3$) δ 150.7, 144.1, 142.2, 141.8, 130.6, 130.2, 129.4, 129.3, 54.0.
HRMS $[C_9H_7N_5+H]^+$ calculated, 186.0780; measured, 186.0773.

methyl 1-(quinoxalin-2-ylmethyl)-1H-1,2,3-triazole-4-carboxylate (11).

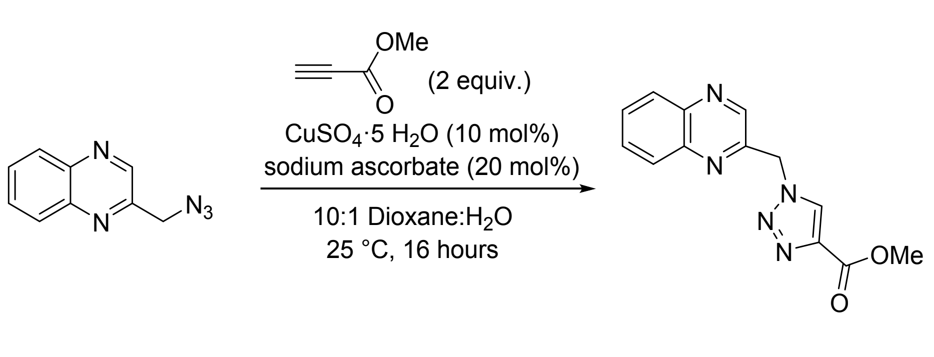

To a round-bottom flask with **10** (0.14 mmol) in 10:1 Dioxane:H_2O (0.35 M) was added methyl propiolate (2 equiv.), followed by sodium ascorbate (20 mol%) and $CuSO_4$ (10 mol%). The reaction mixture was stirred at 25 °C for 16 hours. The crude mixture was quenched with water, extracted with EtOAc (3x), dried over $MgSO_4$, and concentrated via rotary evaporation. The crude mixture was purified via column chromatography (9:1 DCM:MeOH) to yield **11** in 55% yield.
^{1}H NMR (500 MHz, $CDCl_3$) δ 8.91 (s, 1H), 8.37 (s, 1H), 8.14 (dd, *J* = 6.2, 3.6 Hz, 1H), 8.10 – 8.04 (m, 1H), 7.83 (dt, *J* = 6.5, 3.5 Hz, 2H), 5.95 (s, 2H), 3.95 (s, 3H).
^{13}C NMR (126 MHz, $CDCl_3$) δ 161.0, 148.1, 143.8, 142.5, 141.8, 140.6, 131.0, 130.9, 129.5, 129.3, 128.3, 53.9, 52.3.
HRMS $[C_{13}H_{11}N_5O_2+H]^+$ calculated, 270.0991; measured, 270.0983.

2-(1-((2,2,6,6-tetramethylpiperidin-1-yl)oxy)vinyl)-1H-benzo[d]imidazole (12).

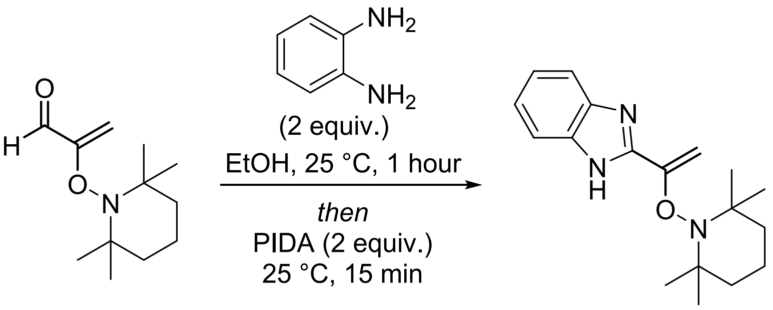

To a round-bottom flask with **2** (0.24 mmol) in EtOH (0.4 M) was added 1,2-phenyldiamine (2 equiv.) and stirred at 25 °C for 1 hour. Iodobenzene diacetate (2 equiv.) was then added to the mixture and stirred for 15 minutes before the crude mixture was quenched with water. The solution was then extracted with EtOAc (3x), dried over $MgSO_4$, and concentrated via rotary evaporation. The crude mixture was purified via column chromatography (7:3 Hex:EtOAc) to yield **12** in 51% yield.
^{1}H NMR (500 MHz, $CDCl_3$) δ 9.31 (s, 1H), 7.80 – 7.75 (m, 1H), 7.53 – 7.45 (m, 1H), 7.29 – 7.27 (2H, overlapped with residual $CHCl_3$), 5.63 (d, *J* = 1.1 Hz, 1H), 5.30 (d, *J* = 1.2 Hz, 1H), 1.71 – 1.57 (m, 5H), 1.48 – 1.41 (m, 1H), 1.29 (s, 6H), 1.14 (s, 6H).
^{13}C NMR (126 MHz, $CDCl_3$) δ 153.3, 147.5, 143.5. 132.8, 123.2, 122.6, 119.8, 110.8, 91.6, 60.8, 39.7, 32.5, 20.9, 17.0.
HRMS $[C_{18}H_{25}N_3O+H]^+$ calculated, 300.2076; measured, 300.2067.

1-(1H-benzo[d]imidazol-2-yl)-2-iodoethan-1-one (13).

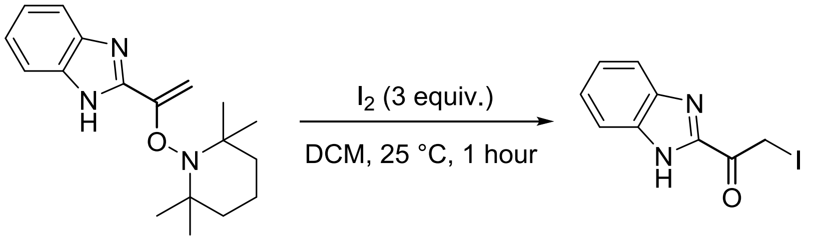

To a covered vial of **12** (0.17 mmol) in DCM (0.1 M) was added I_2 (3 equiv.) and stirred at 25 °C for one hour. The reaction was quenched by addition of sat. $Na_2S_2O_3$ and washed with DCM (3x), dried over $MgSO_4$, and concentrated via rotary evaporation to yield **13** in 99% yield.
Note: Product is unstable in polar solvent, decomposing by release of iodine to afford the free methyl.
^{1}H NMR (500 MHz, $CDCl_3$) δ 10.03 (s, 1H), 7.93 (d, *J* = 8.1 Hz, 1H), 7.56 (d, *J* = 8.1 Hz, 1H), 7.45 (t, *J* = 7.6 Hz, 1H), 7.40 (t, *J* = 7.6 Hz, 1H), 7.40 (t, *J* = 7.6 Hz, 1H), 4.71 (s, 2H).
^{13}C NMR (126 MHz, $CDCl_3$) δ 186.7, 144.7, 143.4, 133.9, 127.1, 124.2, 122.3, 112.1, 0.3.
HRMS $[C_9H_7IN_2O+H]^+$ calculated, 286.9682; measured, 286.9669.

4-phenyl-5-(1-((2,2,6,6-tetramethylpiperidin-1-yl)oxy)vinyl)oxazole (14).

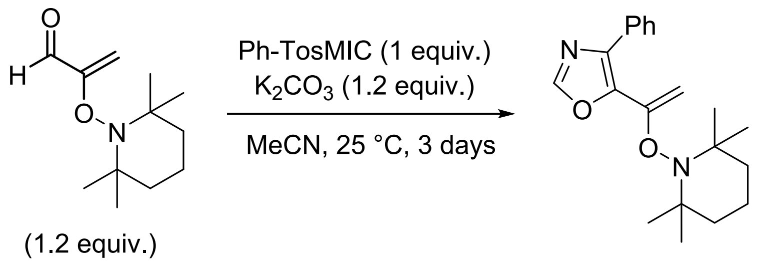

To a round-bottom flask with **2** (0.13 mmol, 1.2 equiv) in MeCN (0.1 M) was added potassium carbonate (1.2 equiv.), followed by Ph-TosMIC (0.1 mmol, 1.0 equiv). The reaction mixture was stirred at 25 °C for 3 days. The crude mixture was quenched with water, extracted with DCM (3x), dried over $MgSO_4$, and concentrated via rotary evaporation. The crude mixture was purified via column chromatography (4:1 Hex:EtOAc) to yield **14** in 55% yield.

^{1}H NMR (500 MHz, $CDCl_3$) δ 7.86 (s, 1H), 7.75 (d, *J* = 8.0 Hz, 2H), 7.37 (t, *J* = 7.5 Hz, 2H), 7.31 (t, *J* = 7.4 Hz, 1H), 5.24 (s, 1H), 4.77 (s, 1H), 1.63 – 1.47 (m, 5H), 1.40 – 1.32 (m, 1H), 1.12 (s, 6H), 1.00 (s, 6H).

^{13}C NMR (126 MHz, $CDCl_3$) δ 153.5, 149.4, 141.6, 136.1, 132.0, 128.6, 128.1, 128.0, 91.4, 60.6, 39.8, 32.3, 20.8, 17.0.

HRMS $[C_{20}H_{26}N_2O_2+H]^+$ calculated, 327.2073; measured, 327.2064.

1-(4-phenyloxazol-5-yl)-2-(pyridin-2-ylthio)ethan-1-one (15).

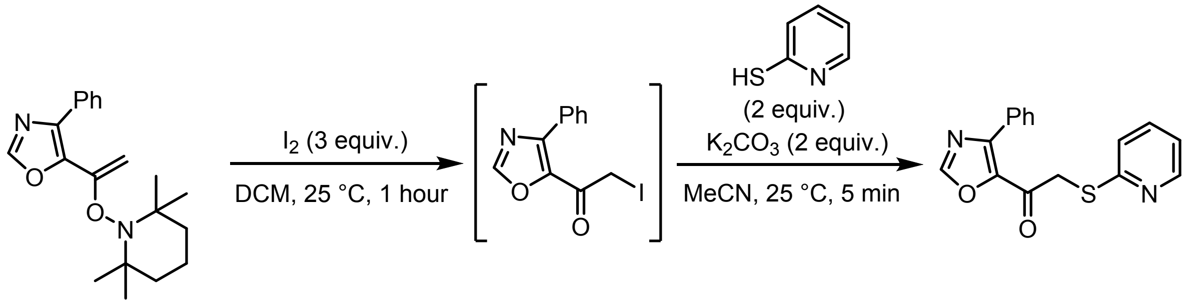

To a round-bottom flask with **14** (0.045 mmol) in DCM (0.1 M) was added I_2 (3 equiv.) and stirred at 25 °C for 1 hour. After stirring one hour, 1,2-phenyldiamine (1.1 equiv.) was added and stirred for an additional hour. The crude mixture was quenched with sat. $Na_2S_2O_3$, extracted with DCM (3x), dried over $MgSO_4$, and concentrated via rotary evaporation. The crude mixture was dissolved in MeCN (0.1 M) and added potassium carbonate (2 equiv.) followed by 2-mercaptopyridine (2 equiv.) and stirred at 25 °C for 5 minutes. The crude mixture was quenched with water, extracted with DCM (3x), dried over $MgSO_4$, and concentrated via rotary evaporation. The mixture was purified via column chromatography (9:1 DCM:Et_2O) to yield **15** in 45% yield.

^{1}H NMR (500 MHz, $CDCl_3$) δ 8.29 (d, *J* = 4.9 Hz, 1H), 8.24 – 8.20 (m, 2H), 8.02 (s, 1H), 7.48 (td, *J* = 7.7, 1.9 Hz, 1H), 7.45 – 7.40 (m, 3H), 7.29 – 7.25 (m, 1H), 6.98 – 6.94 (m, 1H), 4.60 (s, 2H).

^{13}C NMR (126 MHz, $CDCl_3$) δ 183.6, 156.6, 151.0, 149.3, 145.6, 143.5, 136.1, 130.3, 129.7, 129.2, 128.3, 122.1, 119.8, 38.2.

HRMS $[C_{16}H_{12}N_2O_2S +H]^+$ calculated, 297.0698; measured, 297.0688.

1-((1-(1-(4-bromobenzyl)-4-phenyl-1H-imidazol-5-yl)vinyl)oxy)-2,2,6,6-tetramethylpiperidine (16).

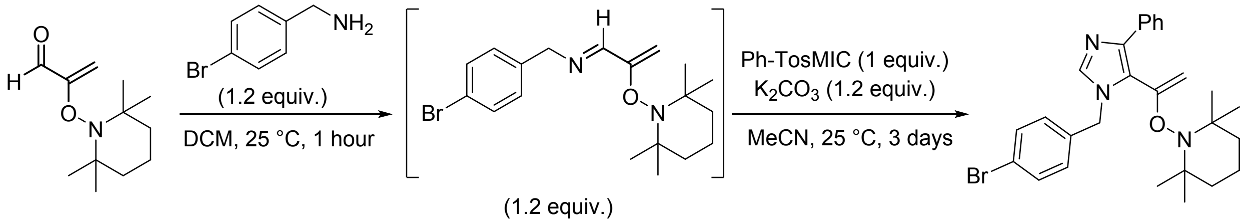

To a round-bottom flask with **2** (0.047 mmol) in DCM (0.2 M) was added 4-bromobenzylamine (1.5 equiv.) and stirred at 25 °C for 1 hour. The crude mixture was quenched with water, extracted with DCM (3x), dried over $MgSO_4$, and concentrated via rotary evaporation. The crude mixture was dissolved in MeCN (0.1 M) and added potassium carbonate (1 equiv.) followed by Ph-TosMIC (0.83 equiv.) and stirred at 25 °C for 3 days. The crude mixture was quenched with water, extracted with DCM (3x), dried over $MgSO_4$, and concentrated via rotary evaporation. The mixture was purified via column chromatography (4:1 Hex:EtOAc) to yield **16** in 68% yield.

^{1}H NMR (500 MHz, CDCl$_3$) δ 7.87 – 7.83 (m, 2H), 7.49 – 7.45 (m, 2H), 7.44 (s, 1H), 7.35 – 7.28 (m, 2H), 7.27 – 7.21 (m, 1H), 7.00 (d, J = 8.4 Hz, 2H), 5.37 (s, 1H), 5.25 (s, 2H), 4.46 (s, 1H), 1.56 – 1.45 (m, 5H), 1.39 – 1.28 (m, 1H), 1.04 (s, 6H), 0.94 (s, 6H).

^{13}C NMR (126 MHz, CDCl$_3$) δ 153.5, 140.8, 137.4, 136.0, 134.8, 132.0, 128.4, 128.1, 127.9, 127.0, 124.7, 121.8, 95.5, 60.4, 48.6, 40.1, 32.7, 20.5, 16.9.

HRMS $[C_{27}H_{32}BrN_3O+H]^+$ calculated, 494.1802; measured, 494.1799.

4-(1-(4-bromobenzyl)-4-phenyl-1H-imidazol-5-yl)thiazol-2-amine (17).

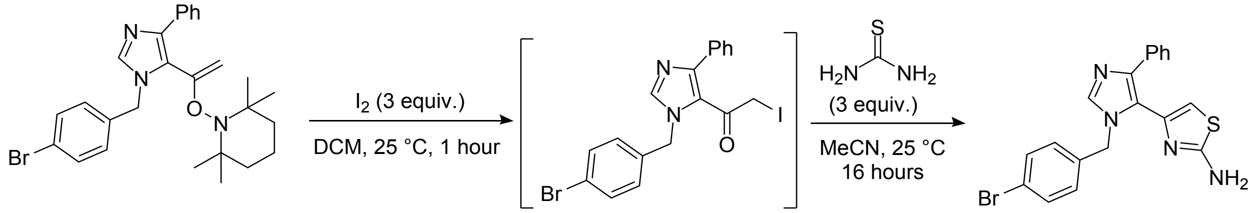

To a round-bottom flask with **16** (0.032 mmol) in DCM (0.1 M) was added I_2 (3 equiv.) and stirred at 25 °C for 1 hour. The crude mixture was quenched with sat. $Na_2S_2O_3$ and washed with DCM (3x), dried over $MgSO_4$, and concentrated via rotary evaporation. The crude mixture was dissolved in MeCN (0.1 M) and added thiourea (3 equiv.) and stirred at 25 °C for 16 hours. The crude mixture was quenched with water, extracted with DCM (3x), dried over $MgSO_4$, and concentrated via rotary evaporation. The mixture was purified via column chromatography (9:1 DCM:MeOH) to yield **17** in 73% yield.

^{1}H NMR (500 MHz, DMSO-d6) δ 7.94 (s, 1H), 7.62 (d, J = 7.3 Hz, 2H), 7.54 (d, J = 8.4 Hz, 2H), 7.32 (t, J = 7.7 Hz, 2H), 7.22 (t, J = 7.3 Hz, 1H), 7.15 (s, 2H), 7.09 (d, J = 8.4 Hz, 2H), 6.52 (s, 1H), 5.23 (s, 2H).

^{13}C NMR (126 MHz, DMSO-d6) δ 168.4, 139.5, 138.4, 137.7, 137.0, 134.8, 131.3, 129.4, 128.0, 126.2, 126.2, 123.3, 120.6, 108.2, 47.3.

HRMS $[C_{19}H_{15}BrN_4S +H]^+$ calculated, 411.0274; measured, 411.0268.

(*E*)-2-(4-((2,2,6,6-tetramethylpiperidin-1-yl)oxy)penta-2,4-dien-1-yl)isoindoline-1,3-dione (19).

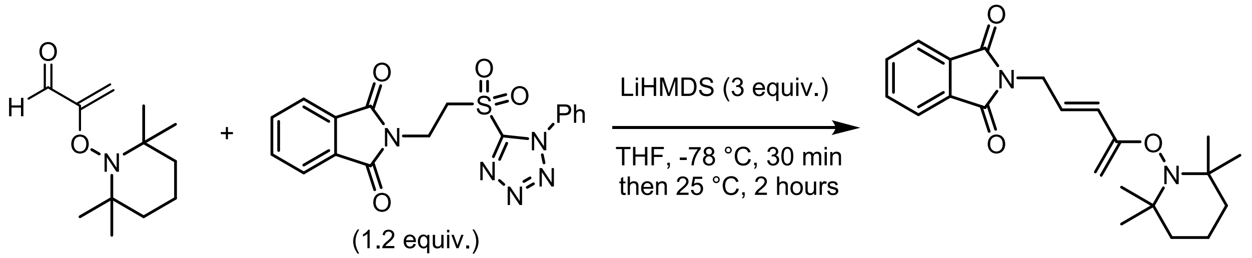

To a flame dried flask charged with a stir bar was added **2**, 2-(2-((1-phenyl-1H-tetrazol-5-yl)sulfonyl)ethyl)isoindoline-1,3-dione **18** (1.2 equiv.), and THF (0.2 M). The reaction vessel was sealed under an atmosphere of nitrogen and cooled in a dry-ice bath to -78 ºC. To the cooled, stirring solution was added a solution of LiHMDS in THF (0.1 M) dropwise over 15 minutes. The reaction was kept at -78 ºC for 1 hour, then allowed to warm to 25 °C over 3 hours and quenched by dropwise addition of cold water. The mixture changed from a homogenous red to an opaque orange-yellow and then further diluted with water and extracted with DCM (3x). The combined organic layer was dried over $MgSO_4$ and concentrated via rotary evaporation. The crude residue was purified by column chromatography (9:1 Hex:EtOAc + 1% triethylamine) to yield **19** 79% yield as a mixture of isomers (5:1 E:Z). It is possible to separate the isomers by column, however deprotection of the phthalimide group in HCl isomerizes any Z products to the desired E product.[6]

^{1}H NMR (500 MHz, CDCl$_3$) δ 7.87 (dd, *J* = 5.4, 3.0 Hz, 2H), 7.72 (dd, *J* = 5.5, 3.0 Hz, 2H), 6.05 (dt, *J* = 15.6, 5.9 Hz, 1H), 5.97 (d, *J* = 15.4 Hz, 1H), 4.87 (s, 1H), 4.35 (d, *J* = 5.9 Hz, 2H), 4.13 (s, 1H), 1.66 – 1.46 (m, 5H), 1.35 (dp, *J* = 12.2, 3.0 Hz, 1H), 1.15 (s, 6H), 1.00 (s, 6H).

^{13}C NMR (126 MHz, CDCl$_3$) δ 167.9, 159.5, 133.9, 132.2, 128.6, 123.3, 121.5, 91.7, 60.4, 39.7, 39.3, 32.3, 20.7, 17.0.

HRMS $[C_{22}H_{28}N_2O_3+H]^+$ calculated, 369.2173; measured, 369.2167.

(*E*)-2-(3-(imidazo[1,2-a]pyrimidin-2-yl)allyl)isoindoline-1,3-dione (20).

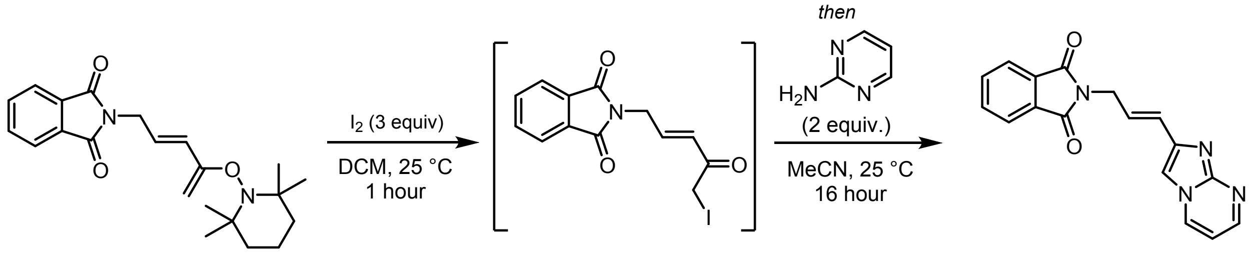

To a flask charged with **19** (1 equiv.) in DCM (0.1 M) was added I_2 (3 equiv.) and the reaction was stirred at 25 °C for 1 hour. The dark mixture was diluted with DCM and quenched with a solution of saturated $Na_2S_2O_3$. The combined organic layer was washed with brine and dried over $MgSO_4$ and concentrated via rotary evaporation. The dried residue was dissolved in acetonitrile (0.1 M), added 2-aminopyrimidine (2 equiv.), and stirred at 25 °C for 24 hours. Saturated aqueous $NaHCO_3$ was then added dropwise until the solution begins to precipitate, then stirred at 25 °C for a further 30 minutes. Then the reaction mixture is diluted with more saturated aqueous $NaHCO_3$ and washed with EtOAc (2x). The organic layer was then extracted with equal portions of saturated aqueous $Na_2S_2O_3$ (2x) and brine, then dried over $MgSO_4$, filtered, and concentrated via rotary evaporation. The crude residue was purified by column chromatography (95:5 DCM:MeOH), to afford **20** in 65% yield.

^{1}H NMR (500 MHz, DMSO-d6) δ 8.88 (dd, *J* = 6.7, 2.1 Hz, 1H), 8.47 (dd, *J* = 4.2, 2.0 Hz, 1H), 7.91 (dd, *J* = 5.4, 3.1 Hz, 2H), 7.89 – 7.84 (m, 3H), 6.98 (dd, *J* = 6.7, 4.1 Hz, 1H), 6.60 (s, 1H), 4.40 (d, *J* = 3.5 Hz, 2H), 3.31 (s, 1H).

^{13}C NMR (126 MHz, DMSO-d6) δ 167.6, 150.5, 147.8, 143.2, 135.0, 134.5, 131.7, 126.3, 123.6, 123.1, 109.5, 108.5.

HRMS $[C_{17}H_{12}N_4O_2+H]^+$ calculated, 305.1033; measured, 305.1026.

2-(2-aminothiazol-4-yl)-2-hydroxycyclopentan-1-one (21).

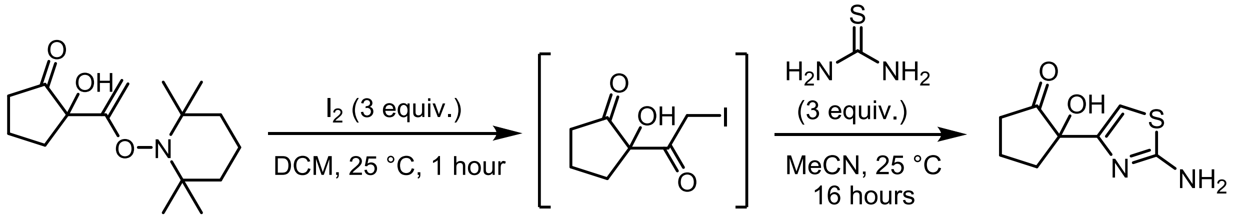

To a round-bottom flask with **5a** (0.21 mmol) in DCM (0.1 M) was added I_2 (3 equiv.) and stirred at 25 °C for 1 hour. After stirring one hour, 1,2-phenyldiamine (1.1 equiv.) was added and stirred for an additional hour. The crude mixture was quenched with sat. $Na_2S_2O_3$, extracted with DCM (3x), dried over $MgSO_4$, and concentrated via rotary evaporation. The crude mixture was dissolved in MeCN (0.1 M) and added thiourea (3 equiv.) and stirred at 25 °C for 16 hours. The crude mixture was quenched with water, extracted with DCM (3x), dried over $MgSO_4$, and concentrated via rotary evaporation. The mixture was purified via column chromatography (9:1 DCM:MeOH) to yield **21** in 31% yield.
^{1}H NMR (500 MHz, CD_3CN) δ 6.45 (s, 1H), 5.53 (bs, 2H), 3.77 (bs, 1H), 2.44 (dt, *J* = 12.7, 6.3 Hz, 1H), 2.34 (td, *J* = 7.5, 2.1 Hz, 2H), 2.05 – 1.95 (m, 3H).
^{13}C NMR (126 MHz, CD_3CN) δ 217.2, 169.3, 153.6, 104.5, 79.8, 37.7, 36.3, 18.4.
HRMS $[C_8H_{10}N_2O_2S +H]^+$ calculated, 199.0541; measured, 199.0532.

***tert*-butyl 4-hydroxy-4-(3-(iodomethyl)quinoxalin-2-yl)piperidine-1-carboxylate (22).**

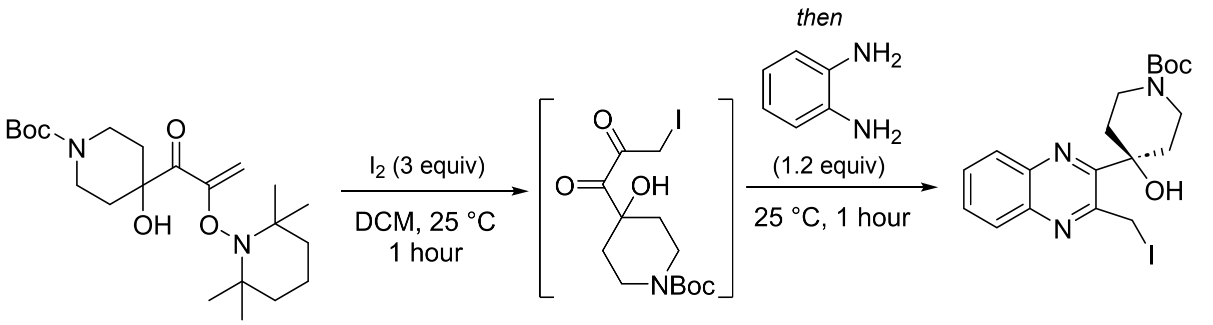

To a round-bottom flask with **4e** (0.11 mmol) in DCM (0.1 M) was added I_2 (3 equiv.) and stirred at 25 °C for 1 hour. After stirring one hour, 1,2-phenyldiamine (1.1 equiv.) was added and stirred for an additional hour. The crude mixture was quenched with sat. $Na_2S_2O_3$, extracted with DCM (3x), dried over $MgSO_4$, and concentrated via rotary evaporation. The mixture was purified via column chromatography (4:1 Hex:EtOAc) to yield **22** in 47% yield.
^{1}H NMR (500 MHz, $CDCl_3$) δ 8.07 – 7.99 (m, 2H), 7.77 (dt, *J* = 6.4, 3.4 Hz, 2H), 5.07 (s, 2H), 4.22 – 3.99 (m, 2H), 3.81 (s, 1H), 3.39 (bs, 2H), 2.44 (td, *J* = 13.1, 4.9 Hz, 2H), 1.85 – 1.79 (m, 2H), 1.52 (s, 9H).
^{13}C NMR (126 MHz, $CDCl_3$) δ 156.2, 155.0, 152.2, 141.3, 139.5, 130.5, 130.4, 128.7, 128.5, 79.7, 73.7, 36.4, 28.5, 6.5, (one signal overlapped).
HRMS $[C_{19}H_{24}IN_3O_3+H]^+$ calculated, 470.0941; measured, 470.0931.

***tert*-butyl 3H-spiro[furo[3,4-b]quinoxaline-1,4'-piperidine]-1'-carboxylate (23).**

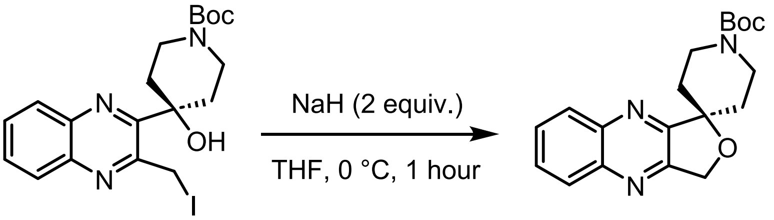

To a round-bottom flask with **22** (0.043 mmol) in THF (0.05 M) was added NaH (2 equiv.) and stirred at 0 °C for 1 hour. The crude mixture was quenched with water, extracted with DCM (3x), dried over $MgSO_4$, and concentrated via rotary evaporation. The mixture was purified via column chromatography (4:1 Hex:EtOAc) to yield **23** in 93% yield.
^{1}H NMR (500 MHz, $CDCl_3$) δ δ 8.13 – 8.04 (m, 2H), 7.77 (dt, *J* = 6.4, 3.5 Hz, 2H), 5.18 (s, 2H), 4.14 (s, 3H), 3.35 (s, 2H), 2.11 (ddd, *J* = 13.6, 11.7, 4.8 Hz, 2H), 1.83 (d, *J* = 13.5 Hz, 2H), 1.50 (s, 8H).

^{13}C NMR (126 MHz, CDCl$_3$) δ 159.1, 155.8, 154.7, 142.3, 142.1, 129.8, 129.7, 129.5, 129.1, 81.9, 79.6, 68.1, 29.7, 28.5.
HRMS [$C_{19}H_{23}N_3O_3$+H]$^+$ calculated, 342.1818; measured, 342.1808.

7'-bromo-3'H-spiro[cyclohexane-1,1'-furo[3,4-b]quinoxaline] (25).

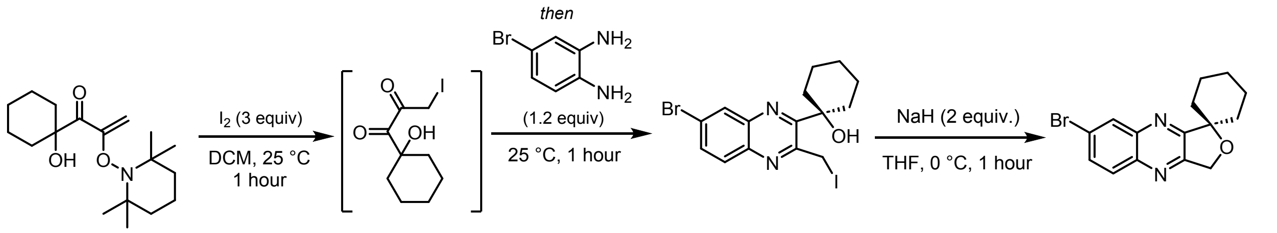

To a round-bottom flask with **4b** (0.27 mmol) in DCM (0.1 M) was added I_2 (3 equiv.) and stirred at 25 °C for 1 hour. After stirring one hour, 4-bromo-1,2-phenyldiamine (1.1 equiv.) was added and stirred for an additional hour. The crude mixture was quenched with sat. $Na_2S_2O_3$, extracted with DCM (3x), dried over $MgSO_4$, and concentrated via rotary evaporation. The mixture was purified via column chromatography (4:1 Hex:EtOAc) to yield **24**. The purified substrate was added to a round-bottom flask and dissolved in THF (0.05 M) and cooled to 0 °C. NaH (2 equiv.) was added to the solution and allowed to stir at this temperature for 1 hour. The crude mixture was quenched with water, extracted with DCM (3x), dried over $MgSO_4$, and concentrated via rotary evaporation. The mixture was purified via column chromatography (4:1 Hex:EtOAc) to yield **25** in 67% yield (1:1 r.r.).
^{1}H NMR (500 MHz, CDCl$_3$) δ 8.23 (d, J = 2.1 Hz, 1H), 7.96 (d, J = 8.8 Hz, 1H), 7.80 (dd, J = 8.8, 2.2 Hz, 1H), 5.13 (s, 2H), 1.95 – 1.71 (m, 9H), 1.53 – 1.41 (m, 1H).
^{13}C NMR (126 MHz, CDCl$_3$) δ 161.1, 157.3, 142.8, 140.9, 132.9, 131.4, 130.6, 123.2, 83.8, 67.8, 34.8, 25.1, 21.7.
HRMS [$C_{15}H_{15}BrN_2O$ +H]$^+$ calculated, 319.0441; measured, 319.0435.

(*E*)-3-(imidazo[1,2-a]pyrimidin-2-yl)prop-2-en-1-aminium chloride (26).

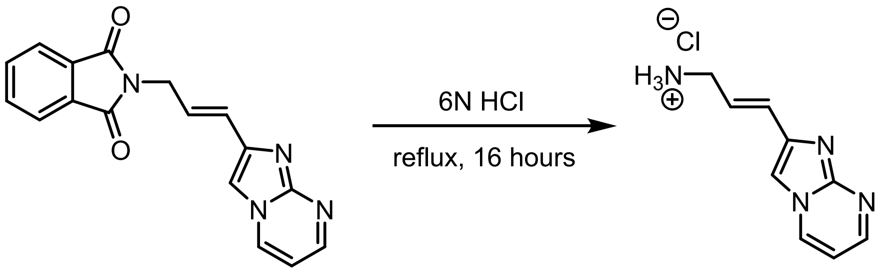

To a flask charged with stir bar was added **20** (1 equiv.) and a 6N solution of HCl (0.2 M). A reflux condenser was attached to the reaction vessel, and the reaction was heated to 100 ºC in an oil bath for 24 hours. The crude mixture was concentrated under nitrogen flow and the product was recrystallized from acetone to afford **26** in 96% yield as the 2x HCl salt.
^{1}H NMR (500 MHz, DMSO-d6) δ 9.21 (dd, J = 7.1, 1.9 Hz, 1H), 8.88 (dd, J = 4.4, 1.9 Hz, 1H), 8.43 (s, 3H), 8.29 (s, 1H), 7.47 (dd, J = 6.8, 4.3 Hz, 1H), 6.89 (dt, J = 16.1, 1.4 Hz, 1H), 6.77 (dt, J = 16.1, 6.3 Hz, 1H), 3.71 (qt, J = 6.1, 3.0 Hz, 2H).
HRMS [$C_9H_{11}N_4$]$^+$ calculated, 175.0978; measured, 175.0976.

HSQC used to confirm carbon peaks due to low resolution ^{13}C NMR.

(*E*)-N-(3-(imidazo[1,2-a]pyrimidin-2-yl)allyl)-1H-pyrrole-2-carboxamide (27).

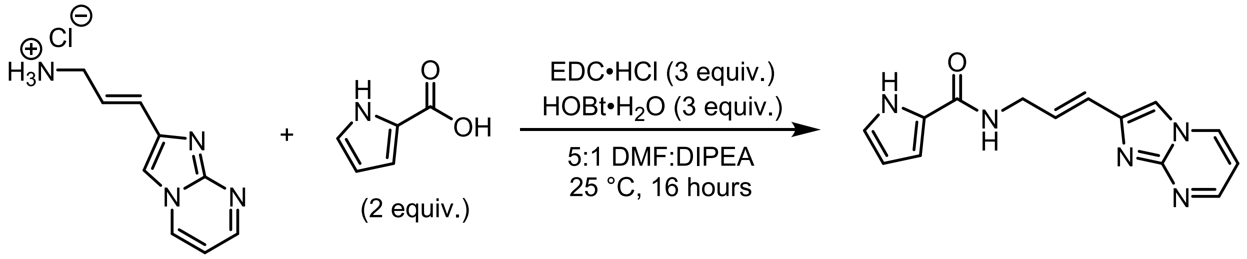

To a flask charged with a stir bar was added **26** (1 equiv.), pyrrole-2-carboxylic acid (2 equiv.), EDC-HCl (3 equiv.), HOBt-hydrate (3 equivalents), and DMF, then DIPEA in a 5:1 ratio (0.1 M). The reaction was sealed and stirred at 25 °C for 16 hours, then partitioned into equal volumes of ethyl acetate and saturated aqueous NH_4Cl and washed with NH_4Cl (3x) and with brine. The organic layer is dried over $MgSO_4$, filtered, and concentrated via rotary evaporation. The crude residue was purified by column chromatography (9:1 DCM:MeOH + 1% triethylamine) and recrystallized from acetone to afford **27** in 66% yield.

^{1}H NMR (500 MHz, DMSO-d6) δ 11.45 (s, 1H), 8.88 (dd, J = 6.7, 2.1 Hz, 1H), 8.48 (dd, J = 4.2, 2.1 Hz, 1H), 8.29 (t, J = 5.7 Hz, 1H), 7.89 (s, 1H), 6.99 (dd, J = 6.7, 4.1 Hz, 1H), 6.86 (dq, J = 10.0, 2.2 Hz, 2H), 6.67 – 6.53 (m, 2H), 6.10 (q, J = 2.8 Hz, 1H), 4.07 (t, J = 5.3 Hz, 2H).

^{13}C NMR (126 MHz, DMSO-d6) δ 161.0, 150.8, 148.3, 144.4, 135.3, 130.7, 126.7, 122.6, 121.8, 110.4, 109.4, 109.0, 108.9.

HRMS $[C_{14}H_{13}N_5O+H]^+$ calculated, 268.1193; measured, 268.1192.

(*E*)-N-(3-(2-amino-1H-imidazol-4-yl)allyl)-1H-pyrrole-2-carboxamide (28).

To a flask charged with stir bar was added **27** and neat hydrazine hydrate (0.2 M) and stirred at 25 °C for 1 hour. The reaction mixture was concentrated by rotary evaporation and further dried by azeotroping the solution with toluene (2x). The crude residue was purified by column chromatography (9:1 DCM:MeOH + 1% triethylamine) to afford **28** in 67% yield.

^{1}H NMR (500 MHz, MeOD) δ 6.92 (dd, J = 2.6, 1.4 Hz, 1H), 6.81 (dd, J = 3.7, 1.4 Hz, 1H), 6.48 (s, 1H), 6.31 (dt, J = 15.9, 1.6 Hz, 1H), 6.18 (dd, J = 3.8, 2.6 Hz, 1H), 5.92 (dt, J = 15.8, 6.1 Hz, 1H), 4.04 (dd, J = 6.1, 1.5 Hz, 2H), 3.33 (p, J = 1.6 Hz, 1H).

^{13}C NMR (126 MHz, MeOD) δ 162.2, 150.5, 129.8, 125.5, 121.4, 120.9, 120.8, 116.0, 110.3, 108.8.

HRMS $[C_{11}H_{13}N_5O+H]^+$ calculated, 232.1193; measured, 232.1193.

VIII. Computational details and Cartesian coordinates

The structures of **INT1**, **2**, and **12** were prepared and subjected to conformational sampling using both manual searches and the open-source *CREST* tool (part of the *x*TB software package). This tool employs the semiempirical extended tight-binding method (GFN2-*x*TB), which provides a good balance for exploring large regions of conformational space. However, its accuracy in computing thermochemical properties is limited, necessitating higher-level quantum chemical calculations.[7] The conformer ensembles obtained from *CREST* were filtered by excluding duplicates, based on an RMSD criterion applied to atomic coordinates within a 1.0 Å spatial cutoff. The selected representative geometries were then optimized at a lower level of theory using Gaussian 16 with the B3LYP functional and the 6-311++G(d,p) basis set for all atoms, except iodine, for which the LanL2DZ pseudopotential was employed to account for core electrons.[8] Dispersion corrections were included via Grimme's D3(BJ) scheme.[9]

All calculations incorporated solvation effects through the PCM model. For solvent mixtures, we adopted a parameter set of seven descriptors to better mimic experimental conditions.[10] Dichloromethane (DCM) solvation was modeled directly using Gaussian 16 parameters. For THF:H_2O (1:1) and MeCN:H_2O (10:1) mixtures, a linear combination of the individual solvent parameters was applied.[11] These descriptors include:

- static dielectric constant at 25 °C (*Eps*);
- optical dielectric constant (Eps_{inf});
- hydrogen-bond acidity and basicity (*Abraham's A* and *B* parameters);
- surface tension at the interface;
- fraction of aromatic carbons (carbon aromaticity);
- fraction of halogens (electronegative halogenicity).

Tables **S14** and **S15** report the parameters used for the pure solvents and the corresponding mixture values.

Table S14. List of seven parameters to simulate the mixture 1:1 of THF:H_2O.

Parameter	*H_2O*	*THF*	*H_2O:THF (1:1)*
**Eps**	78.355	7.4257	42.89
**EpsInf**	1.777849	1.974025	1.875937
**HBondAcidity**	0.0	0.00	0.00
**HBondBasicity**	0.0	0.48	0.24
**SurfaceTensionAtInterface**	0.0	26.4	13.20
**CarbonAromaticity**	0.0	0.0	0.0
**ElectronegativeHalogenicity**	0.0	0.0	0.0

Table S15. List of seven parameters to simulate the mixture 10:1 of MeCN:H_2O.

Parameter	*MeCN*	*H_2O*	*MeCN:H_2O (10:1)*
**Eps**	35.688	78.355	39.528
**EpsInf**	1.806874	1.777849	1.804262
**HBondAcidity**	0.07	0.0	0.06
**HBondBasicity**	0.32	0.0	0.29
**SurfaceTensionAtInterface**	41.25	0.0	37.54
**CarbonAromaticity**	0.0	0.0	0.0
**ElectronegativeHalogenicity**	0.0	0.0	0.0

When radical species were involved, unrestricted open-shell wave functions were employed. The final reported energies were thermally corrected to Gibbs free energies using electronic energies refined at a higher level of theory through single-point (SP) calculations. Specifically, optimized structures were re-evaluated with the ωB97X-D functional, which incorporates Grimme's D2 dispersion correction.[12] The def2-TZVP basis set was used for all atoms, while def2-TZVPD was applied for iodine, with corresponding pseudopotentials for heavy atoms.[13]

Solvent effects, including solvent mixtures, were modeled using the PCM approach with the parameter set described above.[10] Frequency calculations were carried out for all optimized structures to confirm whether they correspond to true minima or transition states (TS). Additionally, intrinsic reaction coordinate (IRC) calculations were performed to verify the correct connection of each TS to the associated reactants, intermediates, or products.[14]

Thermochemical properties were evaluated at temperatures of 273.15 K or 298.15 K, in accordance with experimental conditions, with the pressure fixed at 1.0 atm.

We started our investigation from the computational analysis of the radical azide α-addition to compound **2** (Figure S5). The reaction proceeds through a multi-step process, where firstly the formation of the new C-N_3 bond is achieved and the subsequent loss of ·TMP completes the reaction. The first step occurs with an activation energy of 12.9 kcal/mol (**TS1$_{azide}$**), while the second step (**TS2$_{azide}$**) occurs with an activation barrier of 13.1 kcal/ml – slightly more energy demanding than the previous one.

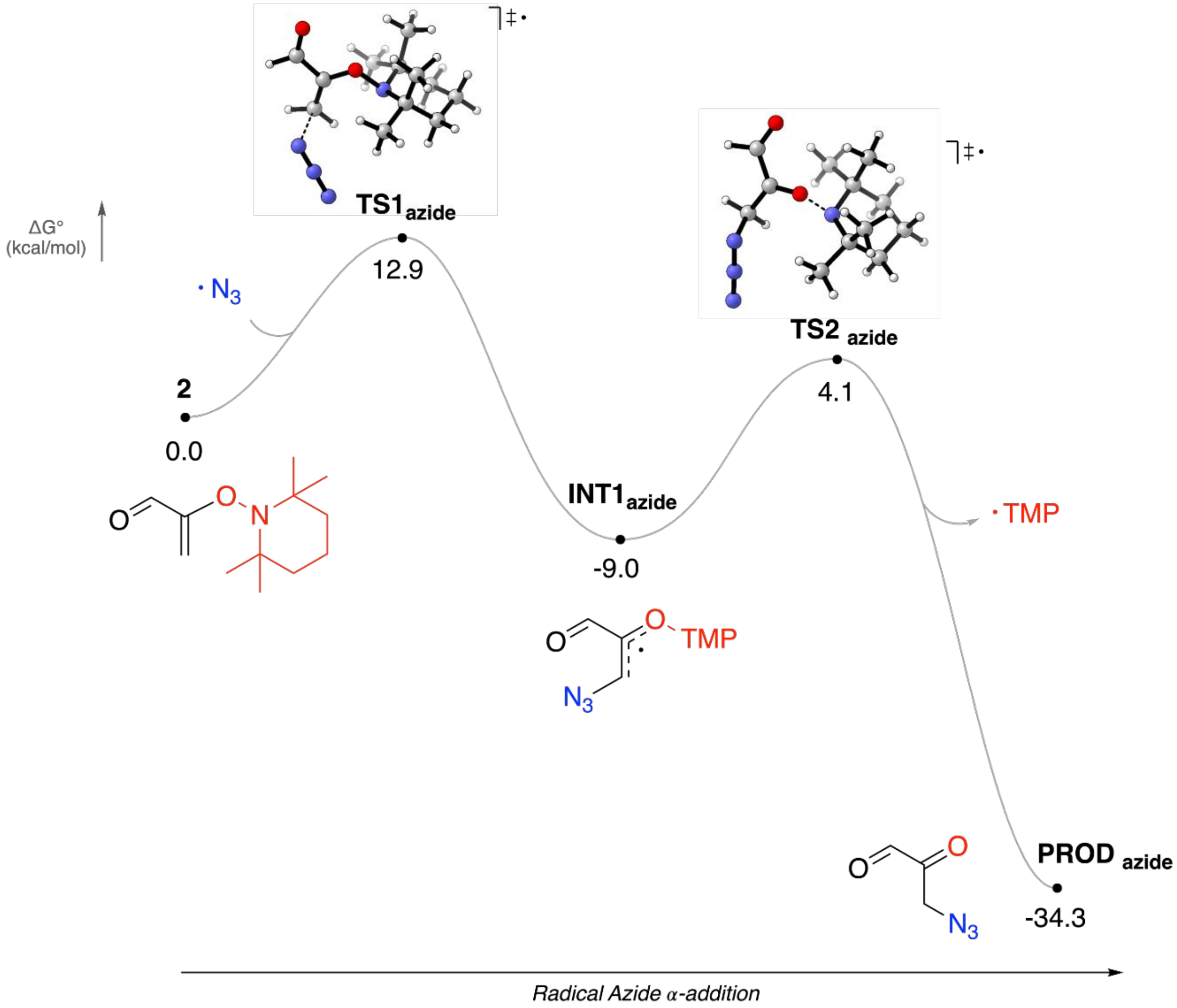

Figure S4. Full Gibbs free energy profile for radical azide α-addition to compound **2** at 25°C. [(PCM-H_2O:MeCN)-(U)ωB97XD/def2tzvp//(PCM-H_2O:MeCN)-GD3BJ-(U)B3LYP/6-311++g(d,p)]

To compare the radical pathway with a possible ionic pathway, we decided to investigate the iodine α-addition of compound **9**. To simulate the super-stoichiometric amount of I_2, a scenario which shows two molecules of I_2 closely interacting with each other (Figure S6). This system is particularly important in the formation of the stable I_3^- upon the delivery of I^+. The investigation started similarly to the one adopted in the N_3 case, but there were large differences. The process remains multi-step, but the first addition of I^+ is almost barrierless. Indeed, we never observed a stable saddle point that can be optimized to a transition state structure: as soon as **9** starts interacting with I_2, a spontaneous heterolytic cleavage of the I-I bond occurs, leading to the formation of **INT1$_{iodine}$** and the concomitant release of I_3^- anion. This step was also investigated with the bare I^+ cation and with one simple I_2 molecule but was unsuccessful in locating the transition state. However, the second step leading to product **10** has a low energy barrier (7.0 kcal/mol) and upon overcoming **TS1$_{iodine}$**, leads to the release

of TMP^+. This step is of particular interest since concomitantly with the release of the TMP^+ cation, we also observed its rearrangement to the ring-contracted iminium ion depicted in Figure S6. This structure is confirmed in the recently published papers from the Maulide and Erker groups.[15]

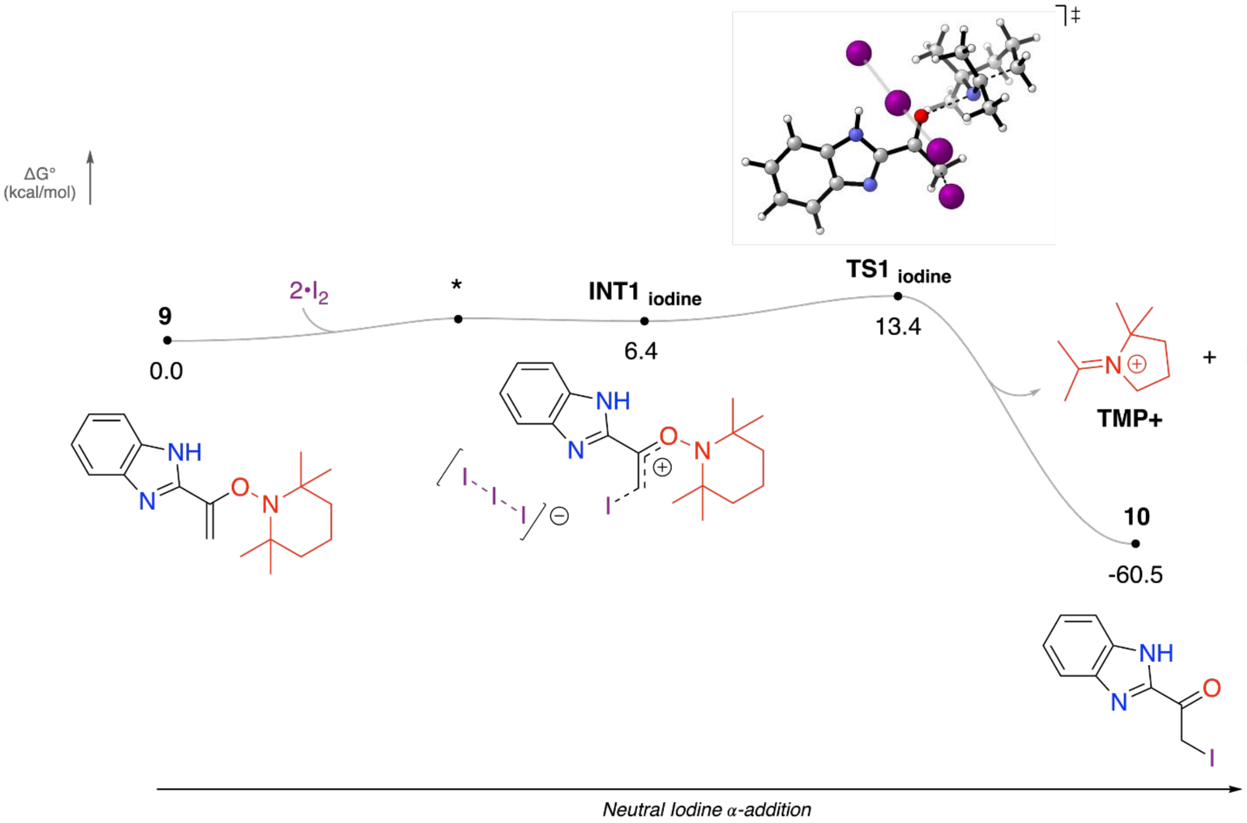

Figure S5: Full Gibbs free energy profile for ionic iodine α-addition to compound **12** at 25°C. [(PCM-CH_2Cl_2)-ωB97XD/def2tzvp&def2tzvpd//(PCM-CH_2Cl_2)-GD3BJ-B3LYP/6-311++g(d,p)&LanL2DZ]

We can thus conclude from the above results that the preferential reactivity of TMP-enols is the ionic pathway, being an almost barrierless process, compared with the higher activation barriers observed in the radical investigation with N_3 radical. Moreover, it seems that the TMP-enol system act as a "masked" enolate which far prefers the Coulombic annihilation of charge instead of the radical homolytic dissociation pathway.

The investigation then moved to the reaction mechanism of the dioxygenation (Figure S7). We started with the computational study for the formation of **INT2** as the initial radical-rebound for TEMPO and **INT1**.

To prove the spin state nature of **TS1**, Yamaguchi's analysis for the open-shell singlet species of the obtained structure was performed.

Yamaguchi's correction formula is reported below, as obtained from the literature.[16]

$$f_{sc} = \frac{\langle S^2 \rangle_{OSS}}{\langle S^2 \rangle_{triplet\ in\ OOS_{geometry}} - \langle S^2 \rangle_{OSS}}$$

$$E_{correction} = f_{sc} \cdot \left(E_{OSS} - E_{triplet\ in\ OSS_{geometry}} \right)$$

Energies reported in Table S3 are at [(PCM-THF:H2O)-ωB97XD/def2tzvp//(PCM-THF:H2O)-GD3BJ-B3LYP/6-311++g(d,p) level of theory and stability of wave function was tested for every system.

Table S16. Yamaguchi's correction for the open-shell singlet spin state of **TS2.**
[(PCM-THF:H_2O)-ωB97XD/def2tzvp//(PCM-THF:H_2O)-GD3BJ-B3LYP/6-311++g(d,p)]

Specie	E (a.u.)	$\langle S^2\rangle_{before}$ annihilation	$\langle S^2\rangle_{after}$ annihilation	f_{sc}	OSS$_{correction}$ (a.u.)	OSS$_{correction}$ (kcal/mol)
			**TS2**			
OSS	-945.803236	0.3659	0.0056			
Triplet wave function in OSS Geometry	-945.774911	2.0083	2.0000	0.223	-0.006310	-3.96

A non-negligible spin contamination deriving from the low-lying triplet spin state was observed and for that reason, the relative energy of **TS2** depicted in Figure S7 have been adjusted accordingly.

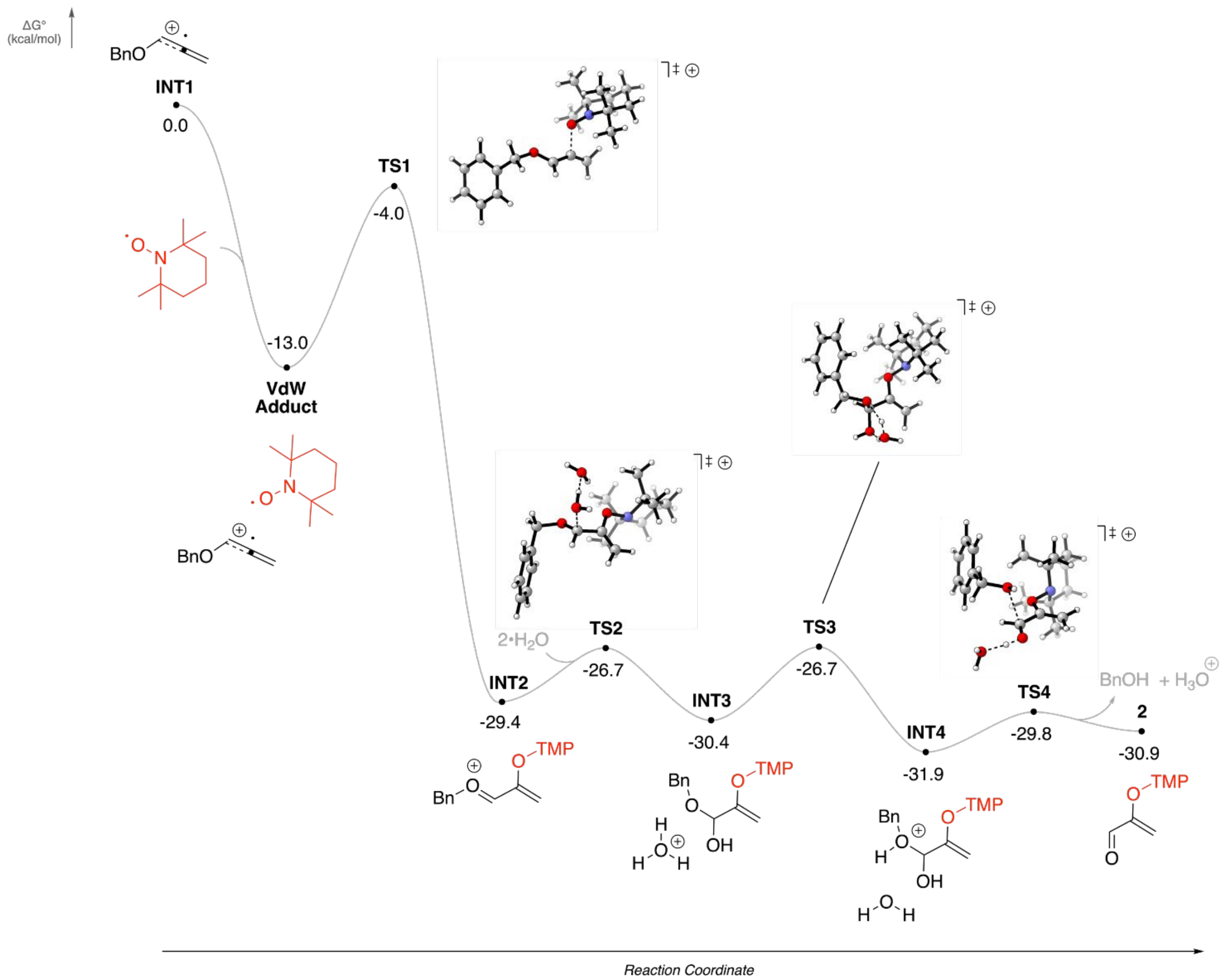

Figure S6. Full Gibbs free energy profile for radical reaction mechanism from **INT1** to **2** at 0°C.
[(PCM-THF:H_2O)-(U)ωB97XD/def2tzvp//(PCM-THF:H_2O)-GD3BJ-(U)B3LYP/6-311++g(d,p)]

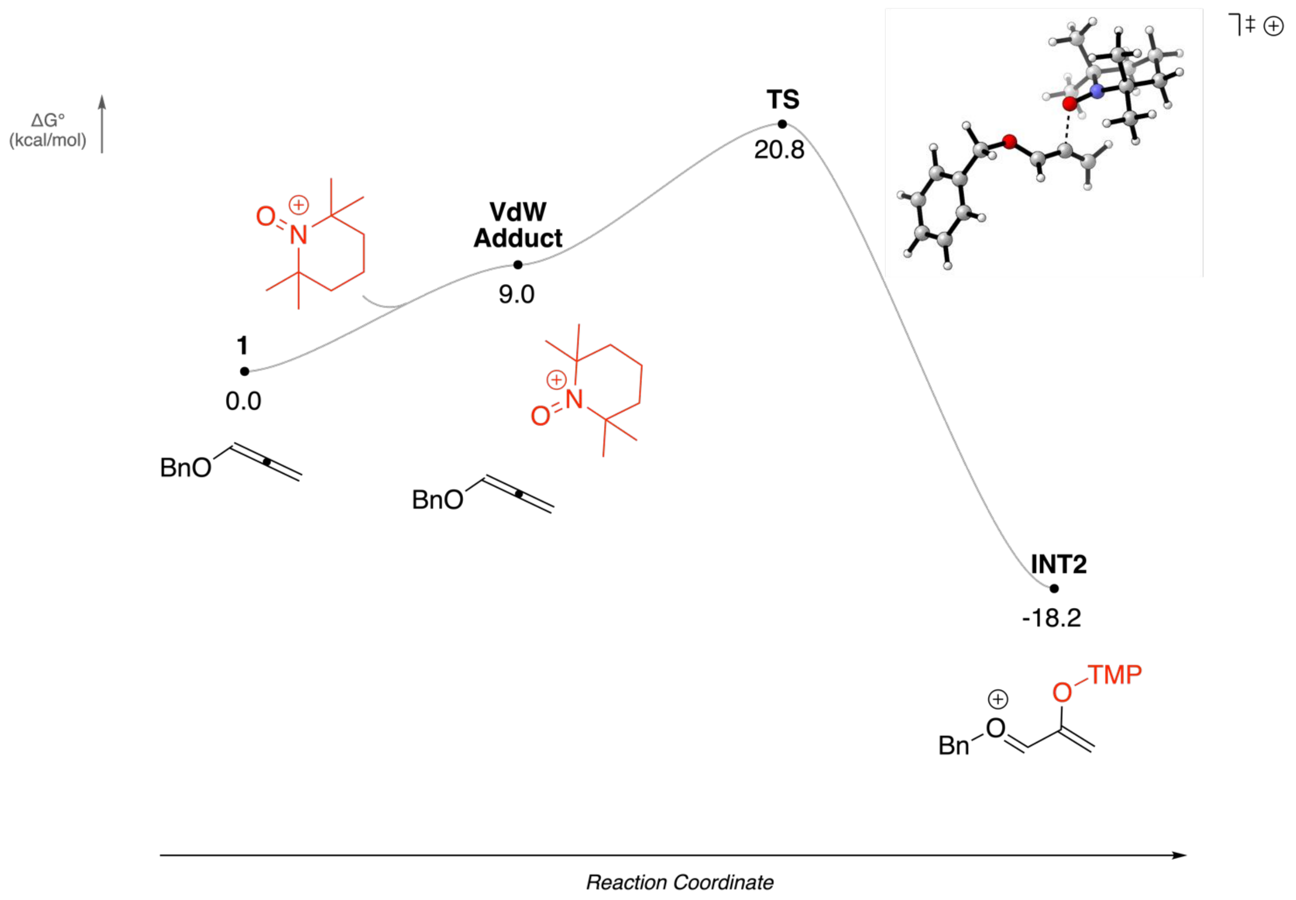

Figure S7. Full Gibbs free energy profile for polar reaction mechanism from **1** to **INT2** at 0 °C.
[(PCM-THF:H_2O)-(U)ωB97XD/def2tzvp//(PCM-THF:H_2O)-GD3BJ-(U)B3LYP/6-311++g(d,p)]

Table S17. Energies, thermal corrections, and free energies for each structure described.

Species	Electronic Energy (a.u.) (U)B3LYP	Zero-Point Energy correction (a.u.) (U)B3LYP	Thermal correction Free Energy (a.u.) (U)B3LYP	Electronic Energy (a.u.) (U)ωB97XD
**2-azide**	-675.30857	0.314404	0.272633	-675.100971
**·N_3-radical (doublet)**	-164.19037	0.009452	-0.012462	-164.143207
**$TS1_{azide}$-radical (doublet)**	-839.50544	0.32572	0.277792	-839.241204
**$INT1_{azide}$-radical (doublet)**	-839.534439	0.329465	0.281869	-839.280158
**$TS2_{azide}$-radical (doublet)**	-839.519937	0.326495	0.278683	-839.256091
**$PROD_{azide}$**	-430.877631	0.069035	0.035332	-430.757172
**·TMP-radical (doublet)**	-408.670769	0.255086	0.219456	-408.536298
**9**	-940.761266	0.403663	0.354859	-940.463076
**I_2**	-22.775072	0.000414	-0.025544	-595.584472
**$INT1_{iodine}$**	-986.375797	0.407802	0.339362	-2131.65749

**$TS1_{iodine}$**	-986.362365	0.403685	0.332963	-2131.639797
**10**	-543.493652	0.145717	0.106023	-829.72527
**I_3^- (anion)**	-34.378282	0.000732	-0.03218	-893.567815
**TMP^+ (cation)**	-408.558378	0.258717	0.222885	-408.428266
**TEMPO (doublet)**	-483.915822	0.260926	0.228367	-483.765541
**INT1 (doublet)**	-462.169659	0.168867	0.134869	-462.014229
**VdW Adduct (singlet)**	-946.127295	0.433925	0.38244	-945.819641
**TS1 (singlet)**	-946.116239	0.433596	0.386632	-945.803236
**INT2 (singlet)**	-1099.123865	0.48616	0.434056	-1098.77701
**H_2O**	-76.466919	0.021199	0.005333	-76.445108
**TS2**	-1099.120136	0.487807	0.438383	-1098.77708
**INT3**	-1099.121338	0.488292	0.437205	-1098.78166
**TS3**	-1099.117477	0.488938	0.44112	-1098.779763
**INT4**	-1099.125376	0.488499	0.439354	-1098.786275
**TS4**	-1099.122471	0.486302	0.435953	-1098.779577
**2**	-675.308592	0.314402	0.277460	-675.100991
**Bn-OH**	-346.908455	0.132707	0.103761	-346.793263
**H_3O^+**	-76.85099	0.032992	0.017363	-76.835993
**1**	-462.394703	0.169769	0.135937	-462.238249
**TEMPO+**	-483.722979	0.26257	0.230531	-483.575924
**VdW Adduct (polar)**	-946.127340	0.43438	0.386176	-945.819566
**TS (polar)**	-946.116252	0.433861	0.386962	-945.801463
**INT2 (polar)**	-946.171057	0.436838	0.39114	-945.867903

Cartesian coordinates

2-azide
(PCM-H_2O:MeCN)-GD3BJ-B3LYP/6-311++g(d,p),
el. energy = -675.308570 a.u.

C	2.12276100	-0.00005300	-0.25194400
C	2.21732200	-0.00005800	-1.58370800
O	0.99361700	-0.00002400	0.51858600
N	-0.24648500	-0.00000500	-0.23146300
C	-0.93504600	1.29363600	0.06174400
C	-0.93507500	-1.29362500	0.06175800
C	-2.28313600	1.24681300	-0.68342300
C	-0.07921600	2.41289500	-0.55038700
C	-1.13714200	1.60340100	1.55833500
C	-2.28316600	-1.24678000	-0.68340500
C	-0.07926800	-2.41290400	-0.55036400
C	-1.13717000	-1.60337200	1.55835400
C	-3.10453000	0.00002900	-0.36654000
H	-2.08283500	1.27571100	-1.75983600
H	-2.83548300	2.15724900	-0.43469100
H	-0.61628300	3.36154400	-0.47672200
H	0.87064500	2.52235400	-0.02416900
H	0.12672400	2.20984900	-1.60239700
H	-0.21082600	1.44655700	2.11199400
H	-1.42387200	2.65220800	1.66725900
H	-1.91918100	1.00071300	2.01525300
H	-2.08286700	-1.27569800	-1.75981800
H	-2.83553400	-2.15720000	-0.43466000
H	0.87059600	-2.52236900	-0.02415300
H	-0.61634900	-3.36154500	-0.47668100
H	0.12666400	-2.20987800	-1.60238000
H	-1.42391500	-2.65217300	1.66729000
H	-0.21084700	-1.44653600	2.11200600
H	-1.91919700	-1.00066700	2.01527100
H	-3.41722500	0.00004000	0.68158700
H	-4.02266400	0.00003600	-0.96066000
H	1.34183600	-0.00004200	-2.21364100
H	3.20383700	-0.00008000	-2.02592400
C	3.31269700	-0.00007500	0.63869100
O	4.46371700	0.00007300	0.25316100
H	3.06930700	0.00013300	1.71666000

·N_3-radical (doublet)
(PCM-H_2O:MeCN)-GD3BJ-UB3LYP/6-311++g(d,p),
el. energy = -164.19037 a.u.

N	0.00000000	0.00000000	-0.00000300
N	0.00000000	0.00000000	1.17603400
N	0.00000000	0.00000000	-1.17603000

$TS1_{azide}$-radical (doublet)
(PCM-H_2O:MeCN)-GD3BJ-UB3LYP/6-311++g(d,p),
im. frequency = -124.43,
el. energy = -839.505440 a.u.

C	2.09276800	-2.23633900	0.87008000
C	1.36884000	-1.19240400	0.09632100
C	1.92509100	-0.59080400	-0.99332500
O	0.17692600	-0.94605000	0.65522600
N	-0.70447800	-0.05326200	-0.09728300
C	-1.04315700	1.07560100	0.82848700
C	-1.80099100	-0.91139400	-0.65896000

C	-2.05511500	1.95984300	0.07365000
C	0.24380600	1.88315200	1.03671800
C	-1.58863200	0.65370100	2.20595400
C	-2.77819100	0.05497900	-1.35775800
C	-1.17465200	-1.81984100	-1.72739200
C	-2.53113300	-1.79992000	0.36588000
C	-3.25925300	1.18660300	-0.45479700
H	-1.53910200	2.43238300	-0.76864000
H	-2.36391200	2.76034500	0.75117500
H	0.01996100	2.77972200	1.61887800
H	0.99763300	1.30950400	1.57724200
H	0.65662300	2.19411700	0.07663900
H	-0.95830100	-0.11327300	2.65688100
H	-1.58313700	1.52479300	2.86505700
H	-2.60958000	0.28136800	2.16396900
H	-2.27418000	0.48468100	-2.22978200
H	-3.61729300	-0.53817500	-1.73091100
H	-0.52498400	-2.57835400	-1.28683700
H	-1.97018600	-2.33973900	-2.26579400
H	-0.59719400	-1.23546600	-2.44538900
H	-3.13413800	-2.53299700	-0.17481700
H	-1.81881500	-2.34307000	0.98766100
H	-3.20041600	-1.24043700	1.01529700
H	-3.86089500	0.79475800	0.36998300
H	-3.91163800	1.85814800	-1.01936700
O	3.19986800	-2.63478500	0.57872100
H	1.36790000	0.13645900	-1.56251000
H	2.82171100	-1.02717600	-1.40387500
H	1.54058500	-2.62471900	1.74258400
N	3.23873300	1.88592600	-0.57049600
N	3.40770400	0.86322600	0.02052100
N	3.07928600	2.88764500	-1.14105200

INT1$_{azide}$-radical (doublet)
(PCM-H_2O:MeCN)-GD3BJ-UB3LYP/6-311++g(d,p),
el. energy = -839.534439 a.u.

C	2.22831100	-2.11235900	0.88754800
C	1.49095400	-1.07155900	0.25251300
C	2.09159900	-0.20432300	-0.80068200
O	0.24428900	-0.92063900	0.72117500
N	-0.64897000	-0.05487000	-0.04847600
C	-1.15991400	0.97359600	0.91383200
C	-1.61512200	-0.96141000	-0.75601000
C	-2.18917300	1.81686400	0.13552600
C	0.02562200	1.87584000	1.28021300
C	-1.76795900	0.40968100	2.21196700
C	-2.62204300	-0.03680000	-1.46919100
C	-0.82418500	-1.72981600	-1.82558300
C	-2.33544900	-1.98338500	0.14344800
C	-3.27306800	0.98027000	-0.53716800
H	-1.65689700	2.39005300	-0.63076600
H	-2.62133400	2.53826100	0.83404500
H	-0.32412100	2.69630700	1.91036700
H	0.79323300	1.33121300	1.83175900
H	0.47000200	2.30456100	0.38143700
H	-1.09980100	-0.31966900	2.67070200
H	-1.90714600	1.23126600	2.91820900
H	-2.73744100	-0.05842900	2.05861500
H	-2.09573600	0.49762800	-2.26703100
H	-3.37137900	-0.67269900	-1.94802800

H	-0.12917100	-2.44627200	-1.38304400
H	-1.52018800	-2.29168300	-2.45207000
H	-0.26749700	-1.04476900	-2.46739600
H	-2.82516300	-2.72433100	-0.49255500
H	-1.62536900	-2.50616300	0.78474000
H	-3.10112100	-1.53298800	0.77081900
H	-3.89581600	0.47924500	0.20919200
H	-3.94090100	1.63056100	-1.10850200
O	3.40840100	-2.37911100	0.60826700
H	1.30765900	0.24933500	-1.40147100
H	2.74457200	-0.81575100	-1.42531900
H	1.67837900	-2.68113400	1.65473100
N	2.88740800	1.95662400	-0.67816900
N	2.93612300	0.85116800	-0.15224300
N	2.91204900	3.01905900	-1.07860500

TS2$_{azide}$-radical (doublet)
(PCM-H_2O:MeCN)-GD3BJ-UB3LYP/6-311++g(d,p),
im. frequency = -410.44,
el. energy = -839.519937 a.u.

C	-2.00745200	2.43698200	0.60675900
C	-1.57733300	1.07251800	0.32488400
C	-2.34630600	0.20765700	-0.62882800
O	-0.53102000	0.65148400	0.92151600
N	0.75769100	-0.06162900	-0.20745700
C	1.20661400	-1.23519600	0.55390500
C	1.67667800	1.05525400	-0.47248300
C	2.37452000	-1.82616300	-0.29551600
C	0.05628200	-2.25005000	0.55524700
C	1.66243000	-1.00302700	2.00775200
C	2.83443000	0.40511200	-1.29875900
C	0.95222200	2.05030000	-1.39375700
C	2.24321400	1.82161400	0.73837800
C	3.45765900	-0.79955700	-0.60504500
H	1.95910600	-2.20863000	-1.23274600
H	2.77180300	-2.68115700	0.25818200
H	0.40796200	-3.20944900	0.94132300
H	-0.76331900	-1.90346600	1.18469500
H	-0.31592800	-2.40236800	-0.45939600
H	0.92272900	-0.41564200	2.55033000
H	1.75625800	-1.97432800	2.49900200
H	2.62552400	-0.50452800	2.08145300
H	2.43212900	0.09805600	-2.26879100
H	3.56943400	1.19309700	-1.48458500
H	0.23839900	2.66455900	-0.84446800
H	1.68053600	2.72659000	-1.84727000
H	0.42433100	1.52209900	-2.18972500
H	2.69665600	2.74833200	0.37901300
H	1.44545400	2.07780100	1.43451900
H	3.00958200	1.26828900	1.27534300
H	3.97706400	-0.49596100	0.30765000
H	4.21475500	-1.24681400	-1.25549500
O	-3.08200600	2.90758000	0.24014300
H	-1.63451900	-0.22309700	-1.33761500
H	-3.08714400	0.80889200	-1.15693200
H	-1.29863800	3.01802900	1.22096700
N	-3.15871900	-1.94147000	-0.45064000
N	-3.04715700	-0.86766000	0.12904700
N	-3.31263200	-2.97859600	-0.88579900

PROD$_{azide}$
(PCM-H_2O:MeCN)-GD3BJ-B3LYP/6-311++g(d,p),
el. energy = -430.877631 a.u.

C	2.23510900	0.38443700	0.13518700
C	0.72853100	0.40889400	-0.16438500
C	0.01009200	-0.92536700	-0.15312200
O	0.18979200	1.46968200	-0.37404000
O	2.83234200	-0.63393700	0.37282800
H	0.09495000	-1.35186900	0.85461700
H	0.52386200	-1.61294100	-0.82578000
H	2.71017500	1.38109600	0.11299600
N	-2.14218900	-0.16996000	0.09551900
N	-1.37912200	-0.84191600	-0.59940000
N	-2.95703900	0.39615800	0.64127800

·TMP-radical (doublet)
(PCM-H_2O:MeCN)-GD3BJ-UB3LYP/6-311++g(d,p),
el. energy = -408.670769 a.u.

C	0.00000000	1.94177600	0.09628500
C	1.24028300	1.22937500	-0.43425300
C	1.26857000	-0.27136100	-0.06436900
C	-1.26857000	-0.27136100	-0.06436900
C	-1.24028300	1.22937500	-0.43425300
H	1.25414300	1.31760700	-1.52673900
H	2.15834500	1.69766000	-0.06731100
H	0.00000000	1.95689600	1.19071700
H	0.00000000	2.98684700	-0.22624200
H	-1.25414300	1.31760700	-1.52673900
H	-2.15834500	1.69766000	-0.06731100
N	0.00000000	-0.96866000	-0.26967300
C	-2.30744500	-0.99313900	-0.93885000
H	-2.38873600	-2.04345800	-0.64923300
H	-3.28929300	-0.52345500	-0.83071400
H	-2.01739400	-0.94661100	-1.99143600
C	-1.68012800	-0.45104600	1.41964800
H	-2.72080800	-0.13842800	1.53816200
H	-1.59696300	-1.49914700	1.71415800
H	-1.06617800	0.14597300	2.09282200
C	2.30744500	-0.99313900	-0.93885000
H	3.28929300	-0.52345500	-0.83071400
H	2.38873600	-2.04345800	-0.64923300
H	2.01739400	-0.94661100	-1.99143600
C	1.68012800	-0.45104600	1.41964800
H	1.59696300	-1.49914700	1.71415800
H	2.72080800	-0.13842800	1.53816200
H	1.06617700	0.14597300	2.09282200

9
(PCM-CH_2Cl_2)-GD3BJ-B3LYP/6-311++g(d,p)&LanL2DZ,
el. energy = -940.761266 a.u.

C	-0.18667300	-1.15849200	-0.16835200
C	0.19453900	-2.42336500	-0.35356000
O	0.62184300	-0.05898200	-0.00838000
N	2.03997200	-0.33557700	-0.04906200
C	2.58724000	-0.02050100	1.30500600
C	2.58708300	0.35541400	-1.25546200
C	4.10911500	-0.24800300	1.22348800
C	1.99329300	-1.04319200	2.28519700
C	2.26425400	1.39292600	1.82927600
C	4.10887600	0.11351700	-1.24335300
C	1.99208100	-0.34195800	-2.48794900

C	2.26605700	1.86044000	-1.35113400
C	4.77150000	0.50689900	0.07413900
H	4.28983000	-1.32047600	1.09426000
H	4.54355000	0.03744500	2.18561800
H	2.46944600	-0.92874200	3.26200700
H	0.91972400	-0.89553600	2.41385200
H	2.16300200	-2.06069600	1.93088900
H	1.20147100	1.61082700	1.71806400
H	2.50828900	1.44064600	2.89333800
H	2.83150400	2.17369500	1.32725300
H	4.28941200	-0.95094900	-1.42729300
H	4.54310600	0.66291700	-2.08333300
H	0.91839200	-0.16374100	-2.56758300
H	2.46744300	0.04870000	-3.39091000
H	2.16187100	-1.41839100	-2.44165300
H	2.50672400	2.21079400	-2.35780400
H	1.20447400	2.03974700	-1.17779100
H	2.83724700	2.46361500	-0.64871700
H	4.70476200	1.58720800	0.23243500
H	5.83803100	0.26746700	0.03899300
H	1.23905300	-2.68689100	-0.39253200
H	-0.56548700	-3.18259900	-0.46447300
C	-1.58819600	-0.73191100	-0.10554400
C	-3.73793900	-0.65766900	-0.08923900
C	-3.29480800	0.67681400	0.09145900
C	-5.10949900	-0.93631600	-0.12291400
C	-4.17745000	1.74561000	0.23944200
C	-5.99376900	0.12527100	0.02421700
H	-5.46204100	-1.95152600	-0.25998600
C	-5.53535800	1.44590900	0.20264900
H	-3.82652300	2.76106700	0.37635700
H	-7.06082200	-0.06223800	0.00192700
H	-6.25747500	2.24608400	0.31355600
N	-1.91740500	0.59094900	0.07595300
N	-2.65013000	-1.50741000	-0.20832000
H	-1.24886100	1.33934200	0.17637400

I_2
(PCM-CH_2Cl_2)-GD3BJ-B3LYP/LanL2DZ,
el. energy = -22.775072 a.u.

I	0.00000000	0.00000000	1.43200200
I	0.00000000	0.00000000	-1.43200200

INT1$_{iodine}$
(PCM-CH_2Cl_2)-GD3BJ-B3LYP/6-311++g(d,p)&LanL2DZ,
el. energy = -986.375797 a.u.

C	1.23354300	-0.32827600	-1.55596600
C	1.67737000	-1.62011400	-2.11083000
O	1.78382500	0.25340000	-0.53683100
N	3.13007900	-0.25245800	-0.11287600
C	3.03098700	-0.51056200	1.36209500
C	4.10886800	0.76590900	-0.63233200
C	4.45127600	-0.94936400	1.77794200
C	2.07686700	-1.69360400	1.54944000
C	2.53351400	0.66932700	2.21315900
C	5.48589200	0.28063500	-0.12718800
C	4.10406900	0.67967500	-2.16529800
C	3.83552100	2.22240700	-0.21761900
C	5.53365900	0.04897500	1.37944100
H	4.66552900	-1.91734400	1.31372500

H	4.43583600	-1.10692200	2.85919400
H	2.09618900	-2.00851400	2.59447200
H	1.04813800	-1.42407000	1.30947900
H	2.37824300	-2.53856600	0.93008900
H	1.65455200	1.13776900	1.77538700
H	2.24687700	0.28811900	3.19515900
H	3.29015900	1.43475600	2.36672800
H	5.73246700	-0.65324000	-0.64215000
H	6.22283200	1.02512300	-0.43874500
H	3.18772900	1.08430700	-2.60186400
H	4.93022300	1.27632100	-2.55615600
H	4.24163100	-0.34835200	-2.50370500
H	4.45608900	2.87794800	-0.83205800
H	2.79348500	2.49708700	-0.38244400
H	4.07795600	2.42036000	0.82280600
H	5.41064400	0.99093000	1.92026300
H	6.51594900	-0.34001100	1.65929100
H	2.65706100	-1.89808300	-1.75038200
H	1.58077300	-1.63759400	-3.18939700
I	0.29761200	-3.18409600	-1.41012400
I	-2.63379500	-2.42148200	1.75492600
C	0.14296400	0.36882800	-2.12769600
C	-1.49413400	0.86192700	-3.41714000
C	-1.38627100	1.91725400	-2.44930800
C	-2.50208900	0.91046700	-4.40683800
C	-2.26549600	3.00813800	-2.42911800
C	-3.35729300	1.98578500	-4.38277400
H	-2.58897400	0.12020700	-5.14098800
C	-3.24083400	3.01756400	-3.40436500
H	-2.18467000	3.78707800	-1.68334100
H	-4.14842700	2.05974900	-5.11830700
H	-3.94996500	3.83590600	-3.43168600
N	-0.33164600	1.57780600	-1.66574100
N	-0.54200300	-0.07249600	-3.19210100
H	-0.02687000	2.08381900	-0.83348300
I	-1.60767300	0.44363900	1.59394300
I	-0.48255000	3.39488700	1.38793700

$TS1_{iodine}$
(PCM-CH_2Cl_2)-GD3BJ-B3LYP/6-311++g(d,p)&LanL2DZ,
im. frequency = -174.03,
el. energy = -986.362365 a.u.

C	0.01082400	-1.01223400	1.69047300
C	0.47523400	-0.38159100	2.96665500
O	0.76261700	-1.21489800	0.74193400
N	3.16524200	-1.16552100	0.98845900
C	3.35600900	-0.26091600	-0.00824600
C	3.35131600	-2.57948400	0.90516200
C	5.08279900	-0.33367600	0.31887100
C	2.93873700	1.14394900	0.40363300
C	3.11453300	-0.53071200	-1.48922100
C	4.94034000	-2.67294500	1.09690800
C	2.72262500	-3.22379700	2.15447100
C	2.90553900	-3.33625400	-0.35609000
C	5.63086500	-1.71946400	0.14270700
H	5.23892400	0.08078500	1.31047700
H	5.36591200	0.38668900	-0.44912100
H	3.46315000	1.89417600	-0.18852900
H	1.87022500	1.24505100	0.20638000
H	3.11683400	1.31710900	1.46363500

H	2.07380300	-0.82371300	-1.62444900
H	3.27936300	0.39391500	-2.04158500
H	3.75142600	-1.30052700	-1.91178700
H	5.17395200	-2.41653900	2.13088200
H	5.19563600	-3.72029200	0.93035100
H	1.64496100	-3.31904000	2.02331300
H	3.13767200	-4.22289300	2.29425700
H	2.92660600	-2.62724500	3.04374300
H	2.91938800	-4.40297800	-0.12623900
H	1.88753300	-3.05725600	-0.62527000
H	3.55385600	-3.17685300	-1.21311900
H	5.55397500	-2.05078700	-0.89320900
H	6.70083100	-1.68111000	0.38176900
H	1.55401000	-0.43678100	3.05188900
H	-0.04803400	-0.76348800	3.83563200
I	-0.02034900	1.75094800	2.88743700
I	-0.39335100	3.97877800	-0.88745400
C	-1.39603300	-1.40097300	1.59761700
C	-3.45088600	-1.82075000	2.03857700
C	-3.24736900	-2.15344300	0.66924400
C	-4.71802300	-1.98874100	2.62139200
C	-4.27599400	-2.64150800	-0.14264400
C	-5.73461500	-2.47359200	1.81850700
H	-4.88320400	-1.73929500	3.66217100
C	-5.51581700	-2.79345000	0.45643400
H	-4.11076600	-2.88261600	-1.18476900
H	-6.72530500	-2.61164400	2.23439900
H	-6.34488200	-3.16698000	-0.13257700
N	-1.92648500	-1.88165400	0.42906200
N	-2.27773000	-1.35437500	2.58530000
H	-1.43302700	-1.96289000	-0.45837600
I	-0.54258200	1.09775600	-1.89432000
I	-0.56884000	-1.87177000	-2.95437600

10
(PCM-CH_2Cl_2)-GD3BJ-B3LYP/6-311++g(d,p)&LanL2DZ,
el. energy = -543.493652 a.u.

C	-0.68146100	1.45025000	0.52678500
C	-1.73939000	0.62833800	1.20644500
O	-0.88552200	2.58676700	0.13011400
H	-2.50337800	1.26294300	1.64092200
H	-1.33099800	-0.09070400	1.90684300
I	-2.76874500	-0.57879200	-0.31083500
C	0.63913800	0.82257300	0.36172300
C	2.31272800	-0.51714600	0.43786900
C	2.74162600	0.62579800	-0.29091900
C	3.19870300	-1.58315700	0.65745000
C	4.03307200	0.73736900	-0.81227600
C	4.47865700	-1.47319500	0.14182800
H	2.88074200	-2.45755700	1.21155400
C	4.88837700	-0.32950300	-0.58211100
H	4.35028800	1.61087900	-1.36752500
H	5.18738400	-2.27847000	0.29231200
H	5.90009000	-0.28769700	-0.96739100
N	1.64876300	1.45766200	-0.31670300
N	1.00021300	-0.35966900	0.82839300
H	1.57202100	2.36851700	-0.74595100

I_3^- (anion)
(PCM-CH_2Cl_2)-GD3BJ-B3LYP/LanL2DZ,

el. energy = -34.378282 a.u.

I	0.00000000	0.00000000	-3.11949300
I	0.00000000	0.00000000	0.00016200
I	0.00000000	0.00000000	3.11933200

TMP^+ (cation)
(PCM-CH_2Cl_2)-GD3BJ-B3LYP/6-311++g(d,p),
el. energy = -408.558378 a.u.

N	0.28786400	0.32528600	-0.04007700
C	1.54894100	0.04399400	0.00967700
C	-0.89758900	-0.64752200	-0.04069600
C	2.55930000	1.14570100	-0.06576000
H	2.62305000	1.64462600	0.90760000
H	3.54105900	0.73706200	-0.29653300
H	2.29503800	1.89703000	-0.80937400
C	-0.19403700	1.73850900	-0.11472400
H	0.37046400	2.35610300	0.57958400
C	2.09855600	-1.33337900	0.14823300
H	1.38100700	-2.08392500	0.44991700
H	2.54267300	-1.62446700	-0.80966600
C	-2.06540900	0.29969700	-0.38887200
C	-0.75711100	-1.72386900	-1.11869700
H	-0.06917500	-2.52395400	-0.85454100
H	-1.74030900	-2.17462900	-1.26821400
H	-0.44200000	-1.28297700	-2.06637000
C	-1.07482300	-1.22820200	1.36697800
H	-0.21748500	-1.81926500	1.68672900
H	-1.94990100	-1.88032300	1.36420900
H	-3.00353200	-0.09479400	-0.00000200
H	2.91494700	-1.30188000	0.87320600
H	-1.23704500	-0.43939600	2.10290800
C	-1.67980000	1.65147100	0.20206600
H	-1.84328900	1.67605000	1.28095300
H	-0.02365300	2.09754400	-1.13244700
H	-2.22802800	2.48156300	-0.24134600
H	-2.15703300	0.38023000	-1.47530300

TEMPO (doublet)
(PCM-THF:H_2O)-GD3BJ-UB3LYP/6-311++g(d,p),
el. energy = -483.915822 a.u.

O	-0.00021700	-2.03269300	-0.08278000
N	-0.00006300	-0.75579500	-0.20415400
C	1.32717800	-0.07385400	-0.02444600
C	-1.32719500	-0.07371400	-0.02445000
C	1.24080000	1.38387200	-0.50636200
C	2.35545100	-0.82674800	-0.87638700
C	1.73367600	-0.15023900	1.45859300
C	-1.24063500	1.38398800	-0.50642200
C	-2.35547100	-0.82662500	-0.87636700
C	-1.73365700	-0.15006200	1.45860100
C	0.00010500	2.11734600	-0.00880500
H	1.23534300	1.39044800	-1.60178600
H	2.15559300	1.89234400	-0.19024400
H	3.31123100	-0.30009300	-0.82589600
H	2.49801100	-1.84506400	-0.51719700
H	2.03598800	-0.86421000	-1.92034400
H	1.68053000	-1.18291300	1.80717600
H	2.76173000	0.20221500	1.57057000
H	1.09546500	0.46419700	2.09357900
H	-1.23511500	1.39054600	-1.60184700

H	-2.15539200	1.89255600	-0.19036000
H	-2.49792200	-1.84499500	-0.51726700
H	-3.31128500	-0.30005300	-0.82572900
H	-2.03610700	-0.86396000	-1.92035900
H	-2.76170000	0.20241900	1.57058500
H	-1.68052900	-1.18272800	1.80721500
H	-1.09541700	0.46437400	2.09355800
H	0.00008000	2.19014000	1.08273300
H	0.00016500	3.14310100	-0.38679300

INT1 (doublet)
(PCM-THF:H_2O)-GD3BJ-UB3LYP/6-311++g(d,p),
el. energy = -462.169659 a.u.

C	3.40974000	-0.40250100	-0.22201100
C	4.37714400	-0.84586800	-0.93846700
H	5.25482800	-0.23276400	-1.14148600
H	4.34794500	-1.84648300	-1.36871600
C	2.40089900	0.06430700	0.56962300
C	0.29437100	1.20919400	0.87084400
O	1.43980400	0.74040100	0.02693600
H	2.39128000	-0.11981800	1.64142000
H	0.54088300	0.99527800	1.90888700
H	0.27656600	2.28129400	0.69111900
C	-0.93622900	0.51290200	0.39145100
C	-1.69858900	1.06892700	-0.64137200
C	-1.31973000	-0.70653100	0.96084600
C	-2.83659700	0.41210500	-1.09896300
H	-1.40269500	2.01525900	-1.07941700
C	-2.45891700	-1.36105500	0.50244900
H	-0.73549400	-1.13736000	1.76609700
C	-3.21580000	-0.80340800	-0.52847900
H	-3.42834500	0.84762000	-1.89477500
H	-2.75822500	-2.30088200	0.95002300
H	-4.10291200	-1.31377400	-0.88415500

VdW Adduct (singlet)
(PCM-THF:H_2O)-GD3BJ-UB3LYP/6-311++g(d,p),
el. energy = -946.127295 a.u.

C	-0.42098000	-1.29451500	1.84709100
C	0.41835800	-1.69063200	2.75816900
O	0.75625100	0.31921500	-0.37440100
N	1.94895300	0.30861500	-0.29960500
C	2.62984500	1.43891800	0.47635100
C	2.70361100	-0.82070500	-1.00645000
C	3.85286300	0.86931500	1.21840000
C	1.60264500	2.01793600	1.44623800
C	3.02951900	2.50518800	-0.56682700
C	3.92266100	-1.21756600	-0.15353200
C	1.73372900	-1.98841600	-1.17260700
C	3.11543400	-0.27143700	-2.39007400
C	4.73239400	-0.03334500	0.36117800
H	3.50298700	0.31458600	2.09298800
H	4.41482700	1.72844600	1.59015300
H	2.11920800	2.75519700	2.06188800
H	0.78565100	2.51568300	0.92548900
H	1.19164300	1.24809200	2.09953700
H	2.17929300	2.77679800	-1.19283700
H	3.34099500	3.38668600	-0.00453800
H	3.85823000	2.19378600	-1.19639200
H	3.57492200	-1.81787500	0.69163400

H	4.53468900	-1.87196800	-0.77753600
H	0.92126700	-1.75045700	-1.85717300
H	2.30089500	-2.82331800	-1.58584300
H	1.31275100	-2.29577000	-0.21532100
H	3.48027400	-1.12304200	-2.96619500
H	2.25950800	0.15729000	-2.91212000
H	3.91165900	0.46585400	-2.33487800
H	5.18222200	0.52434200	-0.46353400
H	5.56133400	-0.40407100	0.96726200
H	1.49545800	-1.59746100	2.63079000
H	0.08249600	-2.13782400	3.69120400
C	-1.28628900	-0.87073400	0.95307300
C	-2.43889500	-1.00326300	-1.12153100
O	-1.77463500	-1.68452900	-0.02372300
H	-1.66423400	0.14672200	0.98236900
H	-1.71756200	-0.36280000	-1.63325000
H	-2.72167000	-1.81483600	-1.79070000
C	-3.64059300	-0.21198800	-0.67776000
C	-4.73846300	-0.86263400	-0.10293200
C	-3.66919700	1.17707600	-0.81847500
C	-5.84372500	-0.13411500	0.32536400
H	-4.72216700	-1.94100100	0.01029100
C	-4.77878800	1.90937500	-0.39495500
H	-2.82054000	1.68778200	-1.26072400
C	-5.86599900	1.25480100	0.17926000
H	-6.68979200	-0.64643800	0.76832100
H	-4.79058700	2.98688500	-0.50970700
H	-6.72815000	1.82136000	0.51116800

TS1 (singlet)
(PCM-THF:H_2O)-GD3BJ-UB3LYP/6-311++g(d,p),
im. frequency = -354.90,
el. energy = -946.116239 a.u.

C	-0.00380100	0.83927900	-1.08513300
C	-0.66507900	0.76151200	-2.20527300
O	-1.02698700	0.20018300	0.36044200
N	-2.24243400	-0.02904900	0.06736900
C	-3.23118800	1.04751600	0.45270500
C	-2.61529800	-1.47997900	-0.17056000
C	-4.54438600	0.81801000	-0.31147000
C	-2.65117800	2.41500500	0.07958700
C	-3.43989700	0.99947800	1.98012400
C	-3.96933700	-1.53916600	-0.89170000
C	-1.54101800	-2.16455400	-1.02120500
C	-2.66642400	-2.17920900	1.20368600
C	-5.02981300	-0.62799500	-0.28080600
H	-4.39838200	1.12165500	-1.35335900
H	-5.29216100	1.49186900	0.11351600
H	-3.38153800	3.17665200	0.35732200
H	-1.72415300	2.62151200	0.61413700
H	-2.46859600	2.49331700	-0.99204100
H	-2.47744100	1.02608000	2.49358200
H	-4.01740400	1.87587800	2.28057100
H	-3.98446800	0.11349800	2.29987900
H	-3.81972700	-1.25798900	-1.93942200
H	-4.29779700	-2.58130800	-0.88741300
H	-0.55512000	-2.09086400	-0.56338700
H	-1.80445000	-3.22063100	-1.09915300
H	-1.49622300	-1.75781400	-2.03013300
H	-2.81233300	-3.24933500	1.04370000

H	-1.72523400	-2.03301300	1.73610500
H	-3.48093300	-1.81925600	1.82814500
H	-5.26928900	-0.93777500	0.74006000
H	-5.95660300	-0.71300300	-0.85296800
H	-1.71682300	0.46475600	-2.16816600
H	-0.21967300	0.93482400	-3.17751100
C	1.13597900	1.26703300	-0.45012600
C	2.94163400	0.97210600	1.10093800
O	1.78064100	0.46010700	0.32997400
H	1.43450400	2.31408100	-0.47634900
H	2.95370300	2.05745700	1.00476000
H	2.71977000	0.68836900	2.12699500
C	4.19077600	0.33145900	0.58486800
C	4.60936700	-0.90270500	1.09108400
C	4.93299500	0.95119300	-0.42494200
C	5.75597700	-1.51296900	0.58917900
H	4.03922300	-1.38209100	1.87893000
C	6.08028900	0.34162100	-0.92597800
H	4.61662700	1.91238000	-0.81445400
C	6.49123900	-0.89164900	-0.42026500
H	6.07760800	-2.46802400	0.98685700
H	6.65434600	0.82888800	-1.70489300
H	7.38534800	-1.36499700	-0.80850300

INT2 (singlet)
(PCM-THF:H_2O)-GD3BJ-B3LYP/6-311++g(d,p),
el. energy = -1099.123865 a.u.

C	-0.00591200	0.04836600	-0.81002700
C	-0.09147900	-0.26625400	-2.11534200
O	-1.01343100	0.20238100	0.08901700
N	-2.29343500	-0.30246200	-0.41232300
C	-3.25414900	0.83939500	-0.48140700
C	-2.62299200	-1.52062500	0.38932000
C	-4.56523800	0.23391000	-1.02411900
C	-2.70822800	1.83997600	-1.51022100
C	-3.51366200	1.59036000	0.83823900
C	-3.97317300	-2.03010200	-0.15629300
C	-1.55123400	-2.57853100	0.08380800
C	-2.67280100	-1.30860900	1.91407300
C	-5.05657300	-0.95626000	-0.20404100
H	-4.39568500	-0.08813700	-2.05672800
H	-5.31376700	1.03009700	-1.05228900
H	-3.44174700	2.63368500	-1.66595800
H	-1.78236900	2.30515100	-1.16644200
H	-2.52514300	1.35350100	-2.46940300
H	-2.58666600	1.90862100	1.31159500
H	-4.09071500	2.49104000	0.61611300
H	-4.08353300	1.00634900	1.55615200
H	-3.80980700	-2.41412200	-1.16856200
H	-4.28298900	-2.87618700	0.46288600
H	-0.58655800	-2.31495100	0.52119000
H	-1.85824900	-3.53407300	0.51434700
H	-1.42821300	-2.70859400	-0.99255300
H	-2.67800400	-2.28431200	2.40536600
H	-1.79146100	-0.76550100	2.25805300
H	-3.56089900	-0.77291300	2.24122100
H	-5.33360800	-0.64101500	0.80575700
H	-5.96292200	-1.36798000	-0.65607100
H	-1.04184400	-0.47458300	-2.58053200
H	0.81419300	-0.33964200	-2.70070600

C	1.31257200	0.16752000	-0.25176300
C	2.84985400	0.28672800	1.61157000
O	1.46941300	0.14454200	1.00269800
H	2.17074600	0.14411900	-0.91329400
H	2.90139000	1.34320700	1.86334300
H	2.75515000	-0.31496900	2.51031700
C	3.94447400	-0.15984400	0.70771500
C	4.29004500	-1.51517400	0.64279700
C	4.62073900	0.76737300	-0.09336000
C	5.29961400	-1.93784400	-0.21581500
H	3.76929300	-2.23303900	1.26641100
C	5.63343700	0.34238900	-0.94956600
H	4.34238800	1.81352900	-0.04941500
C	5.97141400	-1.00889800	-1.01164500
H	5.56690400	-2.98656100	-0.26072600
H	6.15696800	1.06296200	-1.56597800
H	6.76006000	-1.33878500	-1.67743100
O	1.71688300	2.59805600	-0.39393300
H	1.41875200	2.99268400	-1.22139900
H	1.04397900	2.83623300	0.27805100
H	-0.53669400	1.79760900	1.47917300
O	-0.18777400	2.69389500	1.59072900
H	0.12412200	2.73955600	2.50215100

H_2O
(PCM-THF:H_2O)-GD3BJ-B3LYP/6-311++g(d,p),
el. energy = -76.466919 a.u.

O	0.00000000	0.00000000	0.11795700
H	0.00000000	0.76214500	-0.47182800
H	0.00000000	-0.76214500	-0.47182800

TS2 (singlet)
(PCM-THF:H_2O)-GD3BJ-B3LYP/6-311++g(d,p),
im. frequency = -249.29,
el. energy = -1099.120136 a.u.

C	-0.02499300	0.29588200	0.89912300
C	-0.01179800	-0.21296500	2.13219000
O	1.01334700	0.41794300	0.02079500
N	2.19132300	-0.34766400	0.41505700
C	3.31346500	0.60811400	0.65774600
C	2.33545400	-1.45846600	-0.57565800
C	4.52740500	-0.26396800	1.03813600
C	2.93141000	1.47264500	1.86790800
C	3.66675700	1.54988400	-0.50862700
C	3.60074600	-2.23827000	-0.16532300
C	1.12002900	-2.38398900	-0.40744100
C	2.40611000	-1.02140400	-2.05126700
C	4.82947200	-1.35073300	0.01038100
H	4.32419900	-0.73544500	2.00525800
H	5.38483000	0.39972700	1.17654400
H	3.76980000	2.12319000	2.12641200
H	2.07039700	2.10689800	1.64926800
H	2.69957300	0.85165100	2.73395000
H	2.80883200	2.14911000	-0.81019000
H	4.44084400	2.24310300	-0.17088500
H	4.05227600	1.02951800	-1.38133900
H	3.39614500	-2.75114400	0.78025900
H	3.77444200	-3.01068300	-0.91925200
H	0.20156300	-1.90938600	-0.75673400
H	1.27359700	-3.28923700	-0.99921300

H	0.99319300	-2.66930700	0.63781600
H	2.30107400	-1.90526300	-2.68471200
H	1.58747300	-0.34323000	-2.29628700
H	3.34693400	-0.54238700	-2.31228100
H	5.12775700	-0.90786800	-0.94418800
H	5.67631100	-1.95465800	0.34740000
H	0.88649900	-0.63521800	2.55308800
H	-0.92652600	-0.23538100	2.70750500
C	-1.30631600	0.74254700	0.32336000
C	-2.86220900	0.62794400	-1.48910200
O	-1.49929100	0.43696200	-0.93319700
H	-2.15745400	0.69886500	0.99540100
H	-3.02322200	1.70141600	-1.57530800
H	-2.77327200	0.18881900	-2.47918000
C	-3.91144700	-0.03728100	-0.65317400
C	-3.99181500	-1.43440700	-0.60921400
C	-4.80075400	0.72690100	0.10688400
C	-4.94702700	-2.05598100	0.18795600
H	-3.30461600	-2.03085100	-1.19892700
C	-5.76292100	0.10374700	0.90095000
H	-4.74135200	1.80917100	0.07515400
C	-5.83435700	-1.28675800	0.94368600
H	-5.00536800	-3.13743600	0.21717600
H	-6.45123900	0.70281200	1.48486700
H	-6.58035500	-1.77239800	1.56159800
O	-1.23731300	2.54796400	0.28041300
H	-0.94560200	2.91694700	1.12820800
H	-0.55312200	2.77785200	-0.43450000
H	0.86389800	1.87988300	-1.54233100
O	0.51148500	2.78230200	-1.58652000
H	0.17415000	2.90254200	-2.48299200

INT3
(PCM-THF:H_2O)-GD3BJ-B3LYP/6-311++g(d,p),
el. energy = -1099.121338 a.u.

C	-0.06192600	0.25858500	0.88747200
C	-0.12273500	-0.42670400	2.02558300
O	1.01762100	0.45004000	0.06880600
N	2.16320000	-0.38396400	0.40519800
C	3.28770900	0.51293900	0.81031500
C	2.32677500	-1.36274800	-0.71347200
C	4.48957700	-0.41168800	1.08983800
C	2.88203800	1.19663400	2.12420600
C	3.67025400	1.61250000	-0.19806500
C	3.57899600	-2.19826300	-0.38195600
C	1.10259400	-2.29168400	-0.69221300
C	2.43313000	-0.74462000	-2.12049900
C	4.80785200	-1.34962000	-0.07051000
H	4.26476700	-1.01067900	1.97846700
H	5.34648300	0.22070500	1.33606100
H	3.72254200	1.78415100	2.50053700
H	2.03848200	1.87350300	1.97798600
H	2.61158900	0.45799400	2.87956600
H	2.82097700	2.25902800	-0.41682300
H	4.44455900	2.24019400	0.24934000
H	4.06538700	1.22396600	-1.13338000
H	3.35314300	-2.82538800	0.48681600
H	3.76334800	-2.87044900	-1.22411600
H	0.19244000	-1.76253700	-0.97851000
H	1.25651200	-3.10623800	-1.40387000

H	0.96134900	-2.71927100	0.30134700
H	2.40363500	-1.54788000	-2.86030200
H	1.58290300	-0.09098700	-2.32355200
H	3.35262400	-0.18652300	-2.27909900
H	5.12265400	-0.78312600	-0.95169200
H	5.64745900	-1.99751700	0.19552200
H	0.73653400	-0.95326200	2.40886500
H	-1.05943600	-0.47976400	2.56231000
C	-1.28640000	0.90352600	0.32394100
C	-2.89318000	0.74460300	-1.43648200
O	-1.53432100	0.50288100	-0.94212900
H	-2.13264100	0.82883600	1.00401500
H	-3.07696000	1.81975300	-1.44717300
H	-2.85021100	0.38070900	-2.46108500
C	-3.92257700	0.01592600	-0.62024100
C	-3.92614600	-1.38363100	-0.58427800
C	-4.86705600	0.72200000	0.12768300
C	-4.86086200	-2.06357100	0.18929700
H	-3.19265500	-1.93640100	-1.16060400
C	-5.80950700	0.04100500	0.89894700
H	-4.86585400	1.80632300	0.10640700
C	-5.80556400	-1.35131400	0.93180700
H	-4.85813000	-3.14694300	0.21154300
H	-6.53985100	0.59744600	1.47423700
H	-6.53497800	-1.88229700	1.53207700
O	-1.07688700	2.45083600	0.22256800
H	-0.84255500	2.85303300	1.07554100
H	-0.34583000	2.67014400	-0.53993000
H	1.03146900	1.91955400	-1.57211500
O	0.60048600	2.78853900	-1.53625400
H	0.24481800	2.97005000	-2.41695700

TS3
(PCM-THF:H_2O)-GD3BJ-B3LYP/6-311++g(d,p),
im. frequency = -120.23,
el. energy = -1099.117477 a.u.

C	0.32631900	-1.60066600	0.75137900
C	-0.20852400	-2.72937700	1.20712900
O	-0.30856800	-0.51604300	0.22421700
N	-1.75616800	-0.59864400	0.22965100
C	-2.19757100	-0.60088100	-1.19792400
C	-2.24340200	0.46968600	1.15492300
C	-3.73813200	-0.60753700	-1.17544800
C	-1.71096100	-1.91756300	-1.82166400
C	-1.65756700	0.56347100	-2.05088500
C	-3.78260500	0.42880400	1.09286900
C	-1.80267800	0.07744300	2.57336300
C	-1.70836400	1.88474900	0.85916500
C	-4.33164500	0.51524400	-0.32869500
H	-4.07367900	-1.57017700	-0.77479100
H	-4.08898400	-0.54965100	-2.20962600
H	-2.12291400	-2.01603000	-2.82887100
H	-0.62256300	-1.94174400	-1.89727700
H	-2.03800300	-2.77125800	-1.22639700
H	-0.58526100	0.67916100	-1.90117800
H	-1.83483700	0.34233800	-3.10632300
H	-2.13924900	1.51328800	-1.82909200
H	-4.12112200	-0.50842000	1.54766800
H	-4.16486700	1.24387000	1.71372200
H	-0.71964400	0.14910000	2.68793100

H	-2.26266500	0.75564300	3.29609300
H	-2.11079200	-0.94295200	2.80556300
H	-1.91495000	2.52803900	1.71819500
H	-0.63063400	1.86656400	0.70074100
H	-2.17403000	2.34249500	-0.01107100
H	-4.10670200	1.49042500	-0.76995400
H	-5.42184500	0.43028500	-0.31104700
H	-1.27639800	-2.87819600	1.17949200
H	0.42954600	-3.49282900	1.62426000
C	1.80029400	-1.27549300	0.76583000
C	3.25293600	-0.01211000	-0.72563100
O	2.23431300	-1.05311300	-0.56066800
H	1.98342900	-0.39252500	1.37831800
H	4.09526400	-0.22812900	-0.06478300
H	3.57867700	-0.13004300	-1.75738100
C	2.68721700	1.35648600	-0.47798900
C	1.87275500	1.95931100	-1.44215300
C	2.93019100	2.02674000	0.72383700
C	1.30140300	3.20448900	-1.20438900
H	1.68573000	1.44678400	-2.37888500
C	2.35822600	3.27591400	0.96487000
H	3.57302400	1.57559300	1.47223700
C	1.54118400	3.86393800	0.00221900
H	0.66707100	3.66009200	-1.95542400
H	2.55073900	3.78591300	1.90131300
H	1.09263200	4.83252600	0.18911400
O	2.53309400	-2.38745700	1.32195400
H	2.66857600	-2.71129900	-1.25086800
H	3.26523800	-2.07939800	1.87218100
O	2.99774500	-3.53895800	-0.80931100
H	2.87819600	-3.26897800	0.20181400
H	2.44705800	-4.30130100	-1.05761300

INT4
(PCM-THF:H_2O)-GD3BJ-B3LYP/6-311++g(d,p),
el. energy = -1099.125376 a.u.

C	0.12654500	-1.57939300	0.96204100
C	-0.51200800	-2.63528700	1.45497200
O	-0.38484100	-0.48790800	0.32606000
N	-1.83319800	-0.44228100	0.27086900
C	-2.21809000	-0.51422800	-1.17138900
C	-2.25244600	0.73429300	1.09305400
C	-3.75212900	-0.37894900	-1.21735500
C	-1.83433300	-1.91387000	-1.67413200
C	-1.53921000	0.52795100	-2.08144900
C	-3.78500400	0.83021400	0.96387500
C	-1.90662500	0.41213300	2.55466600
C	-1.57386900	2.06700700	0.71879600
C	-4.26867700	0.85586900	-0.48362800
H	-4.19149900	-1.27291600	-0.76187000
H	-4.05650800	-0.37031000	-2.26765200
H	-2.21345800	-2.05160500	-2.68944200
H	-0.75114000	-2.04386900	-1.69838100
H	-2.26368500	-2.68618800	-1.03445700
H	-0.46853700	0.56466800	-1.88600800
H	-1.68695900	0.23837900	-3.12469200
H	-1.94495900	1.52979900	-1.95847500
H	-4.22822500	-0.03366800	1.47080900
H	-4.11142400	1.72267300	1.50494600
H	-0.82723400	0.39625700	2.71565800

H	-2.33134100	1.18031100	3.20525300
H	-2.31620500	-0.55686300	2.84350800
H	-1.75318300	2.79153600	1.51697800
H	-0.49675300	1.93754900	0.61417900
H	-1.95846200	2.49536200	-0.20429400
H	-3.93632800	1.76933700	-0.98506800
H	-5.36184700	0.87208100	-0.50882400
H	-1.58531700	-2.71213000	1.38181100
H	0.05365900	-3.41022400	1.94932400
C	1.61137700	-1.37484100	1.07512300
C	3.30541900	-0.40029600	-0.61748500
O	2.11883600	-1.27694400	-0.34857000
H	1.83770500	-0.39380600	1.48999000
H	4.10060600	-0.69936400	0.06334100
H	3.57553300	-0.66132400	-1.63799400
C	2.89955900	1.02892200	-0.46929600
C	2.12343600	1.63708100	-1.46174700
C	3.25451000	1.75621600	0.67001700
C	1.69338000	2.94972800	-1.30825900
H	1.85327300	1.07725200	-2.34938300
C	2.82713300	3.07442500	0.82168900
H	3.86691800	1.29591900	1.43753500
C	2.04264200	3.66891500	-0.16408100
H	1.08543700	3.41197800	-2.07640900
H	3.10433100	3.63240300	1.70781700
H	1.70494600	4.69144000	-0.04397400
O	2.24357400	-2.41039900	1.70673900
H	2.22677000	-2.23673400	-0.76345800
H	3.01989100	-2.09430200	2.18436500
O	2.42340400	-3.53864600	-1.34533000
H	2.45964100	-4.24792000	-0.68945100
H	1.76928800	-3.80392500	-2.00542400

TS4
(PCM-THF:H_2O)-GD3BJ-B3LYP/6-311++g(d,p),
im. frequency = -47.02,
el. energy = -1099.122471 a.u.

C	0.04141800	-1.98869900	-0.31489900
C	-0.54041100	-2.93805900	-1.06270800
O	-0.49081600	-0.82986000	0.15770400
N	-1.88339400	-0.63438700	-0.20494800
C	-1.93447500	0.56172000	-1.10250800
C	-2.66550100	-0.64392600	1.06912900
C	-3.42391300	0.79234300	-1.42053500
C	-1.20291100	0.18555400	-2.39986800
C	-1.27841500	1.83081000	-0.52988300
C	-4.12908400	-0.36320900	0.67567500
C	-2.57872200	-2.06565600	1.64449300
C	-2.17507500	0.34525000	2.14479500
C	-4.29636600	0.89520000	-0.17216700
H	-3.77939100	-0.04105900	-2.03599000
H	-3.49856000	1.69729300	-2.02981800
H	-1.33423100	0.98577400	-3.13217400
H	-0.13319400	0.05684000	-2.22871100
H	-1.61002000	-0.73574100	-2.82074700
H	-0.29801600	1.60620500	-0.11423500
H	-1.13927000	2.55333700	-1.33701700
H	-1.87913700	2.31155300	0.23918600
H	-4.50510600	-1.22388800	0.11230000
H	-4.71604000	-0.30012400	1.59607900

H	-1.57051400	-2.29544600	1.99417100
H	-3.25597300	-2.15354000	2.49706600
H	-2.86746500	-2.80481100	0.89534300
H	-2.64217200	0.08925700	3.09880000
H	-1.09368200	0.27695200	2.26794600
H	-2.43130000	1.37817700	1.92030600
H	-4.03766000	1.78672900	0.40606500
H	-5.34492000	1.00977800	-0.46058500
H	-1.56850400	-2.84322300	-1.37471700
H	0.03230800	-3.80910700	-1.34642600
C	1.40857700	-2.10892500	0.14101600
C	3.34373400	0.37463100	-0.96852600
O	2.28129200	-0.50971800	-1.41660700
H	1.77963600	-1.36783500	0.84241600
H	4.04157900	-0.18625100	-0.33999800
H	3.88275800	0.74326300	-1.84377300
C	2.73132700	1.51403900	-0.20505400
C	2.33589900	2.67746700	-0.87131700
C	2.52244900	1.41787400	1.17357800
C	1.74019200	3.72500500	-0.17377700
H	2.49540800	2.76073500	-1.94082000
C	1.91462300	2.45861000	1.87299400
H	2.84723900	0.53122900	1.70805400
C	1.52383800	3.61496300	1.19923500
H	1.43868600	4.62249900	-0.70088700
H	1.75308500	2.36994000	2.94082900
H	1.05406900	4.42683500	1.74197800
O	2.11182500	-3.12688300	-0.12596400
H	2.63433800	-1.08264100	-2.10990000
H	3.05142400	-3.06931900	0.30535900
O	4.36870700	-2.82268500	0.93079200
H	5.13837300	-2.95827300	0.36289900
H	4.54885000	-3.29119800	1.75618400

2
(PCM-THF:H_2O)-GD3BJ-B3LYP/6-311++g(d,p),
el. energy = -675.308592 a.u.

C	2.12275600	-0.00005300	-0.25193900
C	2.21732300	-0.00006000	-1.58371000
O	0.99362500	-0.00002400	0.51858700
N	-0.24648600	-0.00000500	-0.23146300
C	-0.93504600	1.29364000	0.06174700
C	-0.93507600	-1.29362800	0.06176200
C	-2.28313000	1.24681500	-0.68343000
C	-0.07920800	2.41289800	-0.55037600
C	-1.13715300	1.60340700	1.55833500
C	-2.28316100	-1.24678200	-0.68341200
C	-0.07926200	-2.41290900	-0.55035200
C	-1.13718100	-1.60337700	1.55835500
C	-3.10452400	0.00002900	-0.36655200
H	-2.08282400	1.27571400	-1.75984300
H	-2.83547500	2.15725000	-0.43469500
H	-0.61628700	3.36154100	-0.47672100
H	0.87064200	2.52236200	-0.02413400
H	0.12674300	2.20984800	-1.60238500
H	-0.21083200	1.44659900	2.11199900
H	-1.42391600	2.65220600	1.66723800
H	-1.91917900	1.00070100	2.01525100
H	-2.08285700	-1.27570000	-1.75982500
H	-2.83552700	-2.15719900	-0.43466400

H	0.87059200	-2.52237700	-0.02411800
H	-0.61635500	-3.36154200	-0.47667800
H	0.12668100	-2.20987800	-1.60236700
H	-1.42396000	-2.65217100	1.66727000
H	-0.21085400	-1.44657700	2.11201000
H	-1.91919500	-1.00065500	2.01527000
H	-3.41722800	0.00004100	0.68157100
H	-4.02264800	0.00003700	-0.96068500
H	1.34184600	-0.00004400	-2.21365600
H	3.20383100	-0.00008200	-2.02594300
C	3.31268200	-0.00007600	0.63869400
O	4.46372000	0.00007400	0.25315900
H	3.06930900	0.00013500	1.71665600

Bn-OH
(PCM-THF:H_2O)-GD3BJ-B3LYP/6-311++g(d,p),
el. energy = -346.908455 a.u.

C	-1.94105800	0.00006800	0.52355500
O	-2.60144700	-0.00005800	-0.75853000
H	-2.23427700	0.88848600	1.09244300
H	-2.23428300	-0.88823300	1.09262200
C	-0.45520900	0.00003500	0.29224900
C	0.24352700	-1.20435400	0.16665200
C	0.24358500	1.20438800	0.16660900
C	1.61517200	-1.20681700	-0.08093700
H	-0.28981000	-2.14364100	0.26693100
C	1.61522600	1.20677800	-0.08097800
H	-0.28971400	2.14370100	0.26685000
C	2.30354100	-0.00003900	-0.20545800
H	2.14641300	-2.14715900	-0.17245800
H	2.14652300	2.14708500	-0.17253400
H	3.37083300	-0.00005800	-0.39418300
H	-3.55281100	-0.00006800	-0.60158000

H_3O^+
(PCM-THF:H_2O)-GD3BJ-B3LYP/6-311++g(d,p),
el. energy = -76.850990 a.u.

O	0.00000000	0.00000000	0.00000000
H	0.00000000	0.97068100	0.00000000
H	0.84063400	-0.48534000	0.00000000
H	-0.84063400	-0.48534000	0.00000000

1
(PCM-THF:H_2O)-GD3BJ-B3LYP/6-311++g(d,p),
el. energy = -462.394703 a.u.

C	2.85997800	-0.69085600	0.17933700
C	3.64057700	-1.70099600	-0.08845500
H	4.71326700	-1.57651600	-0.21767300
H	3.25096000	-2.71036700	-0.19673800
C	2.11193300	0.33905500	0.47754000
C	0.32524000	1.78052300	-0.10139000
O	1.58066600	1.17184000	-0.48117000
H	1.90507000	0.61022500	1.51072200
H	0.44580900	2.28200400	0.86428800
H	0.14943600	2.53489800	-0.86770200
C	-0.80111700	0.78163800	-0.04669200
C	-1.28925400	0.20990600	-1.22700400
C	-1.34890600	0.38690600	1.17566100
C	-2.30532100	-0.74038800	-1.18430200
H	-0.86912500	0.51173000	-2.18033700

C	-2.37040100	-0.56237400	1.22201400
H	-0.97685300	0.82446800	2.09575300
C	-2.84865300	-1.12843200	0.04219000
H	-2.67761200	-1.17551800	-2.10452600
H	-2.78908100	-0.85908000	2.17663100
H	-3.64165400	-1.86643900	0.07554700

TEMPO+

(PCM-THF:H_2O)-GD3BJ-B3LYP/6-311++g(d,p),

el. energy = -483.722979 a.u.

O	0.00003900	-1.94665400	-0.36838400
N	0.00002000	-0.78633000	-0.12300600
C	1.35362600	-0.07742000	0.02474800
C	-1.35363200	-0.07745100	0.02473300
C	1.24788900	1.30417700	-0.65391700
C	2.40699500	-0.95257400	-0.64551000
C	1.63952300	0.03257700	1.53884200
C	-1.24791700	1.30412800	-0.65398000
C	-2.40694500	-0.95268400	-0.64549500
C	-1.63955900	0.03265400	1.53881600
C	-0.00003800	2.09029300	-0.27252300
H	1.27119700	1.15749700	-1.73707900
H	2.15689500	1.84460500	-0.38221900
H	3.34631700	-0.39925000	-0.62081700
H	2.55023400	-1.89676700	-0.12172200
H	2.15318100	-1.15426200	-1.68694200
H	1.53022900	-0.93274200	2.03288700
H	2.68038900	0.34658600	1.62577100
H	1.02030100	0.77087300	2.03850600
H	-1.27116300	1.15742000	-1.73714000
H	-2.15696100	1.84452800	-0.38234400
H	-2.55018000	-1.89683600	-0.12162800
H	-3.34628300	-0.39938000	-0.62088800
H	-2.15308900	-1.15446900	-1.68689700
H	-2.68059500	0.34610400	1.62571500
H	-1.52974400	-0.93249000	2.03308500
H	-1.02073200	0.77141700	2.03829200
H	-0.00006400	2.34334900	0.78980600
H	-0.00004200	3.03915600	-0.81256900

VdW Adduct (polar)

(PCM-THF:H_2O)-GD3BJ-B3LYP/6-311++g(d,p),

el. energy = -946.127340 a.u.

C	-0.37043100	-1.60708300	1.75091300
C	0.51215500	-2.05151200	2.59663700
O	0.65333600	0.25225800	-0.35999900
N	1.84362400	0.31899500	-0.28156700
C	2.44411700	1.42175900	0.59479700
C	2.67394400	-0.69027900	-1.07883100
C	3.70952200	0.88178600	1.28686800
C	1.38314600	1.83088400	1.61363600
C	2.75622400	2.60420200	-0.34890900
C	3.91025600	-1.07928600	-0.24767700
C	1.78443300	-1.89871400	-1.36089800
C	3.05717800	0.00443800	-2.40351800
C	4.64322500	0.10930000	0.36269600
H	3.40400700	0.23911900	2.11688900
H	4.21423800	1.74605500	1.72313700
H	1.84489400	2.55252600	2.28867600
H	0.52251000	2.30211900	1.14102700

H	1.04567500	0.97620100	2.20033000
H	1.88473900	2.86760000	-0.94907600
H	3.00511300	3.45301400	0.28972000
H	3.60211200	2.41319600	-1.00350900
H	3.59435800	-1.76146600	0.54632400
H	4.56380400	-1.64631200	-0.91365400
H	0.96542100	-1.65361100	-2.03543400
H	2.40908900	-2.65453500	-1.83836600
H	1.37480200	-2.31746900	-0.44177800
H	3.48206300	-0.76777400	-3.04652400
H	2.17711900	0.41829200	-2.89649700
H	3.80109700	0.78572800	-2.27509700
H	5.05454700	0.75855400	-0.41360200
H	5.49544600	-0.25434000	0.94013900
H	1.58116300	-1.92172400	2.43680600
H	0.22249600	-2.57751500	3.50364500
C	-1.28255000	-1.13462700	0.93130500
C	-2.42568900	-1.13100400	-1.15802000
O	-1.80867900	-1.88442300	-0.07753600
H	-1.67926400	-0.13182200	1.05738600
H	-1.65260700	-0.56463500	-1.68081800
H	-2.80317000	-1.90558400	-1.82418500
C	-3.53078700	-0.22324500	-0.68865200
C	-4.70220500	-0.76195500	-0.14405100
C	-3.39357000	1.16434000	-0.77131300
C	-5.71663700	0.07455600	0.31115900
H	-4.81514600	-1.83841000	-0.07614600
C	-4.41128200	2.00516100	-0.32016600
H	-2.48655400	1.58805900	-1.18893200
C	-5.57283600	1.46101200	0.22317300
H	-6.62105600	-0.35127900	0.72968000
H	-4.29443600	3.08024700	-0.38977100
H	-6.36436100	2.11173700	0.57599300

TS (polar)
(PCM-THF:H_2O)-GD3BJ-B3LYP/6-311++g(d,p),
im. frequency = -376.43,
el. energy = -946.116252 a.u.

C	0.13694900	0.43531600	-0.98360500
C	-0.43899600	0.27816200	-2.14255600
O	-1.05245900	0.12608400	0.42909700
N	-2.26448600	-0.00665000	0.06478000
C	-3.13869500	1.21617900	0.22094700
C	-2.79190100	-1.42654100	-0.00170900
C	-4.42341900	1.02308600	-0.59956000
C	-2.37885400	2.44209500	-0.29456800
C	-3.44062500	1.41624300	1.72018300
C	-4.09793100	-1.44142100	-0.80856300
C	-1.75697700	-2.33891100	-0.66705600
C	-3.00916700	-1.91188100	1.44657400
C	-5.07739200	-0.34207300	-0.40859800
H	-4.18380600	1.15669900	-1.65958900
H	-5.10958800	1.82805300	-0.32560400
H	-3.02803800	3.31195900	-0.18216700
H	-1.46680300	2.62217500	0.27429300
H	-2.12554700	2.34066200	-1.34981000
H	-2.51347300	1.41339300	2.29550500
H	-3.92728500	2.38457900	1.85151400
H	-4.10357300	0.65081900	2.11824500
H	-3.85513700	-1.33217400	-1.87063100

H	-4.54629700	-2.43033100	-0.68619500
H	-0.80013000	-2.31143500	-0.14667500
H	-2.14061000	-3.35960600	-0.62479900
H	-1.60005900	-2.08545900	-1.71424400
H	-3.27531100	-2.97050900	1.42151600
H	-2.09043400	-1.79814500	2.02419100
H	-3.80909800	-1.37665200	1.95329100
H	-5.41136300	-0.47415100	0.62405900
H	-5.97252800	-0.40699700	-1.03154000
H	-1.51824900	0.10826600	-2.16894500
H	0.10155200	0.26522700	-3.08125900
C	1.27589200	0.81191100	-0.31455400
C	2.92042500	0.54307100	1.39969500
O	1.74692500	0.05674800	0.62598500
H	1.71007800	1.80052400	-0.45729600
H	2.79761500	1.61578700	1.54227800
H	2.81421600	0.02604000	2.34932200
C	4.18880100	0.19294100	0.68645800
C	4.67804500	-1.11711700	0.73105700
C	4.87623300	1.16111500	-0.05074000
C	5.83612600	-1.45587100	0.03811400
H	4.15142800	-1.86833800	1.30886900
C	6.03863700	0.82221900	-0.74102500
H	4.51260300	2.18211200	-0.07783700
C	6.51678400	-0.48620000	-0.69980000
H	6.21097700	-2.47158200	0.07697300
H	6.57014800	1.57868000	-1.30573100
H	7.42077900	-0.74965100	-1.23600700

INT2 (polar)
(PCM-THF:H_2O)-GD3BJ-B3LYP/6-311++g(d,p),
el. energy = -946.171057 a.u.

C	-0.09072400	-0.05600500	-0.66958500
C	-0.01418800	-0.03038700	-2.01870400
O	0.90807100	-0.04623400	0.23514100
N	2.23593400	0.00506100	-0.36977000
C	2.91833400	-1.27843700	-0.01692100
C	2.83975300	1.31422000	0.02940300
C	4.34314300	-1.17499600	-0.59567300
C	2.17234900	-2.40383700	-0.74949500
C	2.94799800	-1.61202600	1.48660500
C	4.26823500	1.31769100	-0.54963300
C	2.02782200	2.41784200	-0.66538500
C	2.84826400	1.59622300	1.54368700
C	5.08146300	0.08698400	-0.15758800
H	4.27333100	-1.18524600	-1.68850400
H	4.88838900	-2.07516100	-0.29958700
H	2.72515200	-3.33883500	-0.63553900
H	1.17255100	-2.55717400	-0.33861200
H	2.08669100	-2.18284200	-1.81488000
H	1.95806000	-1.49516400	1.92886900
H	3.25260900	-2.65396500	1.60945500
H	3.65089800	-0.99732400	2.04431200
H	4.19780100	1.36432300	-1.64146900
H	4.75842100	2.23738100	-0.21930800
H	1.02042600	2.49673500	-0.25211200
H	2.52344000	3.37973500	-0.51739200
H	1.95619800	2.22959300	-1.73807500
H	3.09047900	2.64942500	1.70378100
H	1.86644900	1.40552500	1.97832700

H	3.58551200	1.00495700	2.08195700
H	5.26542200	0.07250400	0.92034200
H	6.06366200	0.12546200	-0.63625900
H	0.94017800	0.00202100	-2.52122600
H	-0.92412000	-0.04090300	-2.60178400
C	-1.40182100	-0.09537500	-0.10023100
C	-2.93811600	-0.15622700	1.79683000
O	-1.55549500	-0.11627000	1.15059900
H	-2.26840400	-0.10505500	-0.75666000
H	-2.90752400	-1.09820000	2.33798700
H	-2.87855700	0.68026700	2.48760000
C	-4.05676800	-0.05629400	0.82543900
C	-4.52709700	1.20011600	0.42251100
C	-4.63385700	-1.21579900	0.29350300
C	-5.55987100	1.29399600	-0.50486900
H	-4.08407700	2.09865600	0.83706300
C	-5.66821500	-1.11926900	-0.63277700
H	-4.27354300	-2.18868100	0.60801500
C	-6.12942600	0.13473700	-1.03244400
H	-5.92389500	2.26690100	-0.81132800
H	-6.11546400	-2.01835200	-1.03841700
H	-6.93590300	0.20909100	-1.75212400

IX. References.

(1). Wang, J.; Yang, S. A Silver Triflate-Catalyzed Cascade of in Situ-Oxidation and Allylation of Arylbenzylamines. *Tetrahedron Lett.* **2016**, *57*, 3444–3448.

(2). Trost, B. M.; Xie, J. Palladium-Catalyzed Asymmetric Ring Expansion of Allenylcyclobutanols: An Asymmetric Wagner-Meerwein Shift. *J. Am. Chem. Soc.* **2006**, *128*, 6044–6045.

(3). Trost, B. M.; Xie, J. Wagner - Meerwein Shift : Control of Absolute Stereochemistry in the C - C Bond Migration Event. *J. Am. Chem. Soc.* **2008**, *130*, 6231–6242.

(4). Xiang, M.; Pfa, D. E.; Ortiz, E.; Brito, G. A.; Krische, M. J. Enantioselective Ruthenium-BINAP-Catalyzed Carbonyl Reductive Coupling of Alkoxyallenes: Convergent Construction of Syn-Sec,Tert - Diols via (Z) - σ -Allylmetal Intermediates. *J. Am. Chem. Soc.* **2021**, *143***,** 8849−8854.

(5). Deagostino, A.; Prandi, C.; Toppino, A.; Venturello, P. Palladium-Catalysed Heck Reaction on 1,2-Dien-1-Ols: A Stereoselective Synthesis of α-Arylated α,β-Unsaturated Aldehydes. *Tetrahedron* **2008**, *64*, 10344–10349.

(6). Ando, N.; Terashima, S. A Novel Synthesis of the 2-Aminoimidazol-4-Carbaldehyde Derivatives, Versatile Synthetic Intermediates for 2-Aminoimidazole Alkaloids. *Synlett* **2006**, No. 17, 2836–2840.

(7). (a) Grimme, S.; Bannwarth, C.; Dohm, S.; Hansen, A.; Pisarek, J.; Pracht, P.; Seibert, J.; Neese, F. Fully Automated Quantum-Chemistry-Based Computation of Spin–Spin-Coupled Nuclear Magnetic Resonance Spectra. *Angew. Chemie - Int. Ed.* **2017**, *56*, 14763–14769. (b) Bannwarth, C.; Ehlert, S.; Grimme, S. GFN2-XTB - An Accurate and Broadly Parametrized Self-Consistent Tight-Binding Quantum Chemical Method with Multipole Electrostatics and Density-Dependent Dispersion Contributions. *J. Chem. Theory Comput.* **2019**, *15*, 1652–1671. (c) Pracht, P.; Bohle, F.; Grimme, S. Automated Exploration of the Low-Energy Chemical Space with Fast Quantum Chemical Methods. *Phys. Chem. Chem. Phys.* **2020**, *22*, 7169–7192.

(8). (a) M. J. Frisch, G. W. Trucks, H. B. Schlegel, G. E. Scuseria, M. A. Robb, J. R. Cheeseman, G. Scalmani, V. Barone, G. A. Petersson, H. Nakatsuji, X. Li, M. Caricato, A. V. Marenich, J. Bloino, B. G. Janesko, R. Gomperts, B. Mennucci, H. P. Hratchian, J. V. Ortiz, A. F. Izmaylov, J. L. Sonnenberg, D. Williams-Young, F. Ding, F. Lipparini, F. Egidi, J. Goings, B. Peng, A. Petrone, T. Henderson, D. Ranasinghe, V. G. Zakrzewski, J. Gao, N. Rega, G. Zheng, W. Liang, M. Hada, M. Ehara, K. Toyota, R. Fukuda, J. Hasegawa, M. Ishida, T. Nakajima, Y. Honda, O. Kitao, H. Nakai, T. Vreven, K. Throssell, J. A. Montgomery, Jr., J. E. Peralta, F. Ogliaro, M. J. Bearpark, J. J. Heyd, E. N. Brothers, K. N. Kudin, V. N. Staroverov, T. A. Keith, R. Kobayashi, J. Normand, K. Raghavachari, A. P. Rendell, J. C. Burant, S. S. Iyengar, J. Tomasi, M. Cossi, J. M. Millam, M. Klene, C. Adamo, R. Cammi, J. W. Ochterski, R. L. Martin, K. Morokuma, O. Farkas, J. B. Foresman, D. J. Fox, Gaussian16, Revision C.01; Gaussian, Inc., Wallingford, CT, (2019). (b) Becke, A. D. Thermochemistry. III. The Role of Exact Exchange. *J. Chem. Phys.* **1993**, *98*, 5648–5652. (c) Lee, C.; Yang, W.; Parr, R. G. Development of the Colle-Salvetti correlation-energy formula into a functional of the electron density. *Phys. Rev. B* **1988**, *37*, 785-789. (d) Clark, T.; Chandrasekhar, J.; Spitznagel, G. W.; Schleyer, P. V. R. Efficient Diffuse Function-augmented Basis Sets for Anion Calculations. III. The 3-21+G Basis Set for First-row Elements, Li–F. *J. Comput. Chem.* **1983**, *4*, 294–301. (e) Krishnan, R.; Binkley, J. S.; Pople, J. A. Self-consistent molecular orbital methods. XX. A basis set for correlated wave functions. *J. Chem. Phys*. **1980**, *72*, 650-654. (f) Wadt, W. R.; Hay, P. J. Ab initio effective core potentials for molecular calculations. Potentials for main group elements Na to Bi. *J. Chem. Phys*. **1985**, *82*, 284-298.

(9). Grimme, S.; Ehrlich, S.; Goerigk, L. Effect of the Damping Function in Dispersion Corrected Density Functional Theory. *J. Comp. Chem*. **2011**, *32*, 1456-65.

(10). (a) Tomasi, J.; Mennucci, B.; Cance, E. The IEF Version of the PCM Solvation Method: An Overview of a New Method Addressed to Study Molecular Solutes at the QM Ab Initio Level. *J. Mol. Struct.* **2018**, *464*, 211–226. (b) Mennucci, B.; Tomasi, J. Continuum Solvation Models: A New Approach to the Problem of Solute's Charge Distribution and Cavity Boundaries. *J. Chem. Phys.* **1997**, *106*, 5151–5158. (c) Mennucci, B.; Cancès, E.; Tomasi, J. Evaluation of Solvent Effects in Isotropic and Anisotropic Dielectrics and in Ionic Solutions with a Unified Integral Equation Method: Theoretical Bases, Computational Implementation, and Numerical Applications. *J. Phys. Chem. B* **1997**, *101*, 10506–10517.

(11). (a) Weast, R. C. *CRC Handbook of Chemistry and Physics, 76th Ed*, FL, (1995). (b) Winget, P.; Dolney, D. M.; Giesen D. J.; Cramer C. J.; Truhlar D. G. "Minnesota Solvent Descriptor Database" (2021).

(12). Chai, J.; Head-Gordon, M. Long-Range Corrected Hybrid Density Functionals with Damped Atom-Atom Dispersion Corrections. *Phys. Chem. Chem. Phys.* **2008**, *10*, 6615–6620.

(13). (a) Weigend, F.; Ahlrichs, R. Balanced Basis Sets of Split Valence, Triple Zeta Valence and Quadruple Zeta Valence Quality for H to Rn: Design and Assessment of Accuracy. *Phys. Chem. Chem. Phys.* **2005**, 3297–3305. (b) Weigend, F. Accurate Coulomb-Fitting Basis Sets for H to Rn. *Phys. Chem. Chem. Phys.* **2006**, *8*, 1057–1065. (c) Rappoport, D.; Furche, F. Property-Optimized Gaussian Basis Sets for Molecular Response Calculations. *J. Chem. Phys.* **2010**, *133*. (d) Peterson, K. A.; Figgen, D.; Goll, E.; Stoll, H.; Dolg, M. Systematically Convergent Basis Sets with Relativistic Pseudopotentials. II. Small-Core Pseudopotentials and Correlation Consistent Basis Sets for the Post-d Group 16-18 Elements. *J. Chem. Phys.* **2003**, *119*, 11113–11123.

(14). (a) Gonzalez, C.; Schlegel, H. B. Reaction Path Following in Mass-Weighted Internal Coordinates. *J. Phys. Chem.* **1990**, *94*, 5523–5527. (b) Gonzalez, C.; Bernhard Schlegel,

H. An Improved Algorithm for Reaction Path Following. *J. Chem. Phys.* **1989**, *90*, 2154–2161.

(15). (a) Tao, X.; Kehr, G.; Wang, X.; Daniliuc, C. G.; Grimme, S.; Erker, G. Rapid Dihydrogen Cleavage by Persistent Nitroxide Radicals under Frustrated Lewis Pair Conditions. *Chem. - A Eur. J.* **2016**, *22*, 9504–9507. (b) Pinto, A.; Kaiser, D.; Maryasin, B.; Di Mauro, G.; González, L.; Maulide, N. Hydrative Aminoxylation of Ynamides: One Reaction, Two Mechanisms. *Chem. - A Eur. J.* **2018**, *24*, 2515–2519.

(16). Kitagawa, Y.; Saito, T.; Ito, M.; Shoji, M.; Koizumi, K.; Yamanaka, S.; Kawakami, T.; Okumura, M.; Yamaguchi, K. Approximately Spin-Projected Geometry Optimization Method and Its Application to Di-Chromium Systems. *Chem. Phys. Lett.* **2007**, *442*, 445–450.

X. NMR Spectra.

$^{1}H\{^{13}C\}$ 500 MHz NMR for Compound 3c.

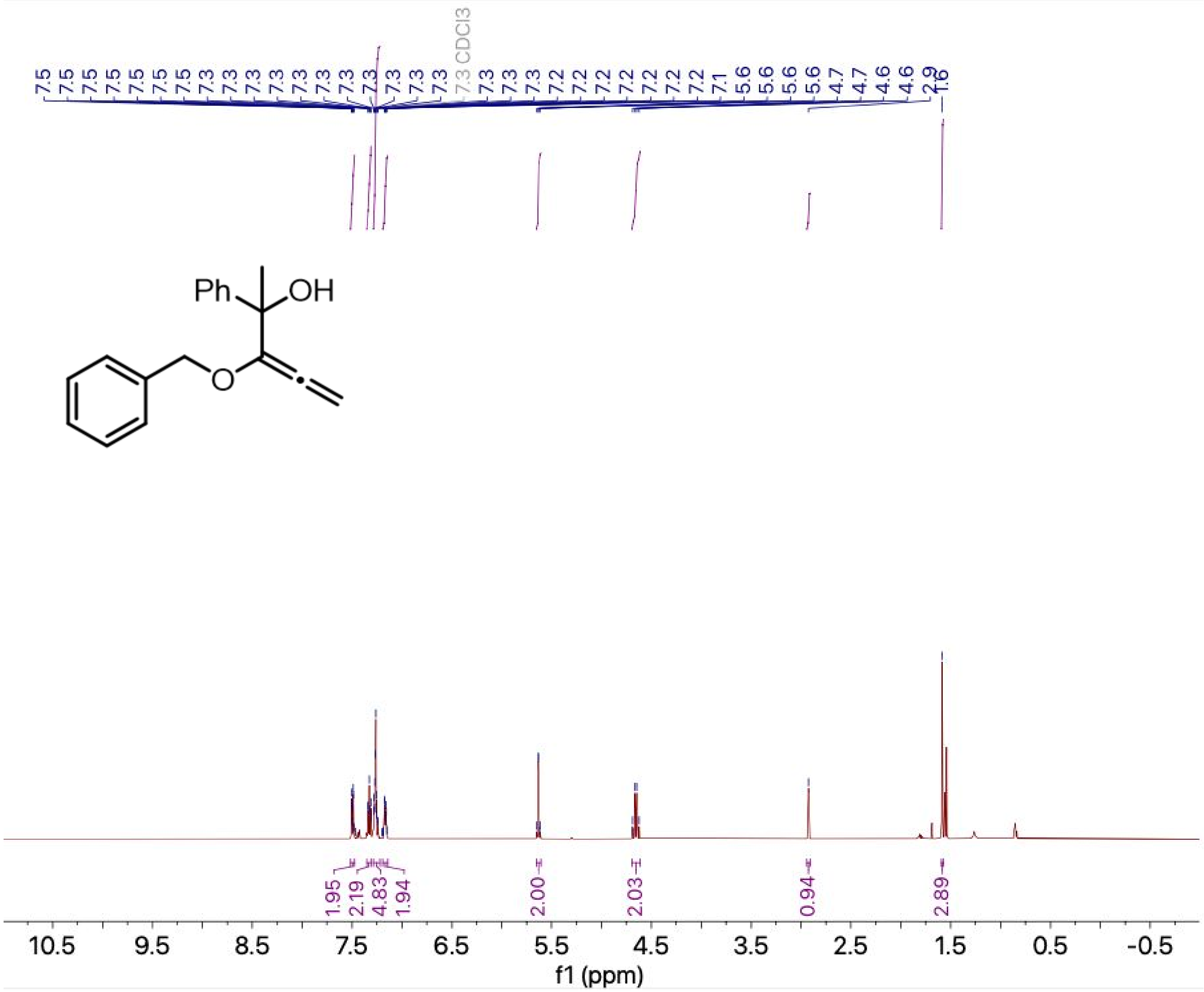

$^{13}C\{^{1}H\}$ 500 MHz NMR for Compound 3c.

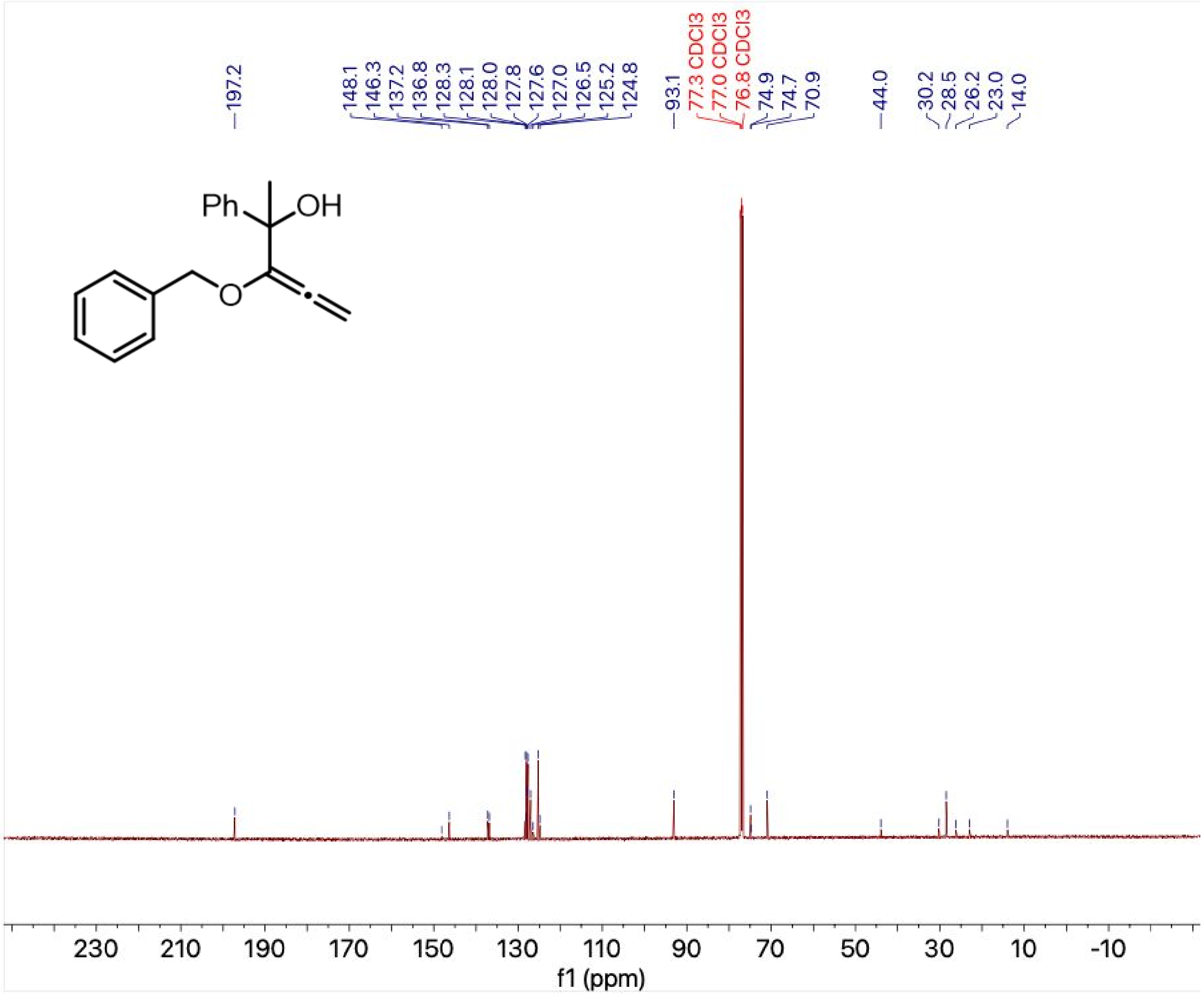

$^{1}H\{^{13}C\}$ **500 MHz NMR for Compound 3d.**

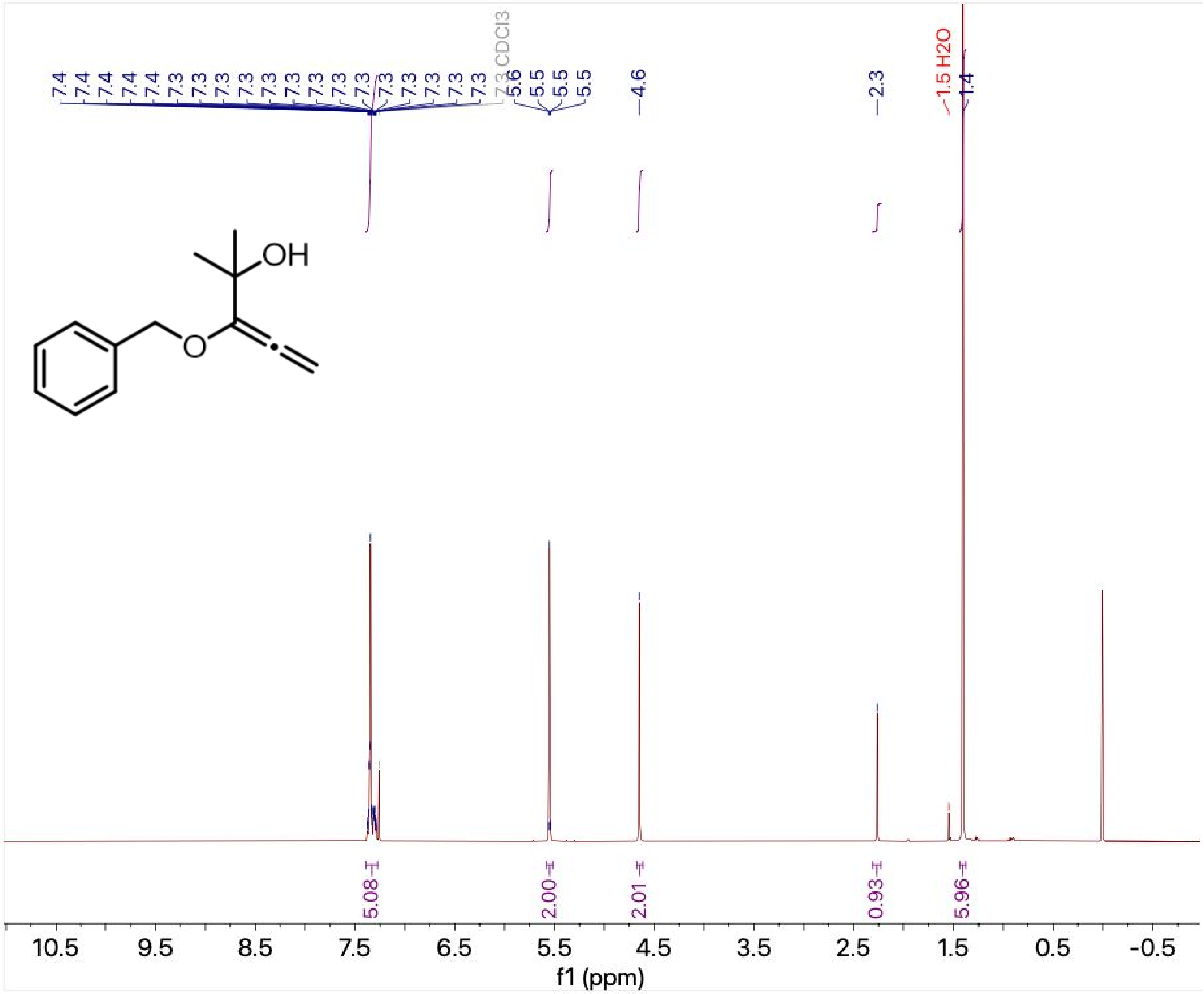

$^{13}C\{^1H\}$ 500 MHz NMR for Compound 3d.

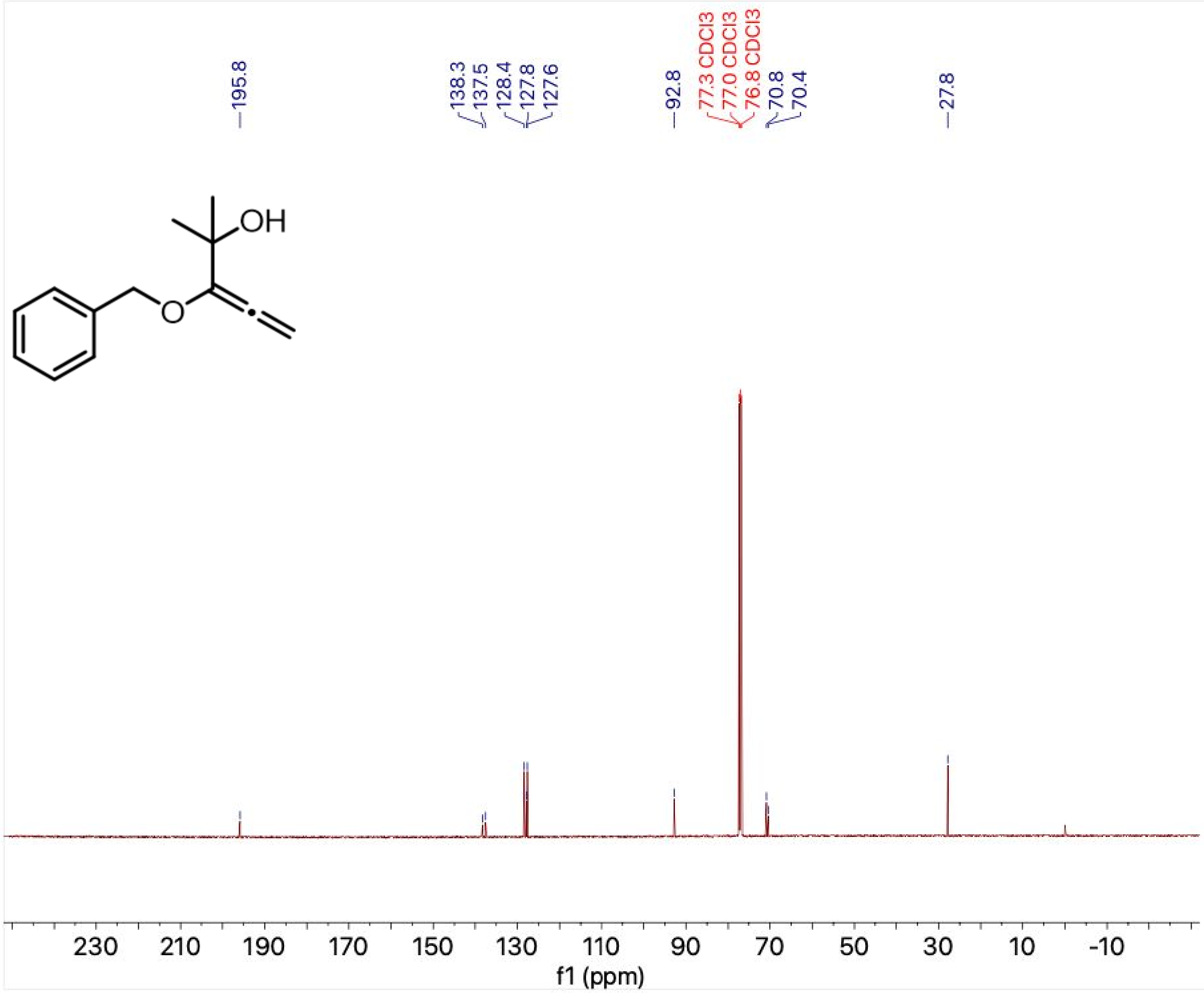

$^{1}H\{^{13}C\}$ 500 MHz NMR for Compound 3f.

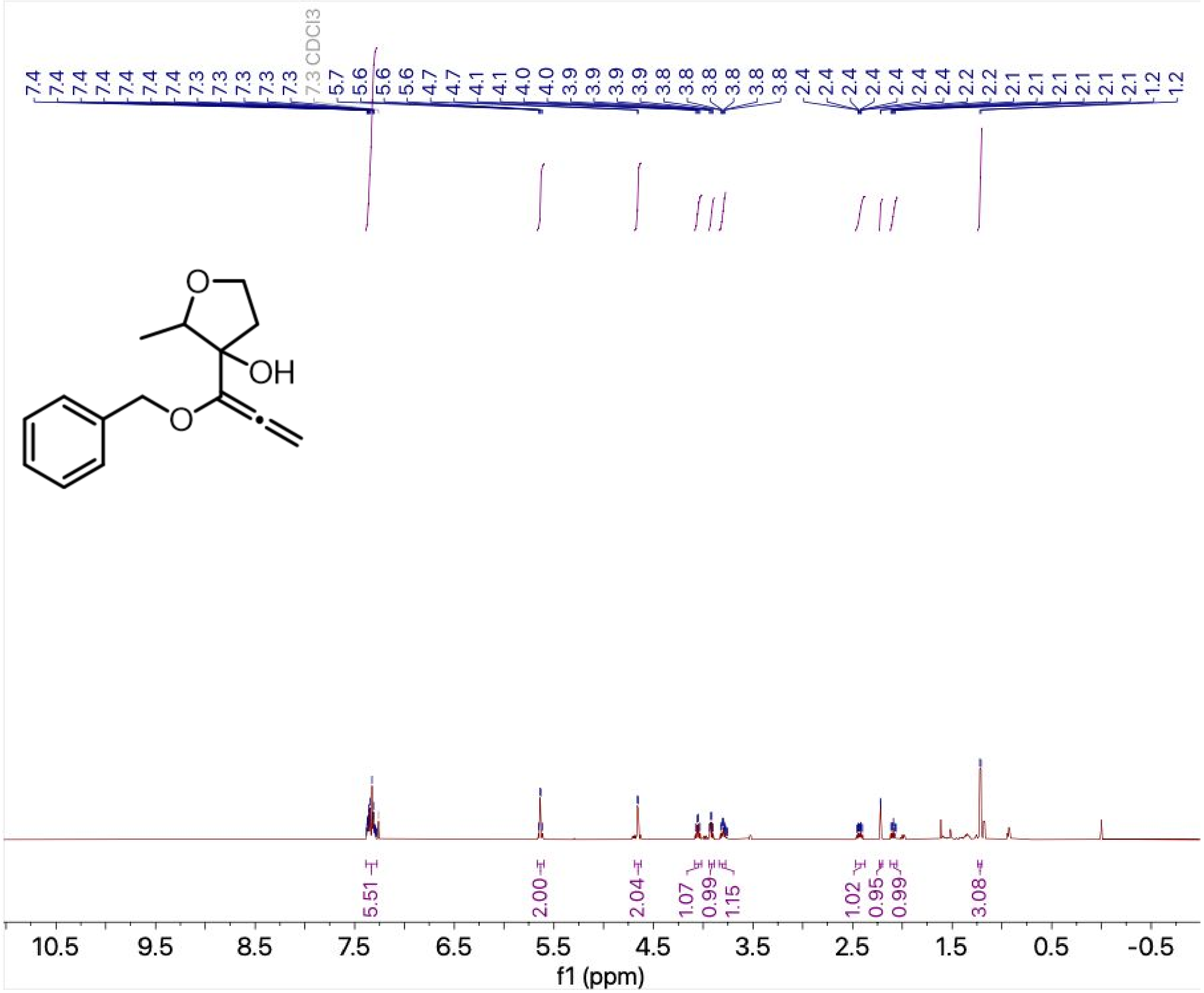

$^{13}C\{^1H\}$ **500 MHz NMR for Compound 3f.**

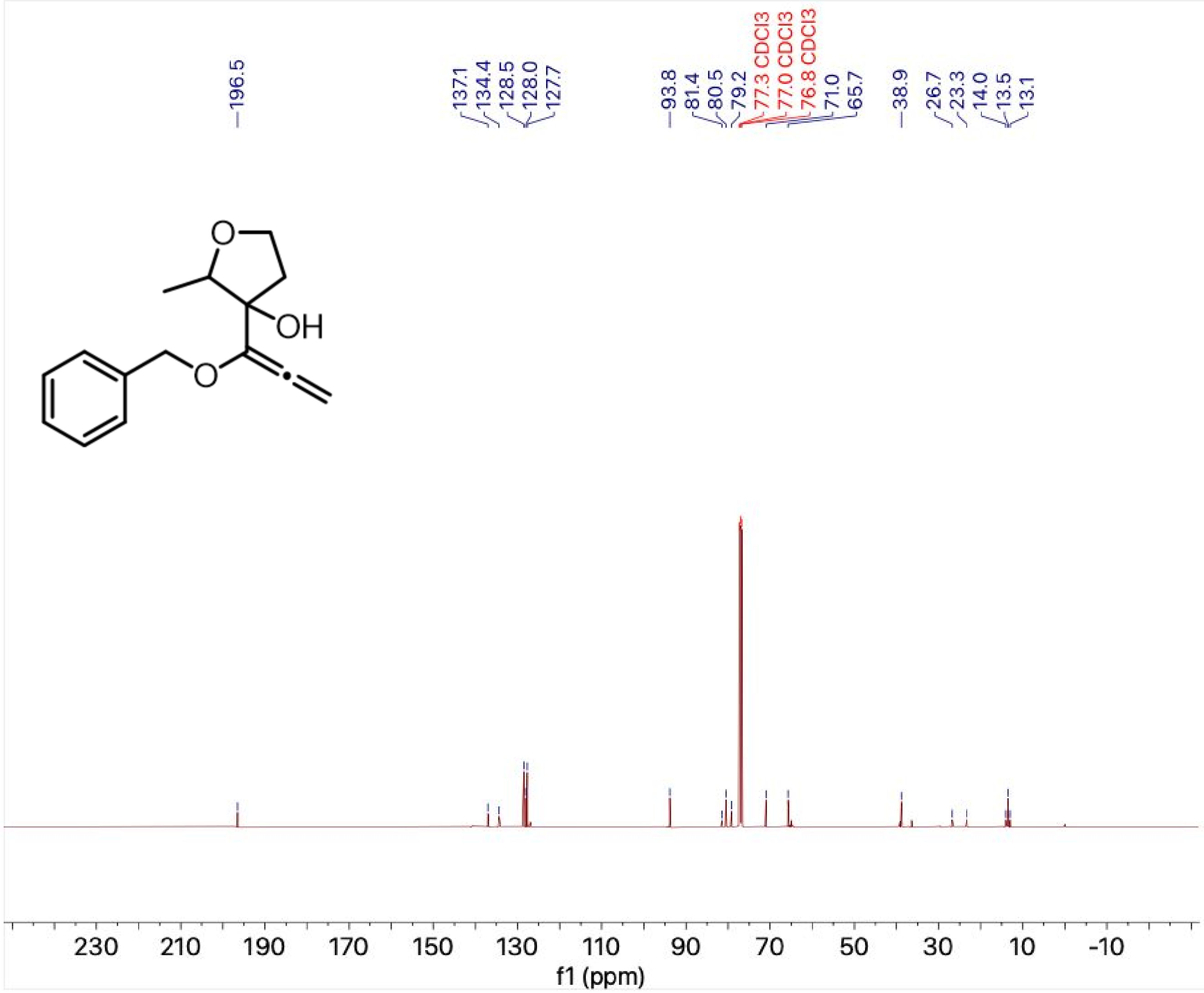

$^{1}H\{^{13}C\}$ 500 MHz NMR for Compound 3g.

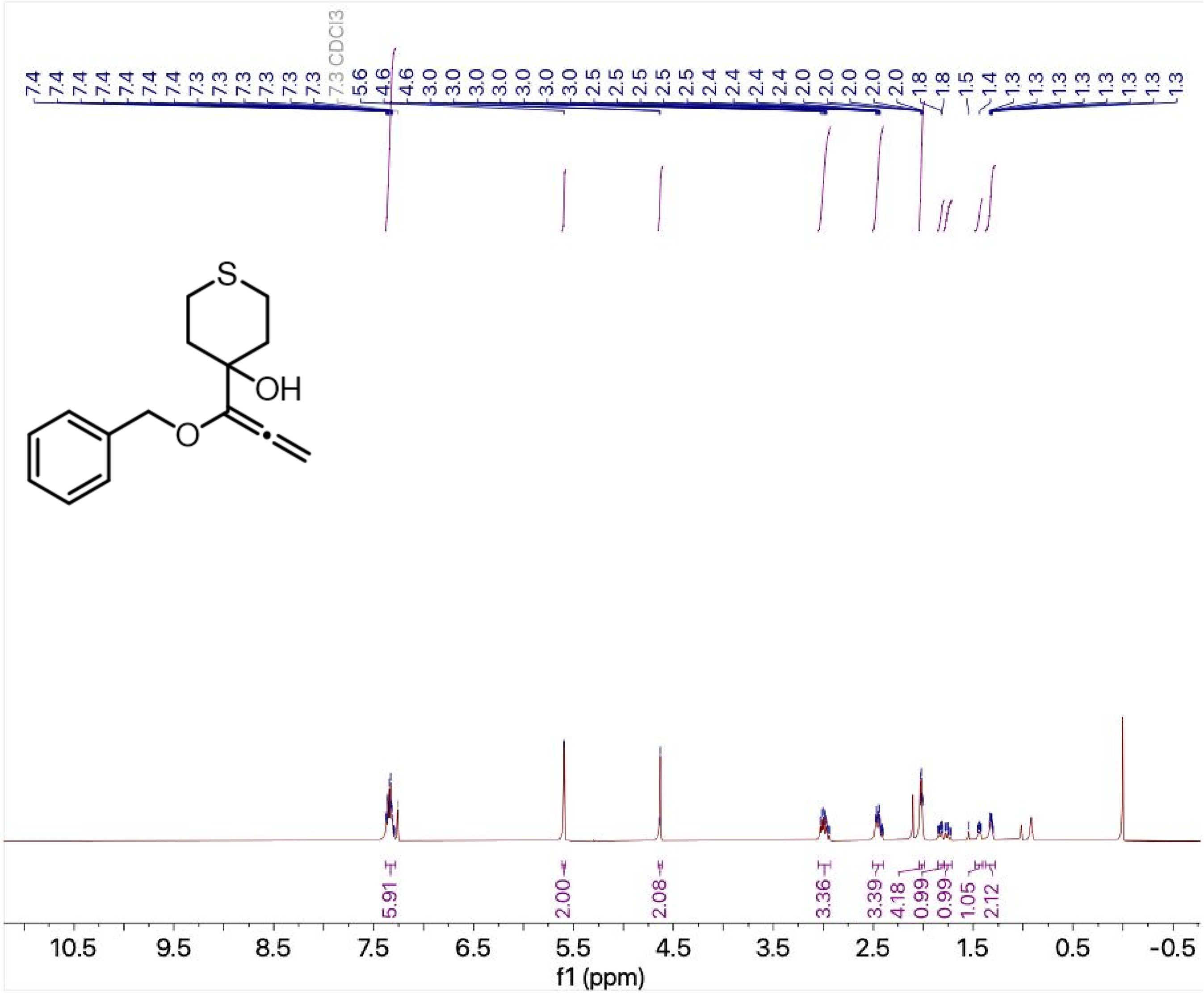

$^{13}C\{^{1}H\}$ **500 MHz NMR for Compound 3g.**

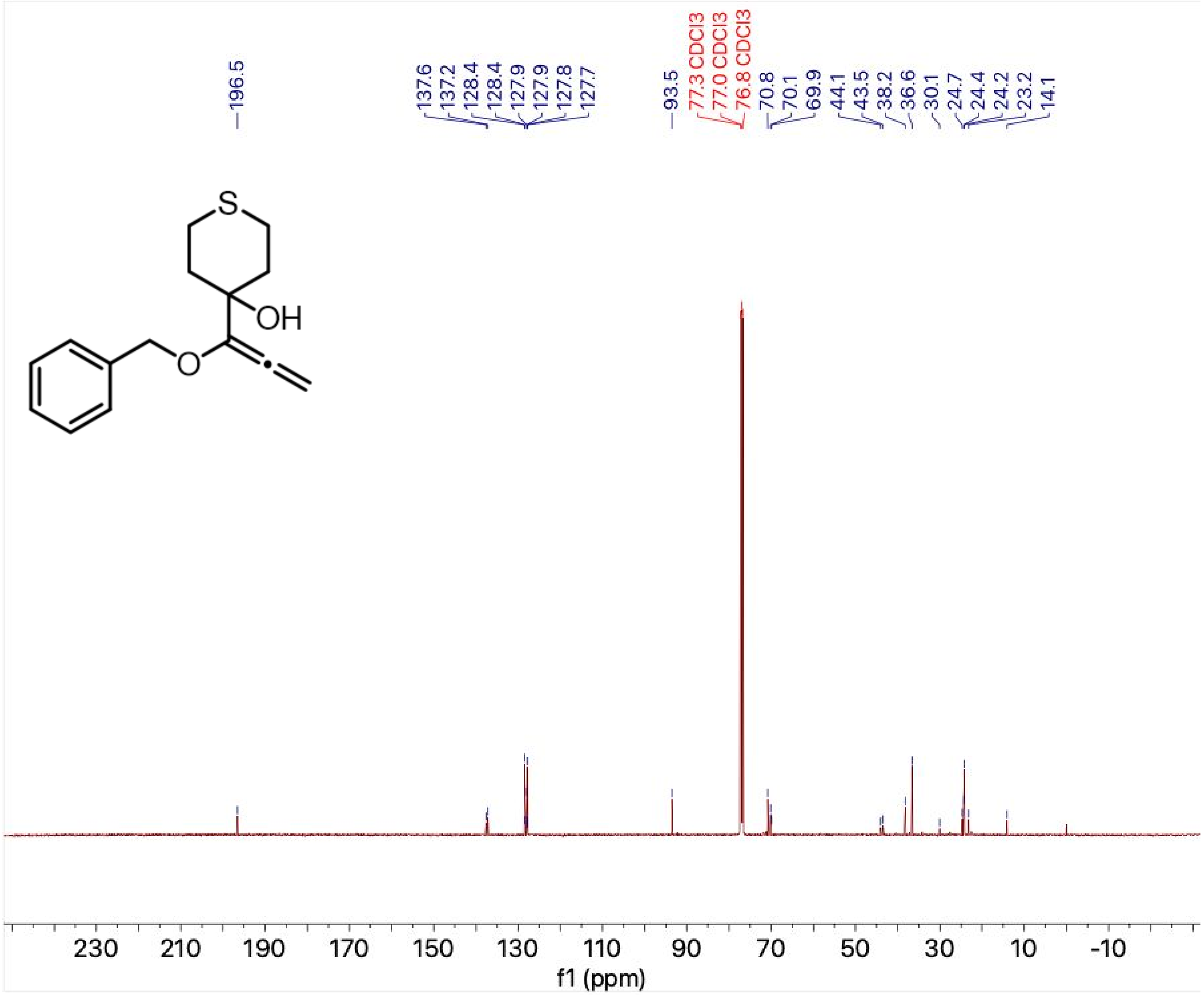

$^{1}H\{^{13}C\}$ **500 MHz NMR for Compound 3h.**

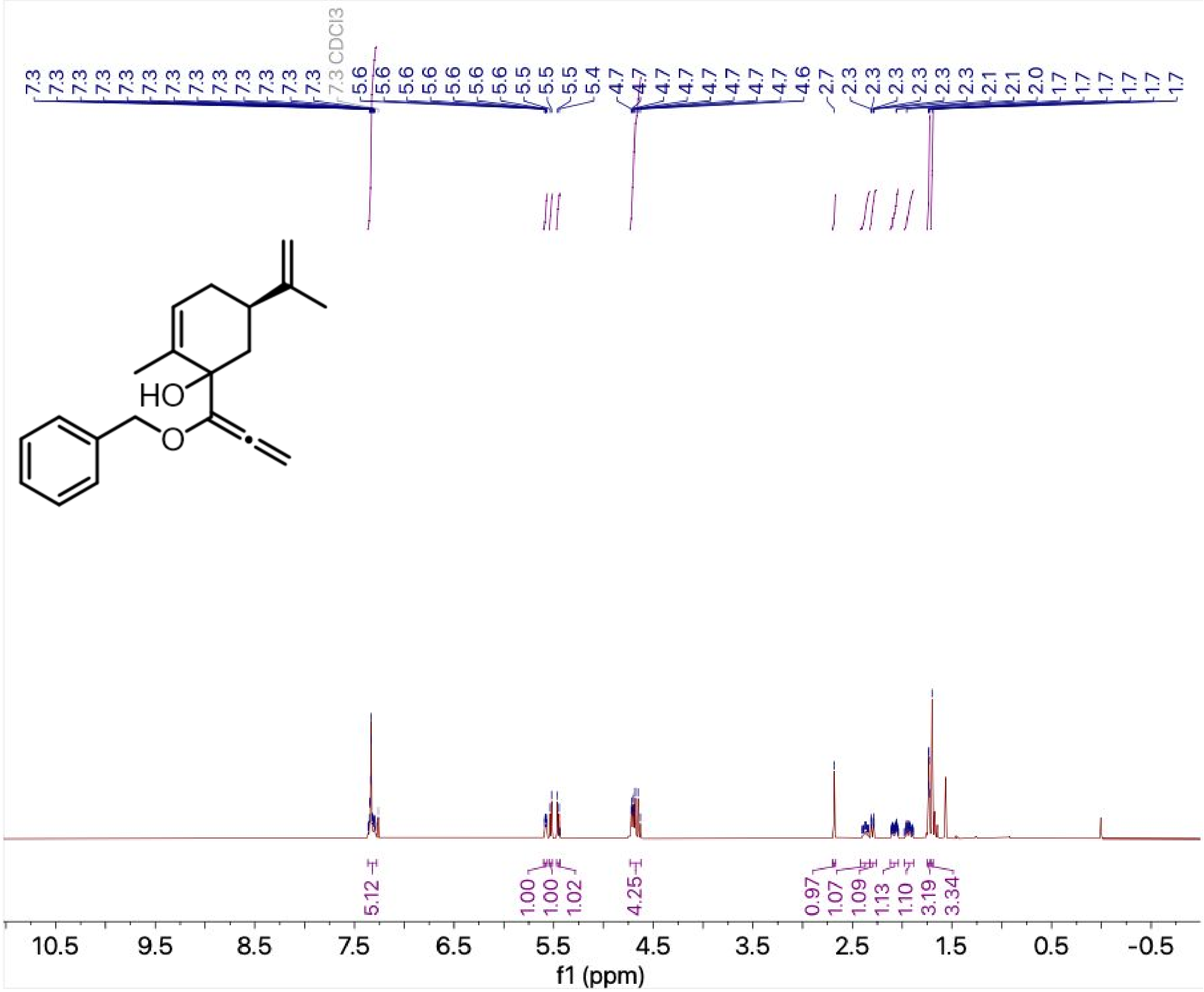

$^{13}C\{^{1}H\}$ 500 MHz NMR for Compound 3h.

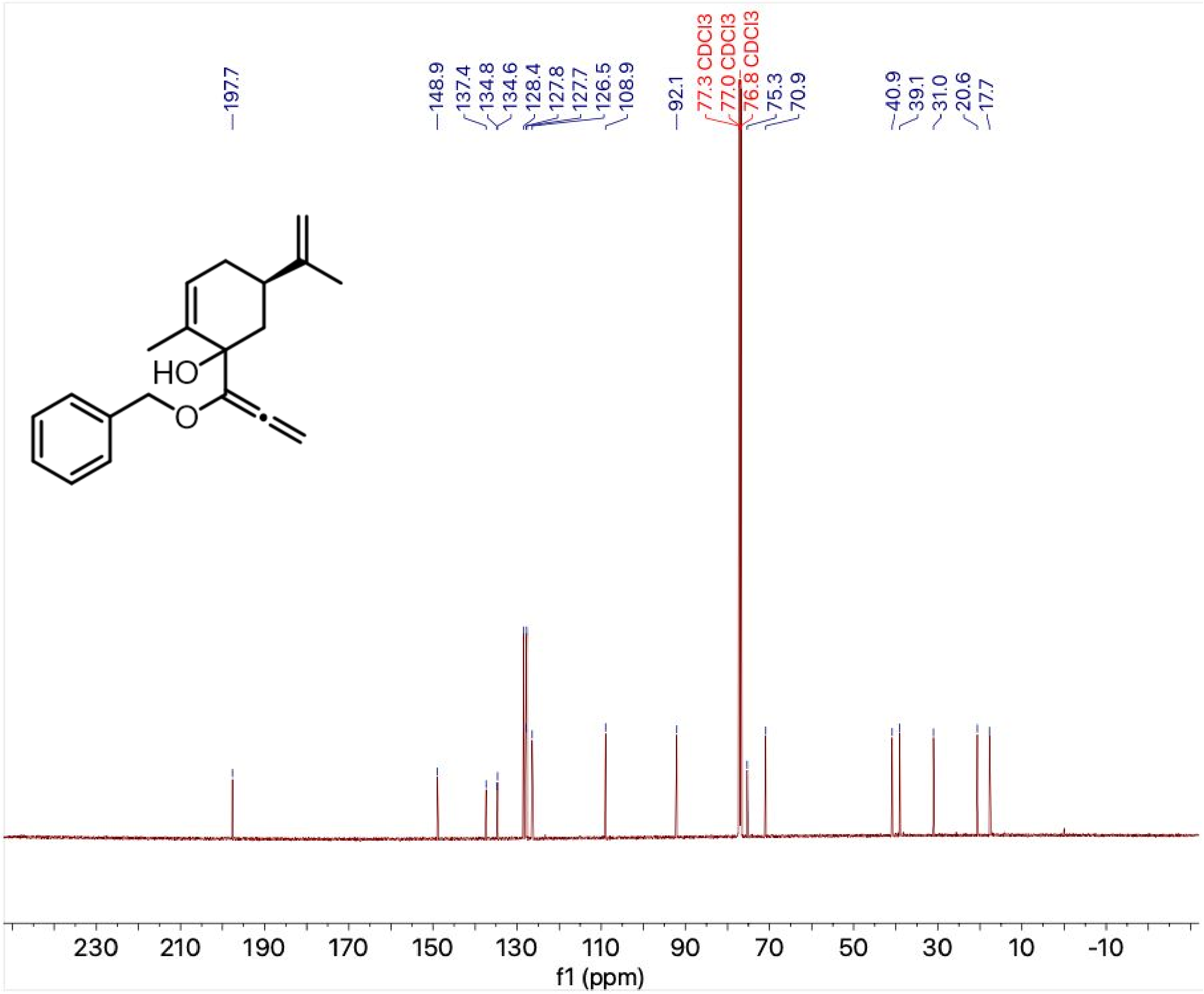

$^{1}H\{^{13}C\}$ 500 MHz NMR for Compound 3i.

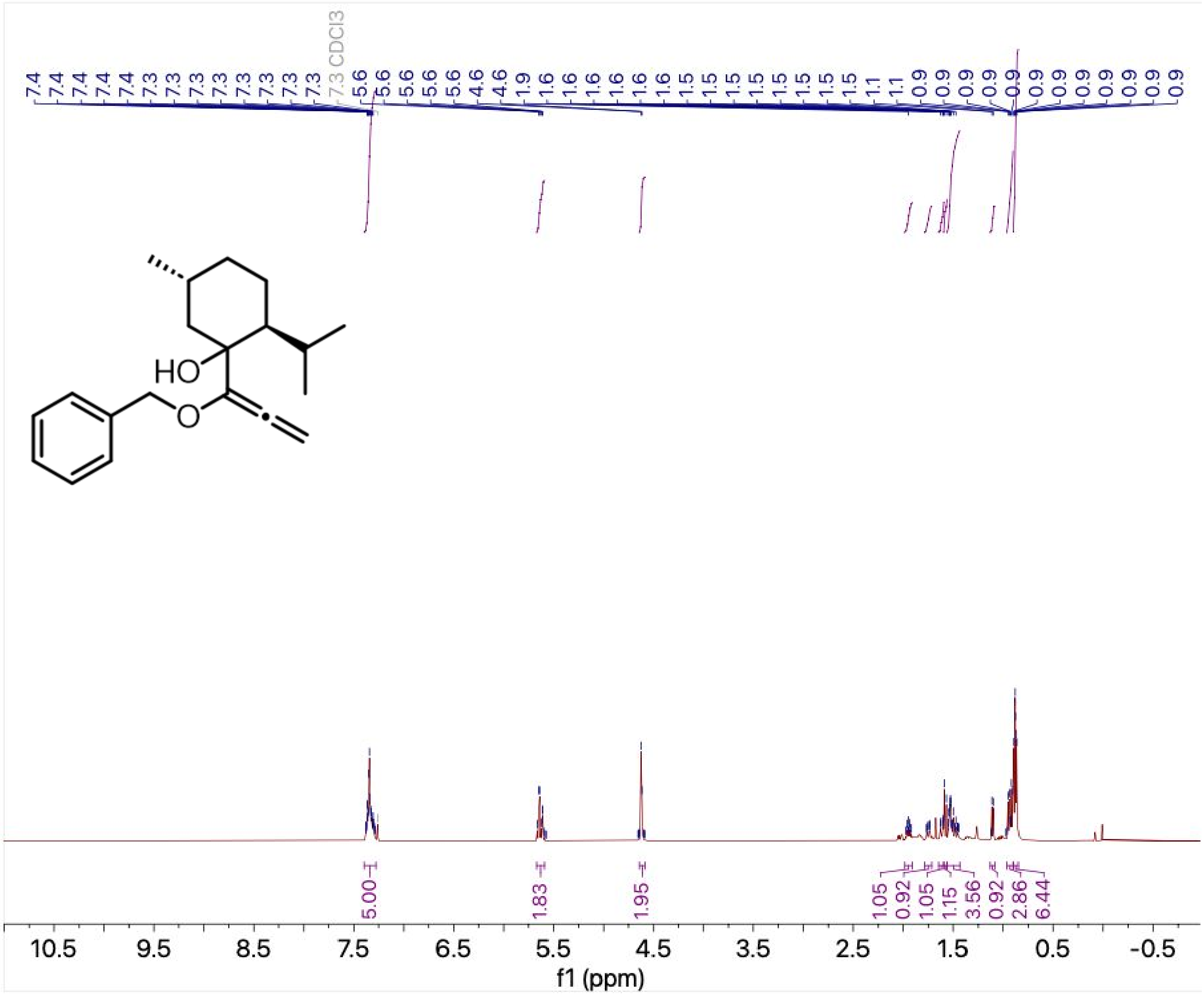

$^{1}H\{^{13}C\}$ **500 MHz NMR for Compound 3i.**

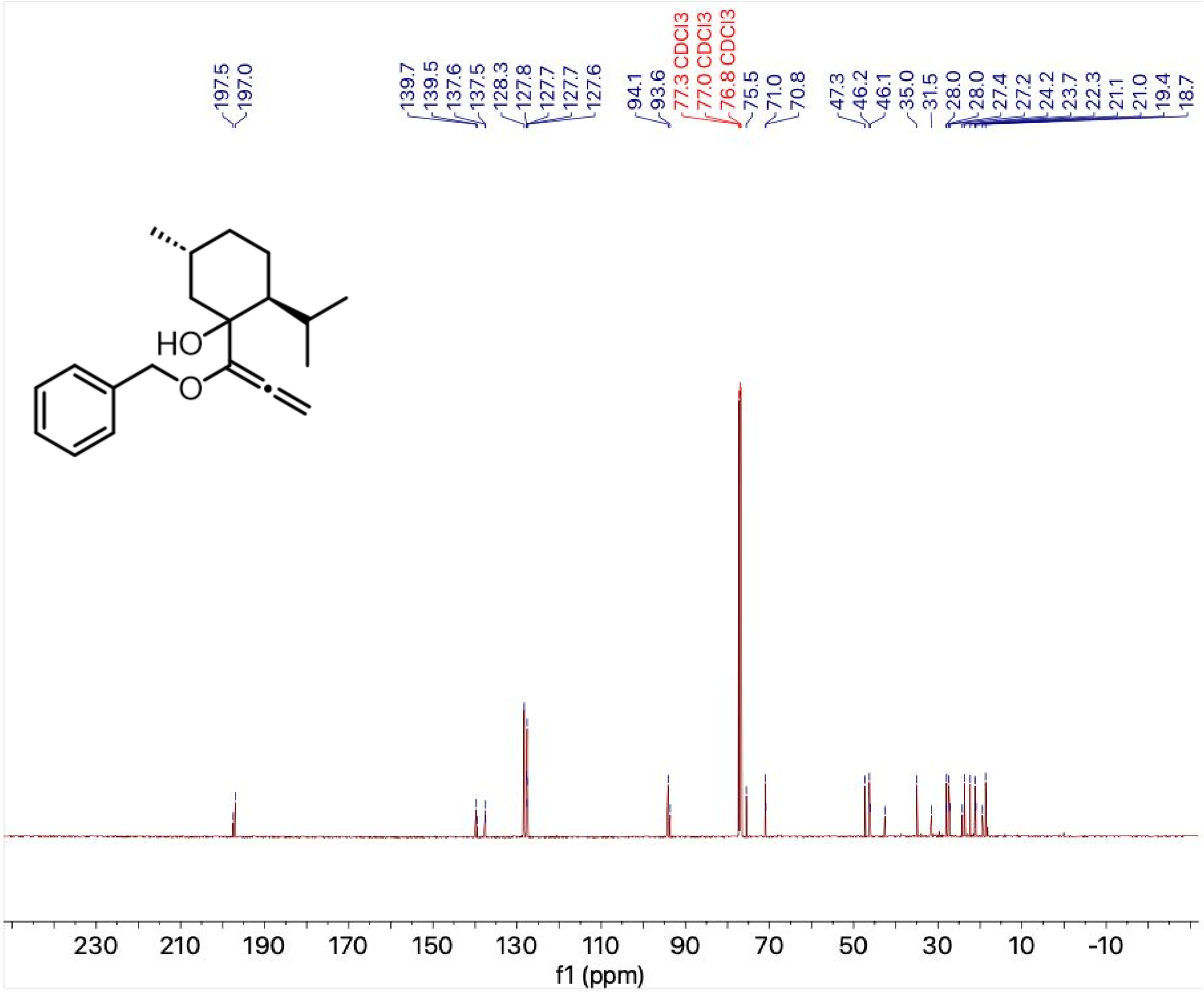

$^{1}H\{^{13}C\}$ 500 MHz NMR for Compound 3j.

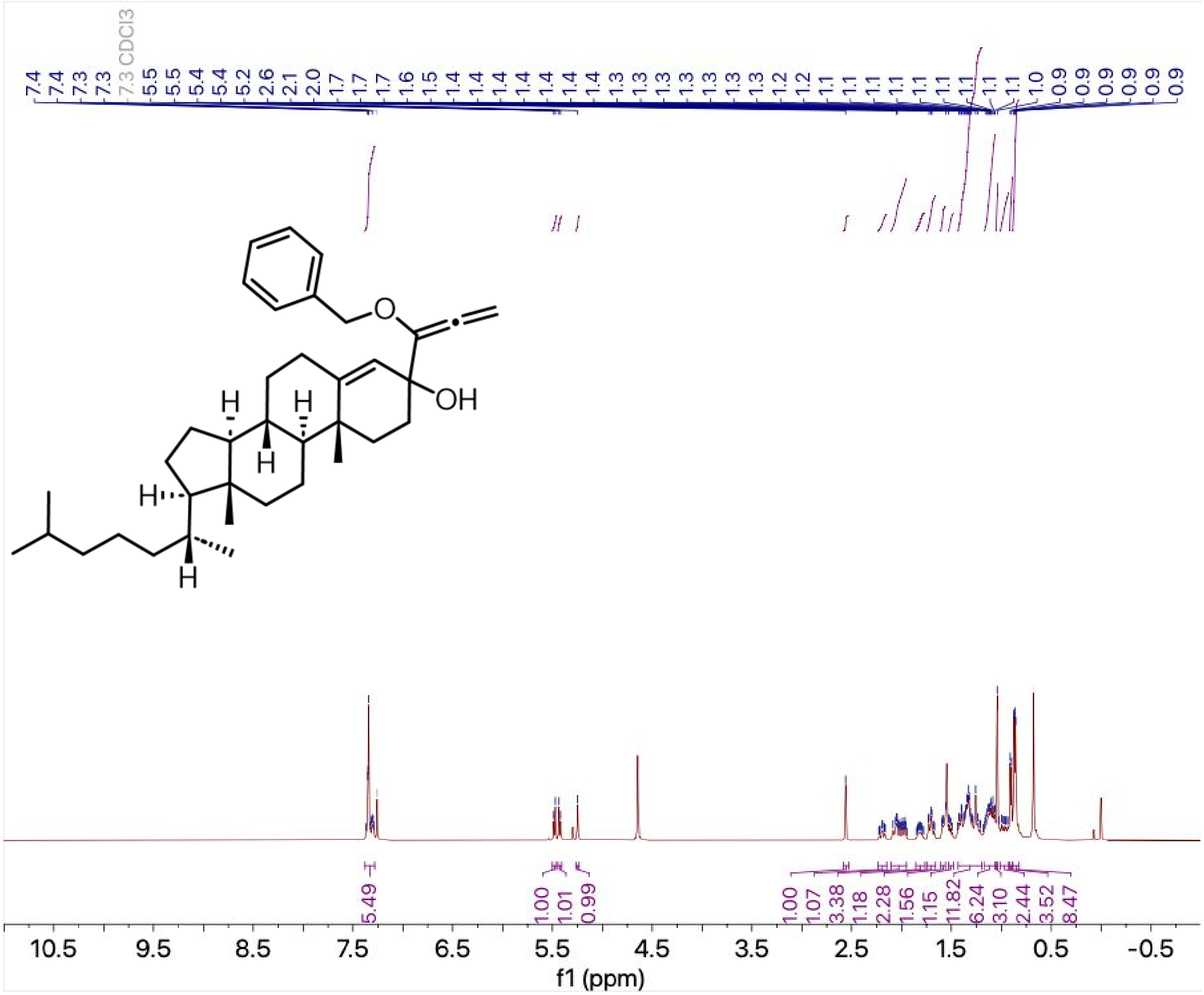

$^{13}C\{^{1}H\}$ 500 MHz NMR for Compound 3j.

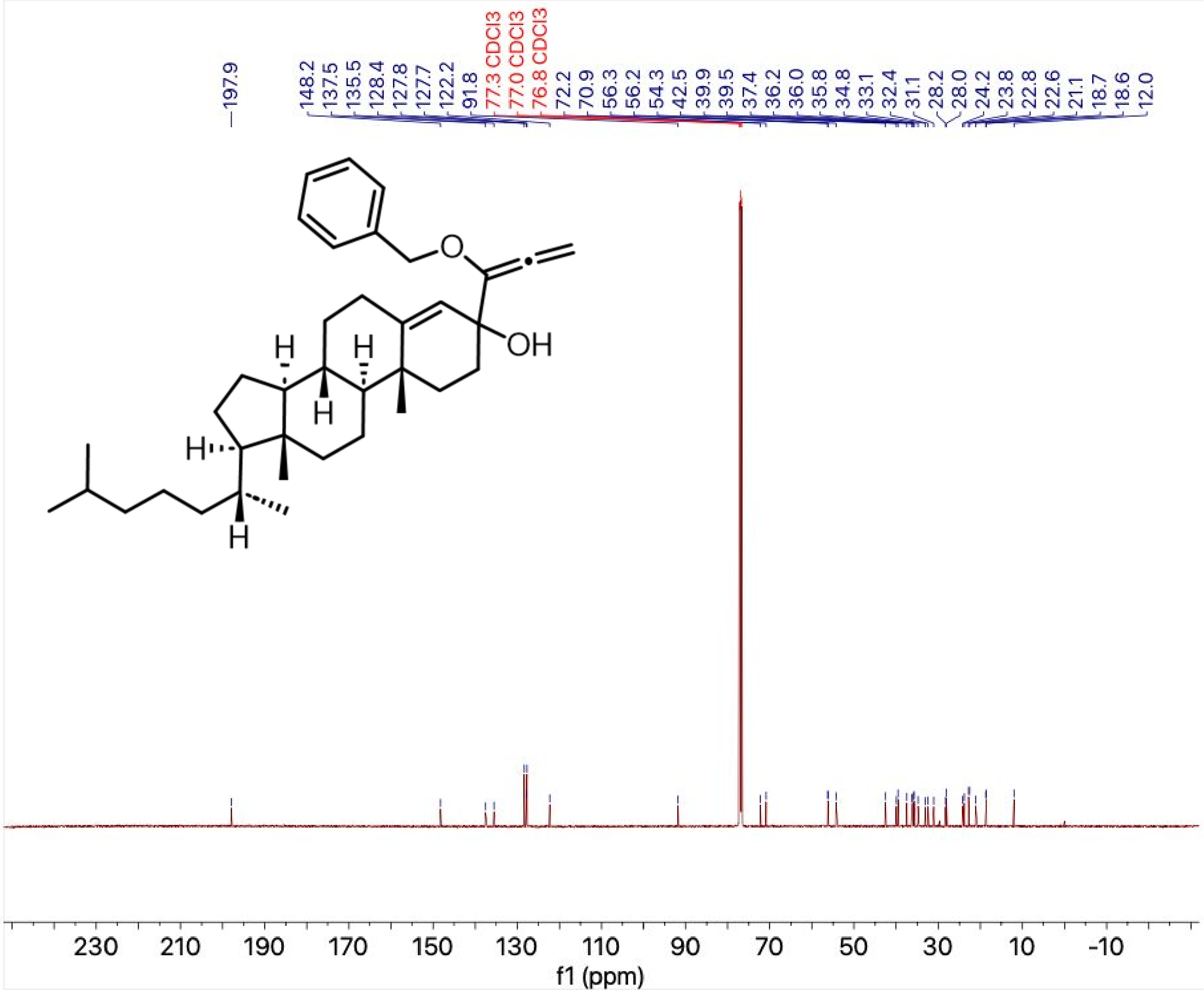

$^{1}H\{^{13}C\}$ 500 MHz NMR for Compound 3k.

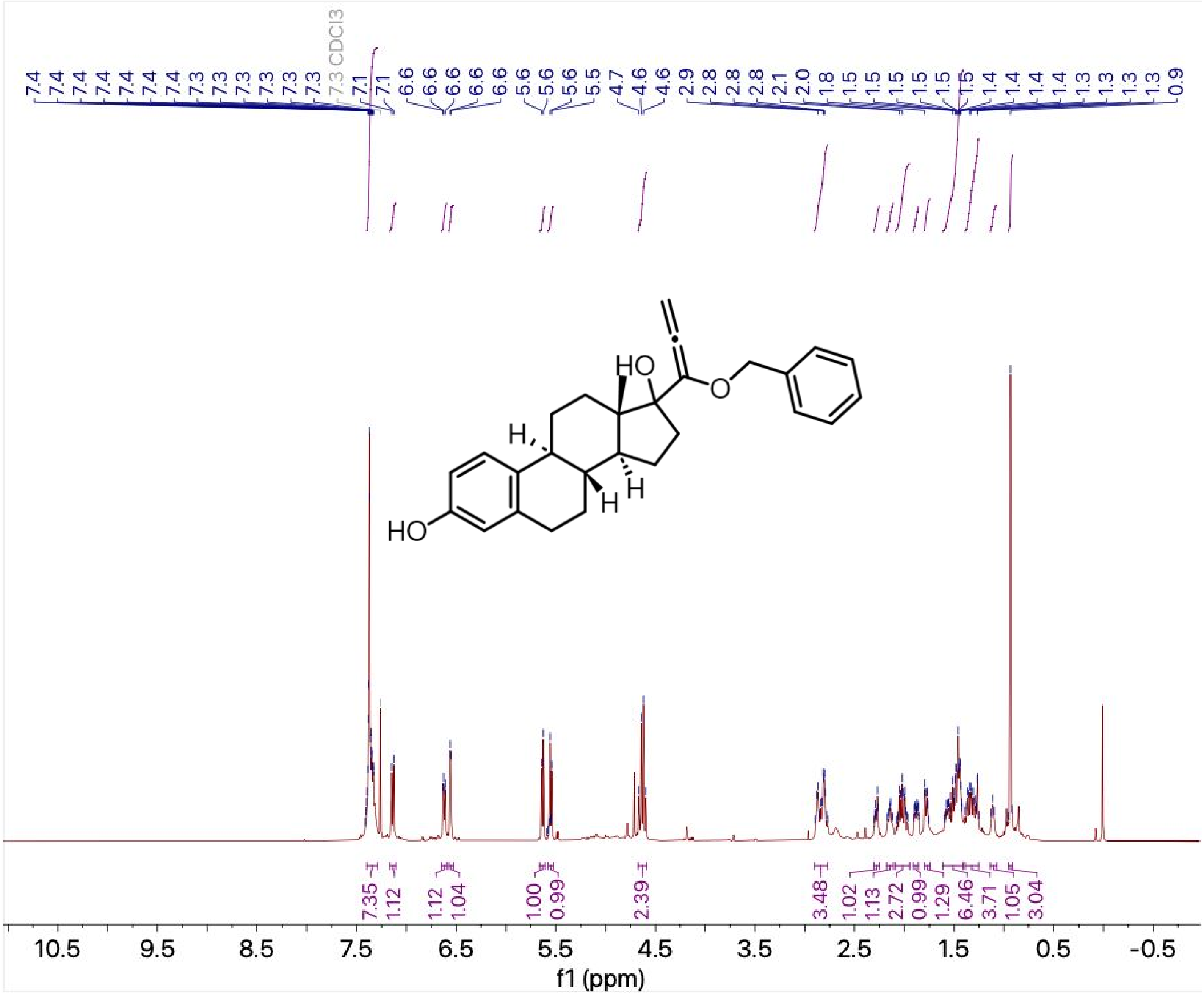

$^{13}C\{^1H\}$ 500 MHz NMR for Compound 3k.

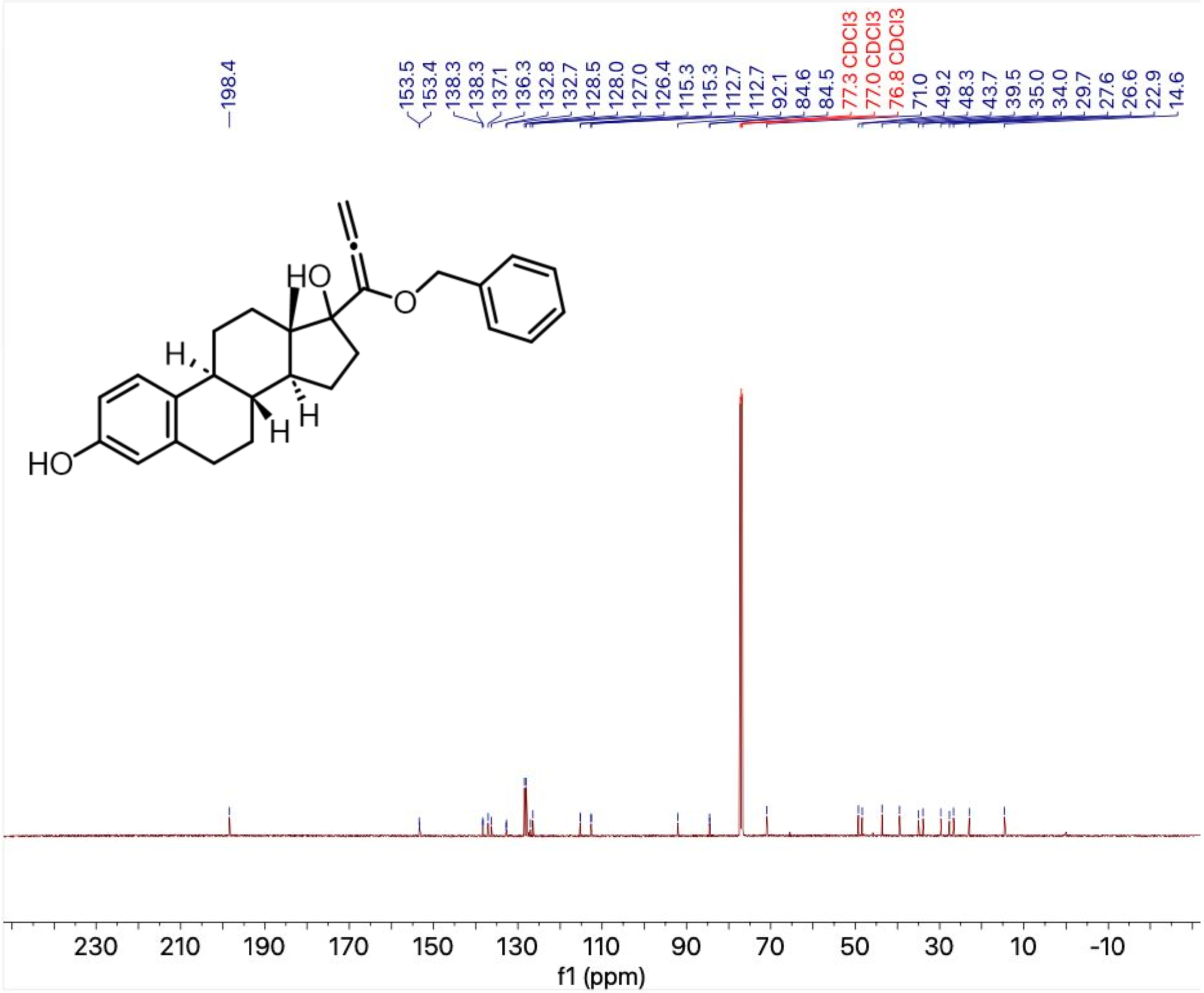

$^{1}H\{^{13}C\}$ 500 MHz NMR for Compound 3l.

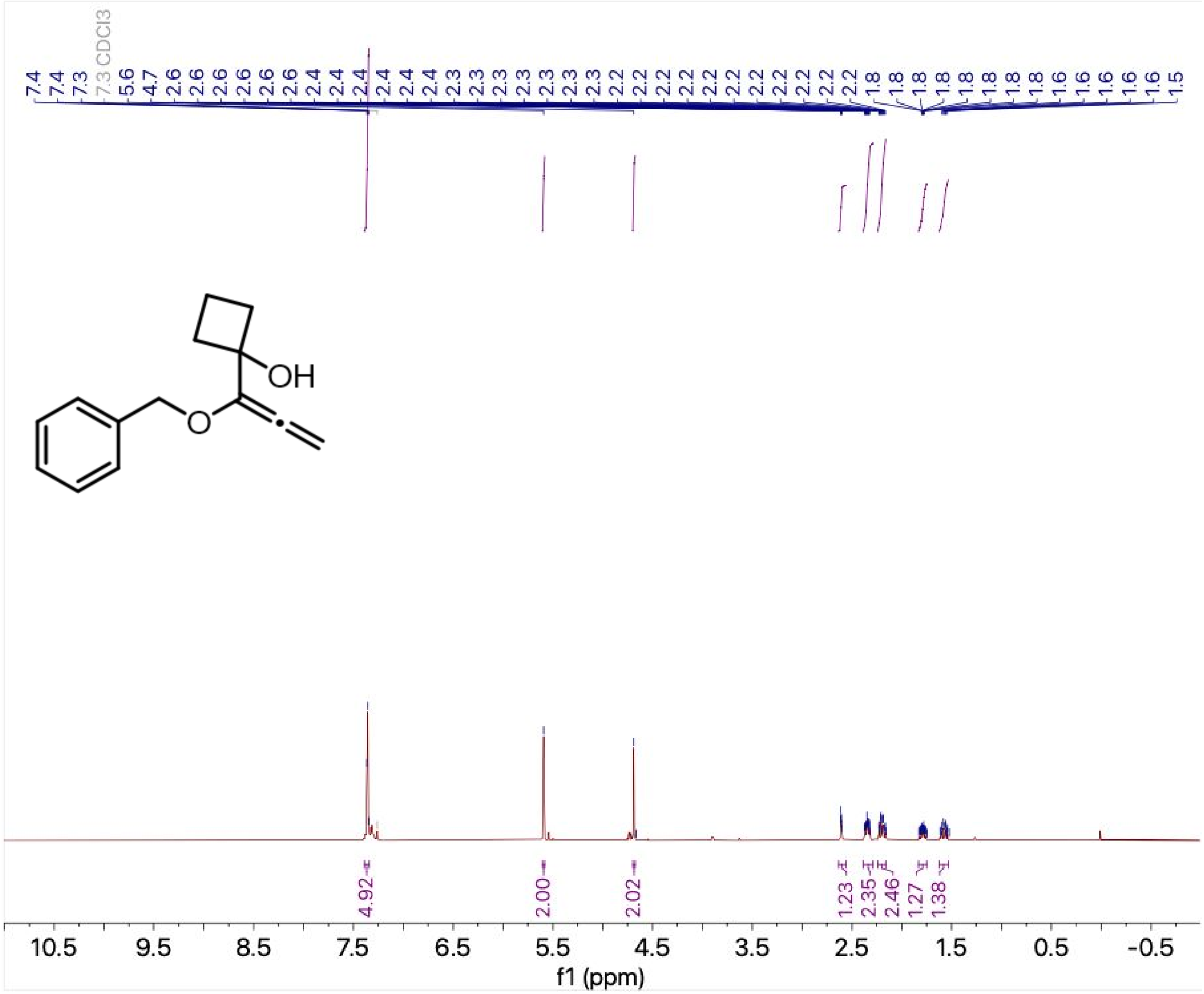

$^{13}C\{^{1}H\}$ 500 MHz NMR for Compound 3l.

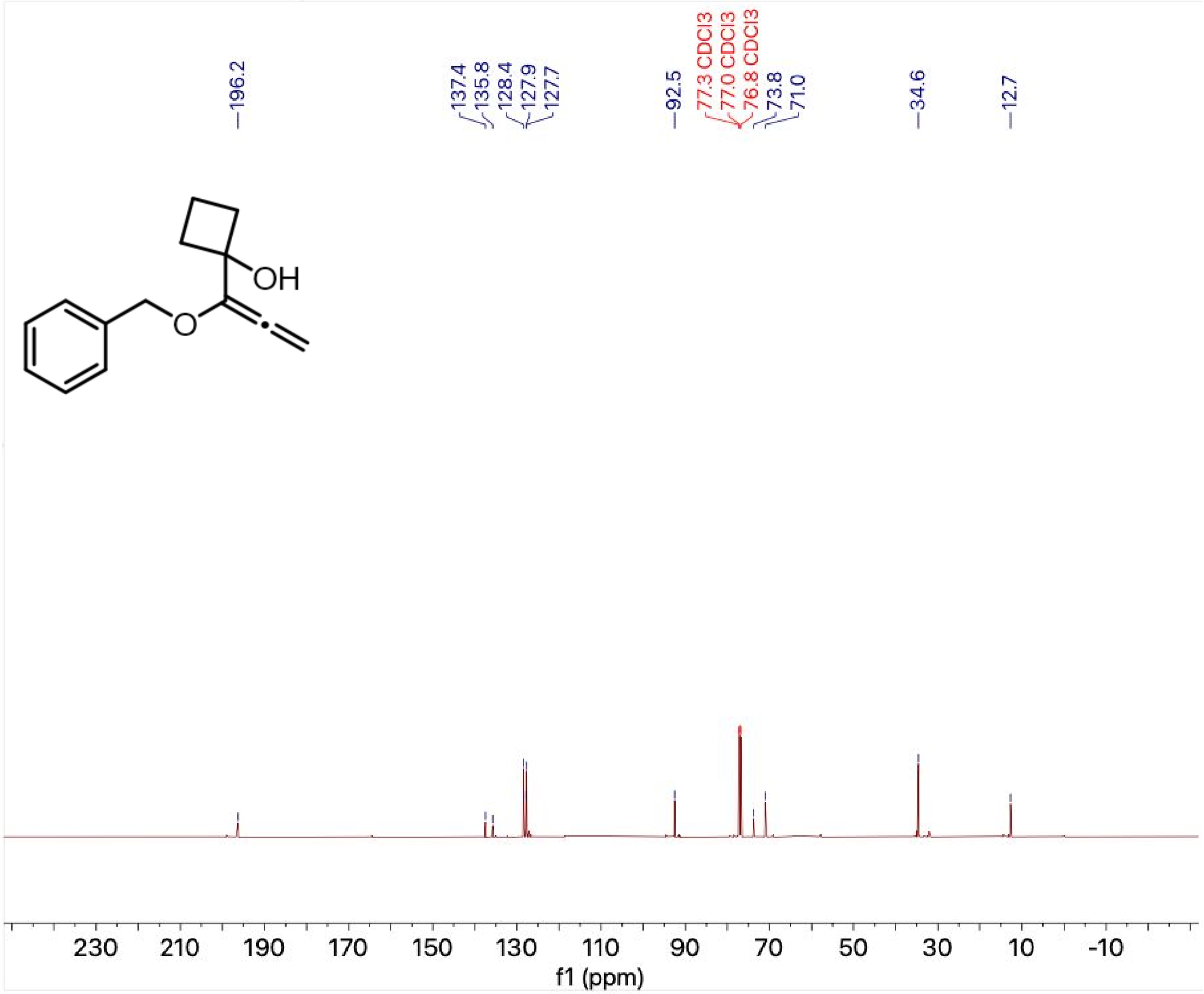

$^{1}H\{^{13}C\}$ **500 MHz NMR for Compound 3m.**

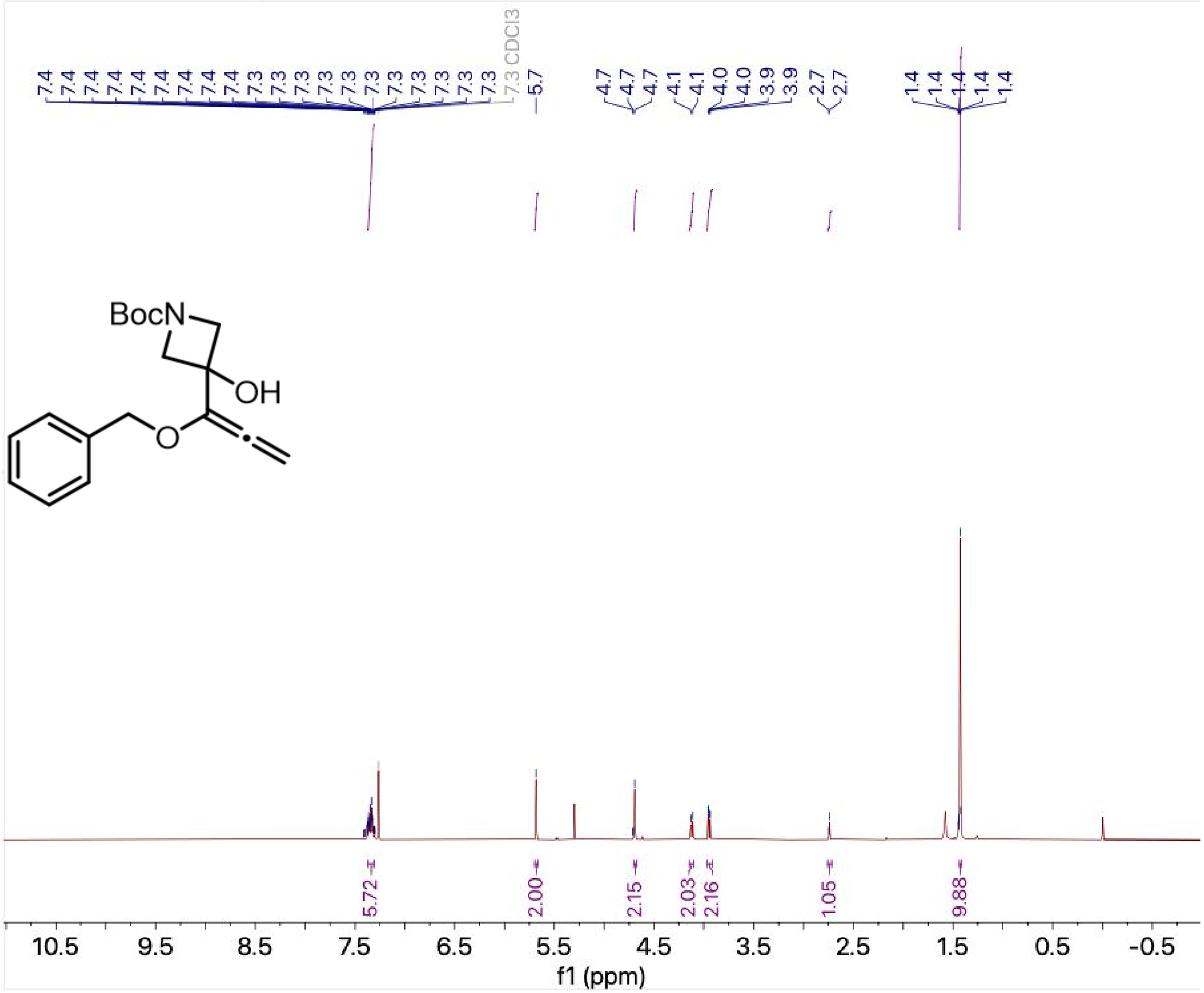

$^{13}C\{^{1}H\}$ 500 MHz NMR for Compound 3m.

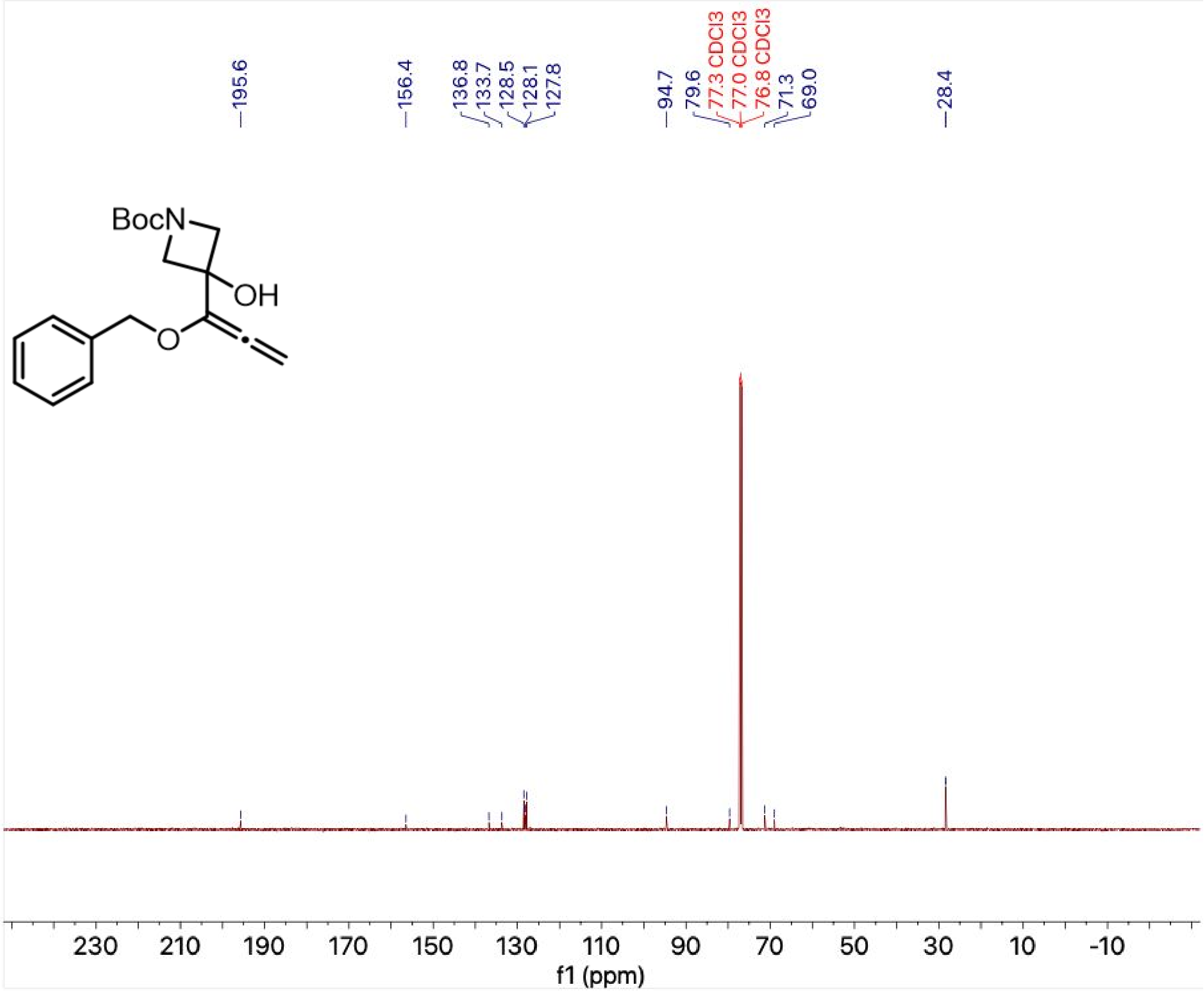

H{^{13}C} 500 MHz NMR for Compound 3n.

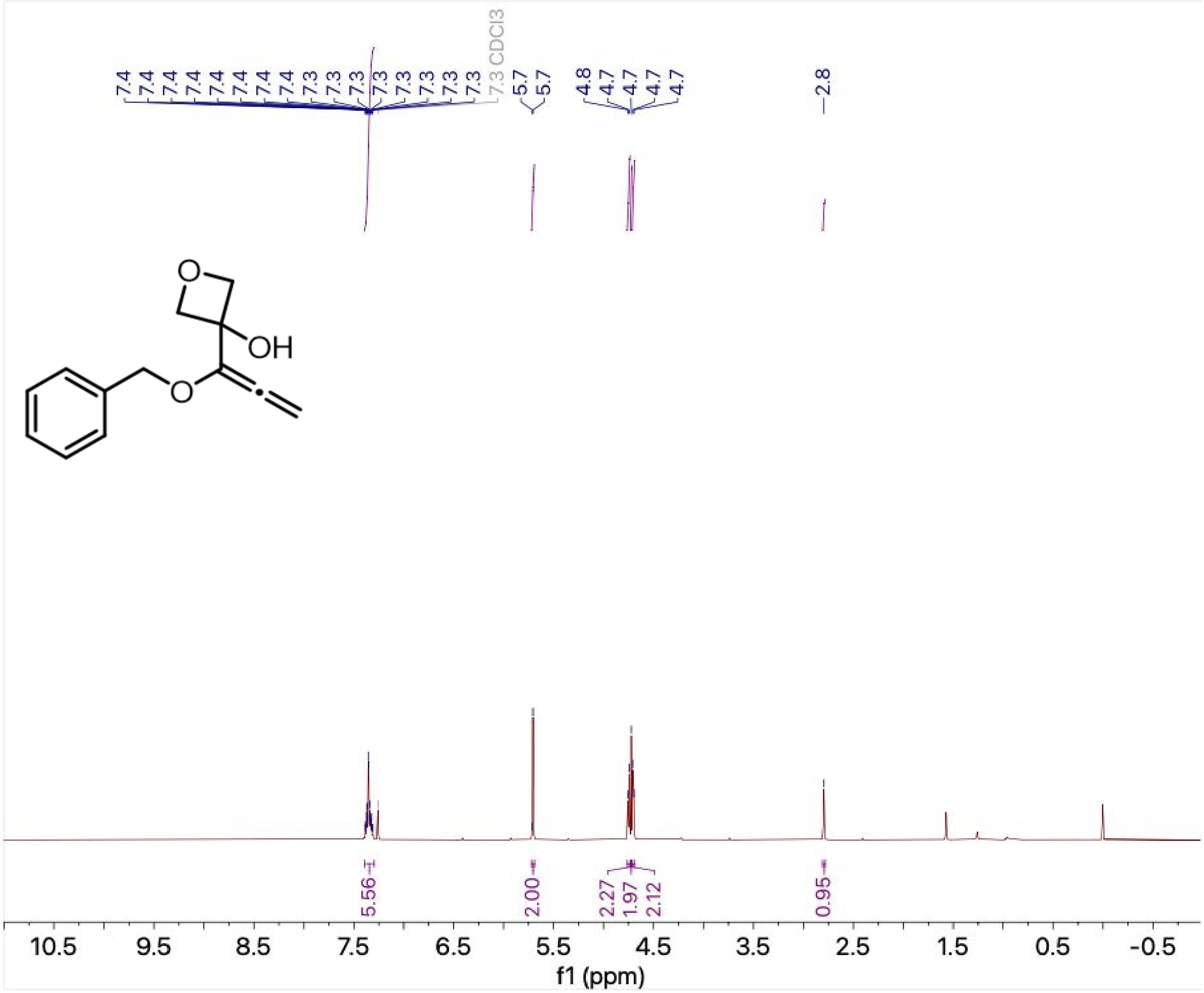

$^{13}C\{^1H\}$ 500 MHz NMR for Compound 3n.

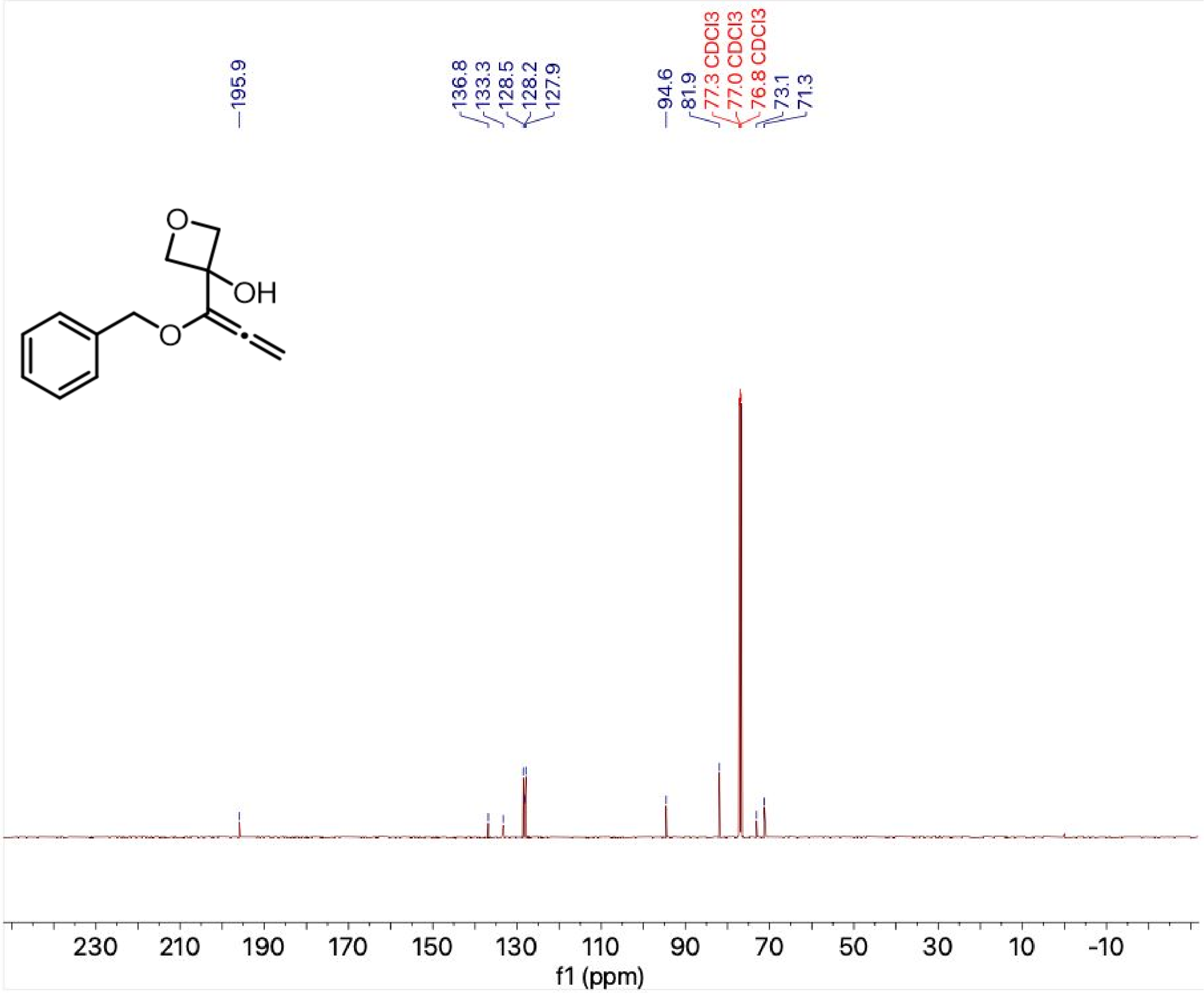

$^{1}H\{^{13}C\}$ 500 MHz NMR for Compound 3o.

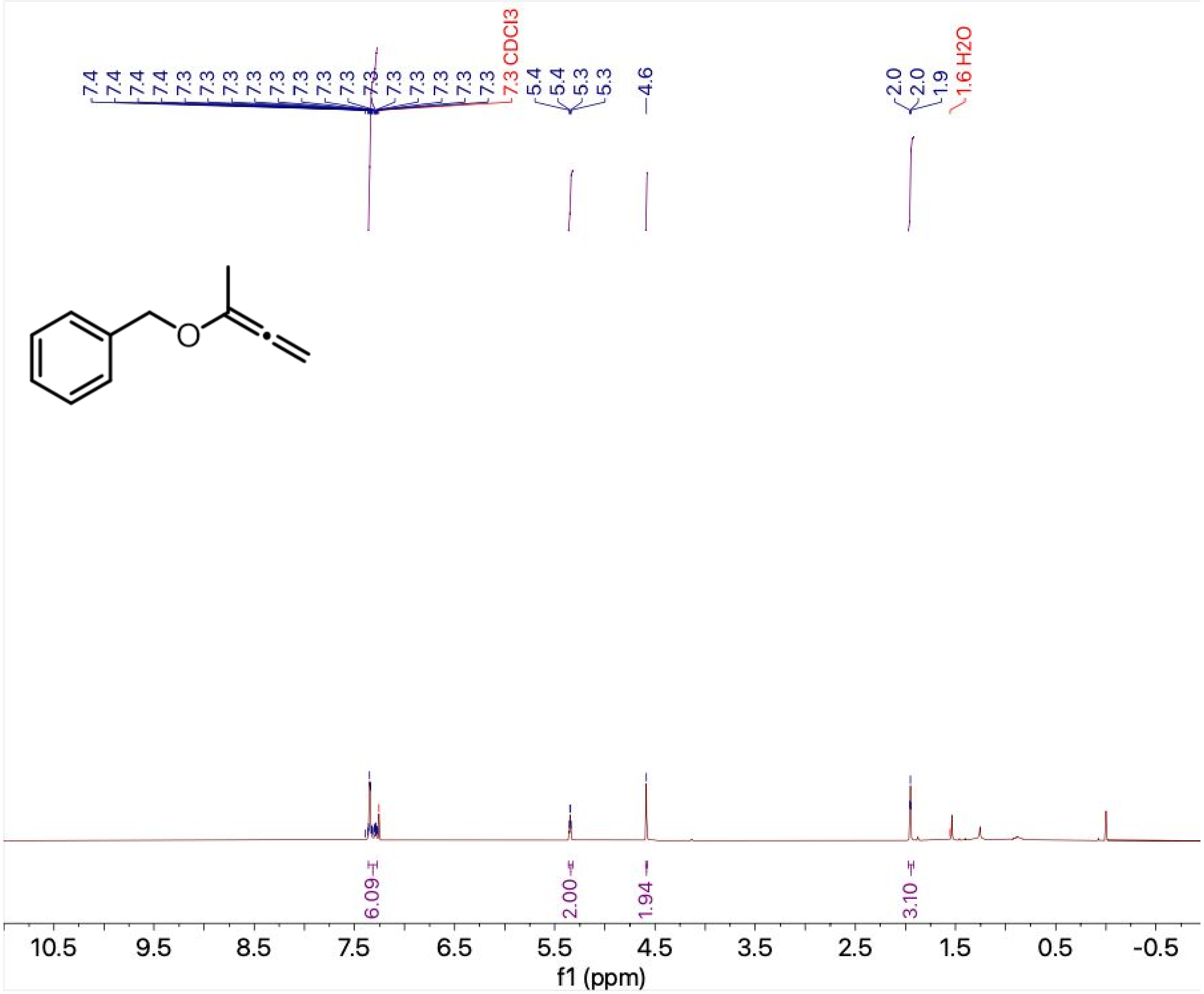

$^{13}C\{^1H\}$ **500 MHz NMR for Compound 3o.**

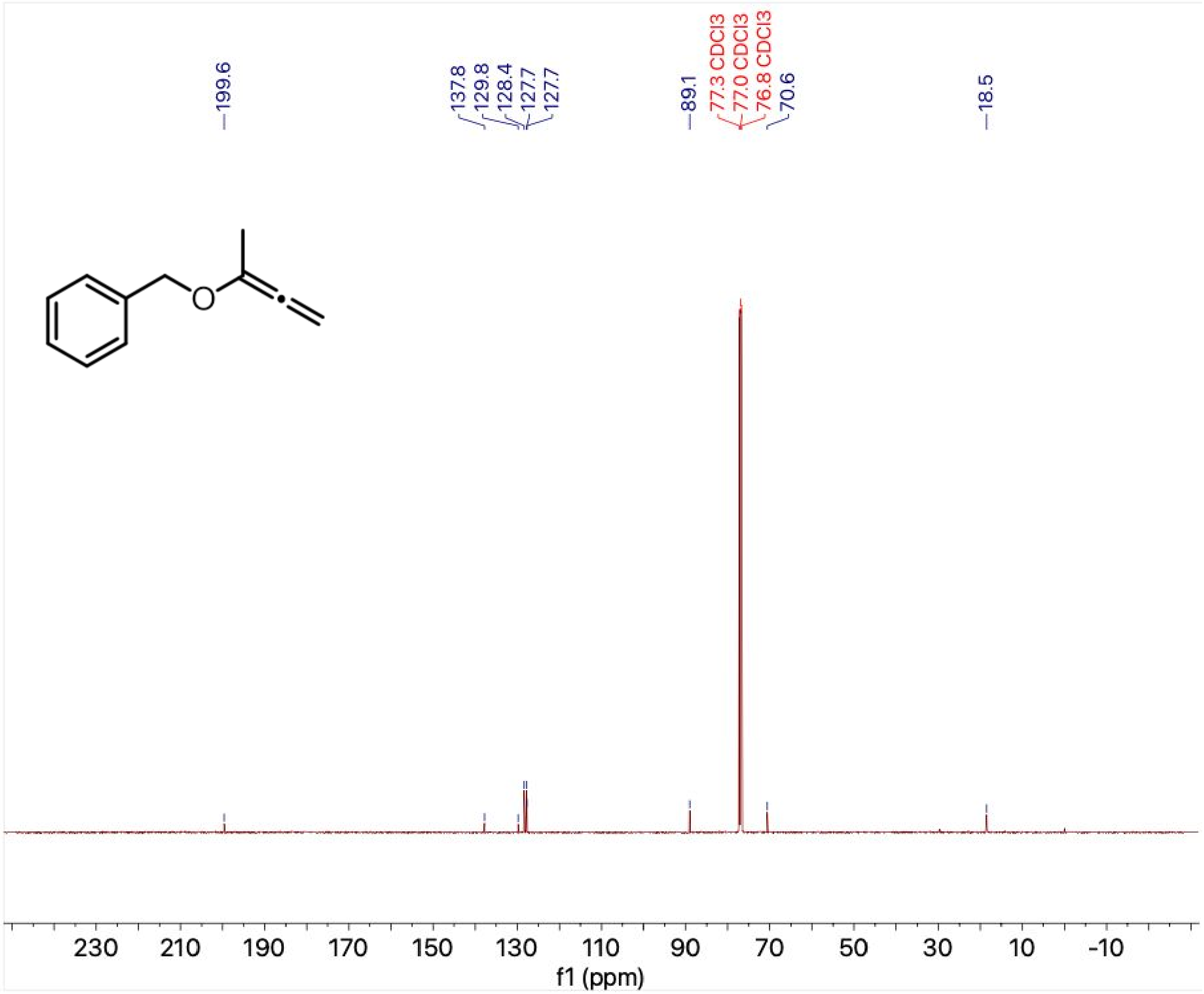

$^{1}H\{^{13}C\}$ 500 MHz NMR for Compound 3r.

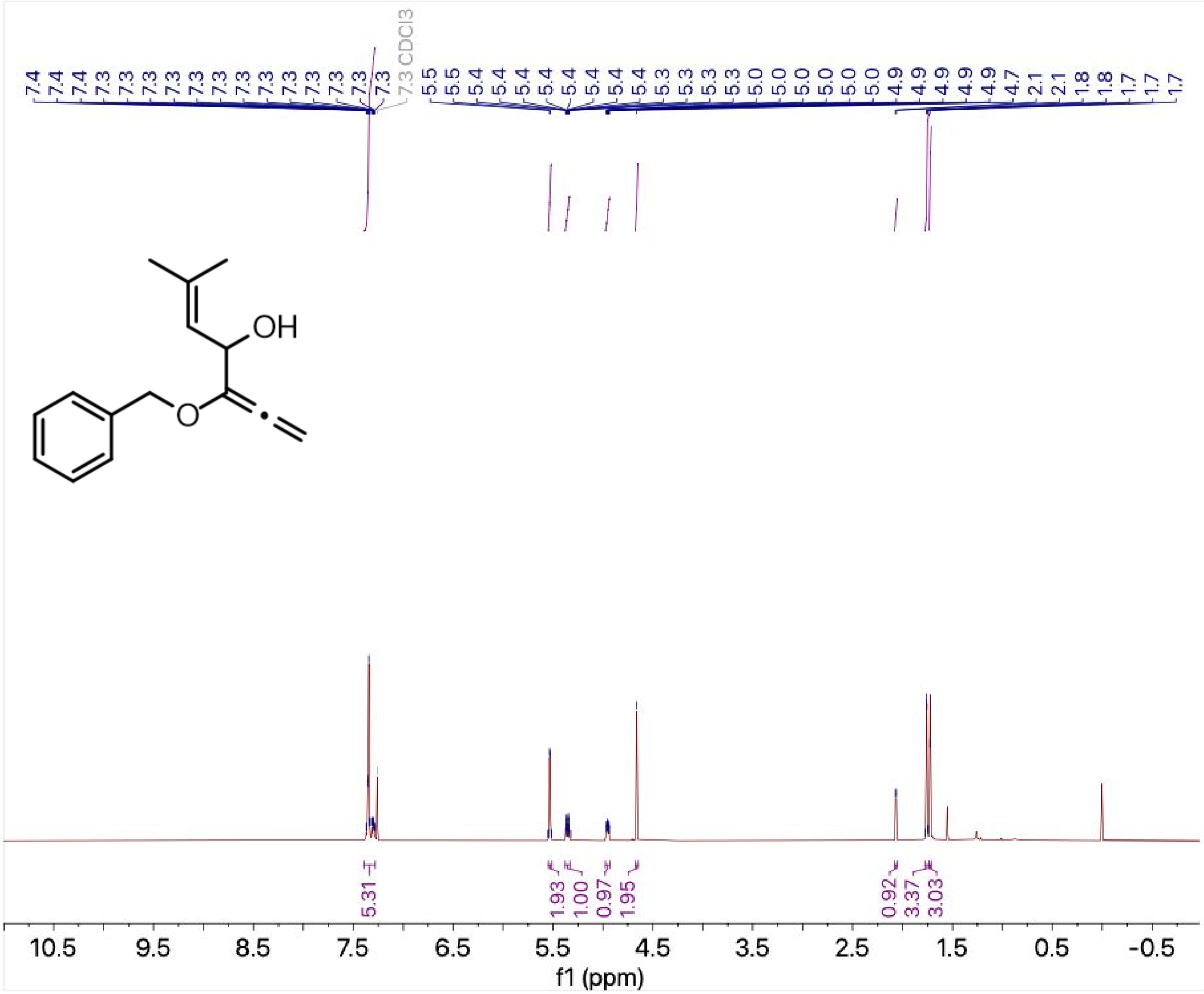

$^{13}C\{^1H\}$ **500 MHz NMR for Compound 3r.**

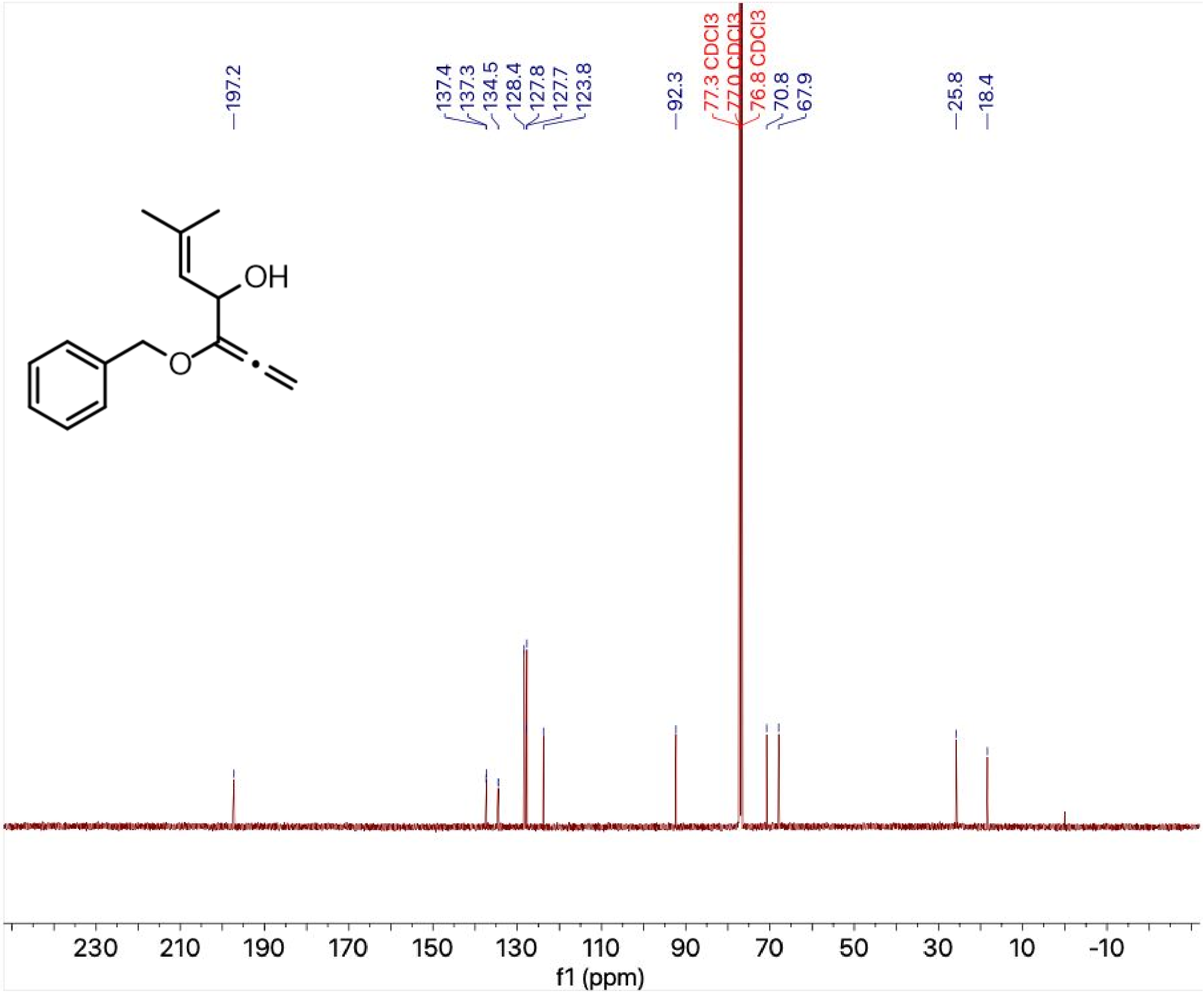

^{1}H{^{13}C} 500 MHz NMR for Compound 3s.

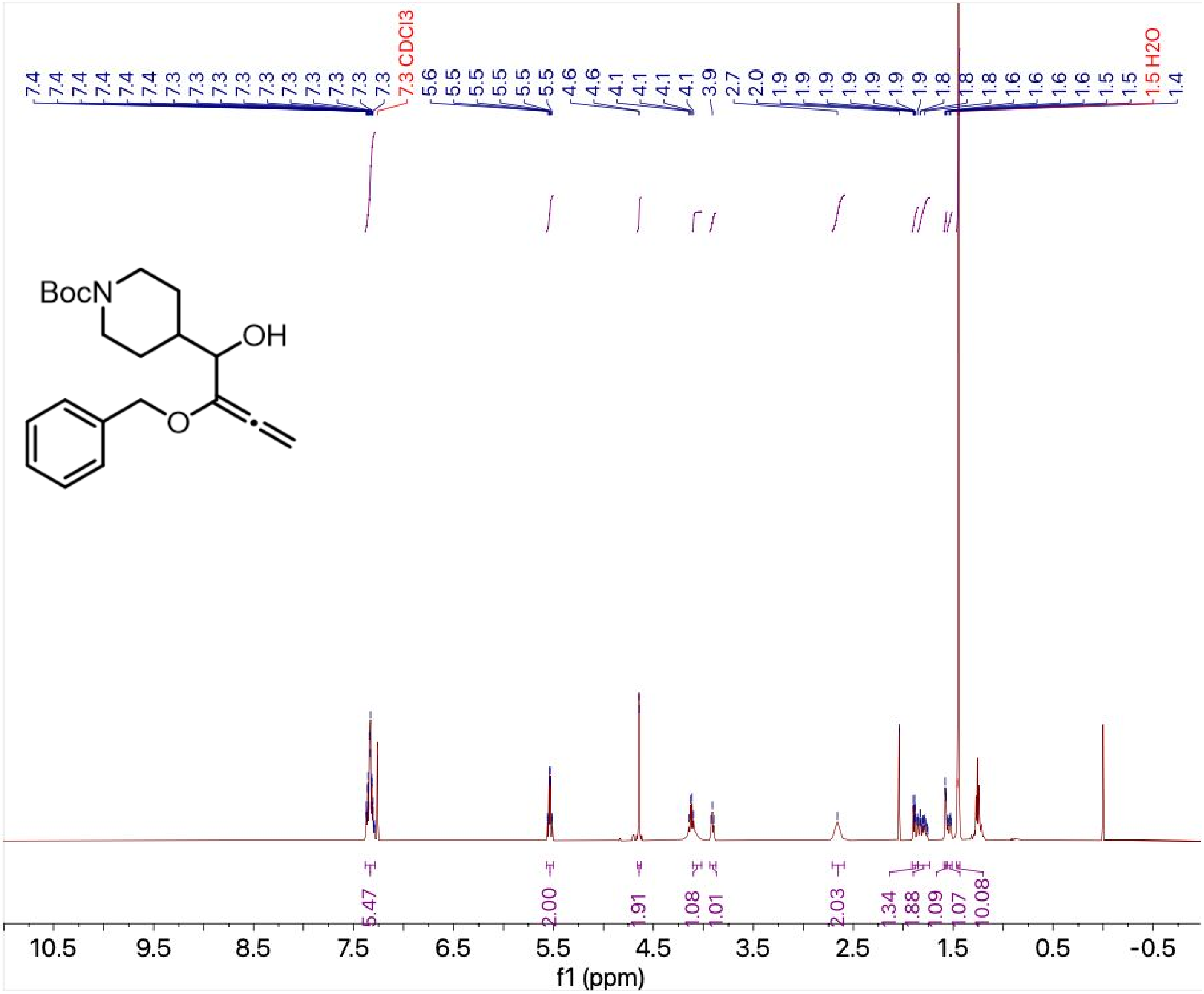

$^{13}C\{^{1}H\}$ 500 MHz NMR for Compound 3s.

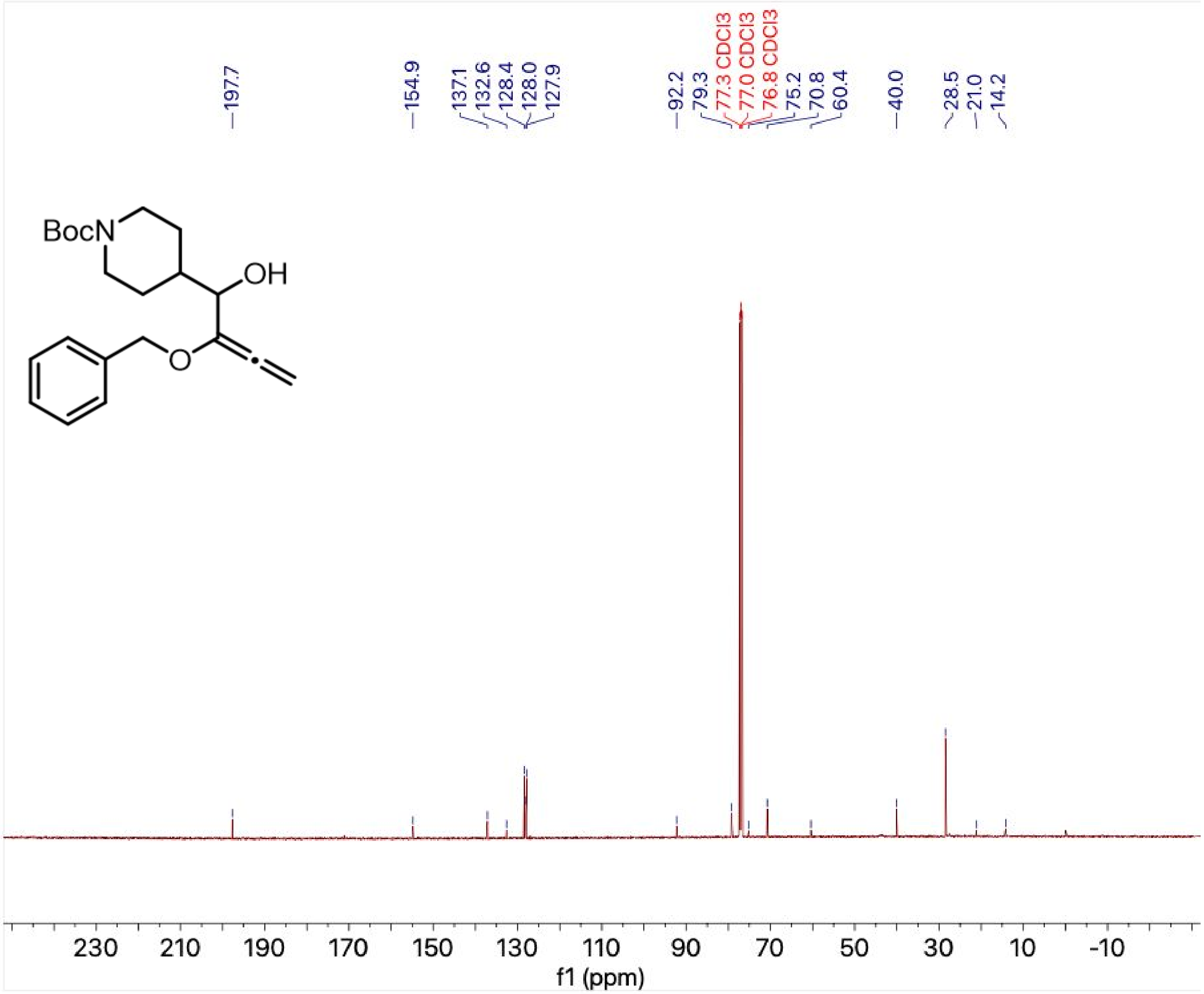

$^1H\{^{13}C\}$ 500 MHz NMR for Compound 3t.

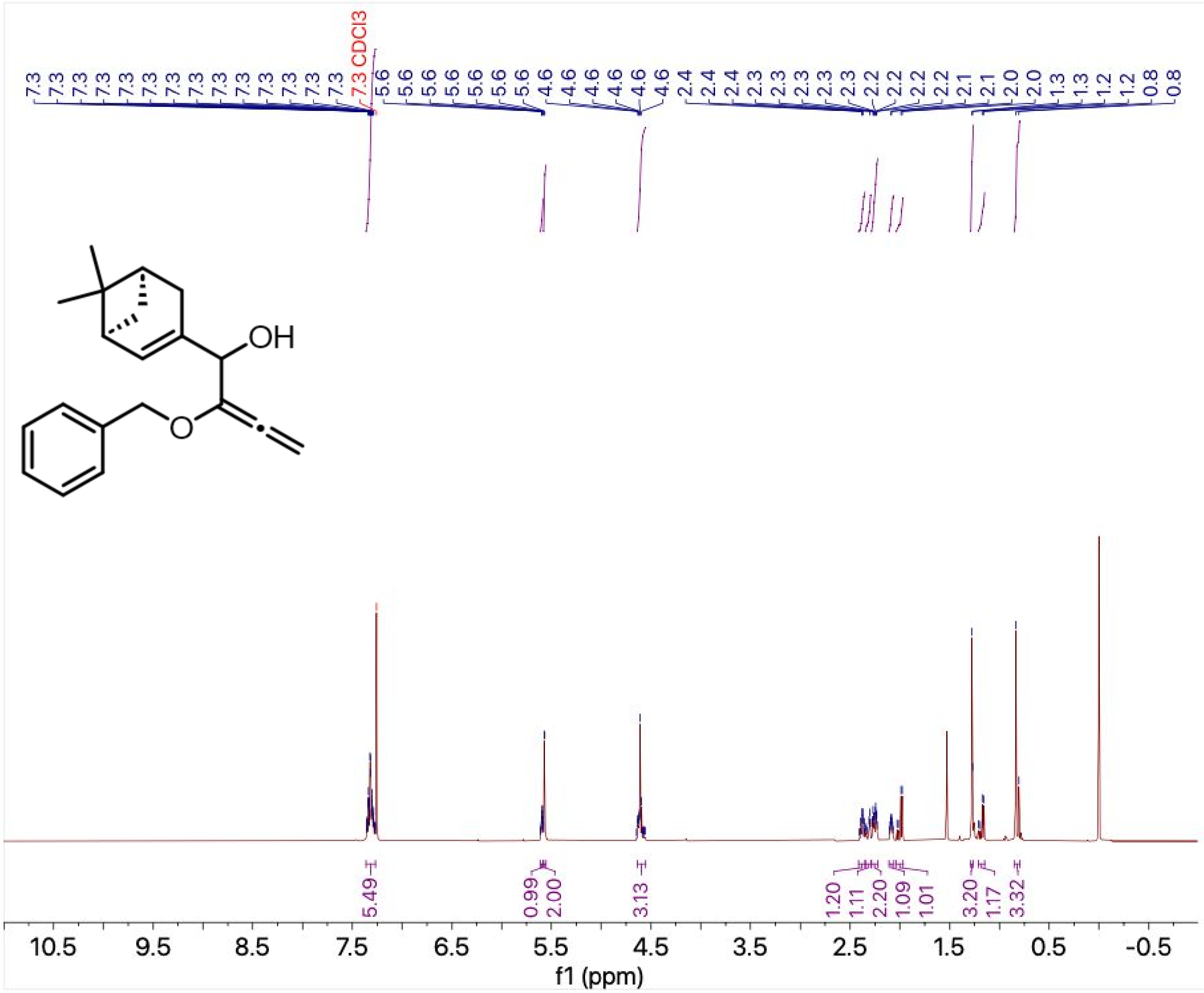

$^{13}C\{^1H\}$ 500 MHz NMR for Compound 3t.

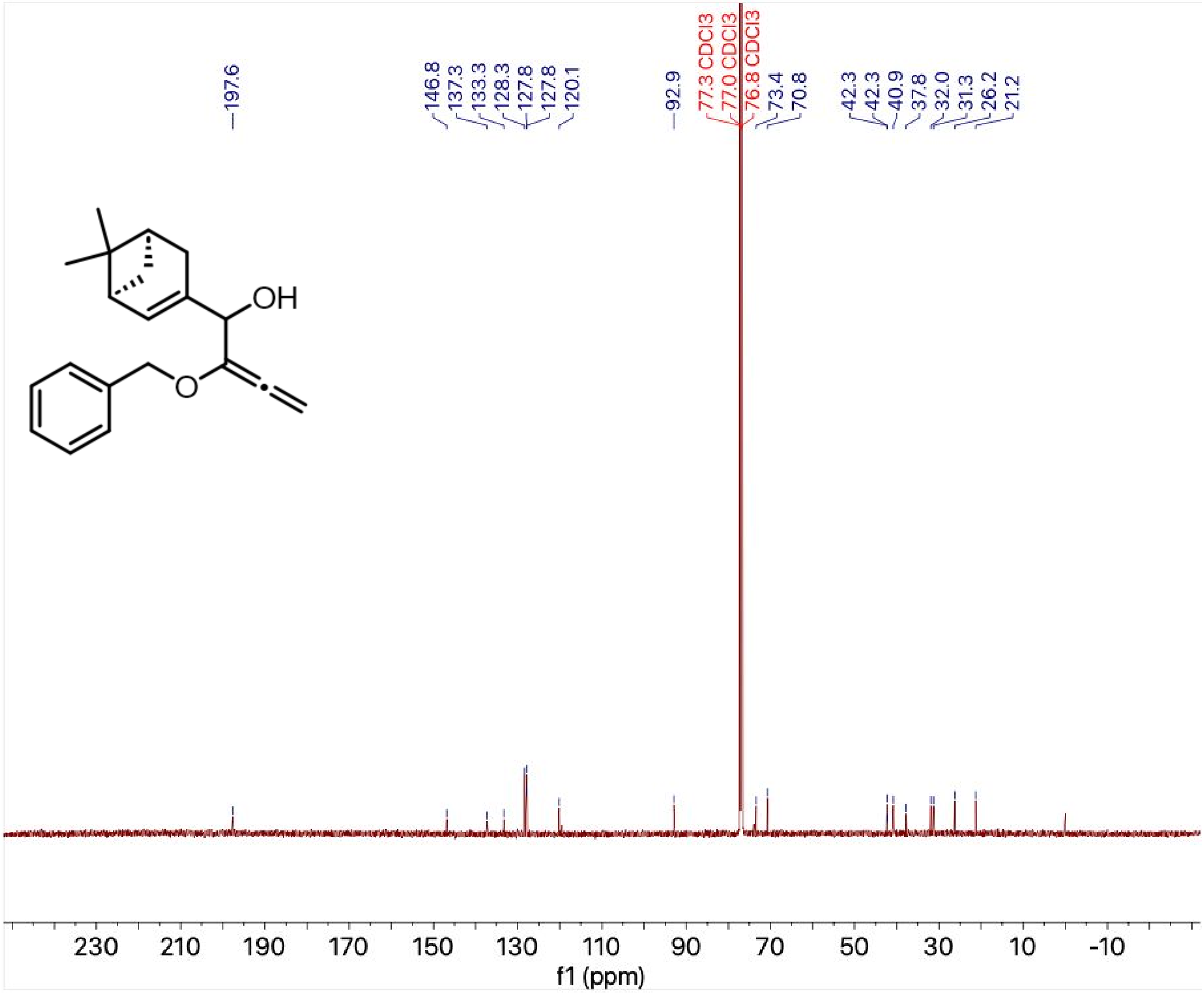

^{1}H{^{13}C} 500 MHz NMR for Compound 3u.

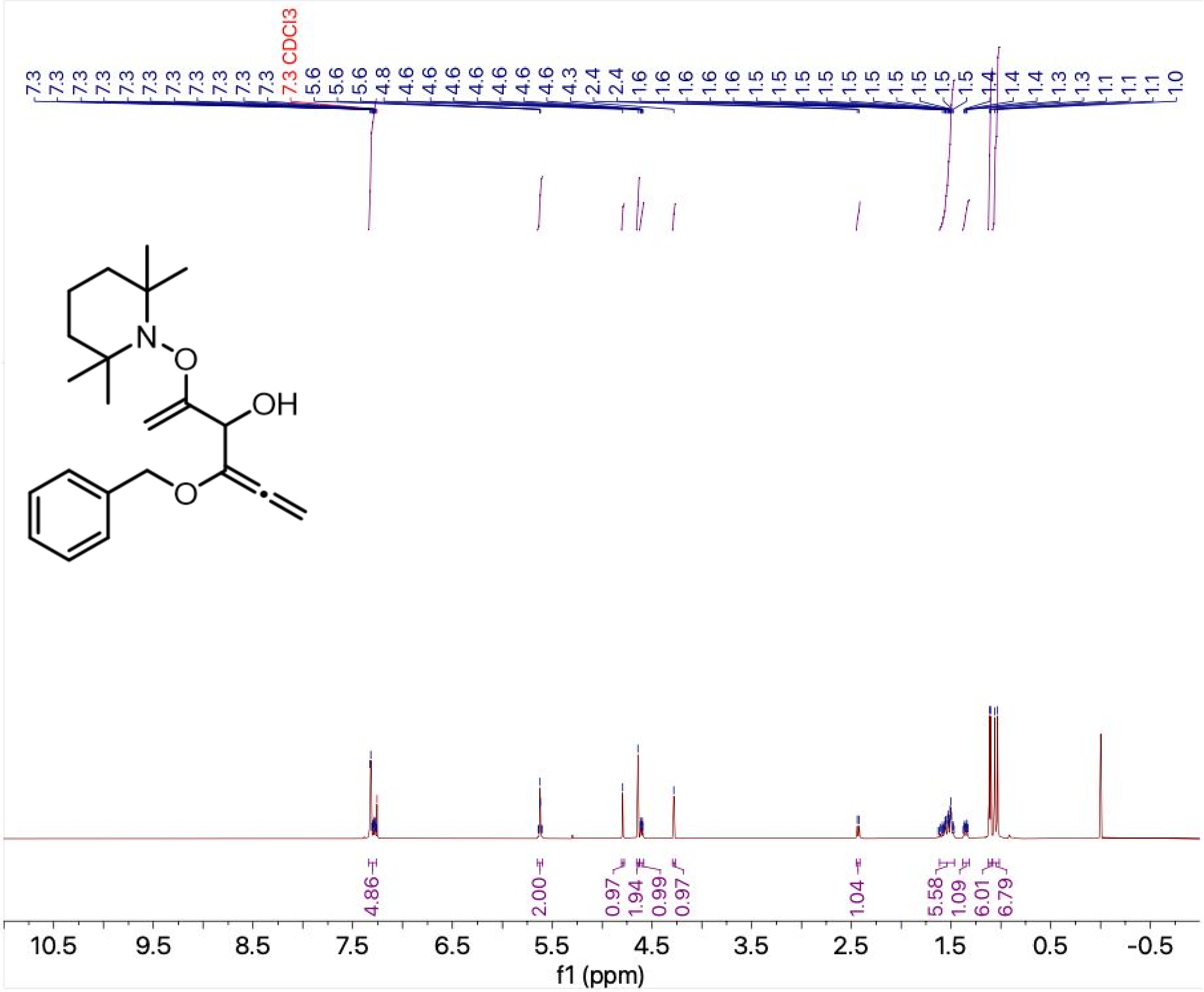

$^{13}C\{^1H\}$ 500 MHz NMR for Compound 3u.

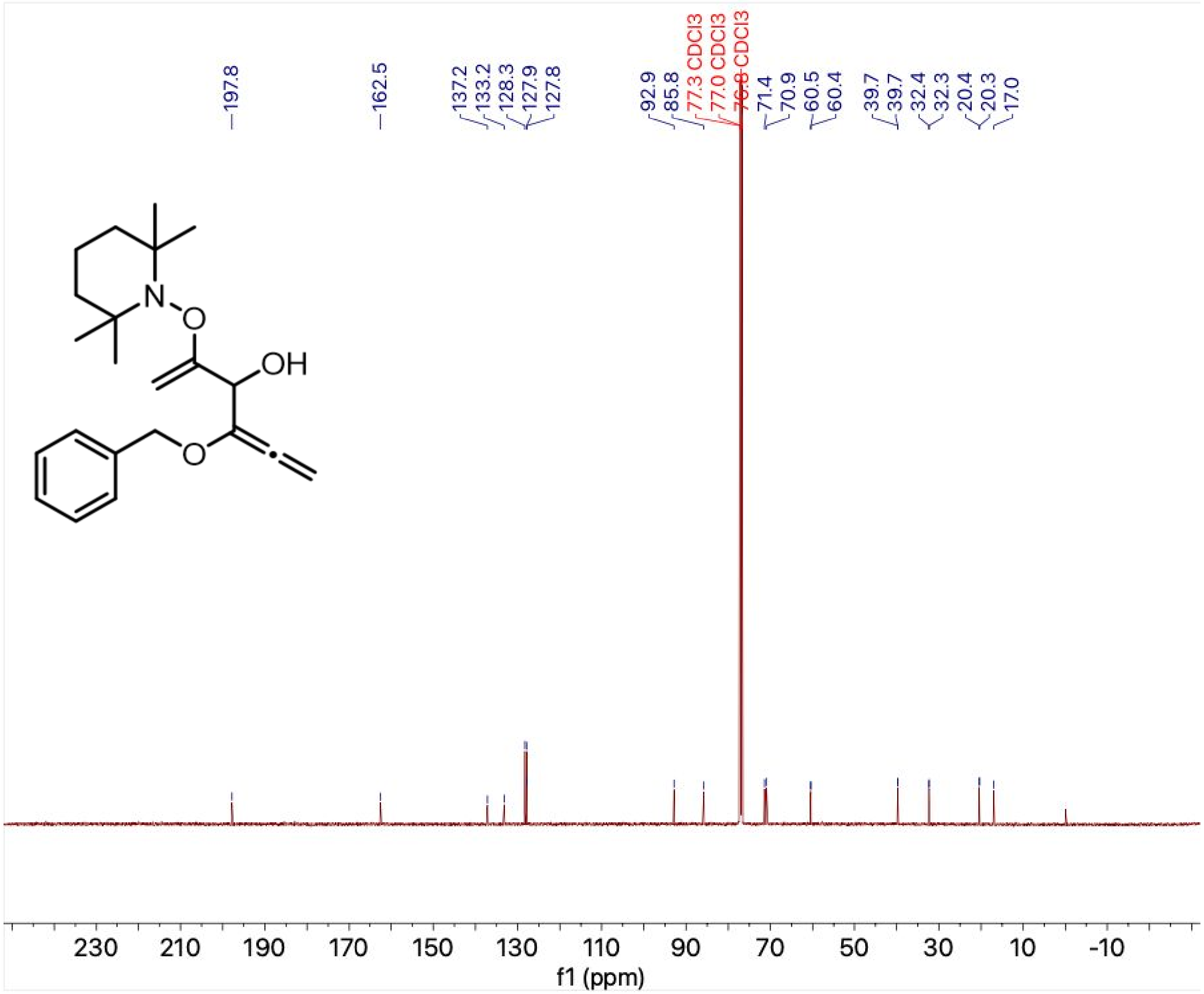

$^{1}H\{^{13}C\}$ 500 MHz NMR for Compound 7v.

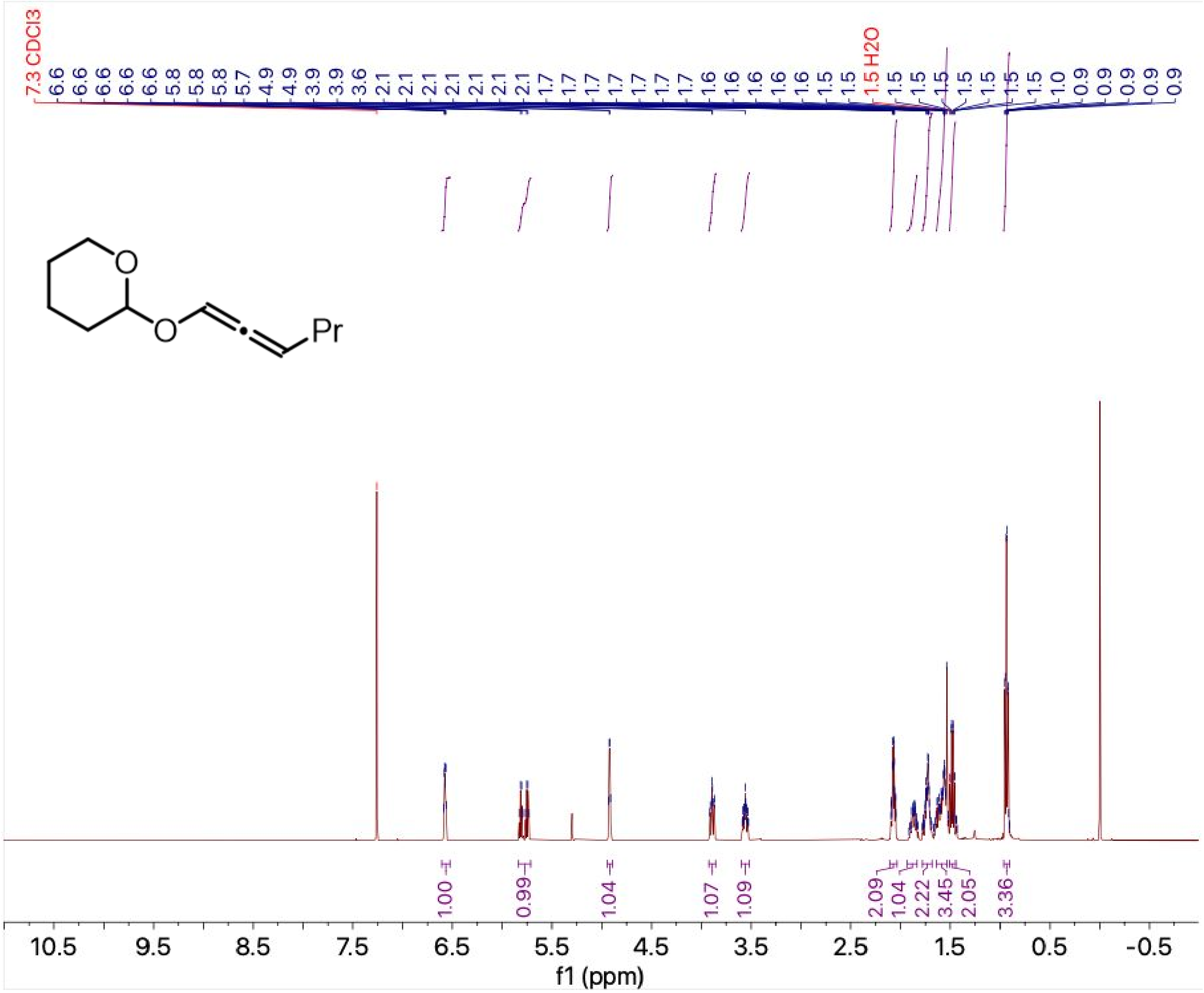

$^{13}C\{^1H\}$ **500 MHz NMR for Compound 7v.**

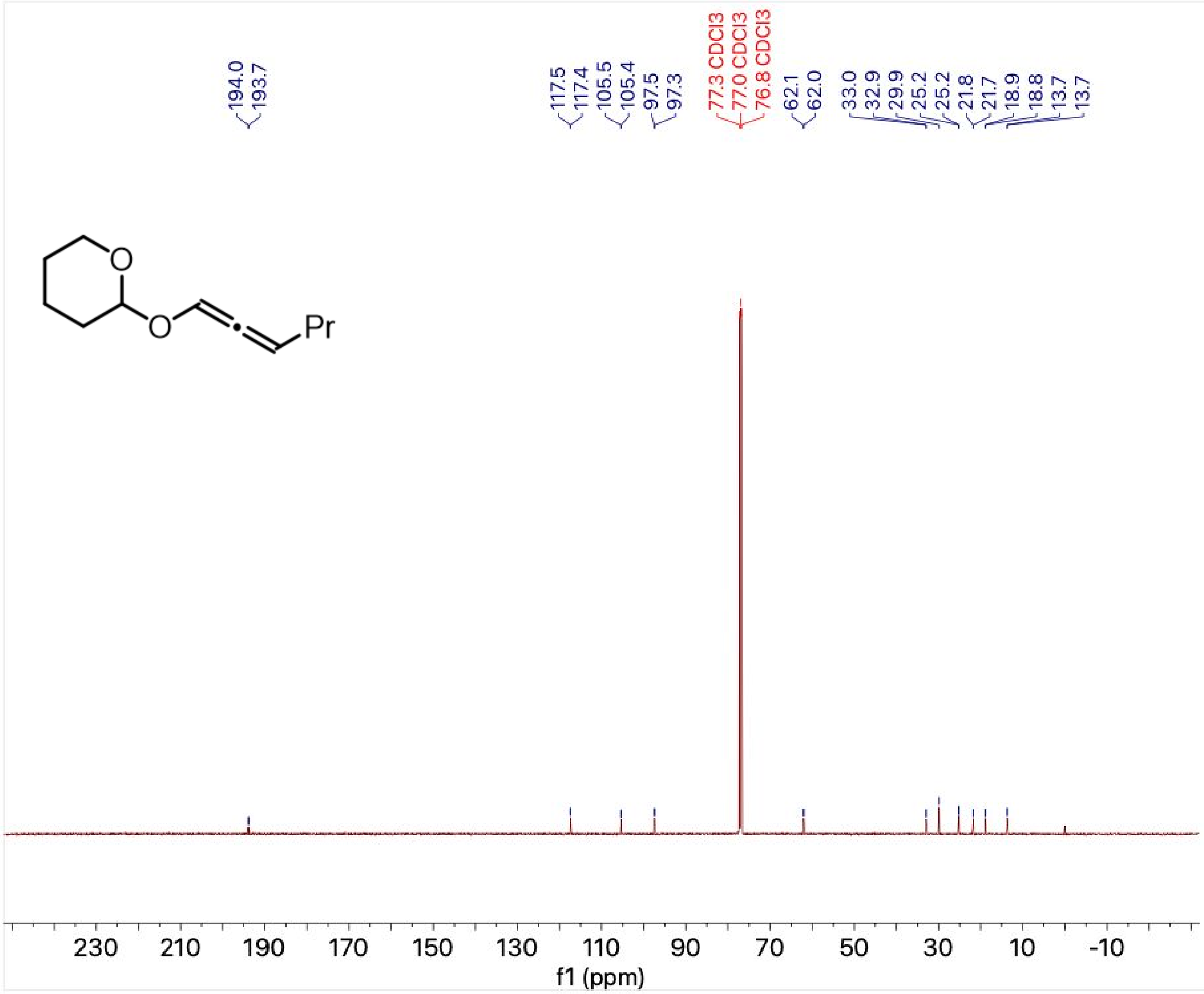

^{1}H{^{13}C} 500 MHz NMR for Compound 7w.

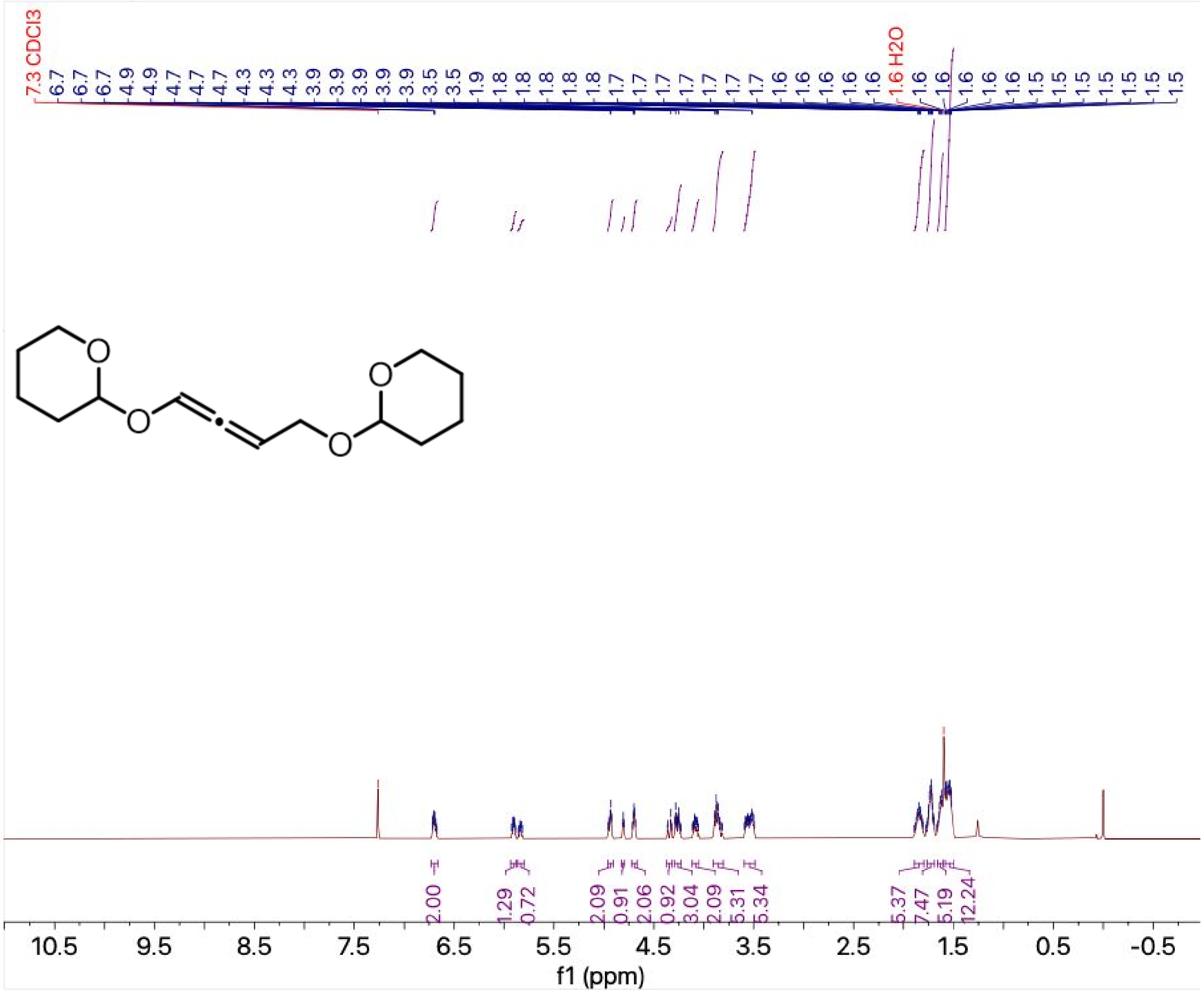

$^{13}C\{^{1}H\}$ 500 MHz NMR for Compound 7w.

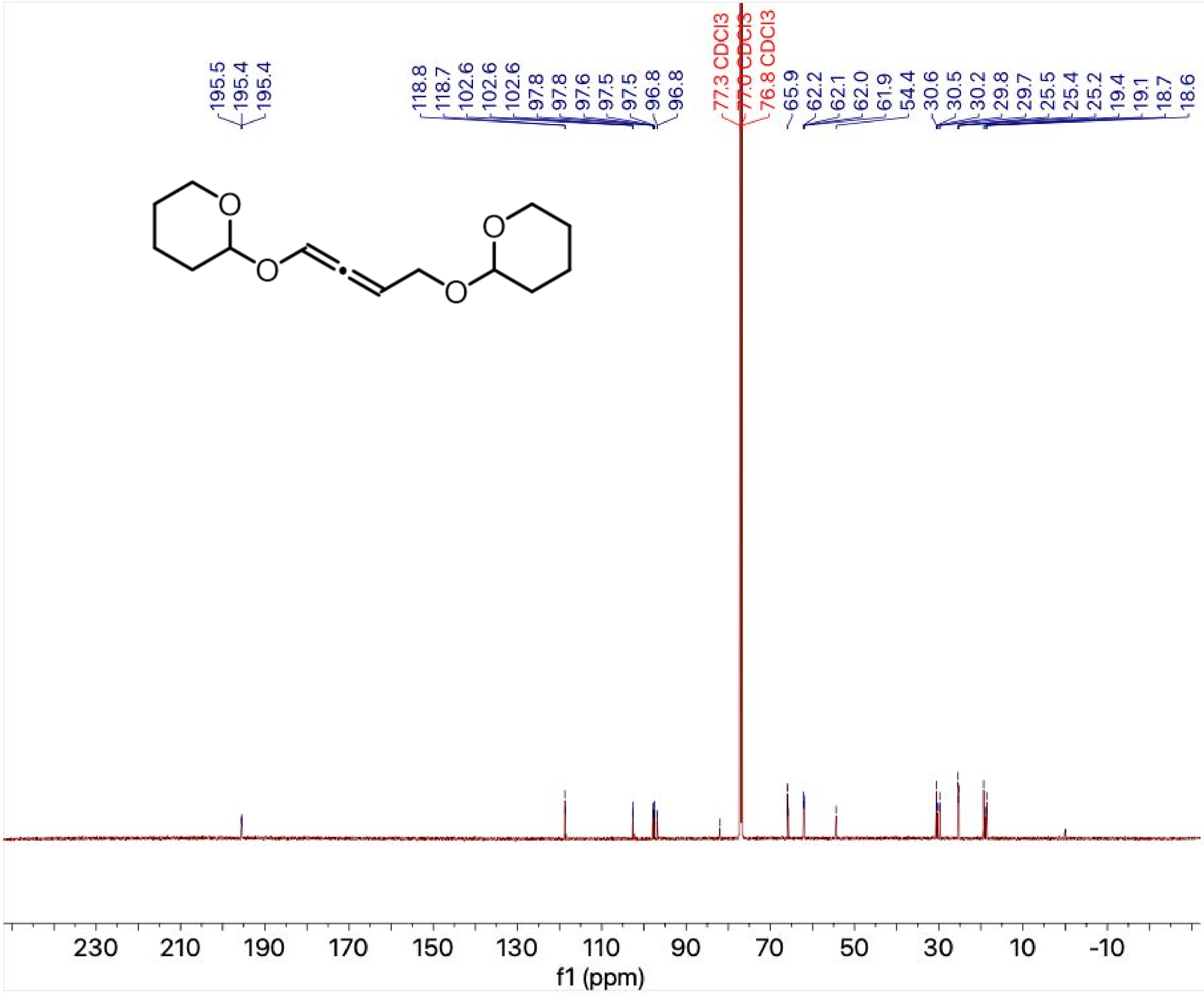

^{1}H{^{13}C} 500 MHz NMR for Compound 4a.

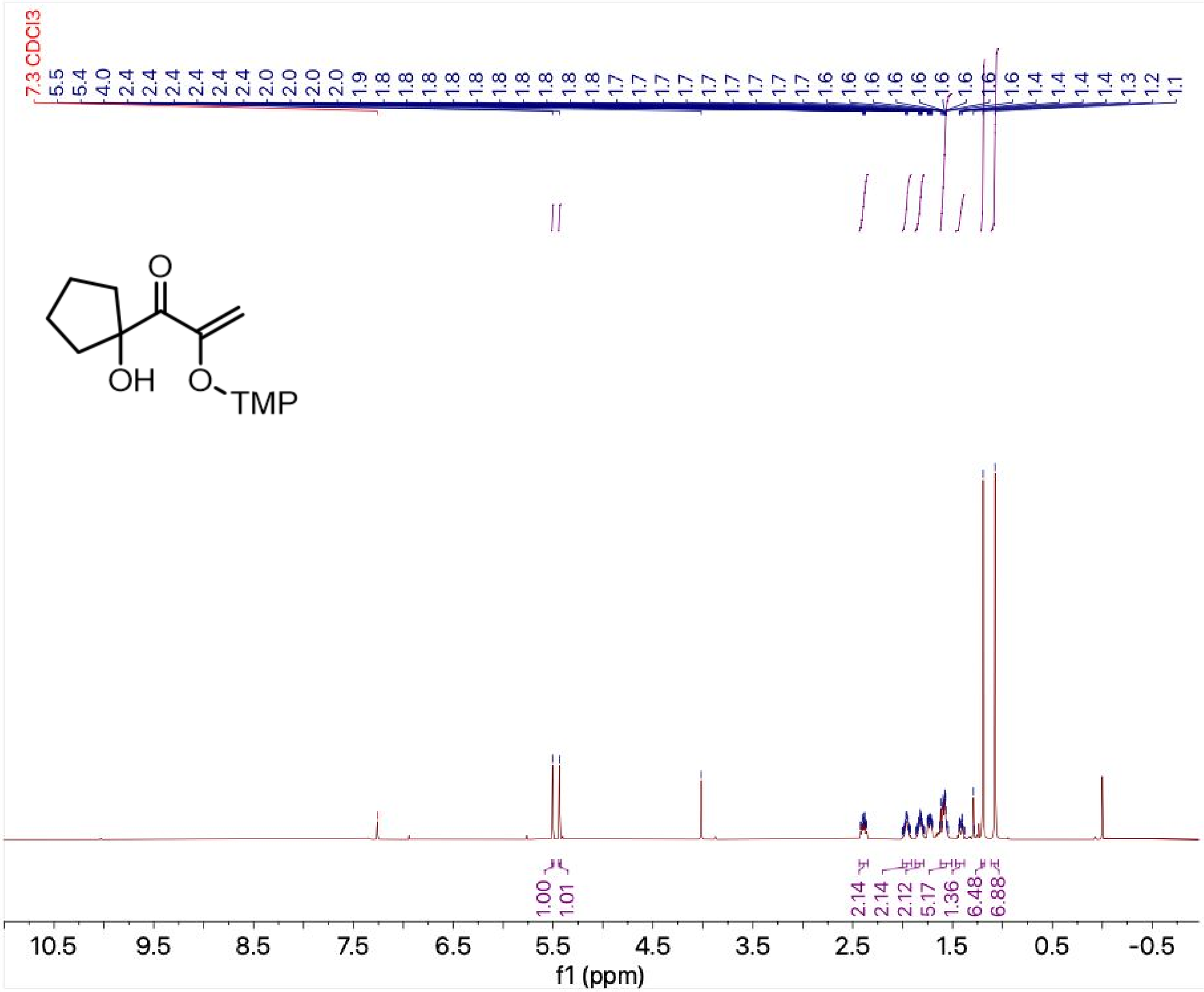

$^{13}C\{^1H\}$ **500 MHz NMR for Compound 4a.**

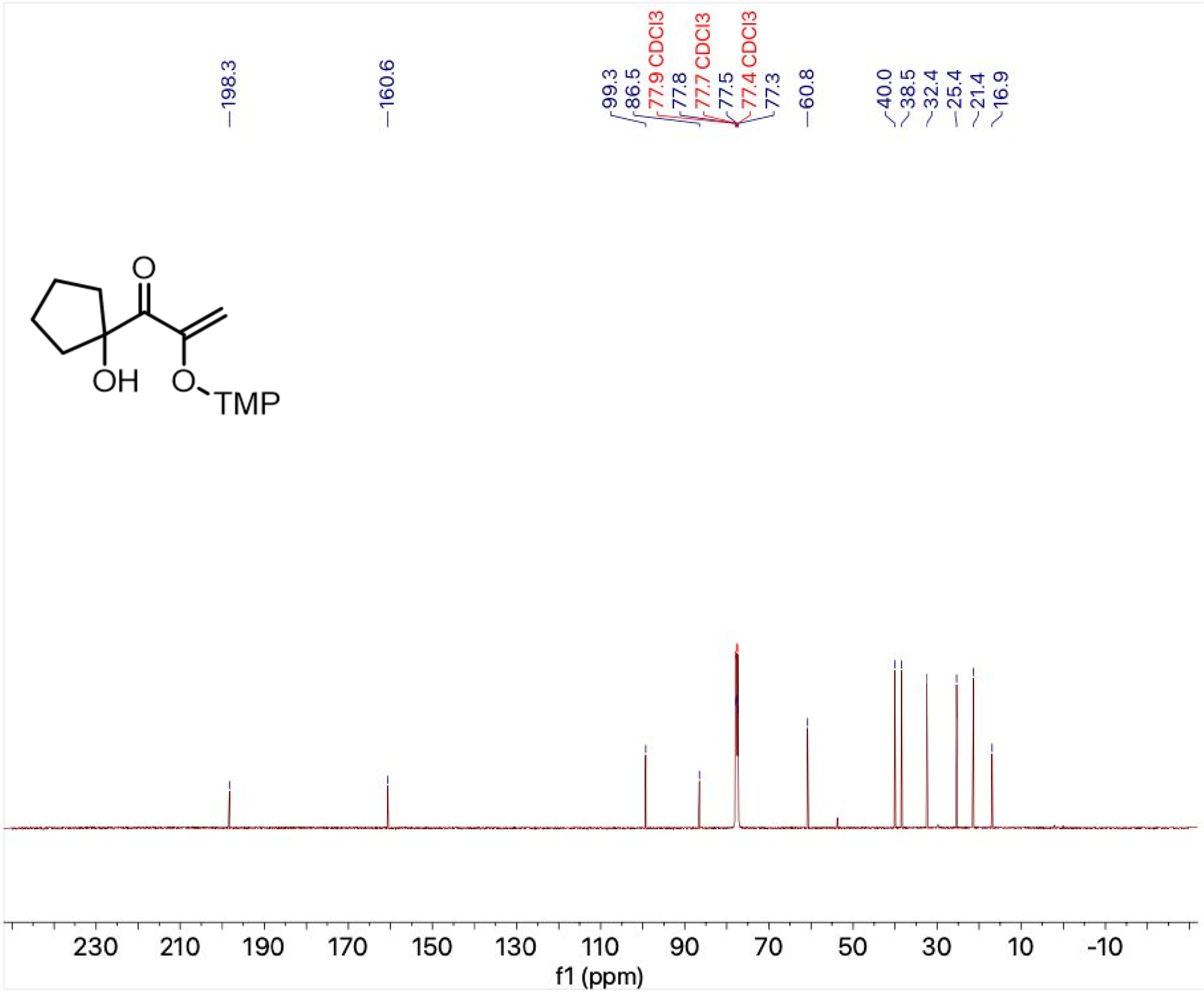

$^{1}H\{^{13}C\}$ **500 MHz NMR for Compound 4b.**

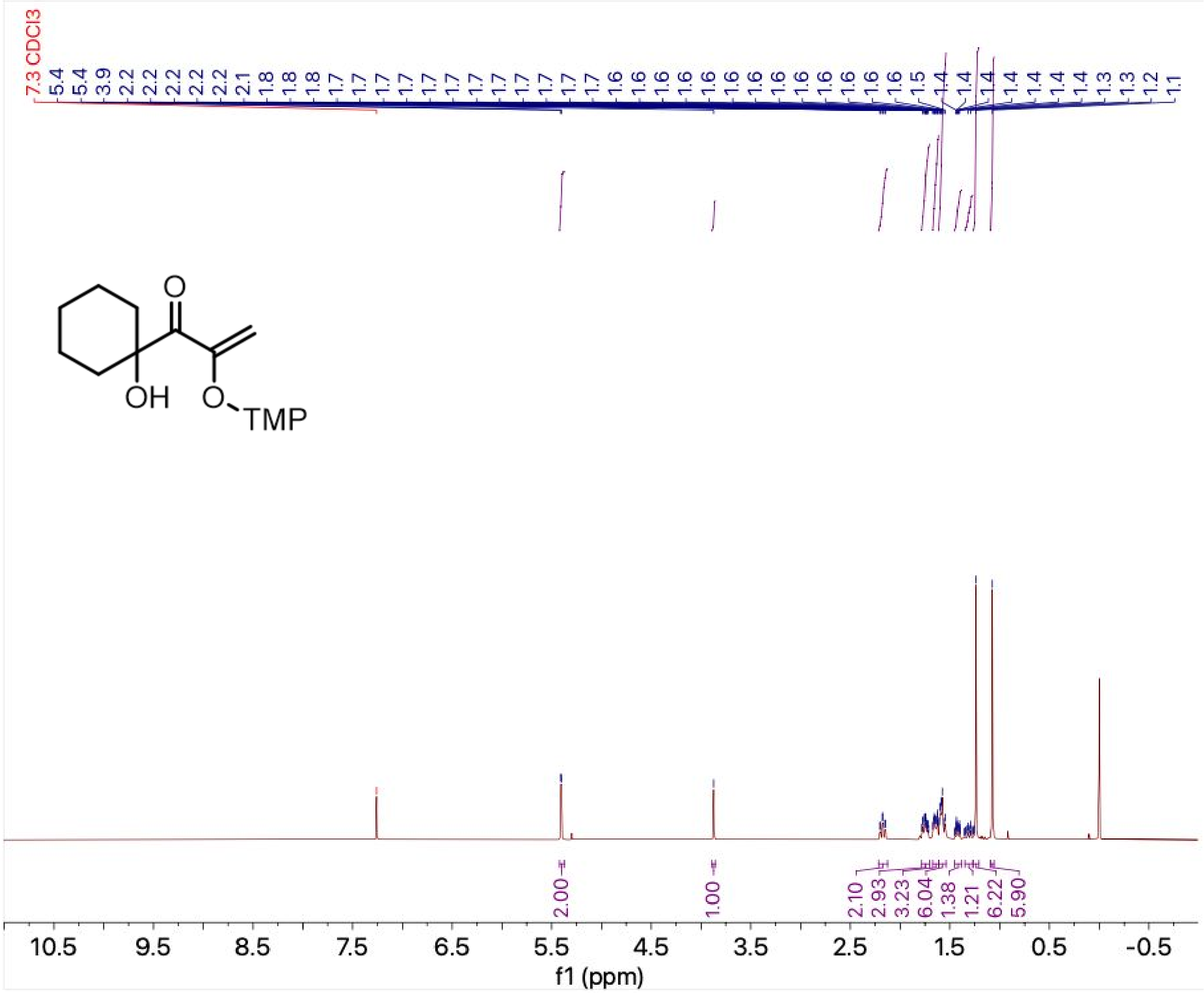

$^{13}C\{^1H\}$ 500 MHz NMR for Compound 4b.

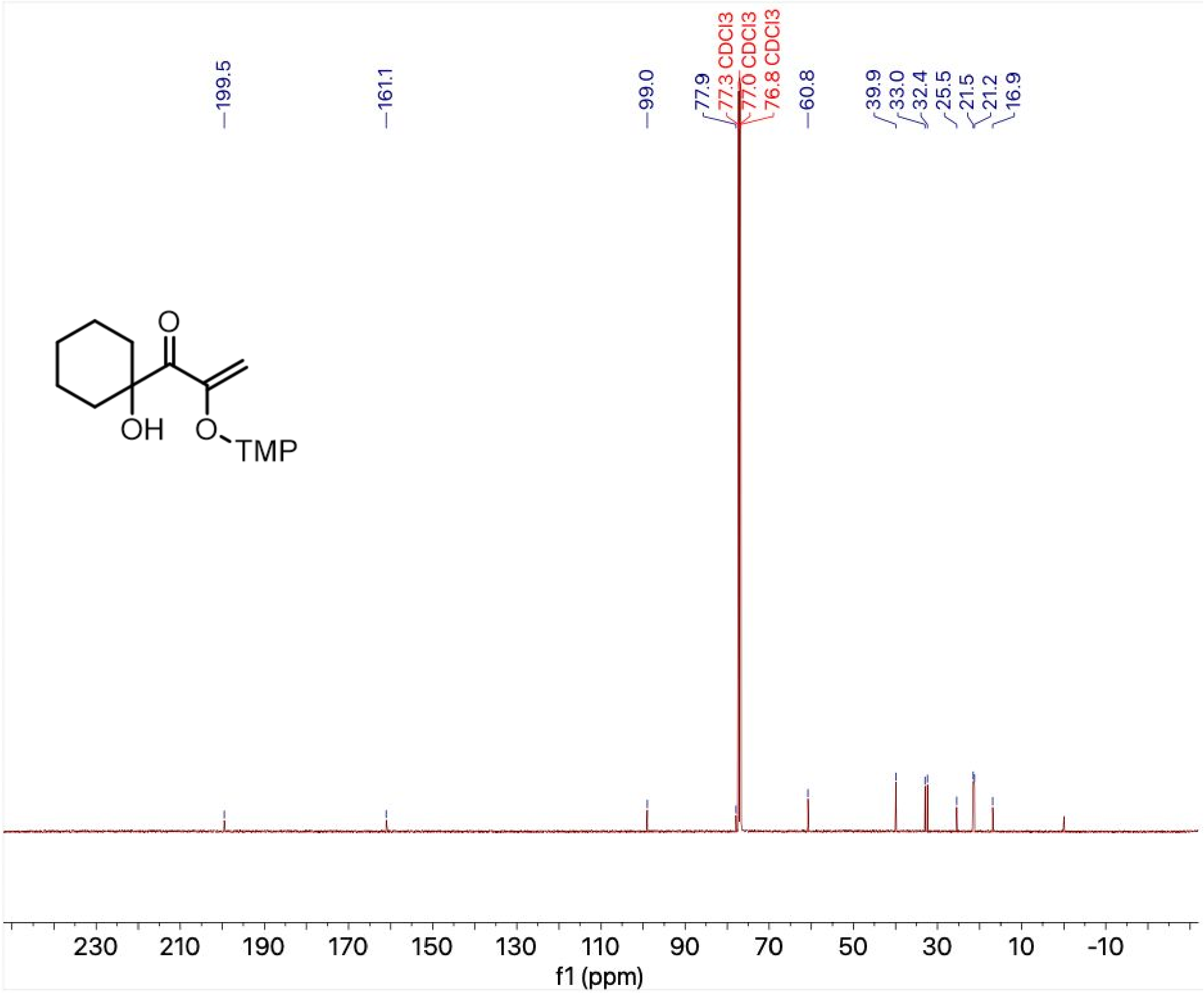

$^{1}H\{^{13}C\}$ 500 MHz NMR for Compound 4c.

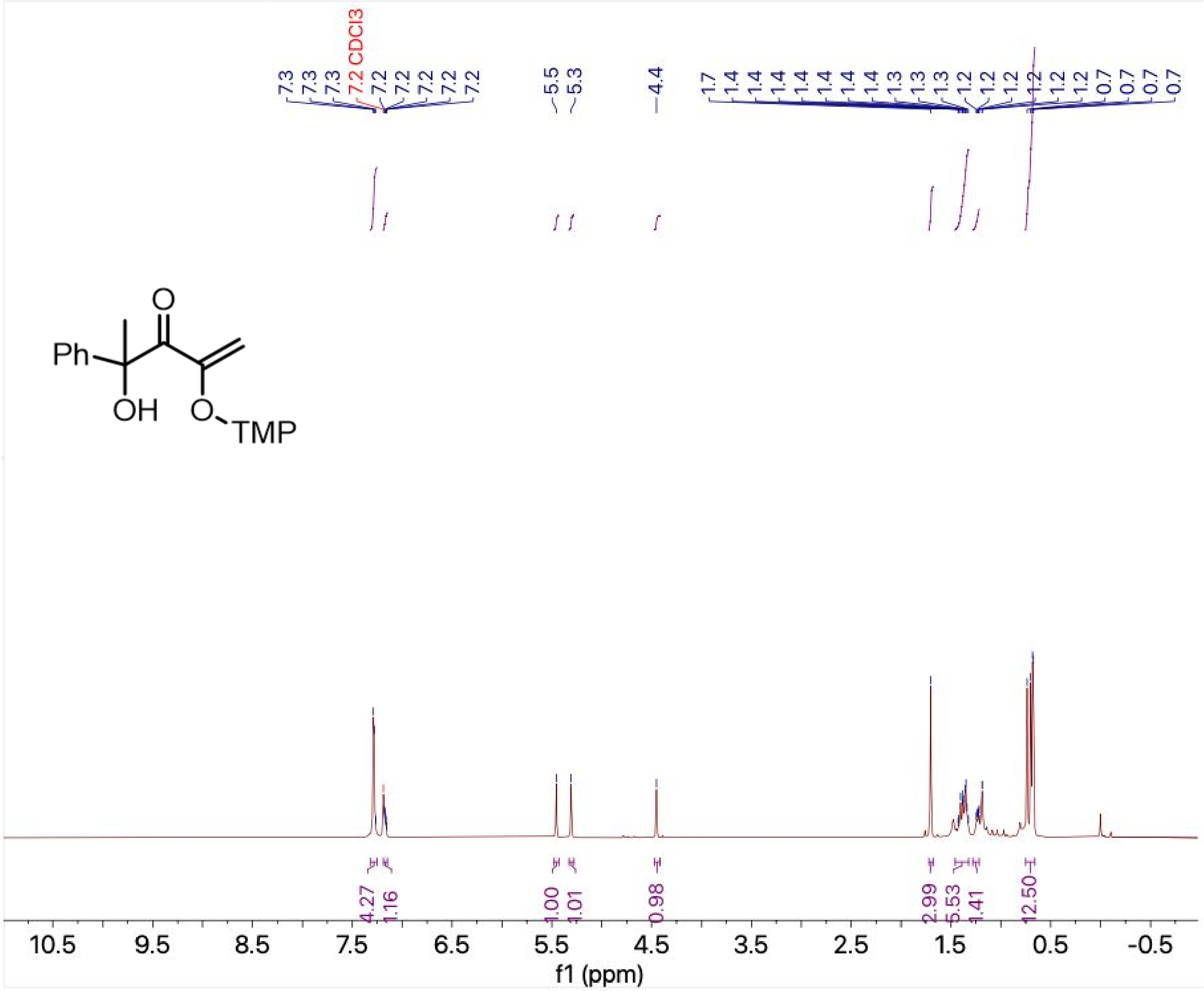

$^{13}C\{^1H\}$ **500 MHz NMR for Compound 4c.**

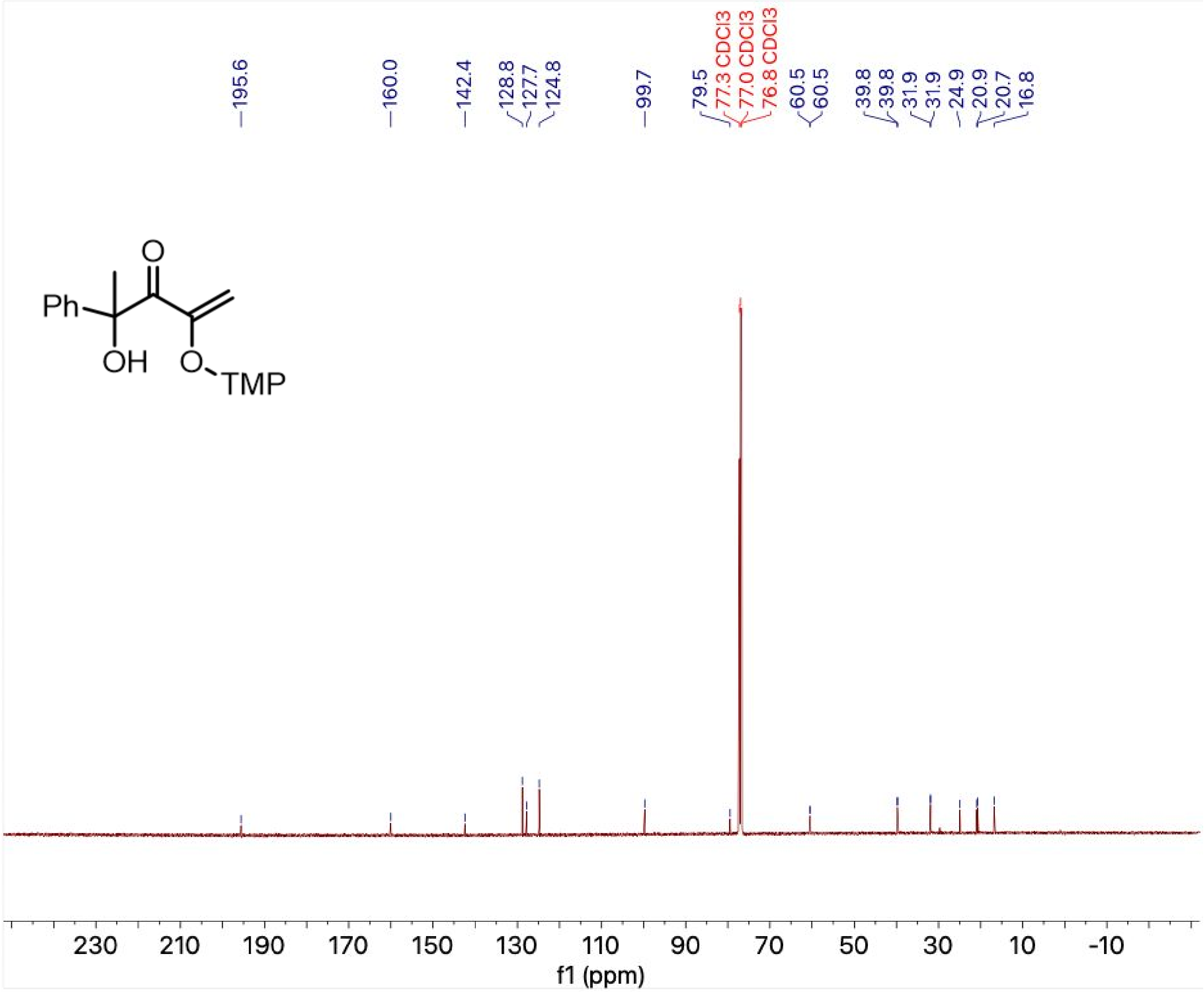

$^1H\{^{13}C\}$ 500 MHz NMR for Compound 4d.

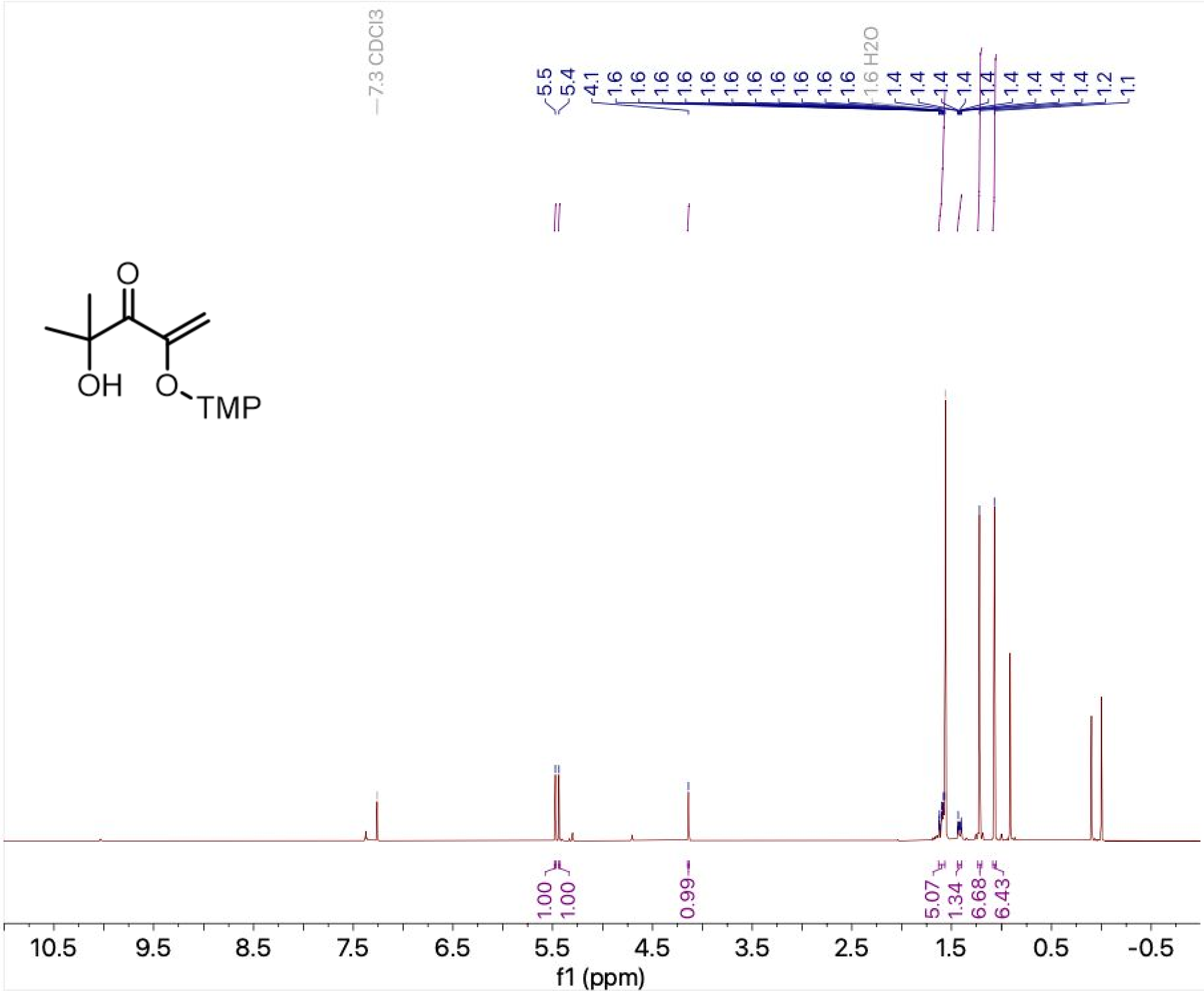

$^{13}C\{^1H\}$ **500 MHz NMR for Compound 4d.**

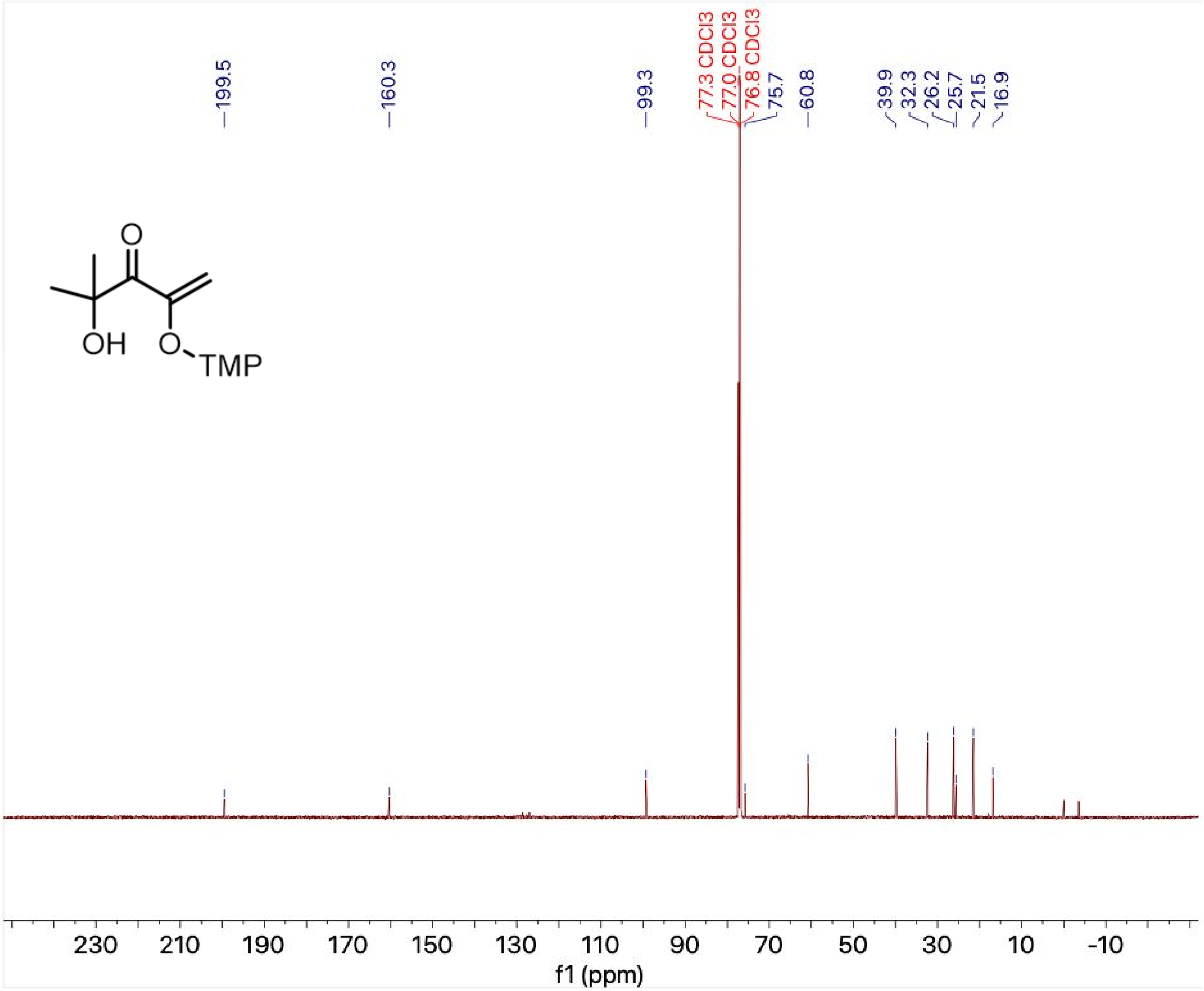

^{1}H{^{13}C} 500 MHz NMR for Compound 4e.

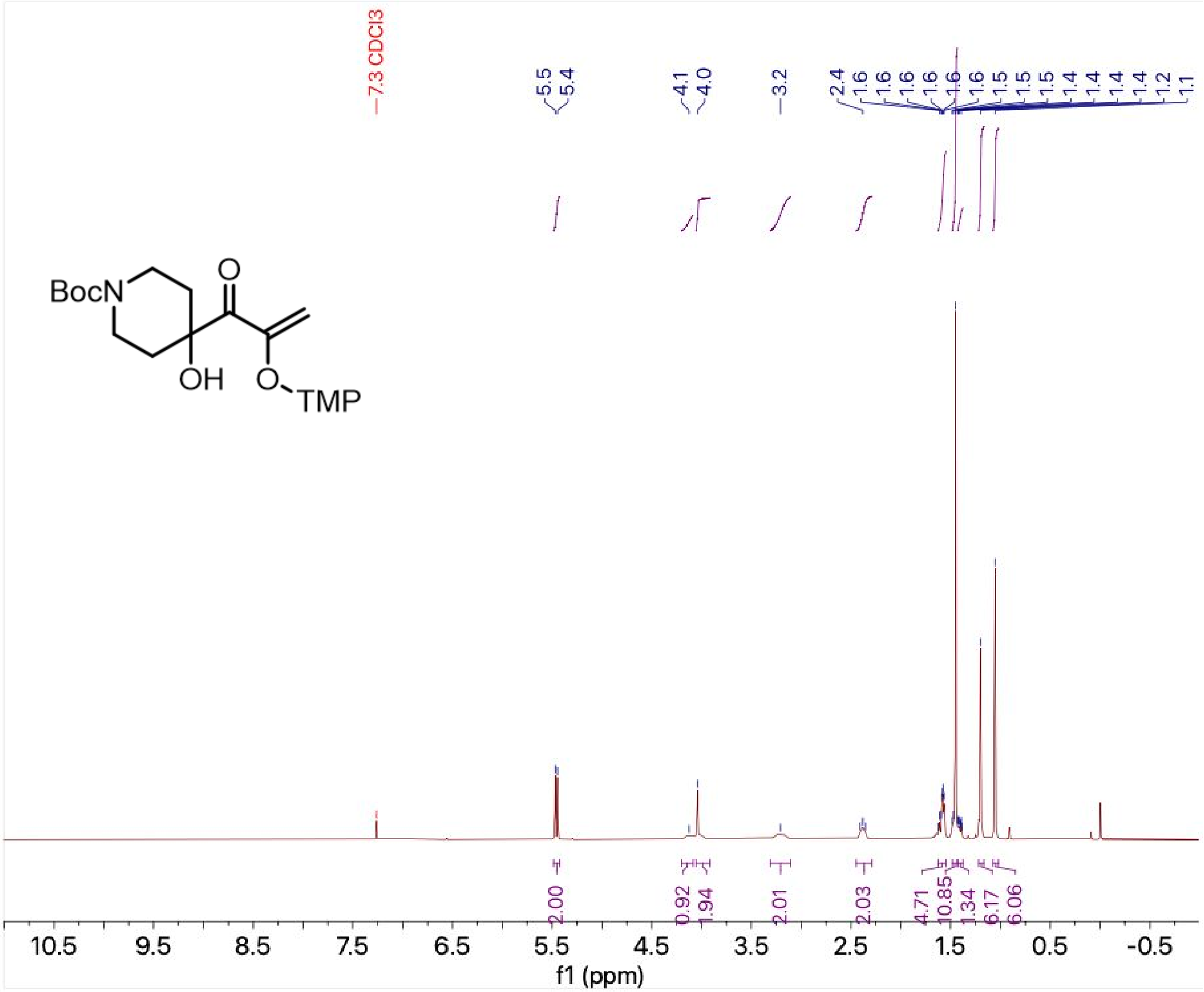

$^{13}C\{^{1}H\}$ 500 MHz NMR for Compound 4e.

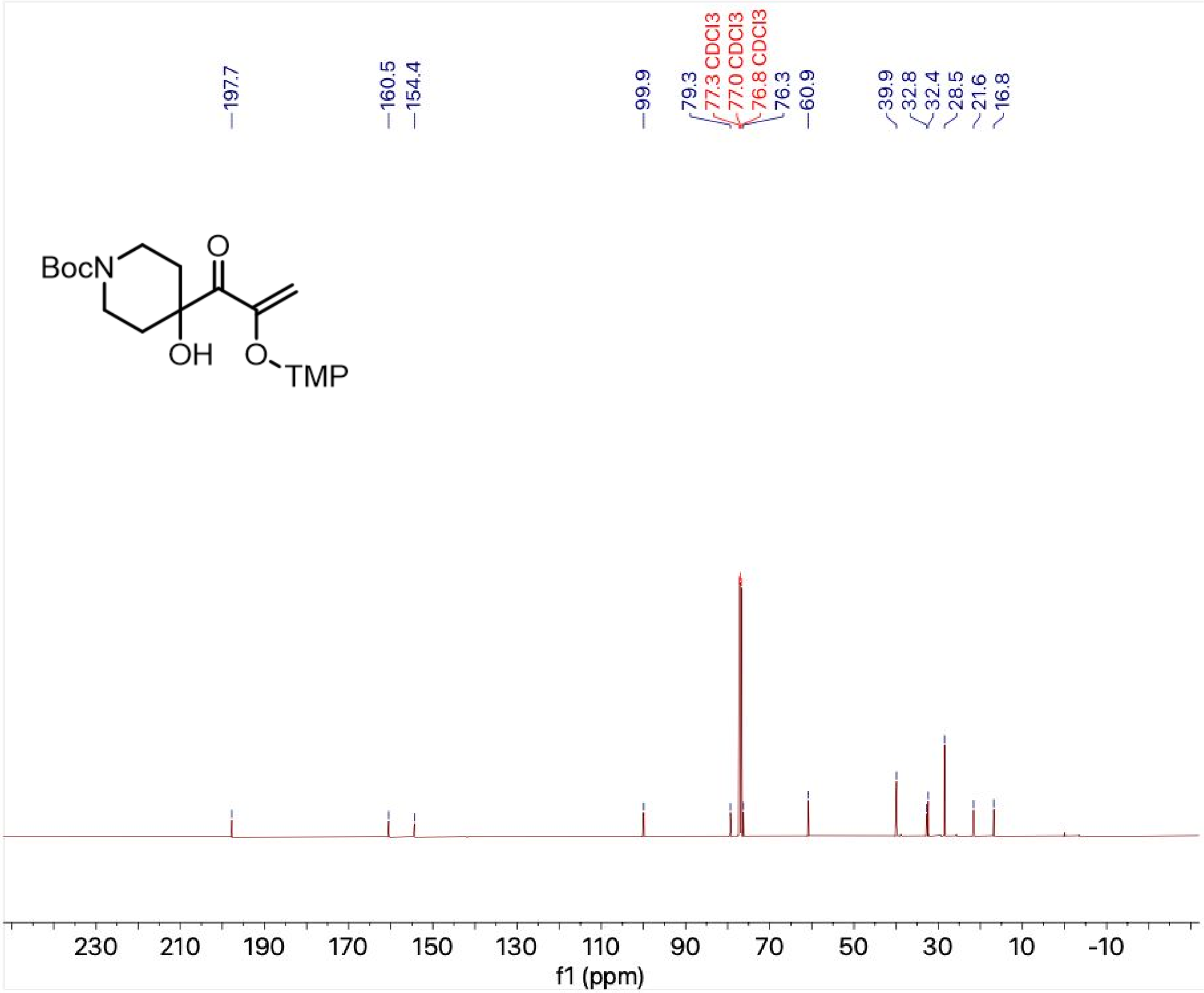

$^1H\{^{13}C\}$ **500 MHz NMR for Compound 4f.**

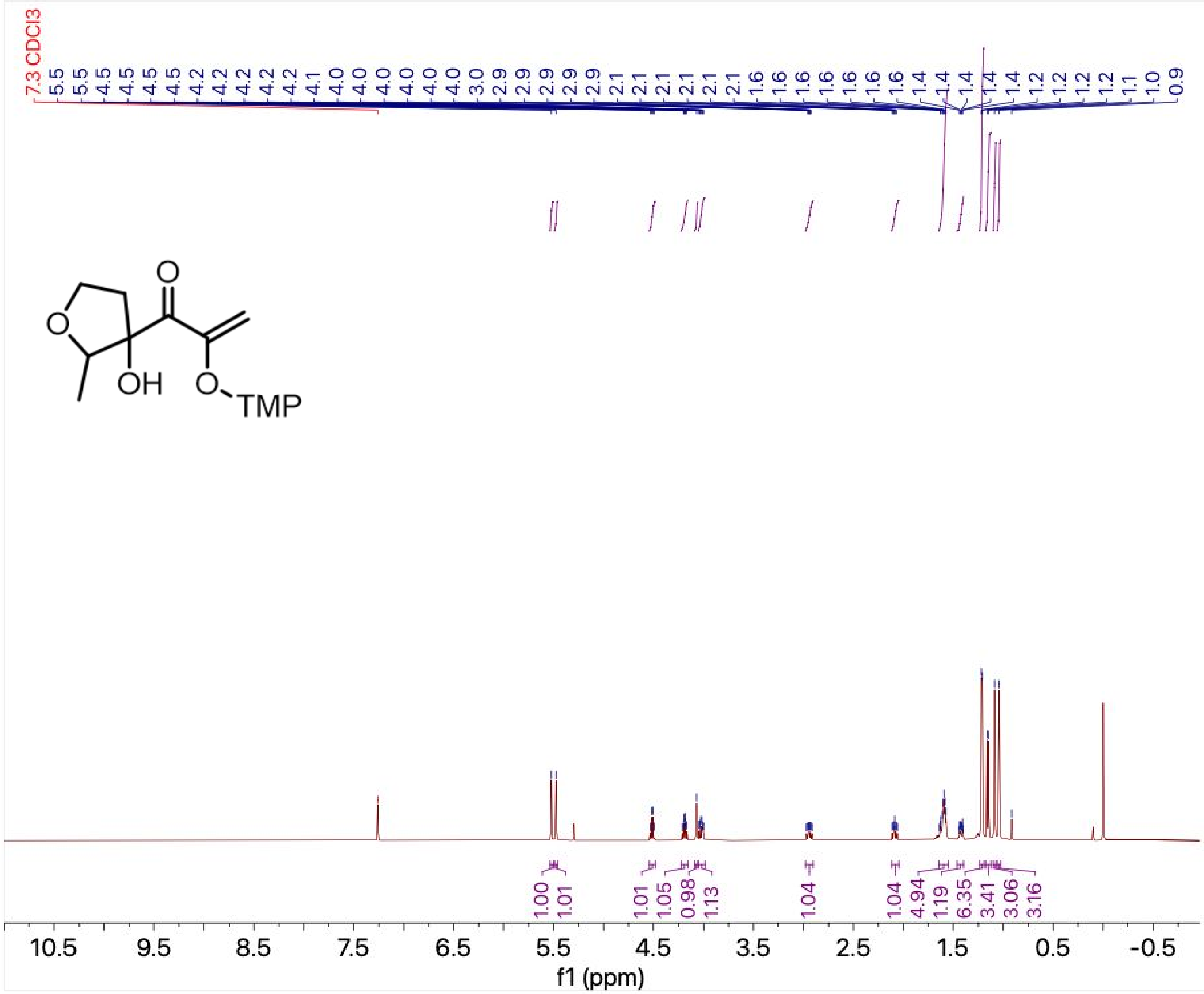

$^{13}C\{^1H\}$ 500 MHz NMR for Compound 4f.

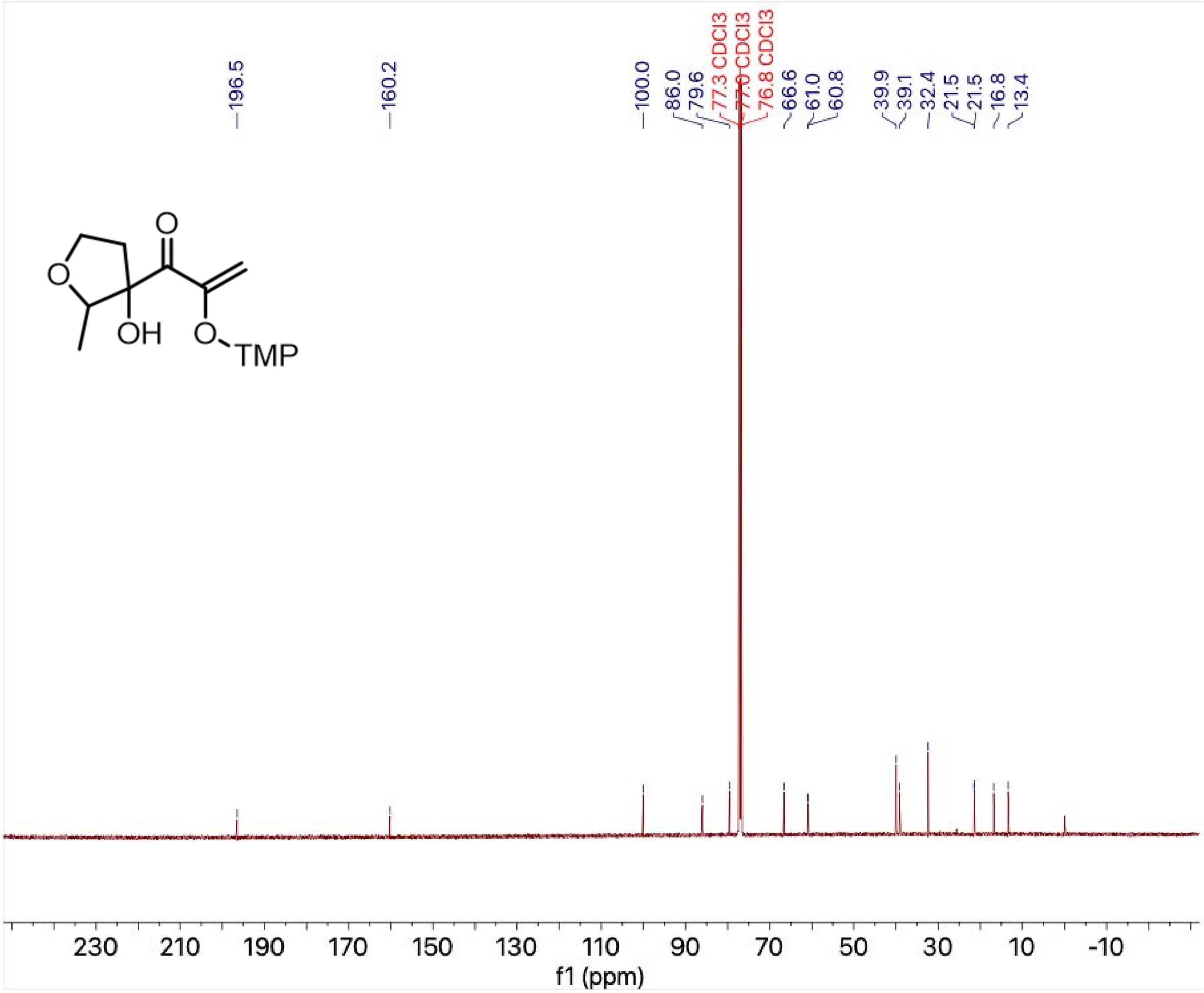

$^{1}H\{^{13}C\}$ **500 MHz NMR for Compound 4g.**

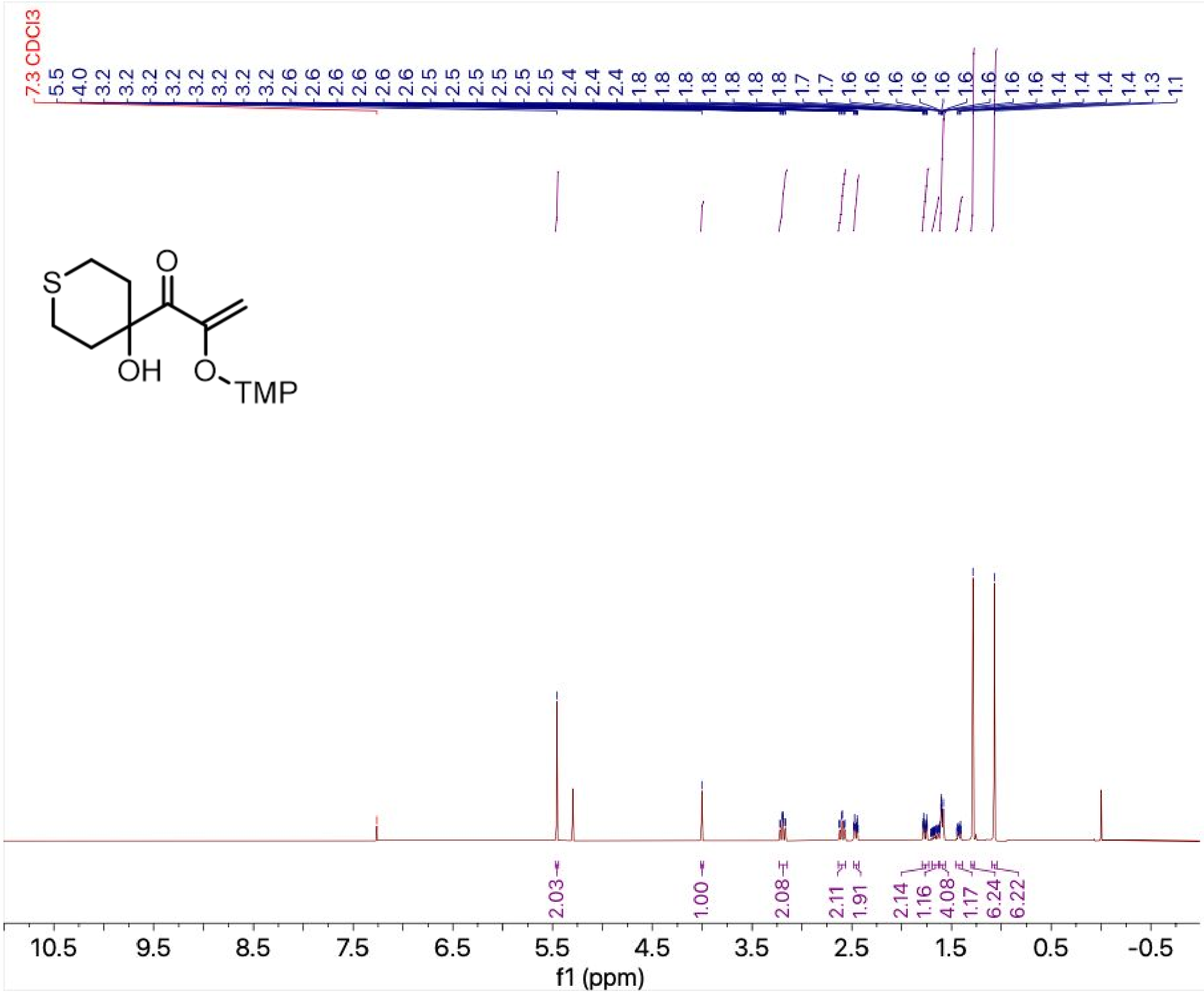

$^{13}C\{^1H\}$ **500 MHz NMR for Compound 4g.**

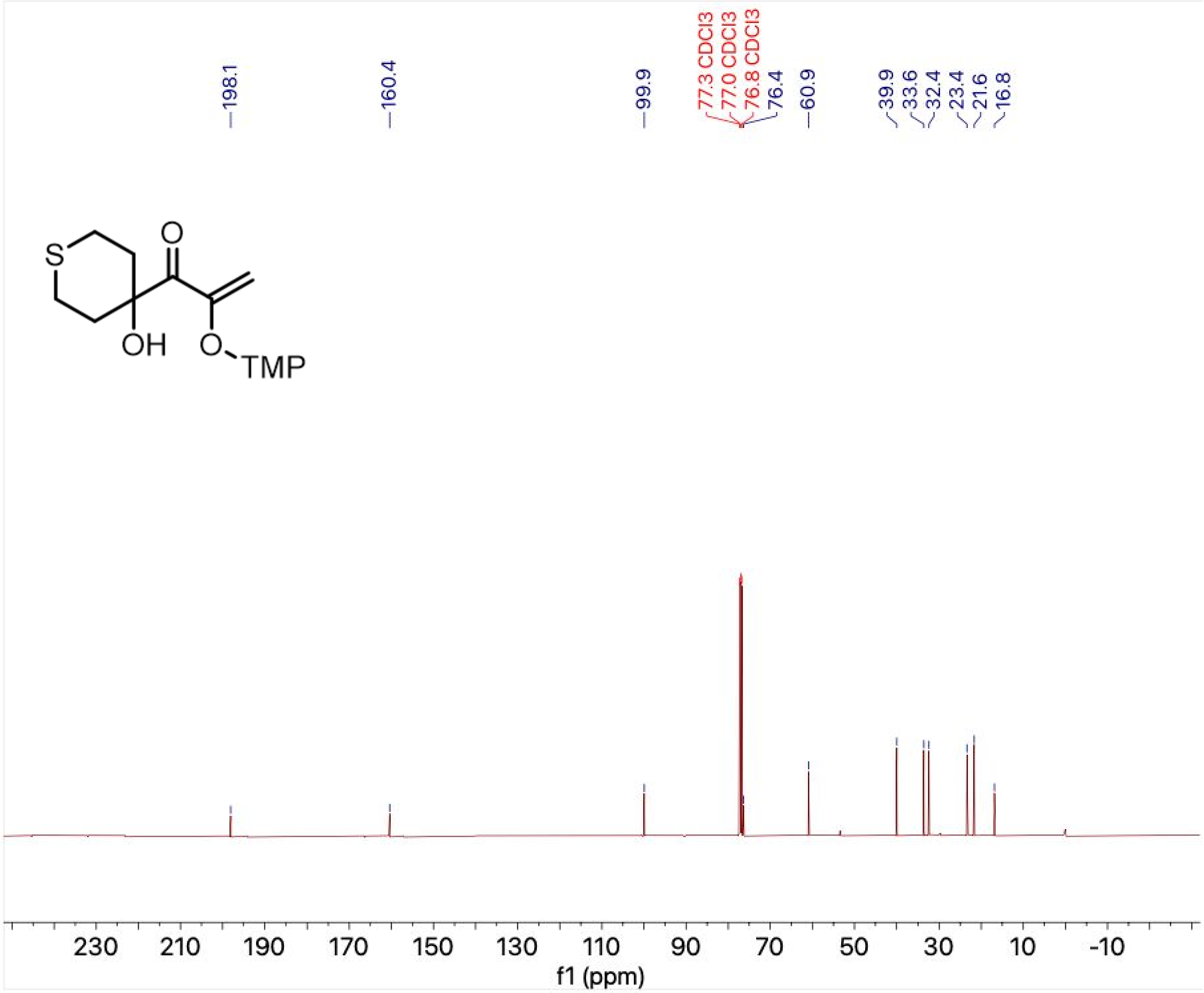

$^{1}H\{^{13}C\}$ 500 MHz NMR for Compound 4h.

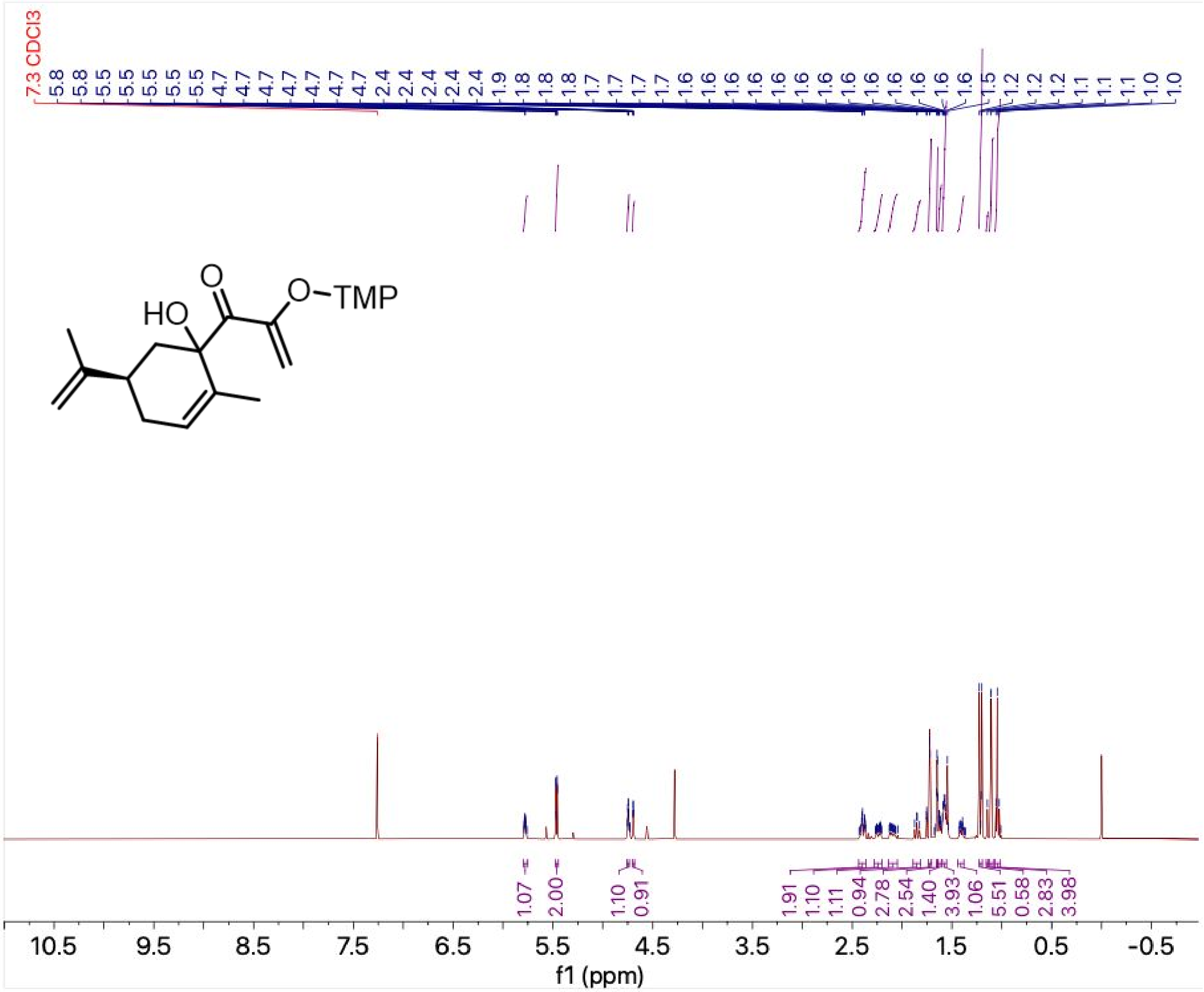

$^{13}C\{^{1}H\}$ 500 MHz NMR for Compound 4h.

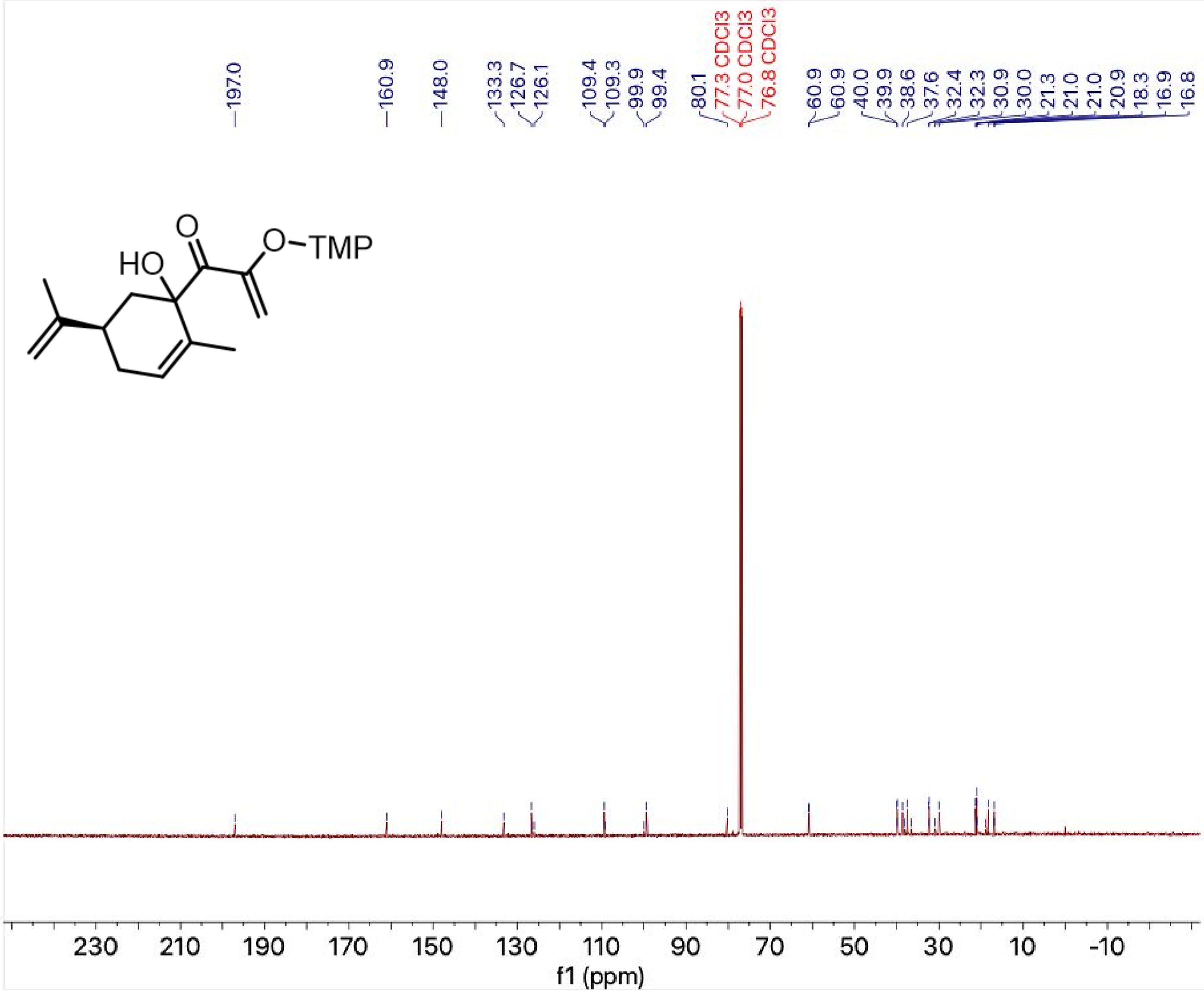

$^{1}H\{^{13}C\}$ 500 MHz NMR for Compound 4i.

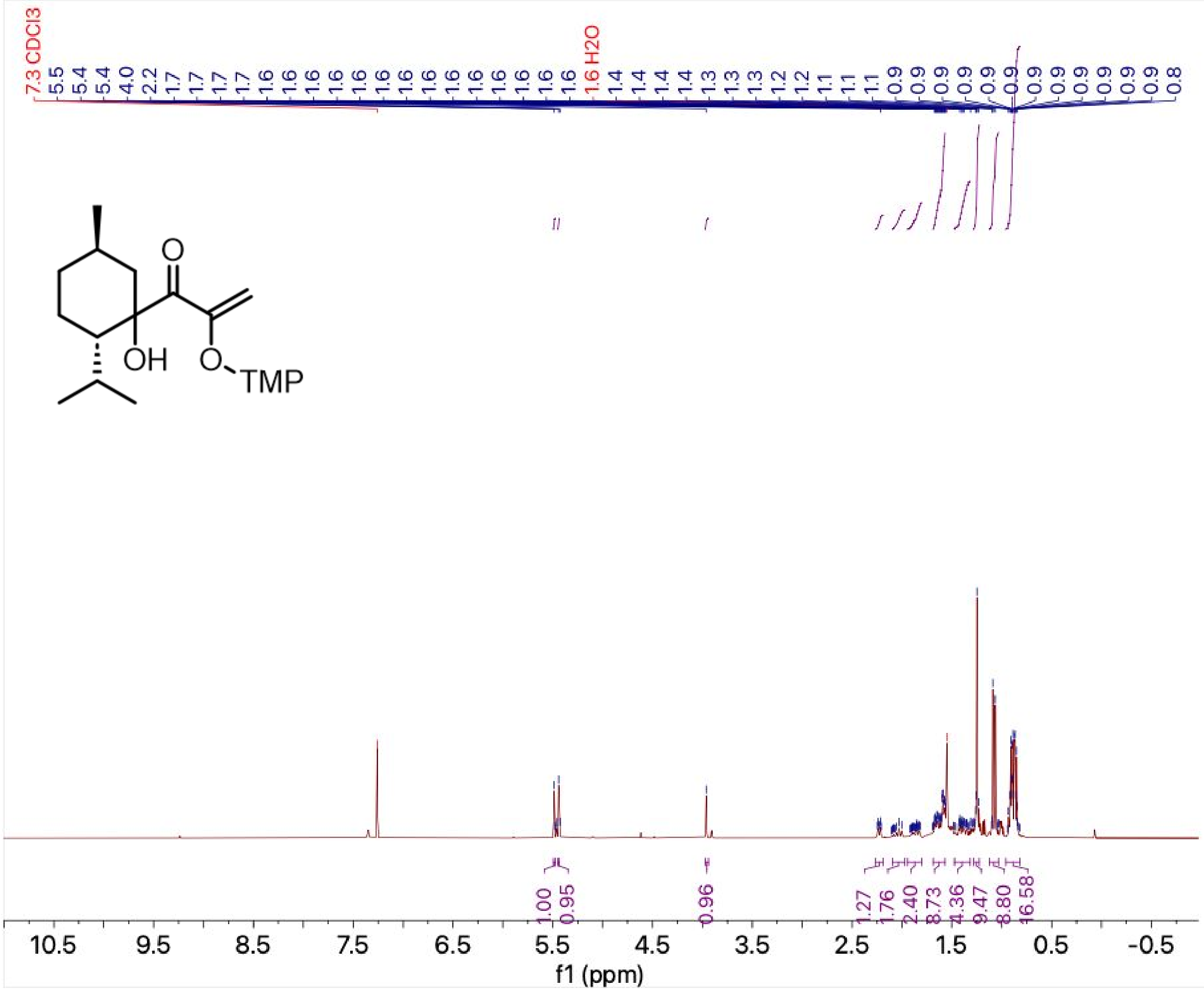

$^{13}C\{^1H\}$ 500 MHz NMR for Compound 4i.

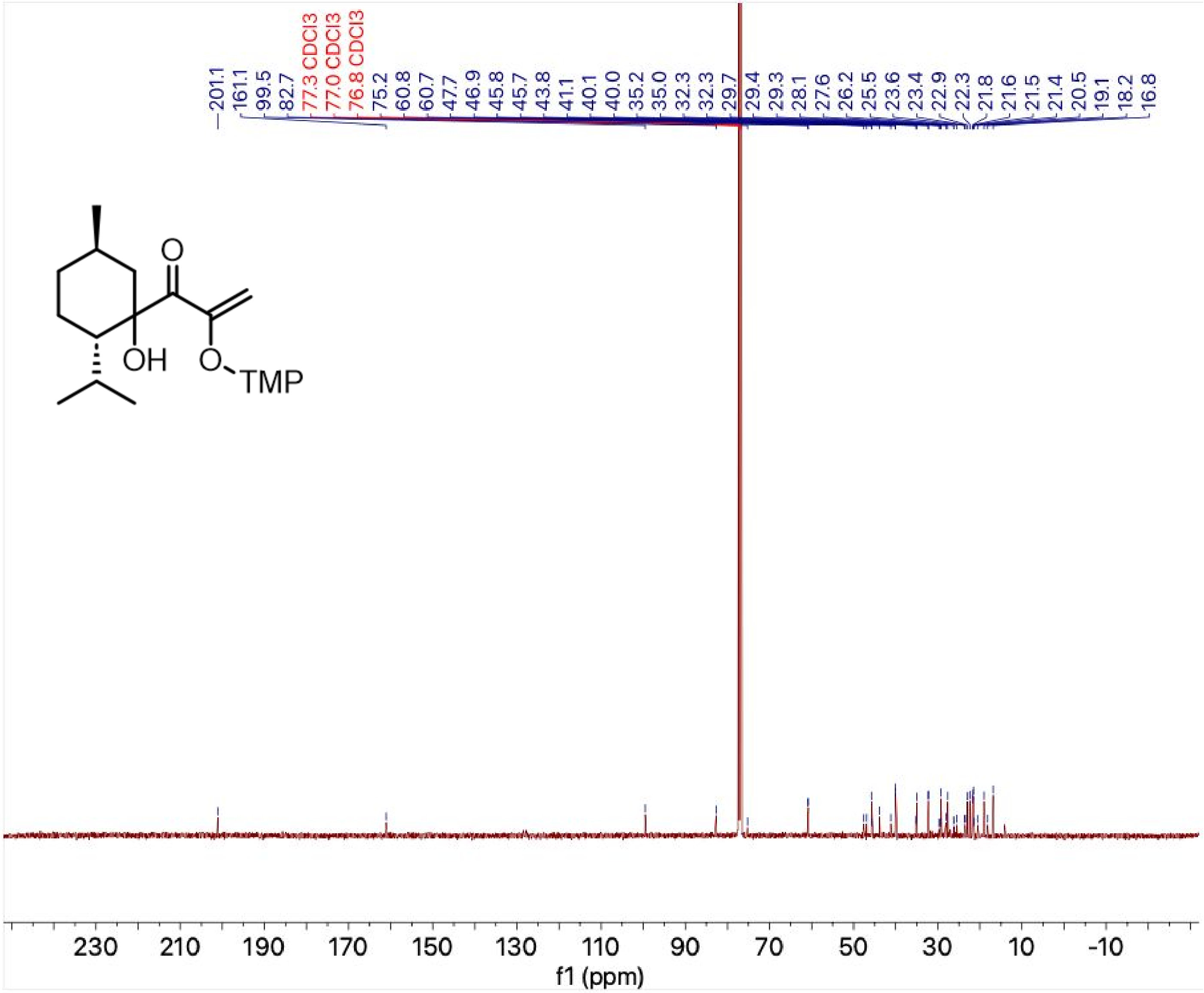

$^{1}H\{^{13}C\}$ **500 MHz NMR for Compound 4j.**

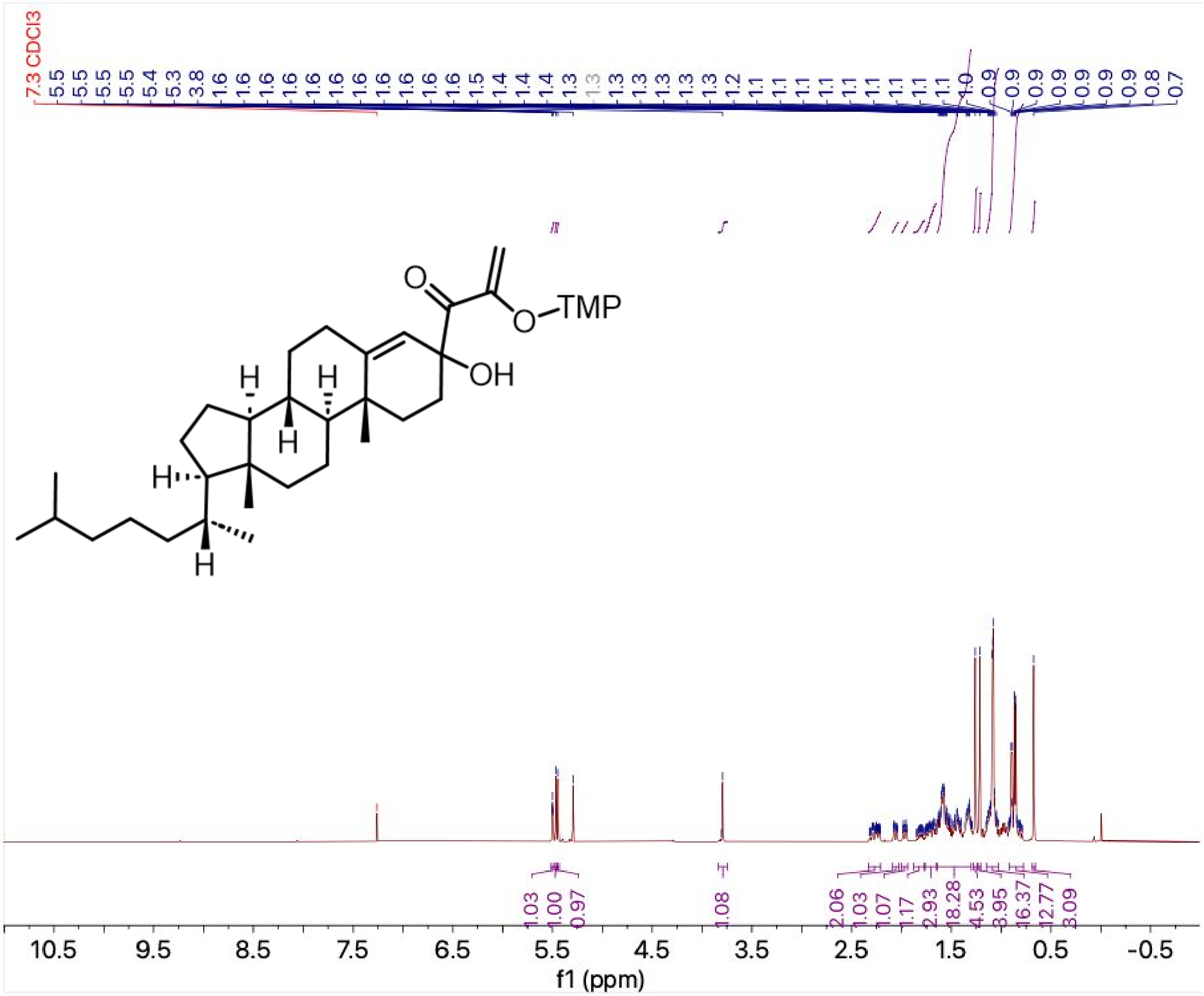

$^{13}C\{^1H\}$ 500 MHz NMR for Compound 4j.

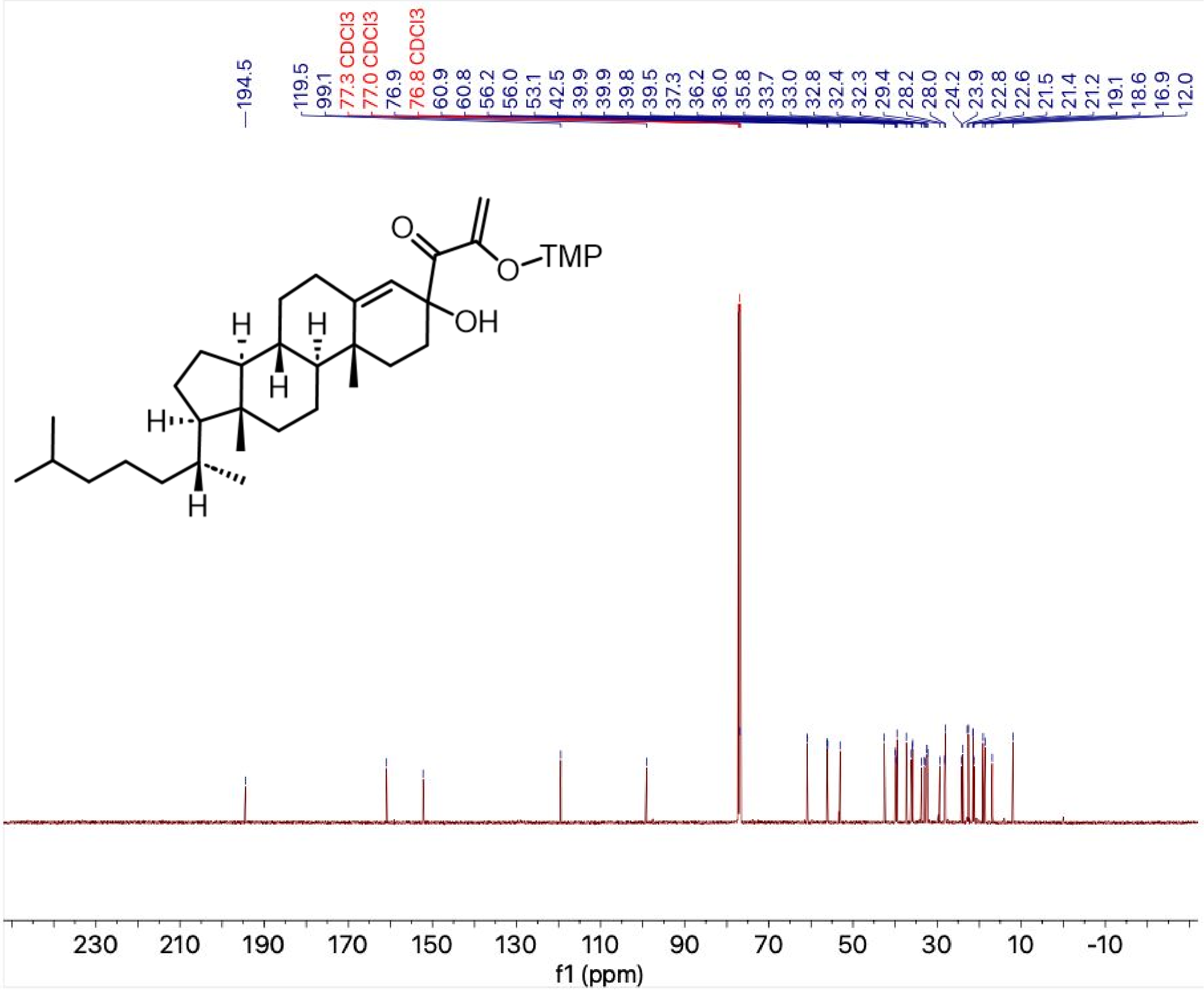

$^{1}H\{^{13}C\}$ 500 MHz NMR for Compound 4k.

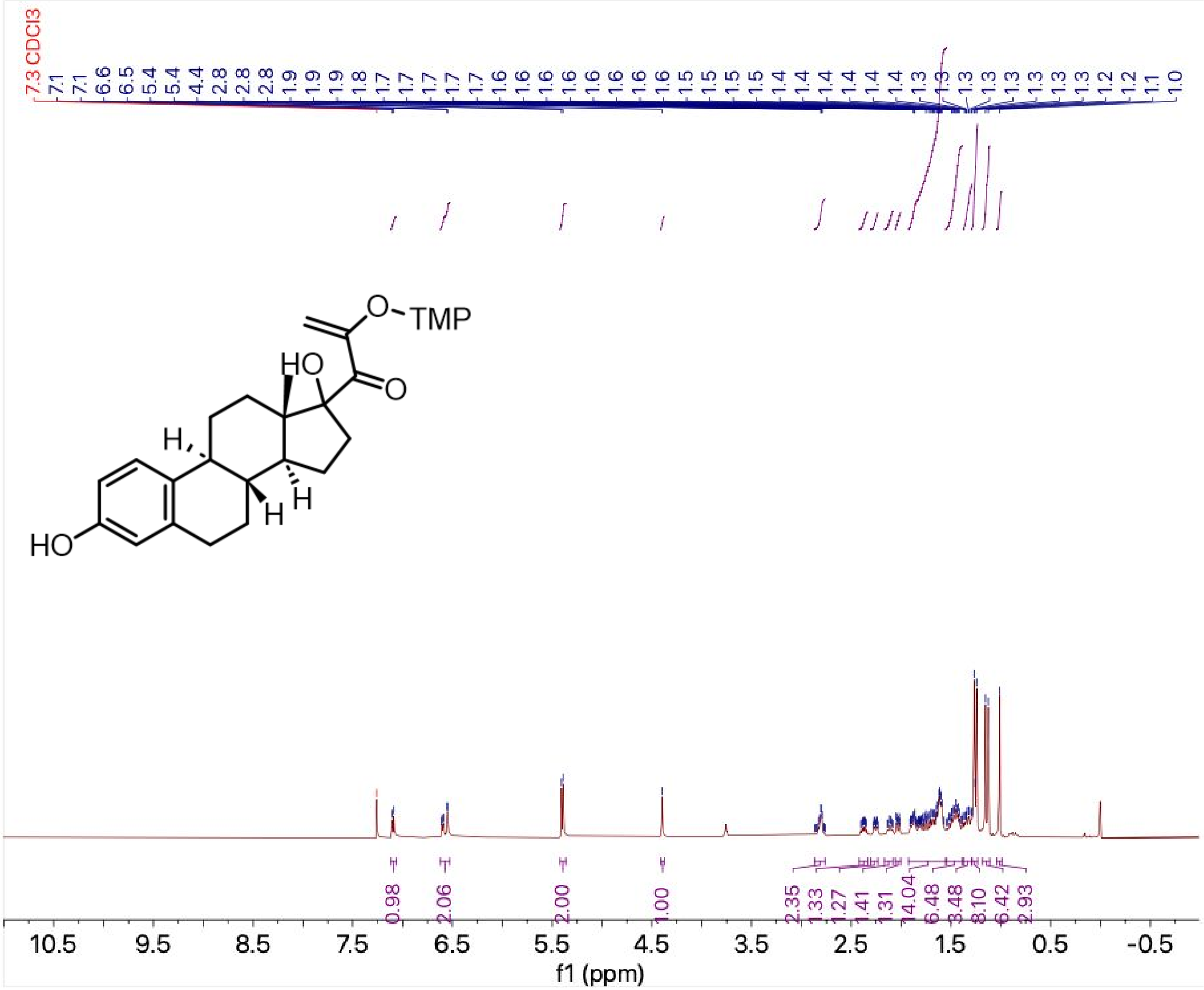

$^{13}C\{^{1}H\}$ **500 MHz NMR for Compound 4k.**

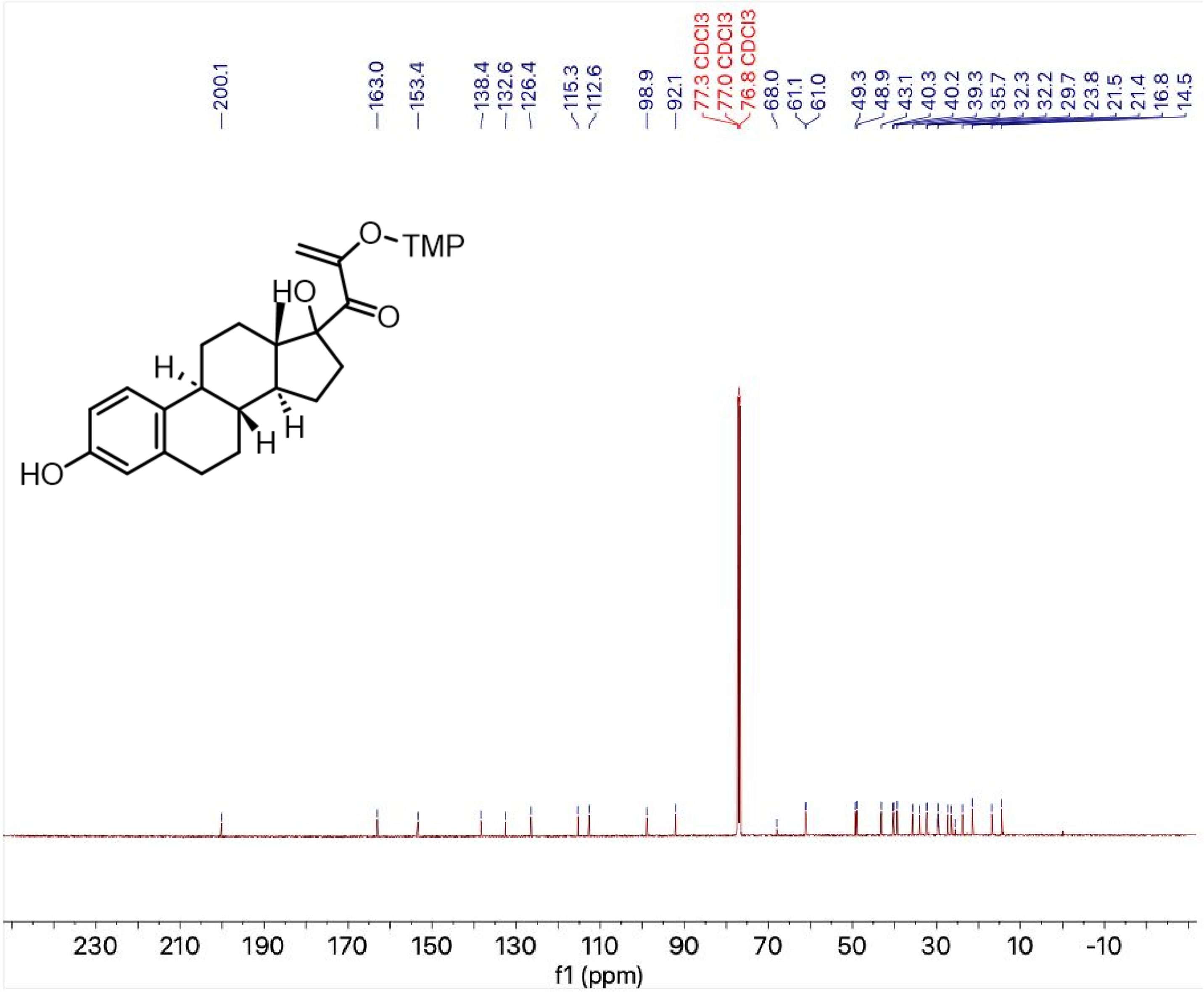

^{1}H{^{13}C} 500 MHz NMR for Compound 5l.

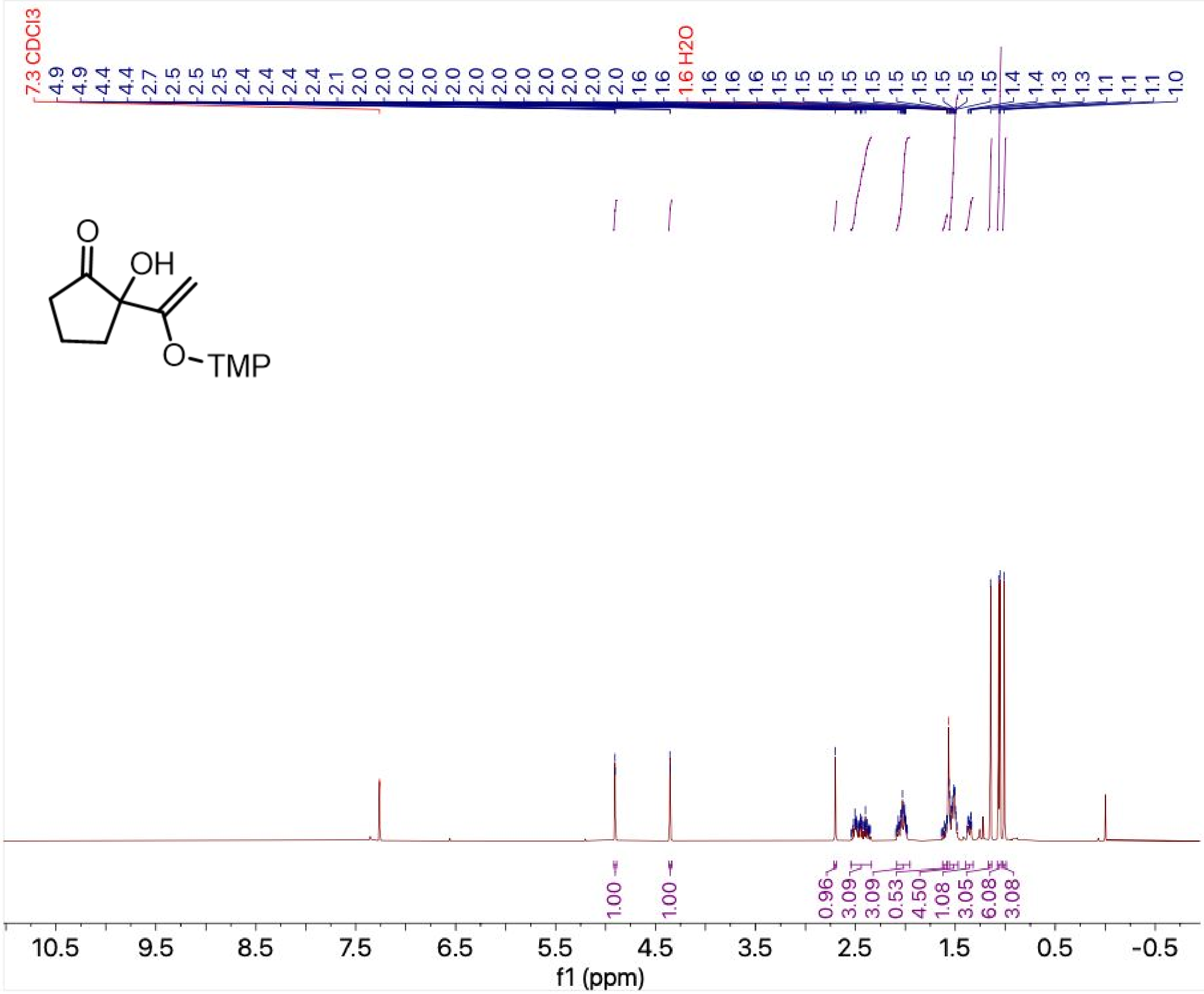

$^{13}C\{^{1}H\}$ 500 MHz NMR for Compound 5l.

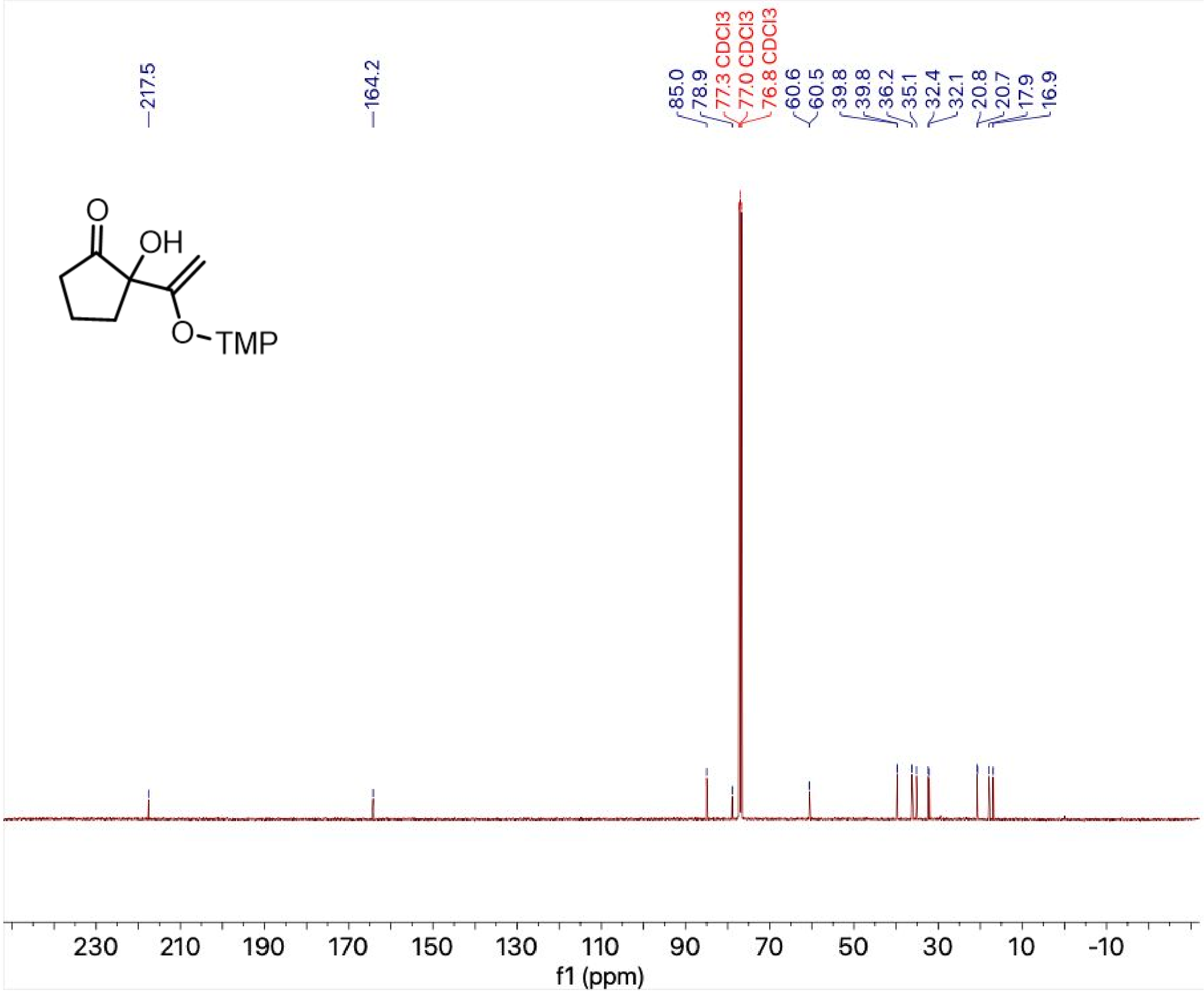

$^{1}H\{^{13}C\}$ 500 MHz NMR for Compound 5m.

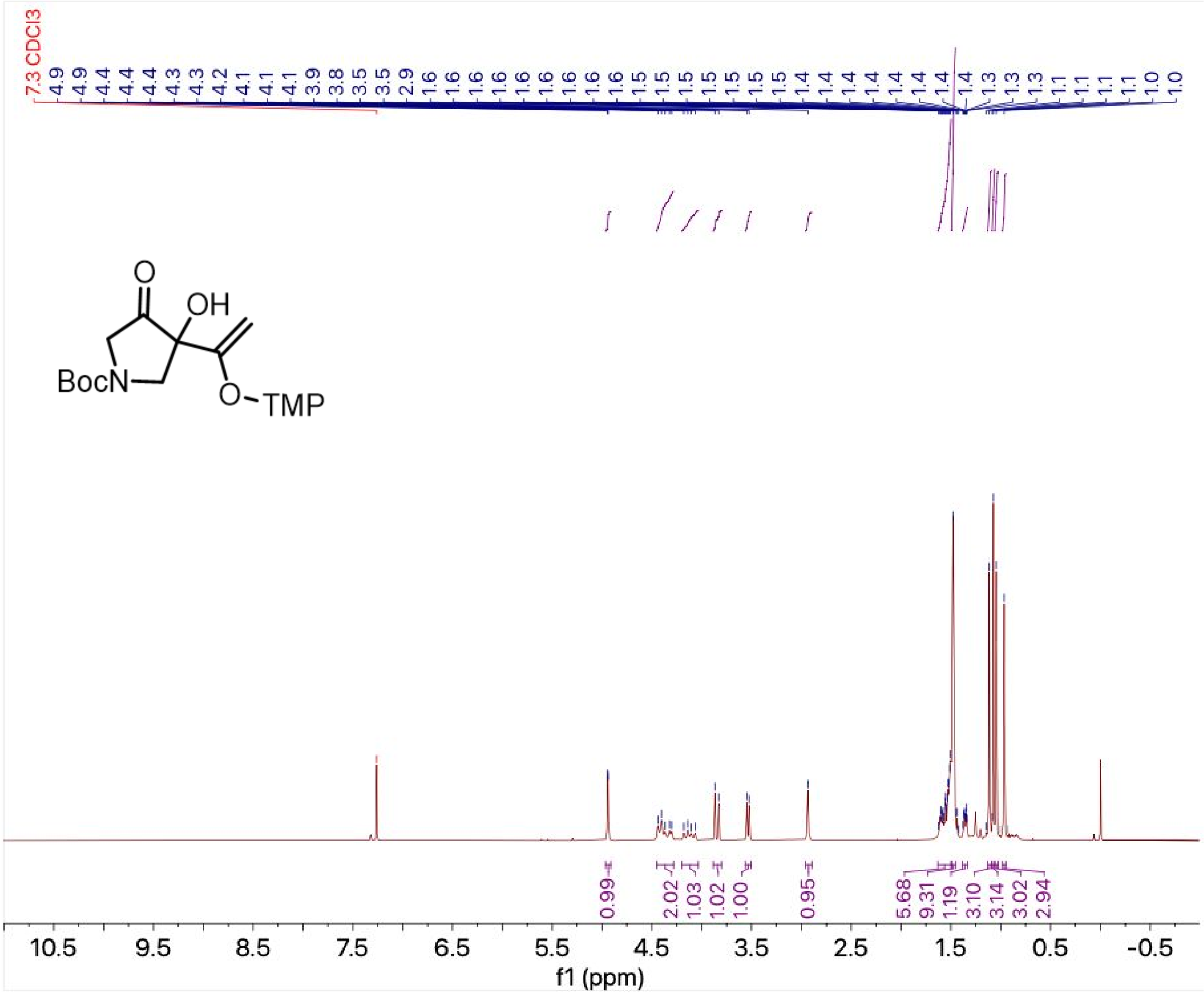

$^{13}C\{^{1}H\}$ 500 MHz NMR for Compound 5m.

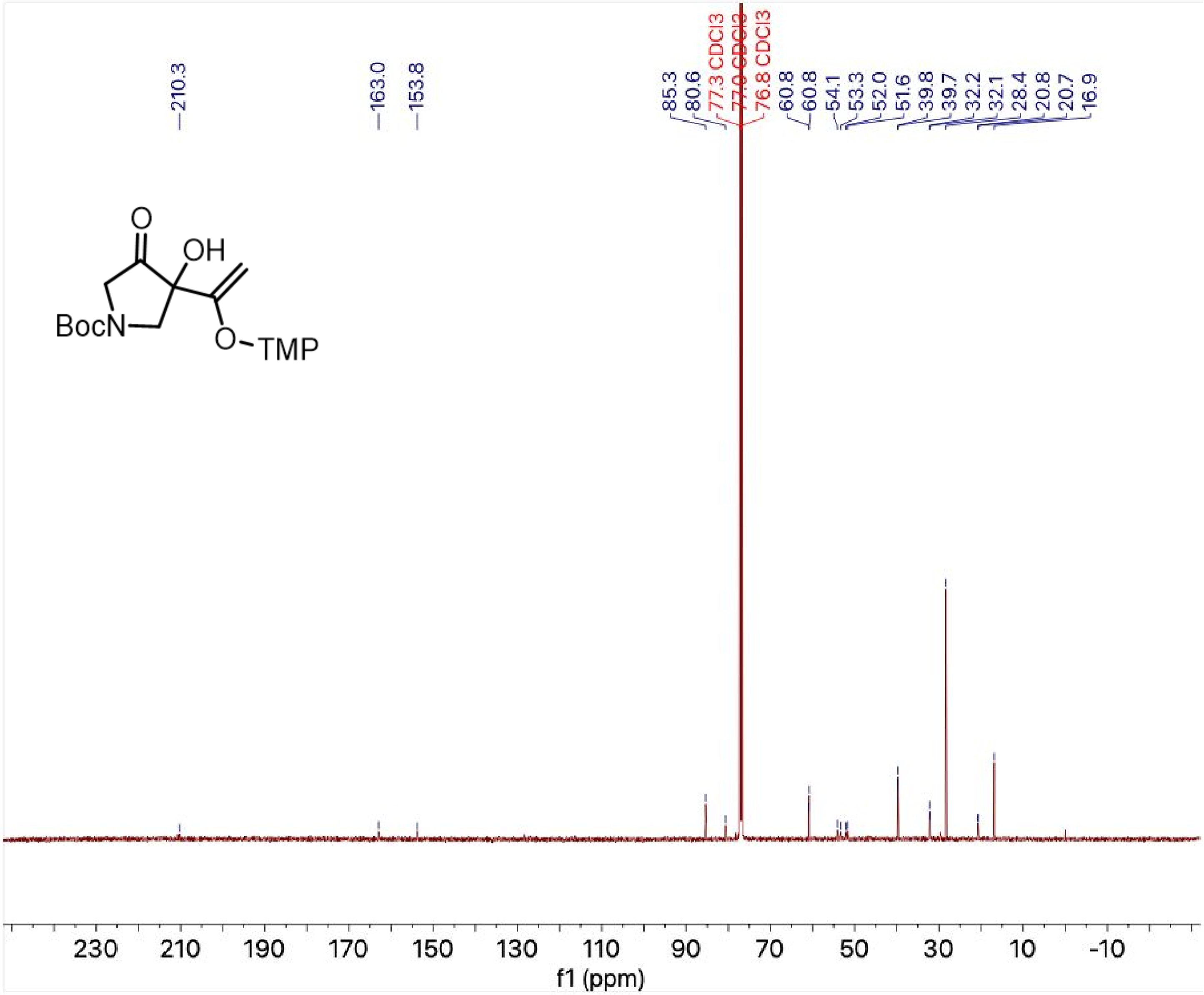

^{1}H{^{13}C} 500 MHz NMR for Compound 5n.

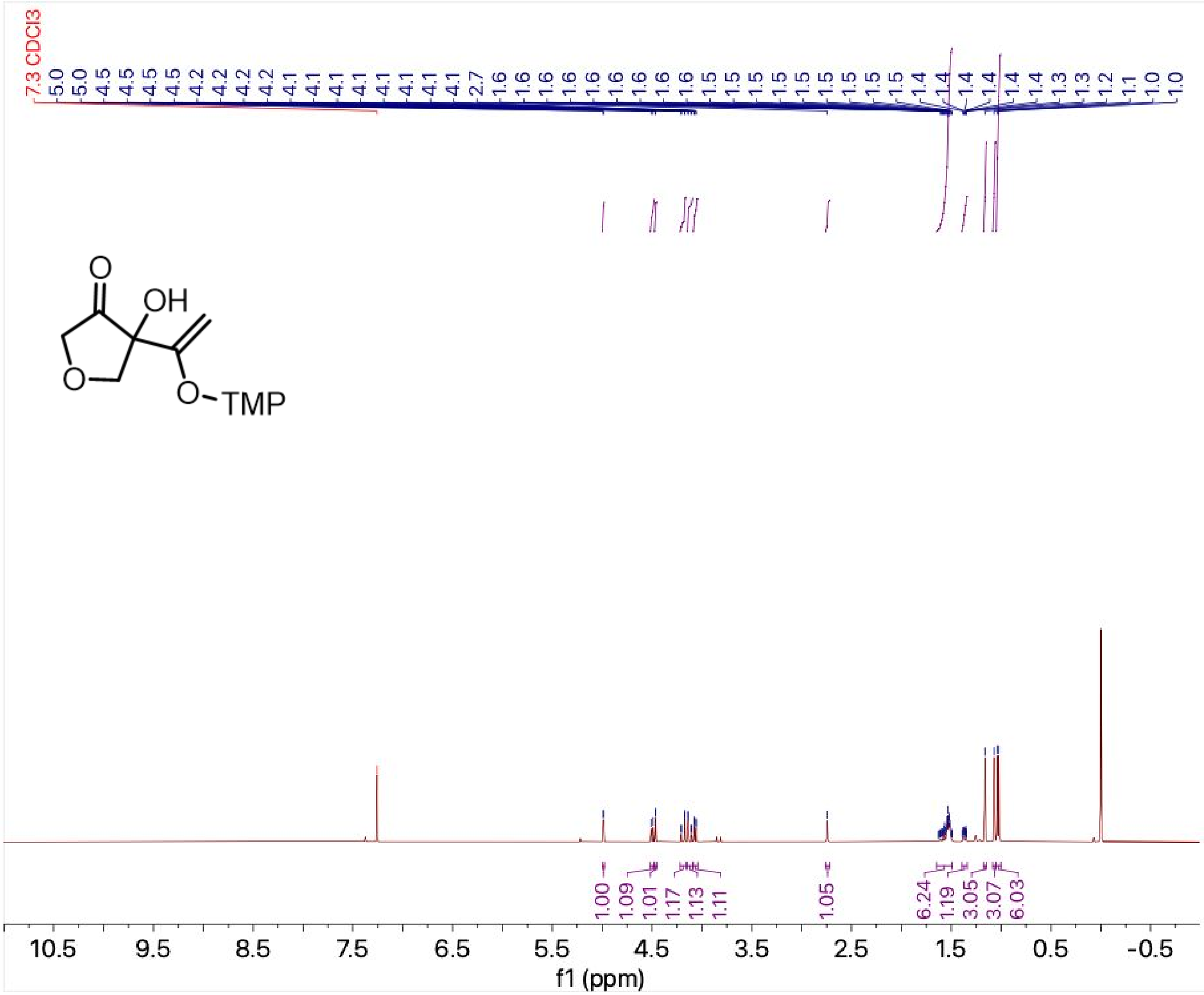

$^{13}C\{^1H\}$ 500 MHz NMR for Compound 5n.

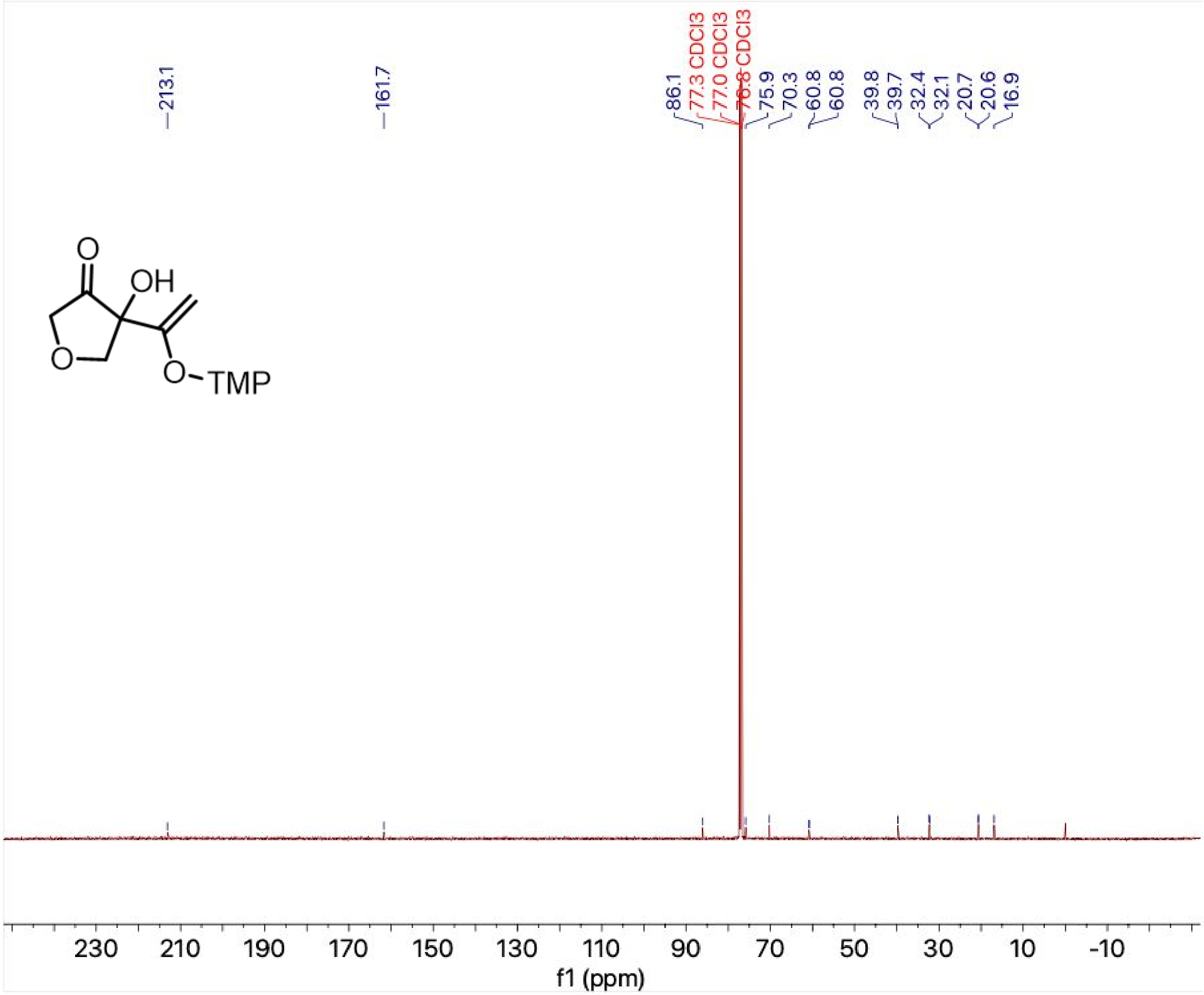

$^{1}H\{^{13}C\}$ **500 MHz NMR for Compound 4o.**

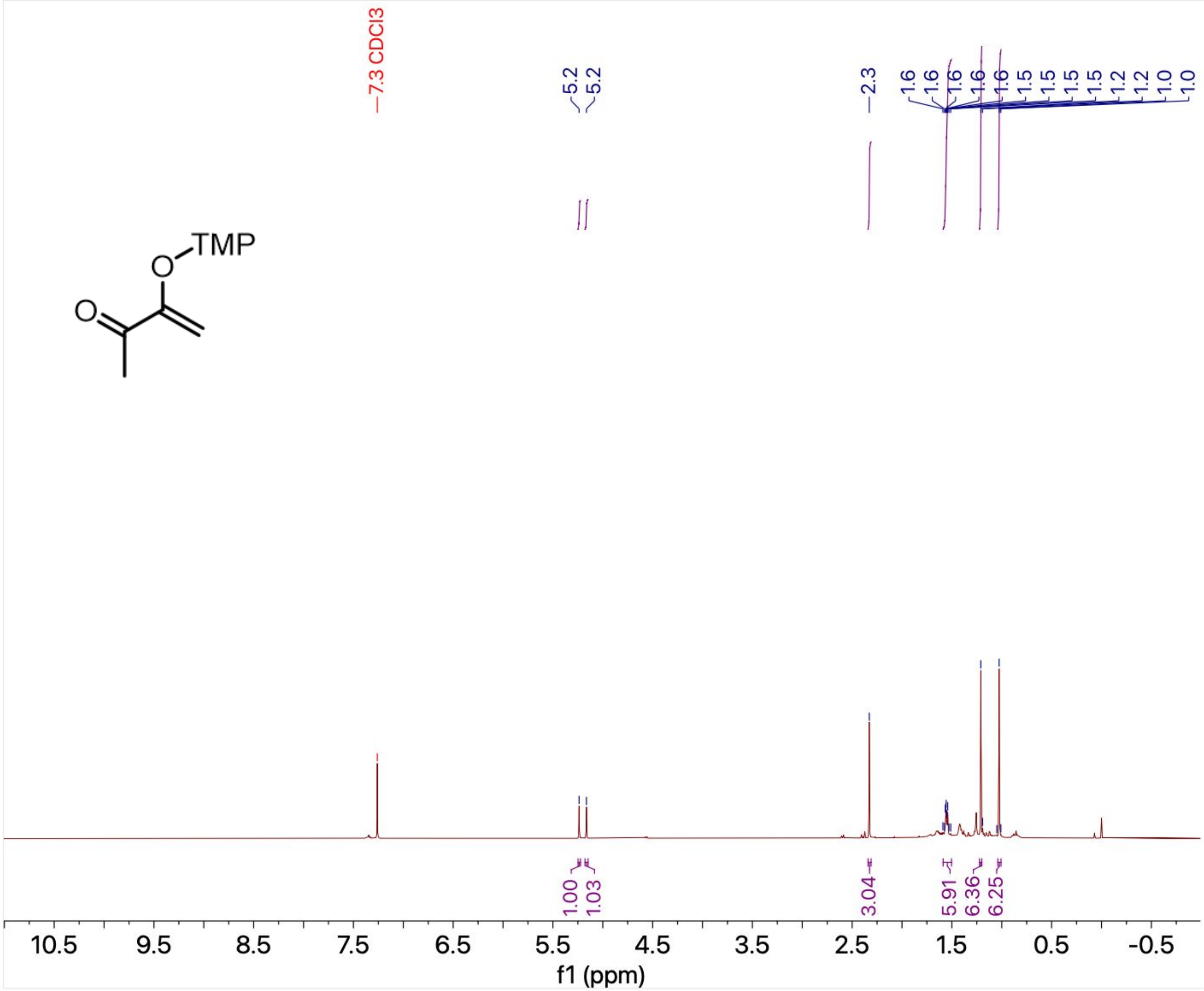

$^{13}C\{^{1}H\}$ **500 MHz NMR for Compound 4o.**

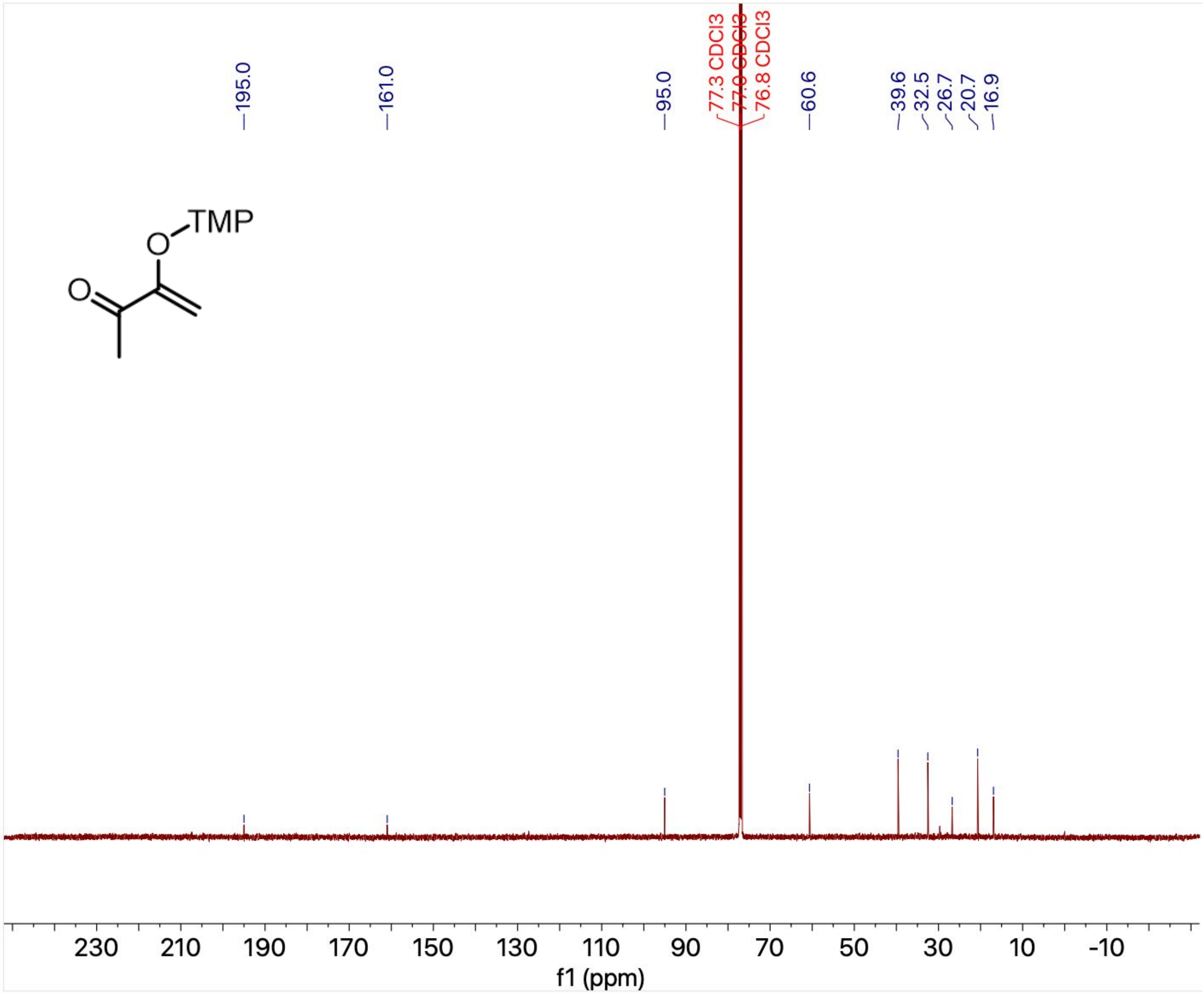

$^{1}H\{^{13}C\}$ **500 MHz NMR for Compound 4p.**

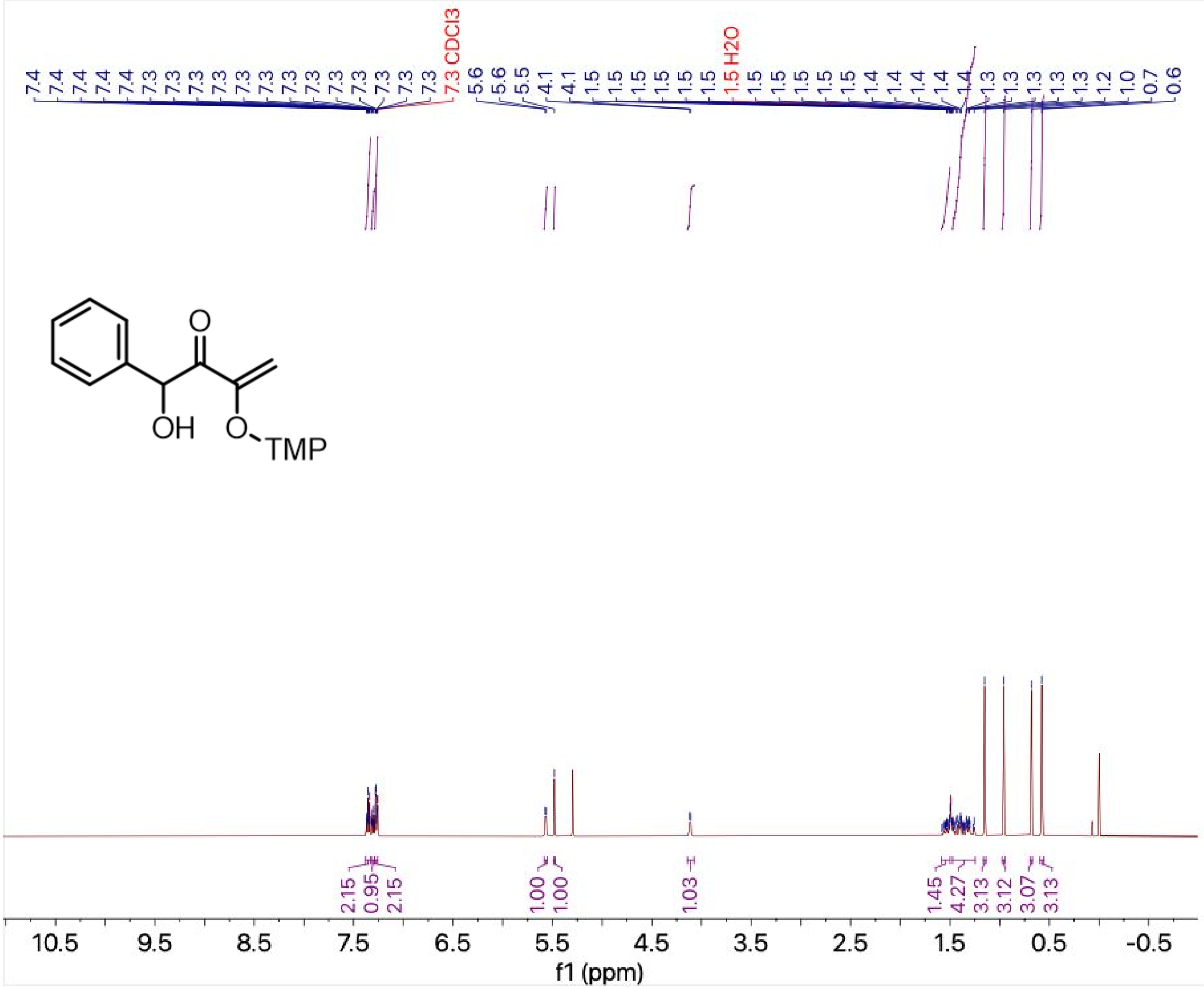

$^{13}C\{^{1}H\}$ 500 MHz NMR for Compound 4p.

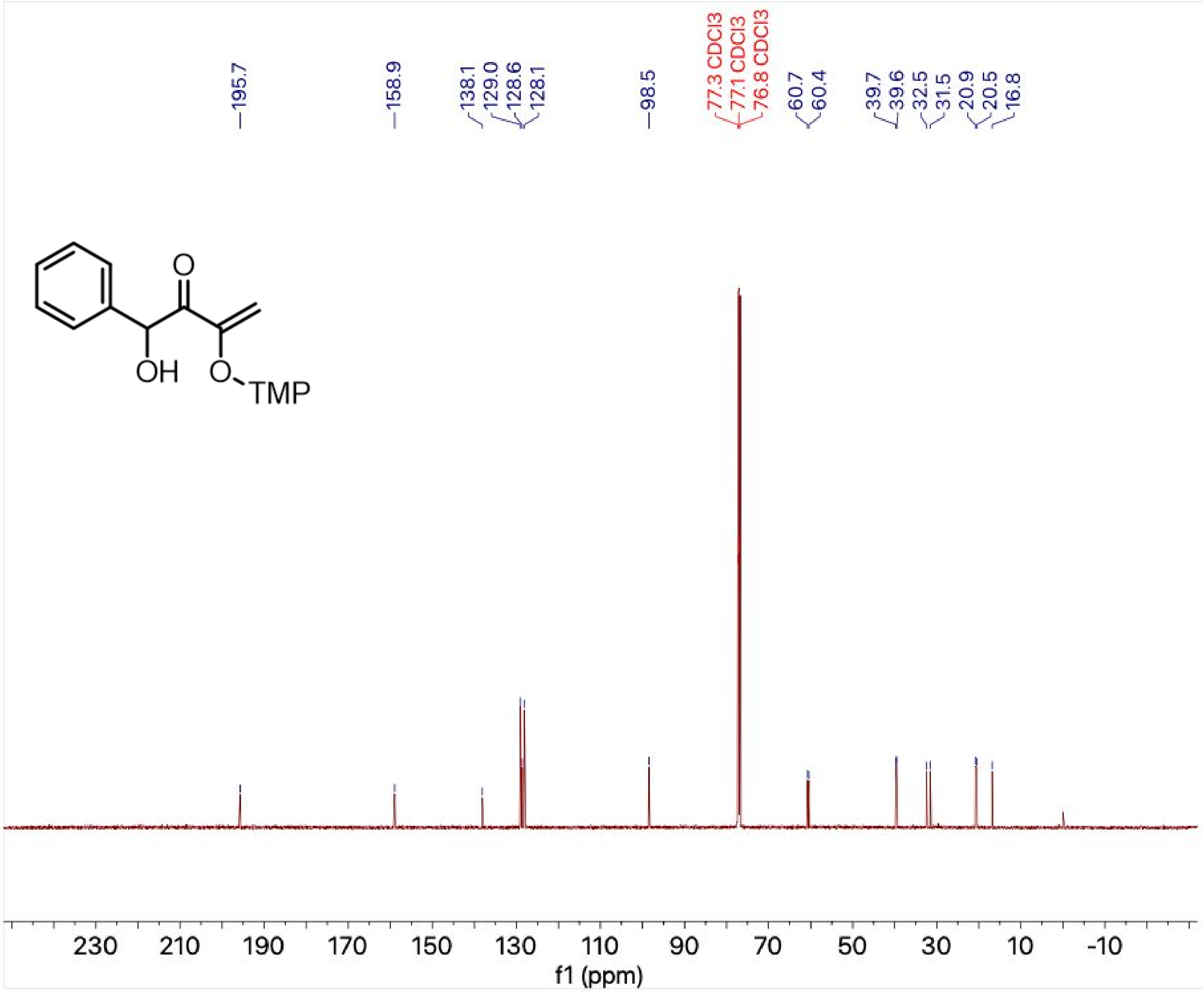

$^{1}H\{^{13}C\}$ 500 MHz NMR for Compound 4q.

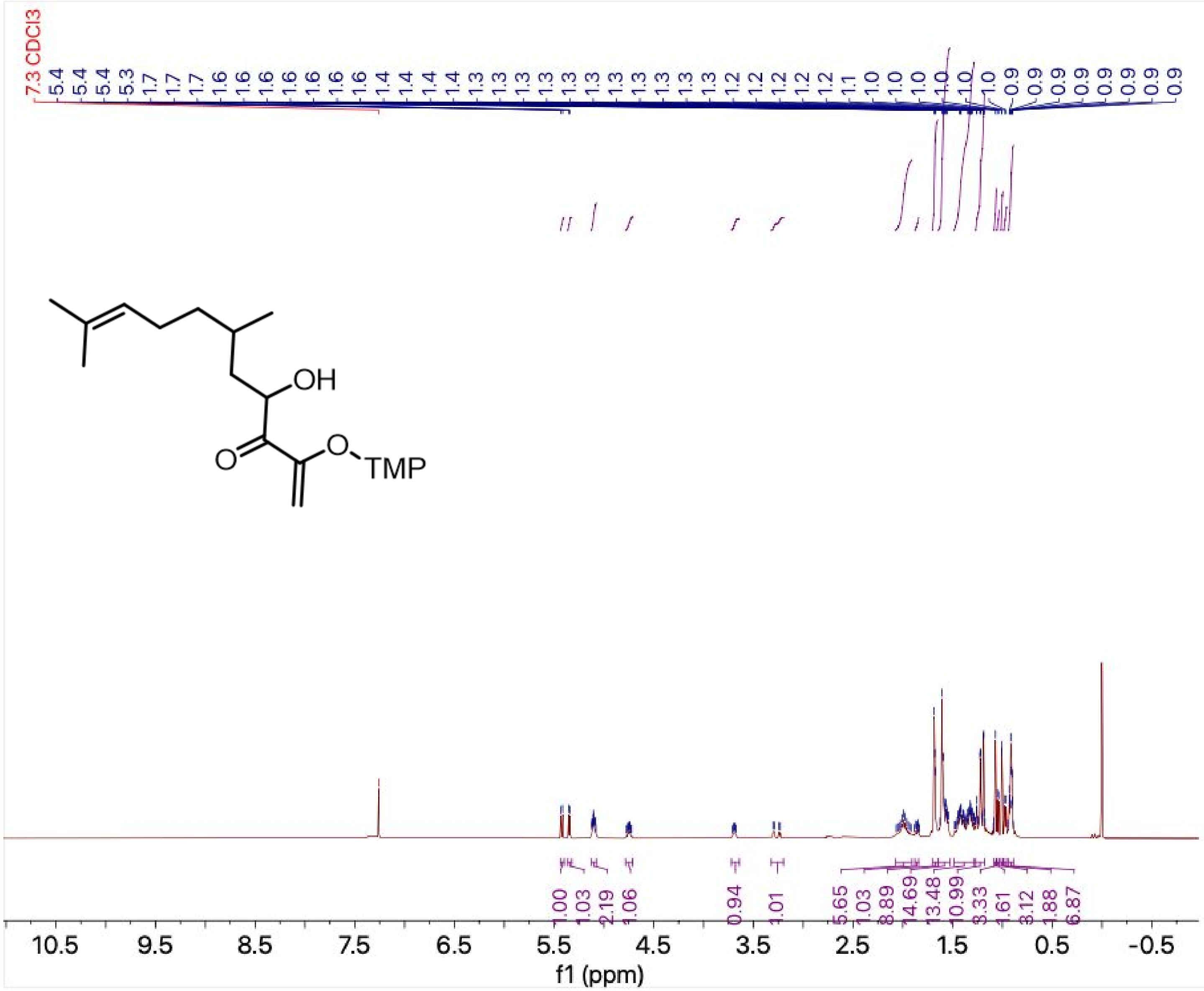

$^{13}C\{^1H\}$ 500 MHz NMR for Compound 4q.

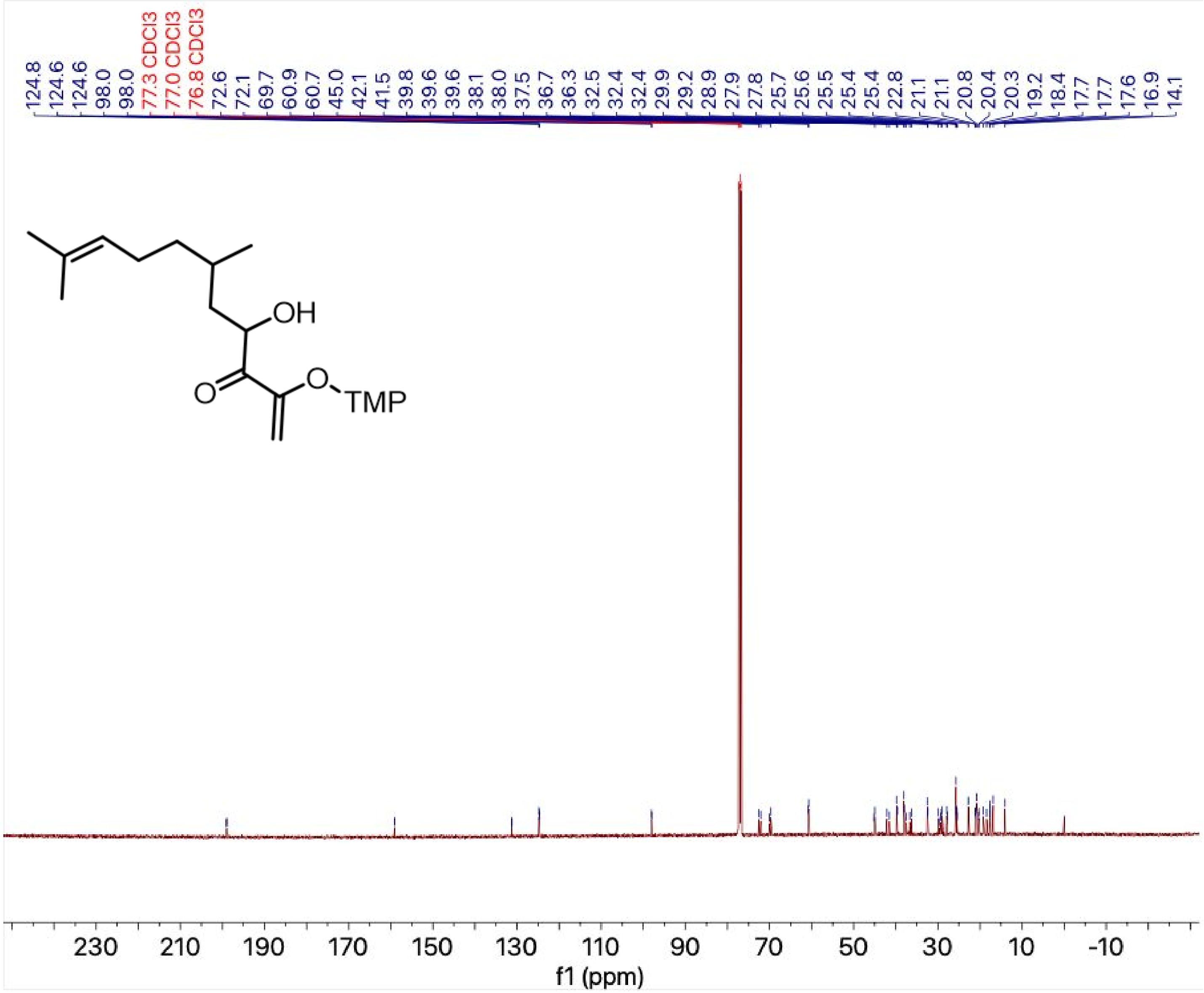

$^{1}H\{^{13}C\}$ 500 MHz NMR for Compound 4r.

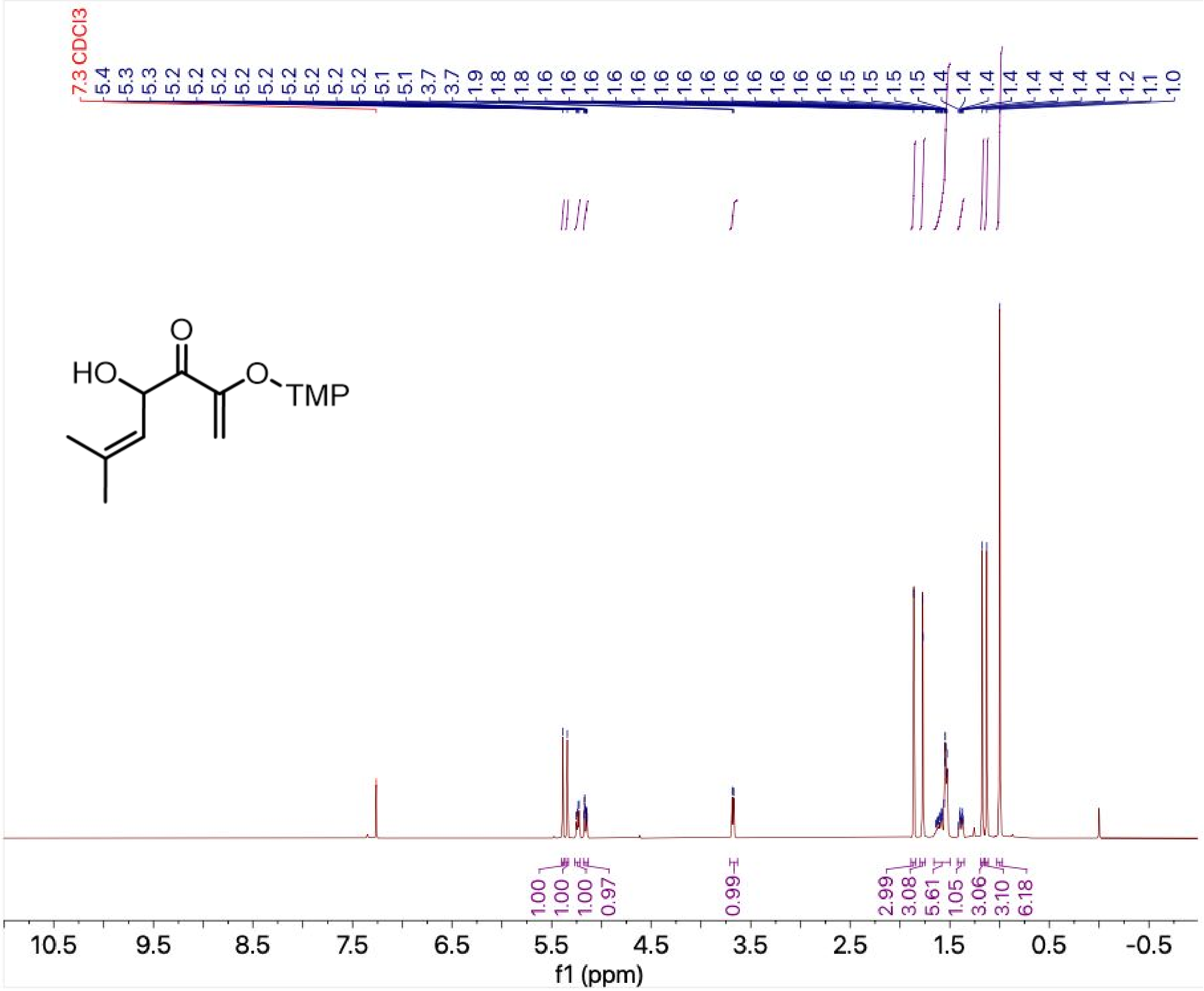

$^{13}C\{^1H\}$ **500 MHz NMR for Compound 4r.**

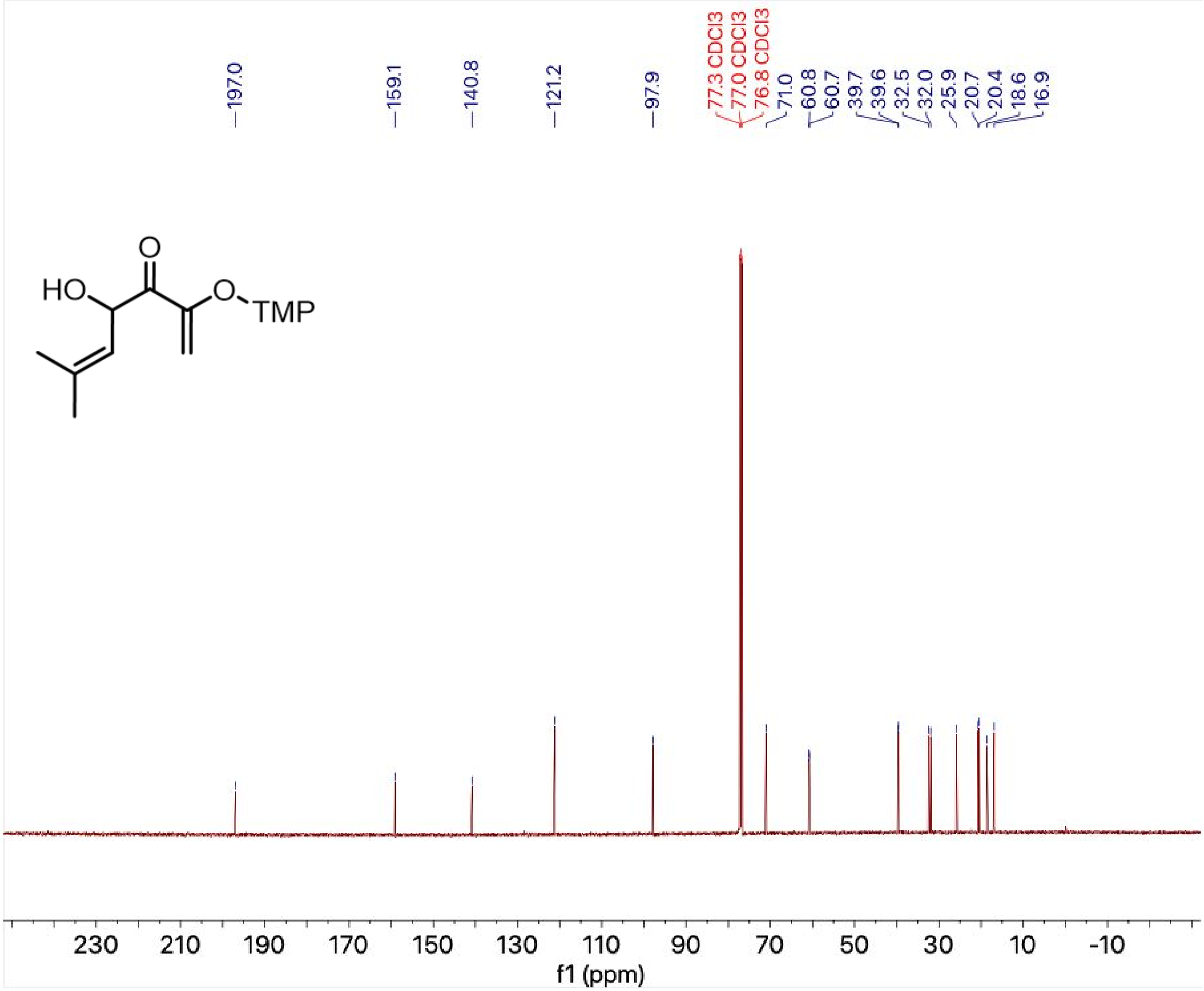

^{1}H{^{13}C} 500 MHz NMR for Compound 4s.

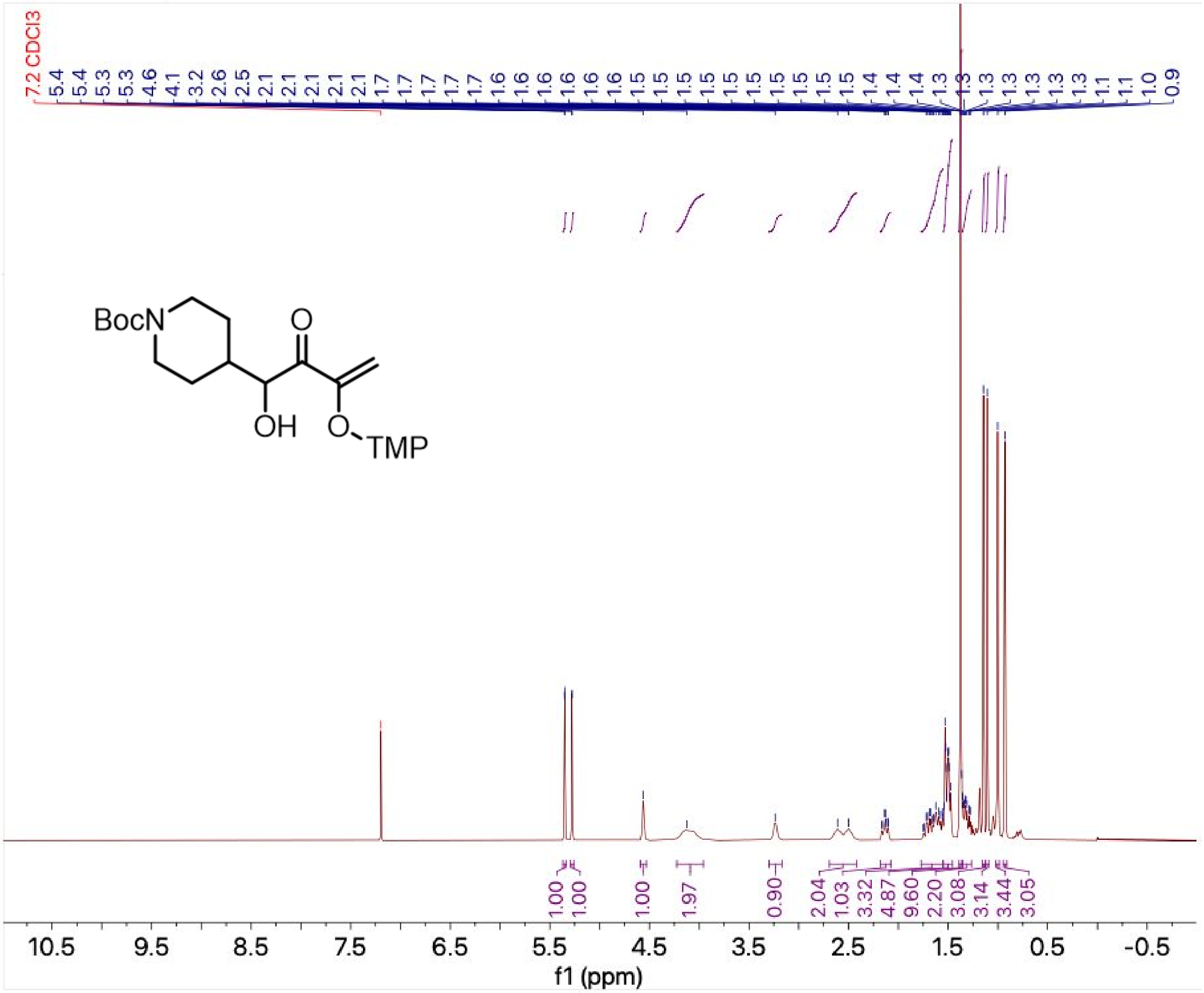

$^{13}C\{^{1}H\}$ 500 MHz NMR for Compound 4s.

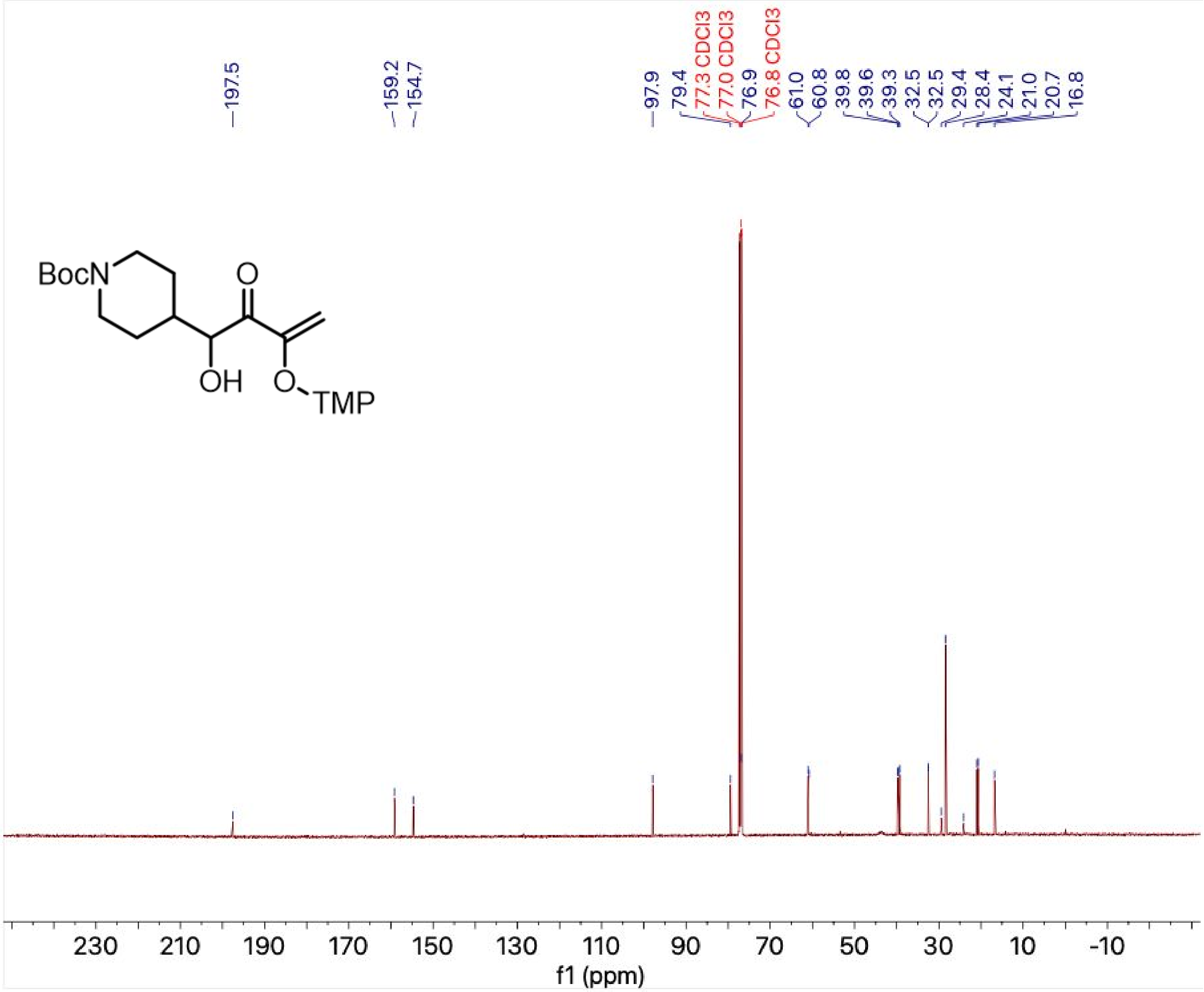

$^1H\{^{13}C\}$ **500 MHz NMR for Compound 4t.**

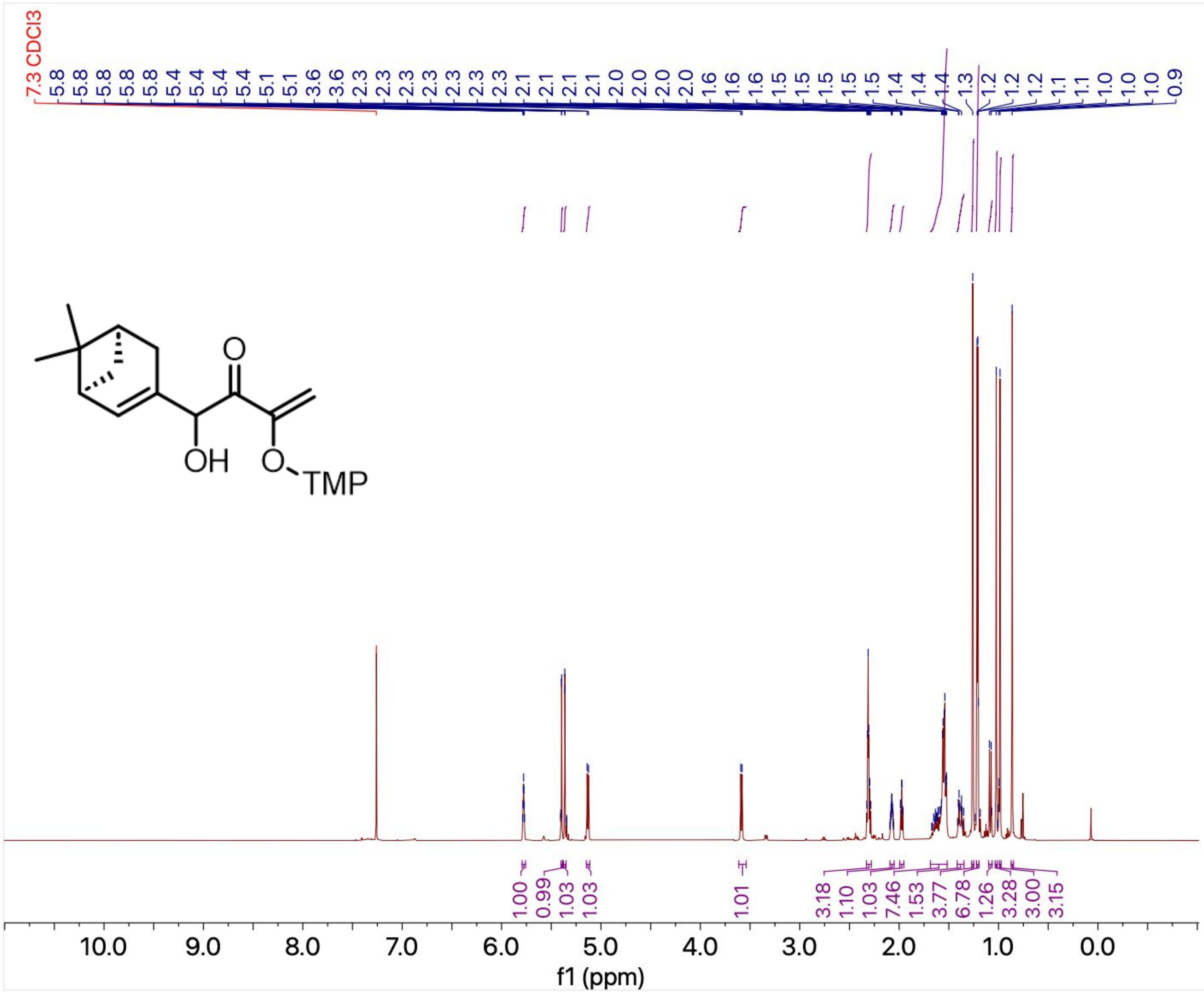

$^{13}C\{^{1}H\}$ 500 MHz NMR for Compound 4t.

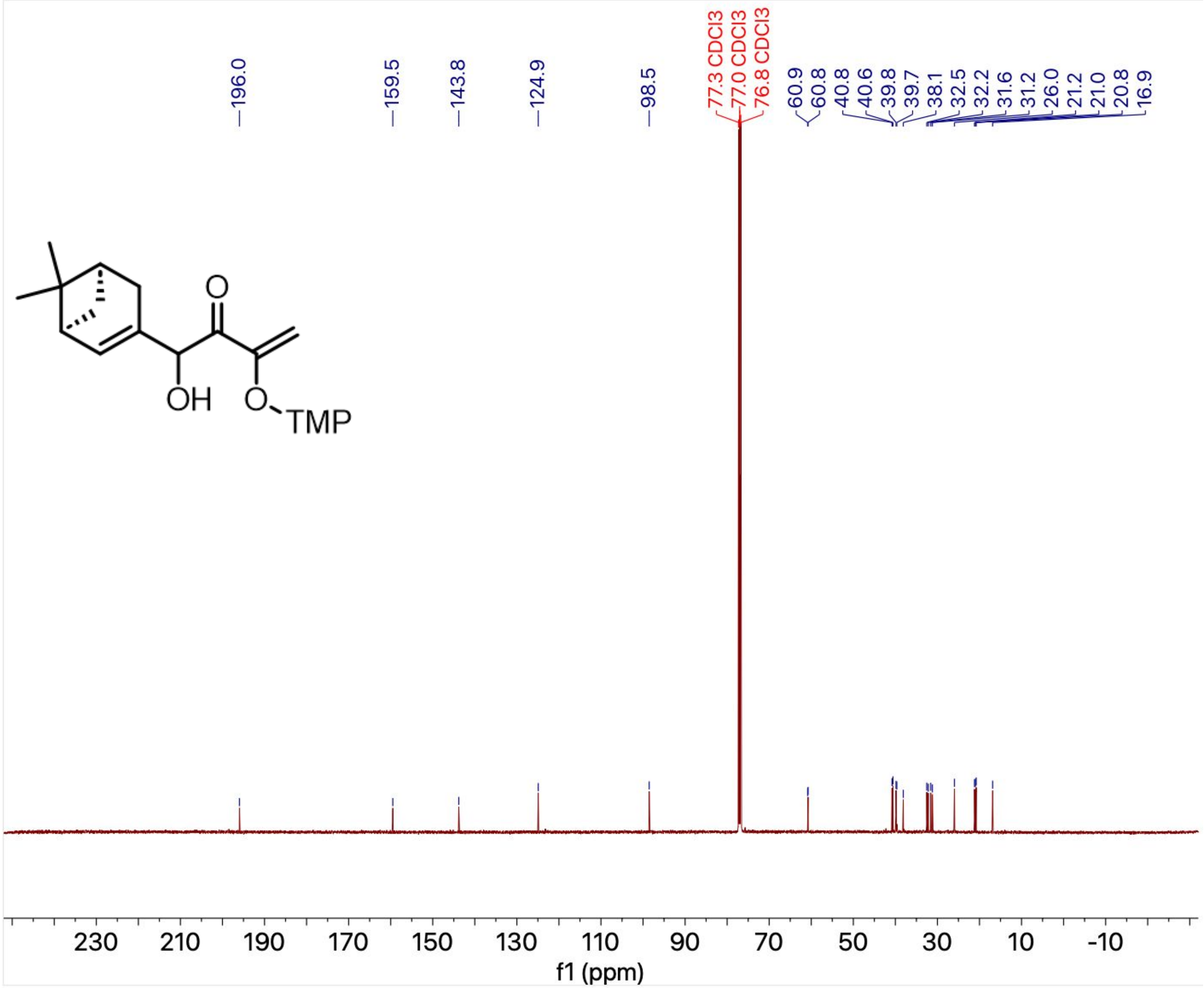

$^1H\{^{13}C\}$ **500 MHz NMR for Compound 4u.**

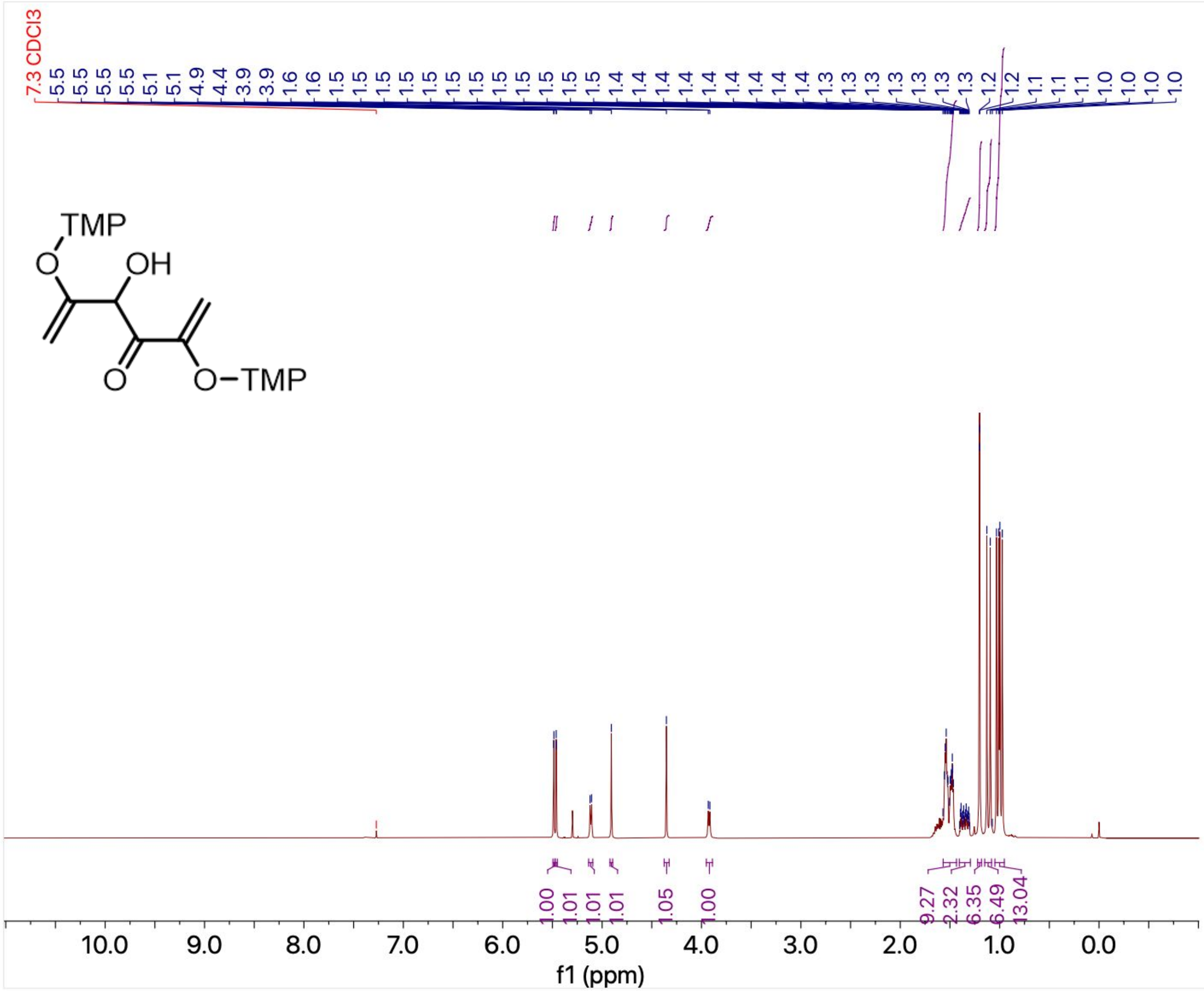

$^{13}C\{^{1}H\}$ **500 MHz NMR for Compound 4u.**

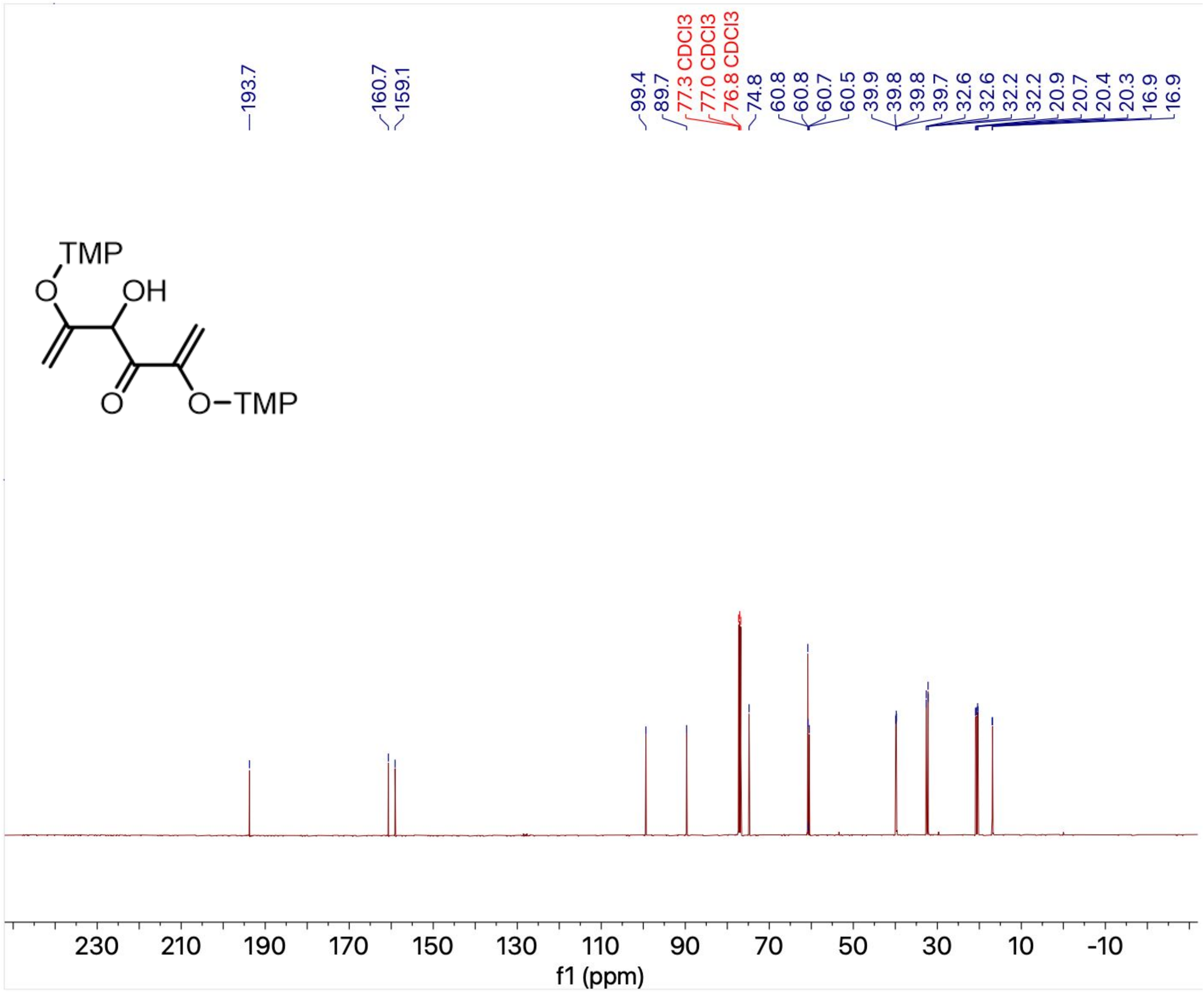

$^{1}H\{^{13}C\}$ 500 MHz NMR for Compound 4v.

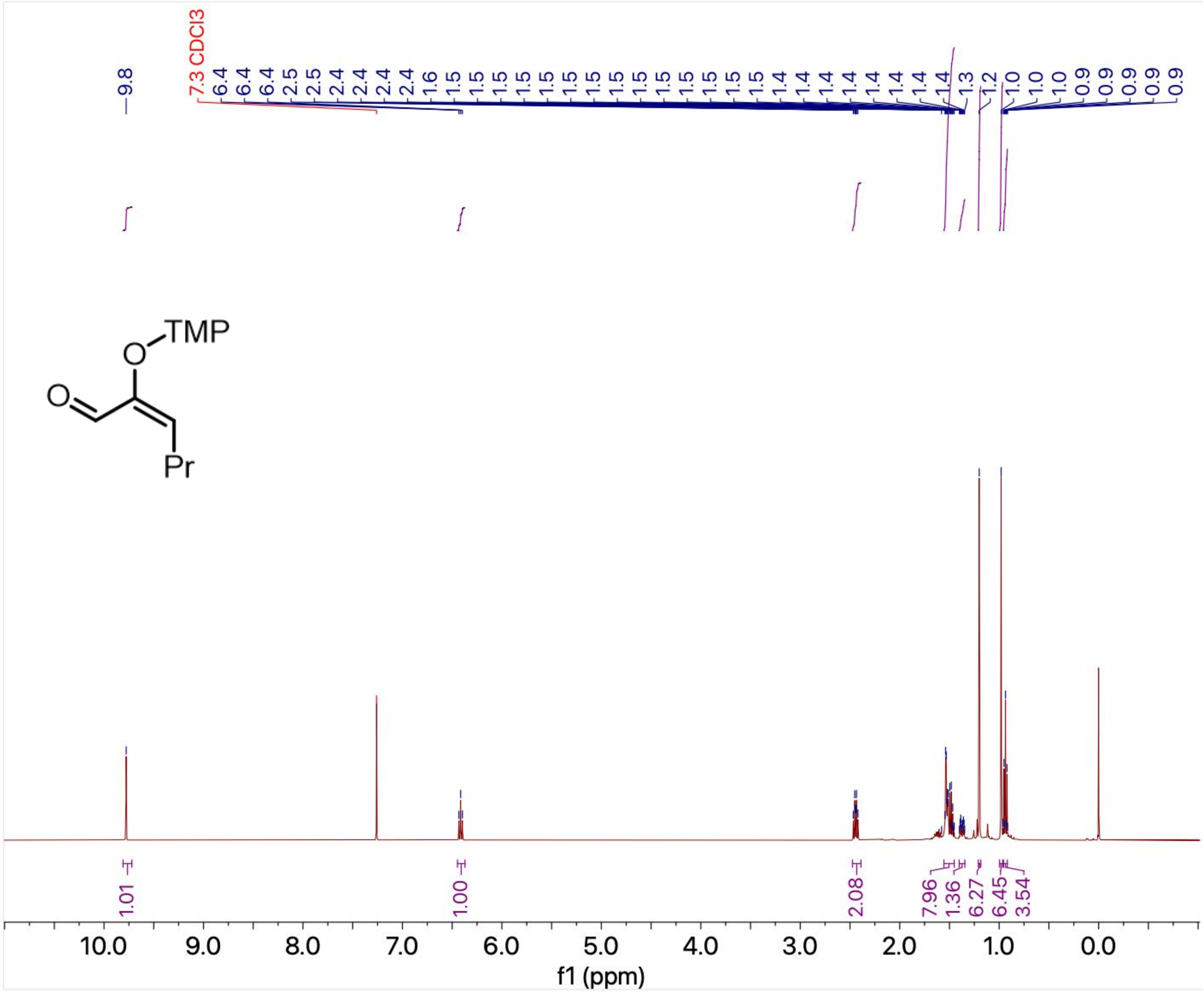

$^{13}C\{^{1}H\}$ 500 MHz NMR for Compound 4v.

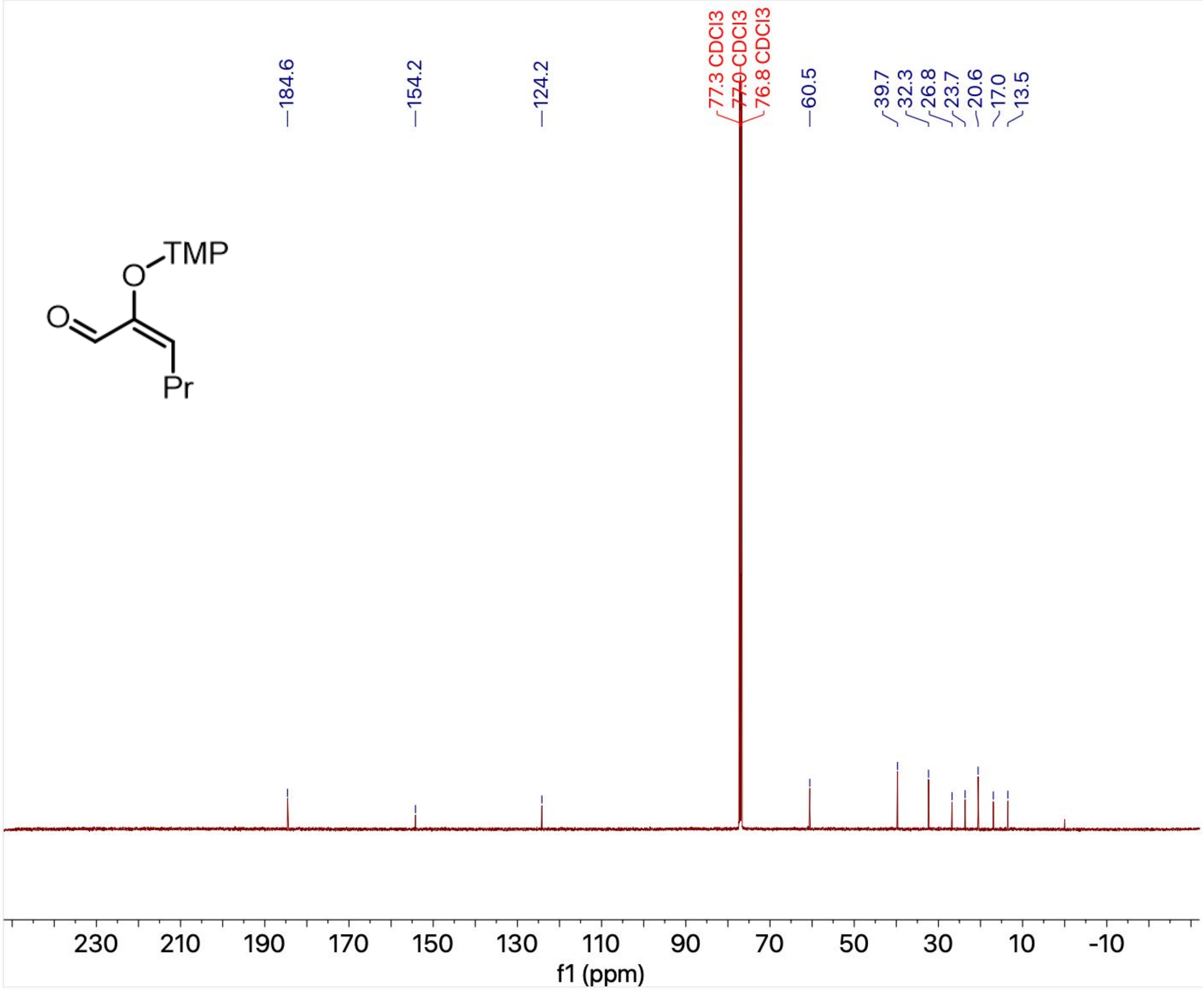

$^{1}H\{^{13}C\}$ 500 MHz NMR for Compound 4w.

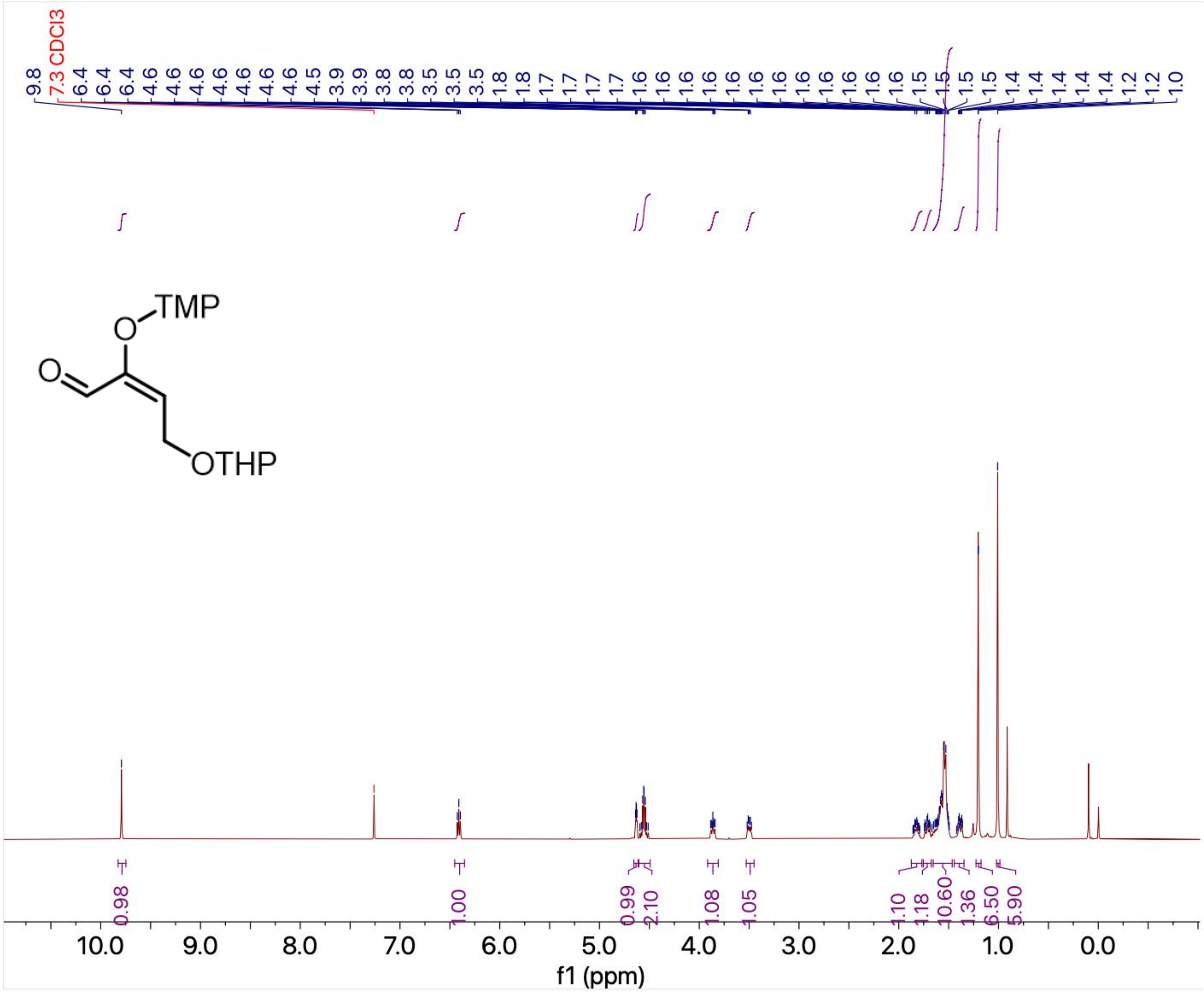

$^{13}C\{^1H\}$ 500 MHz NMR for Compound 4w.

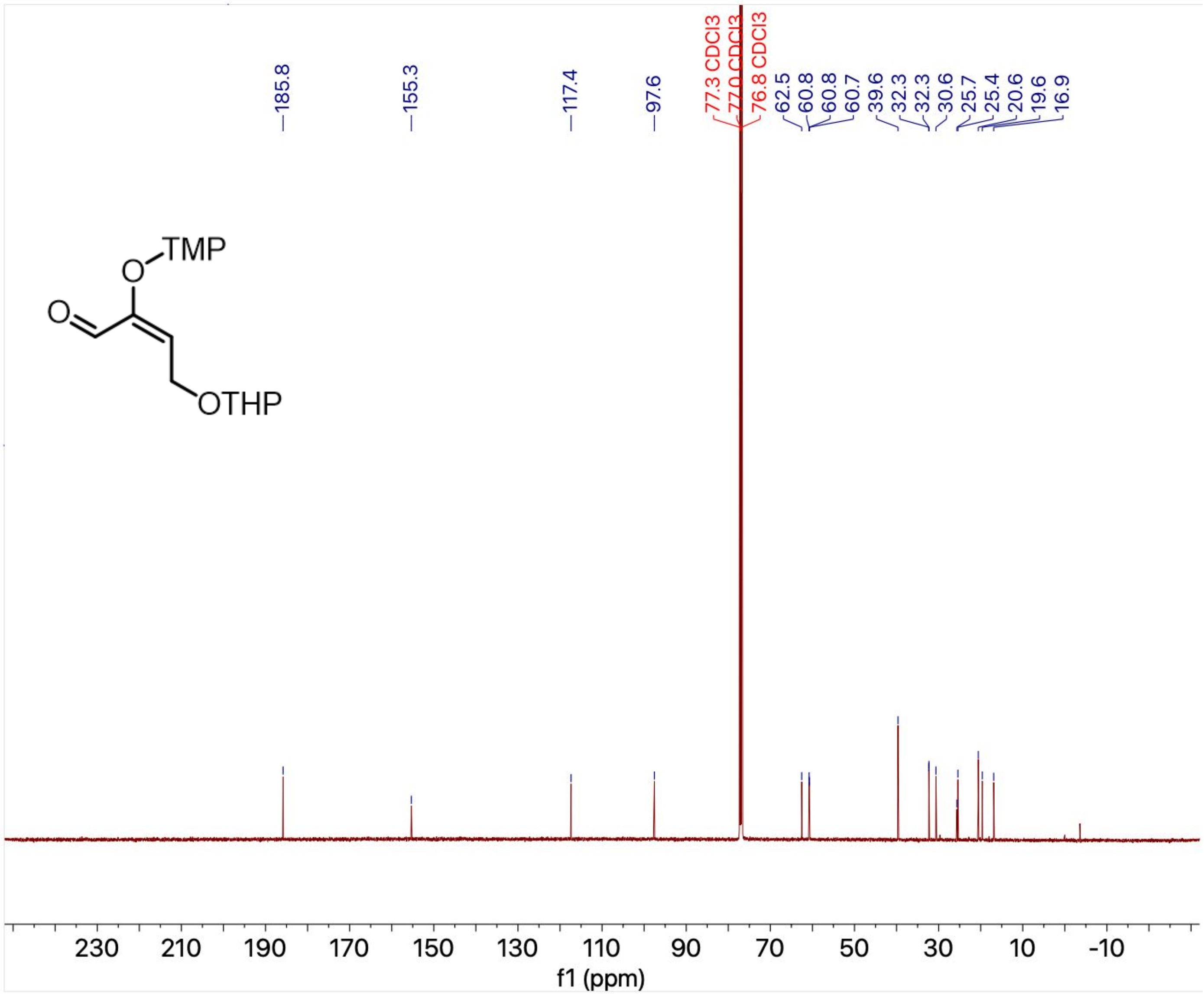

$^{1}H\{^{13}C\}$ **500 MHz NMR for Compound 8 and TMP+.**

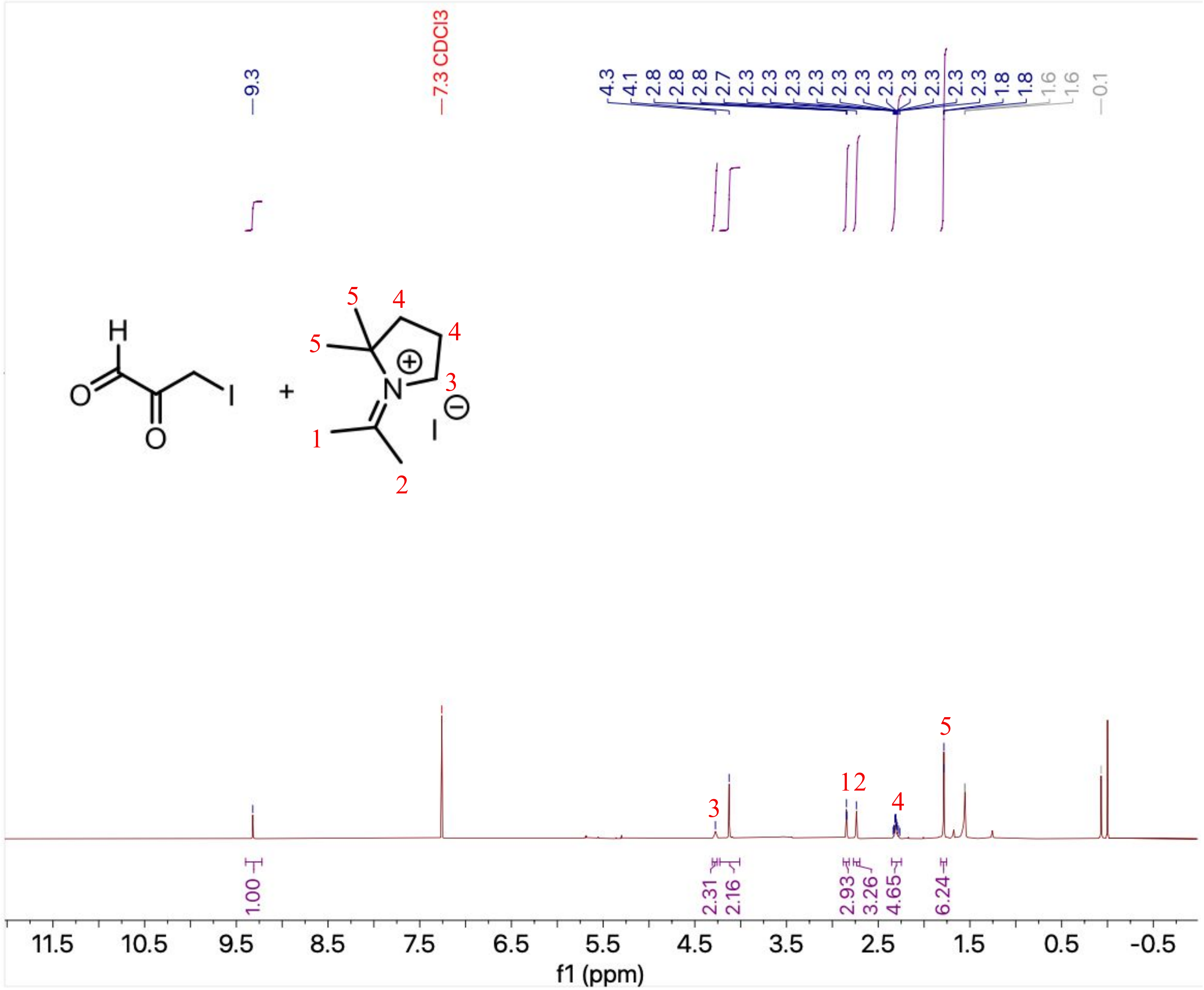

$^{13}C\{^{1}H\}$ 500 MHz NMR for Compound 8 and TMP+.

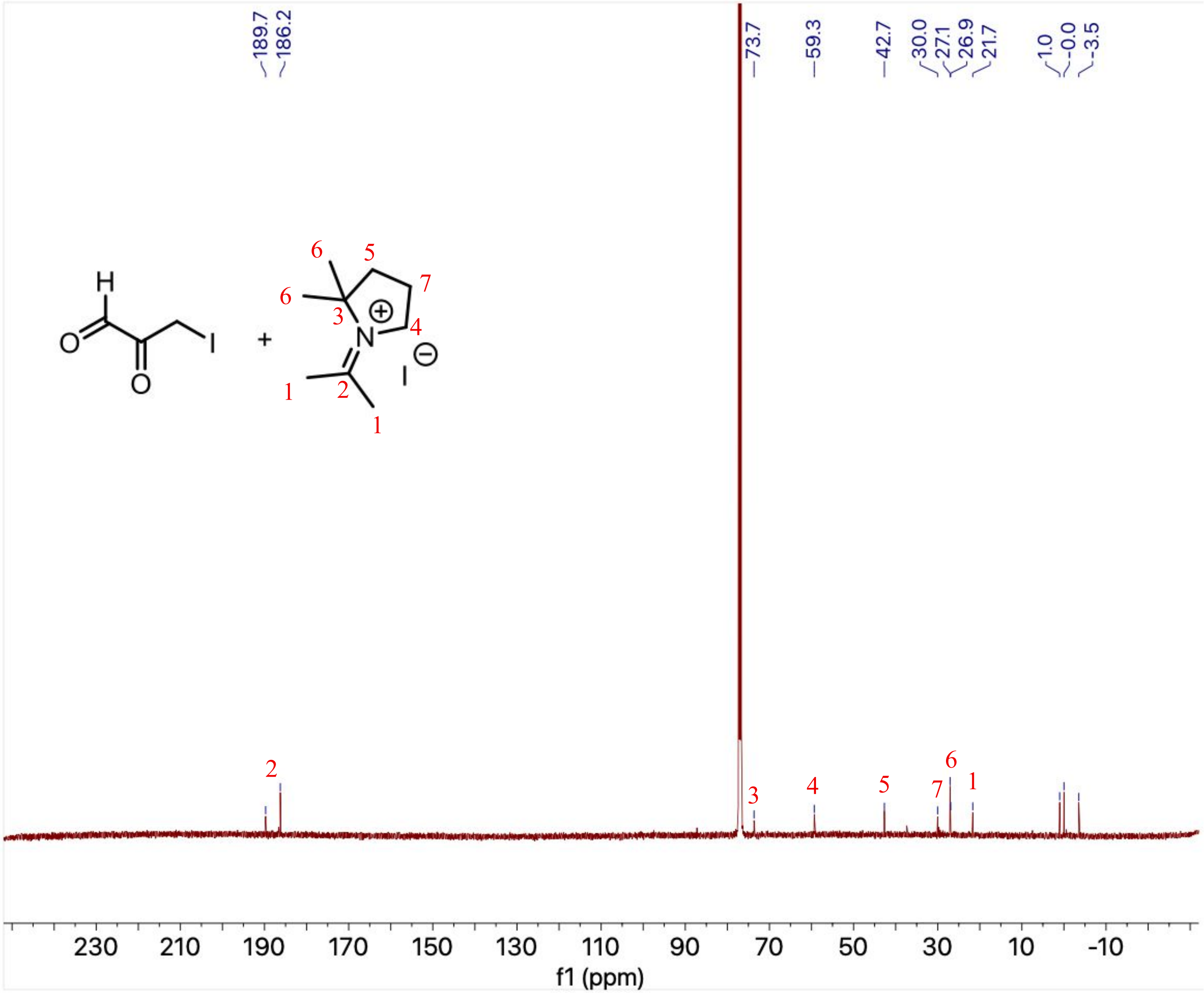

$^1H\{^{13}C\}$ 500 MHz NMR for Compound 9.

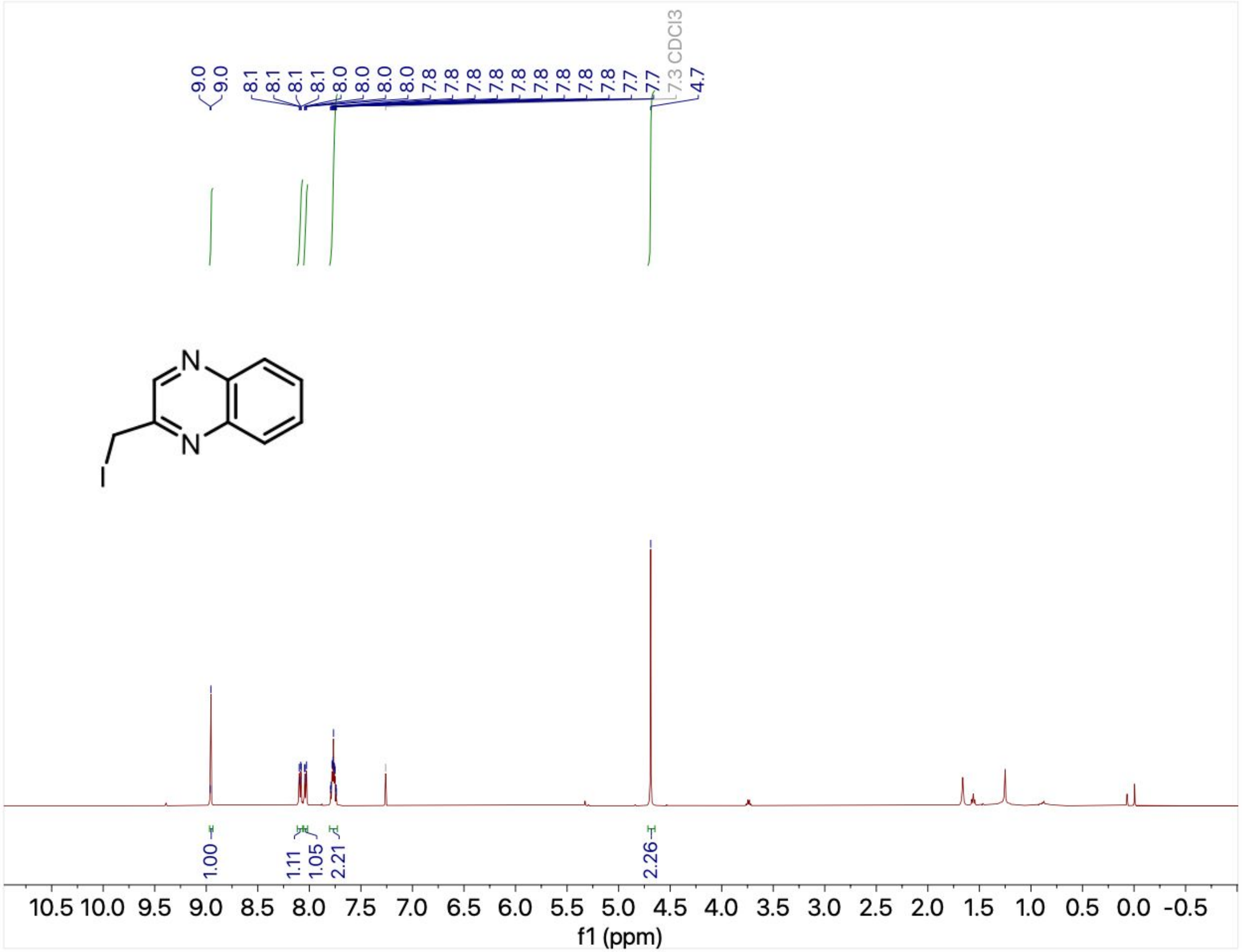

$^{13}C\{^{1}H\}$ **500 MHz NMR for Compound 9.**

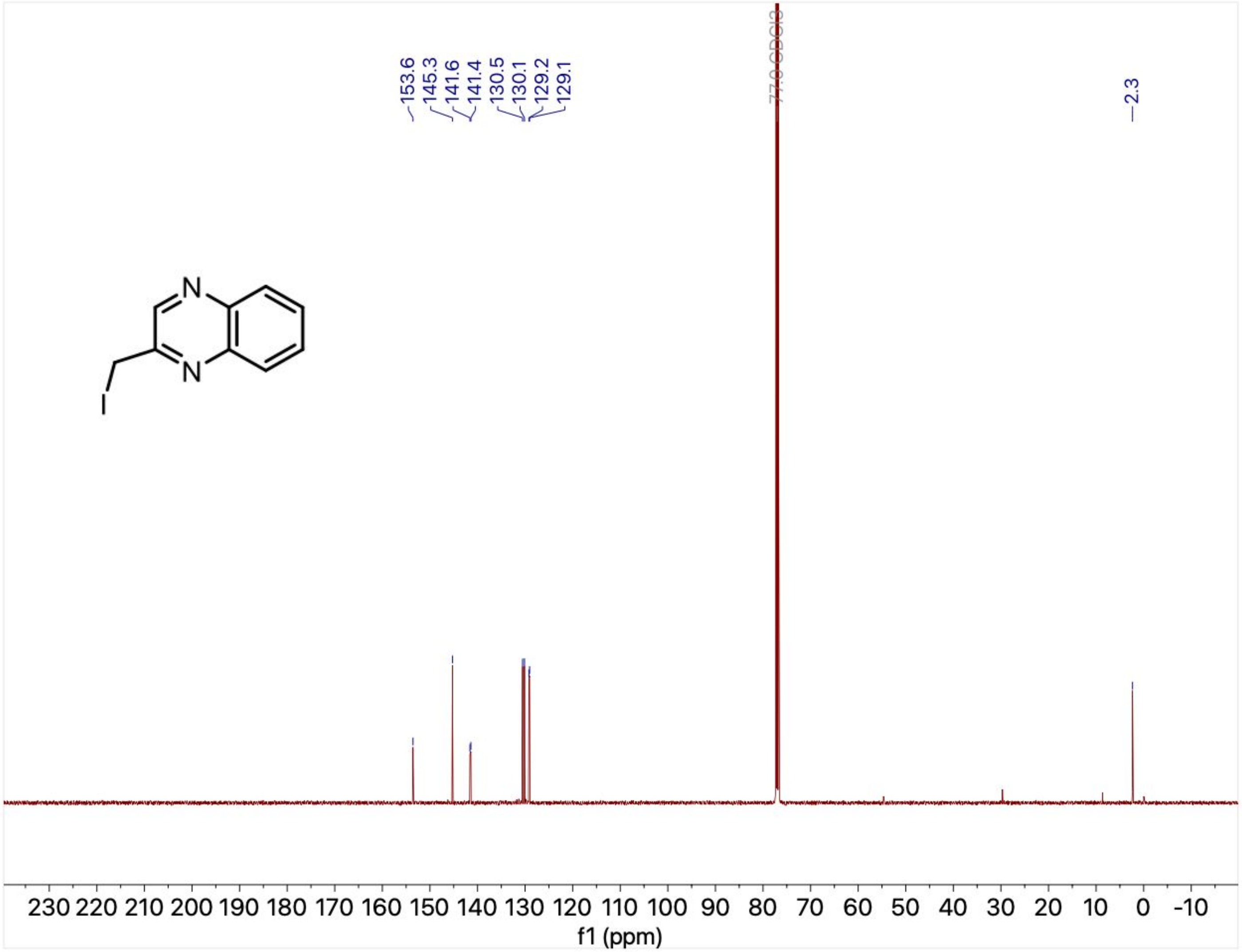

$^{1}H\{^{13}C\}$ 500 MHz NMR for Compound 10.

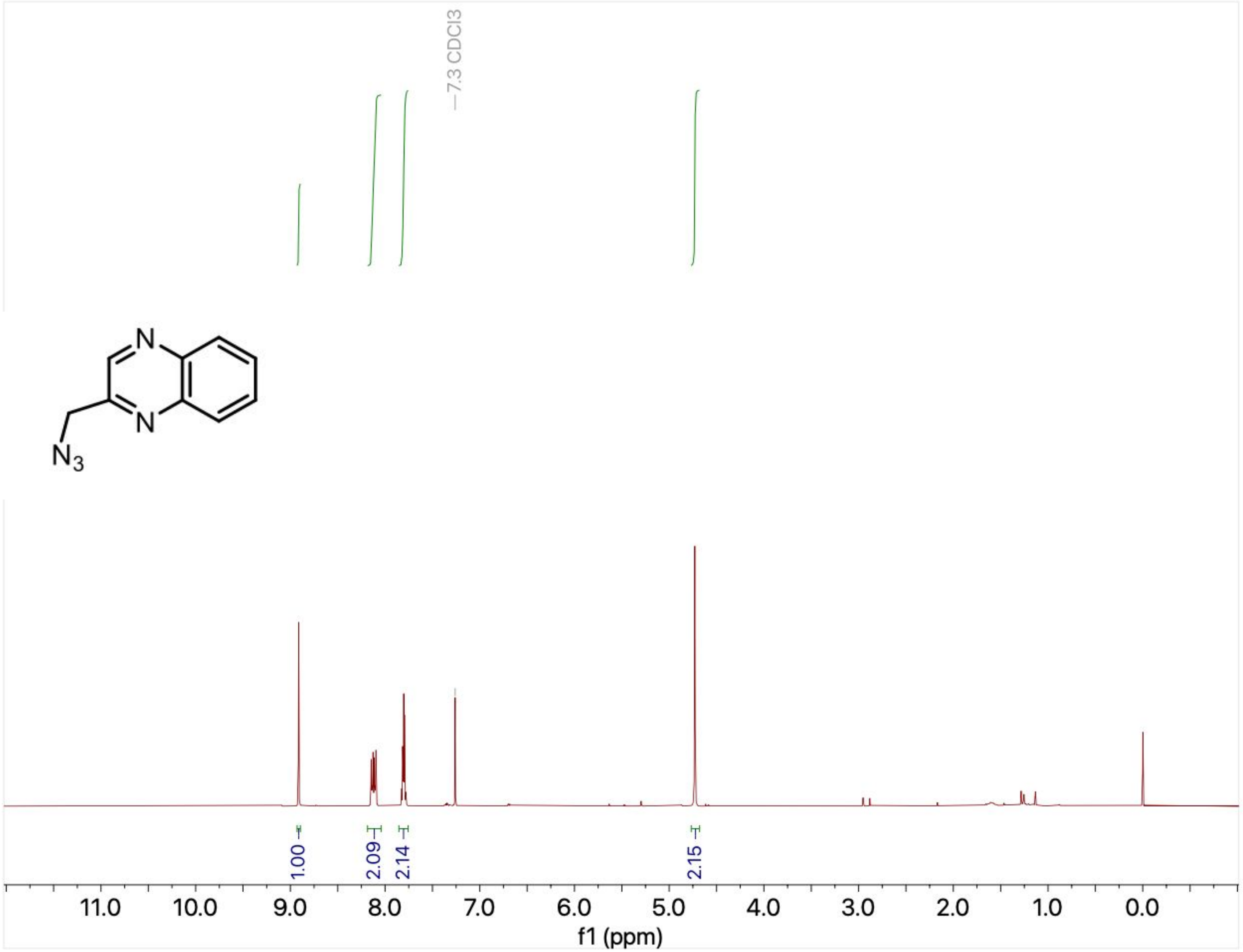

^{13}C{^{1}H} 500 MHz NMR for Compound 10.

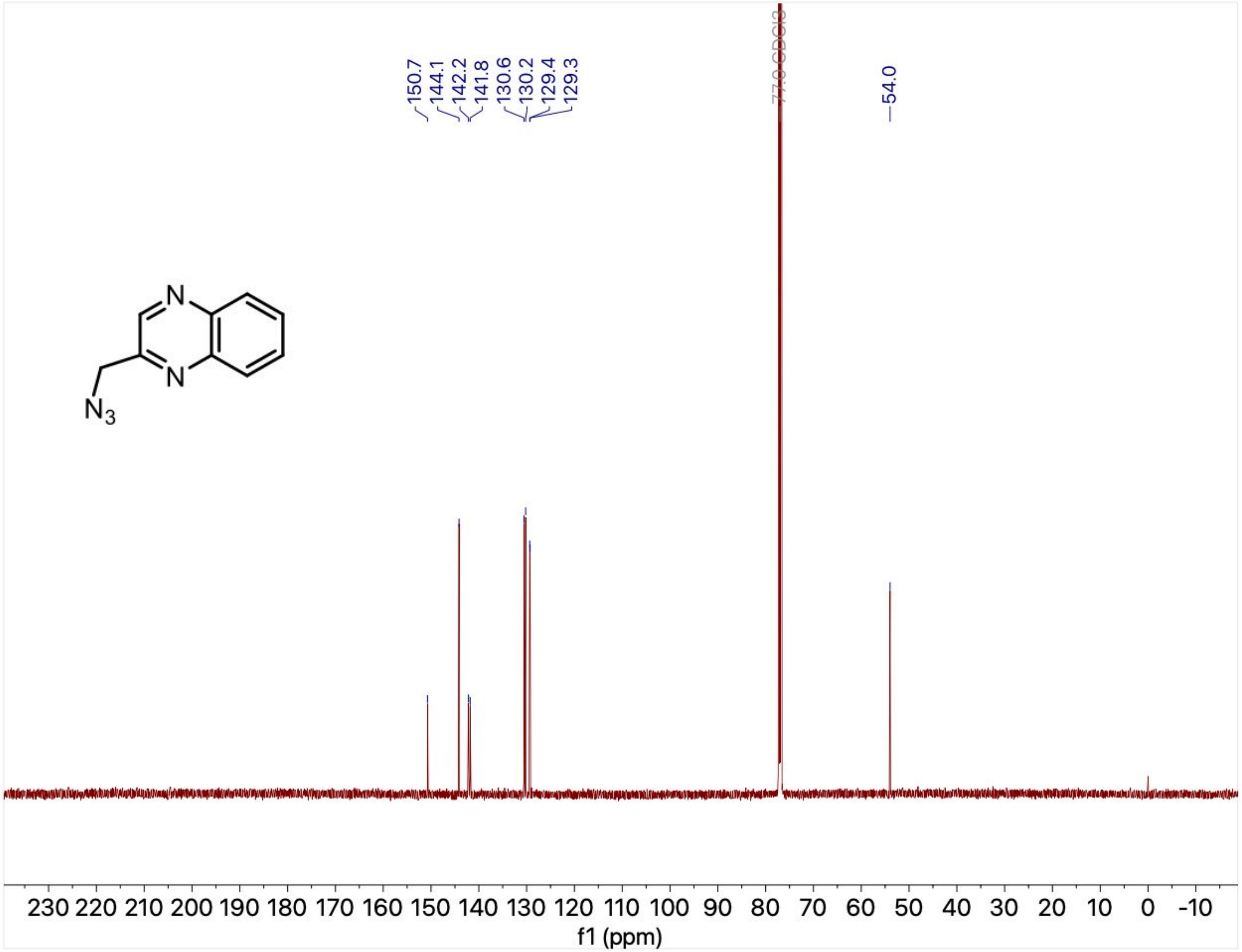

$^{1}H\{^{13}C\}$ **500 MHz NMR for Compound 11.**

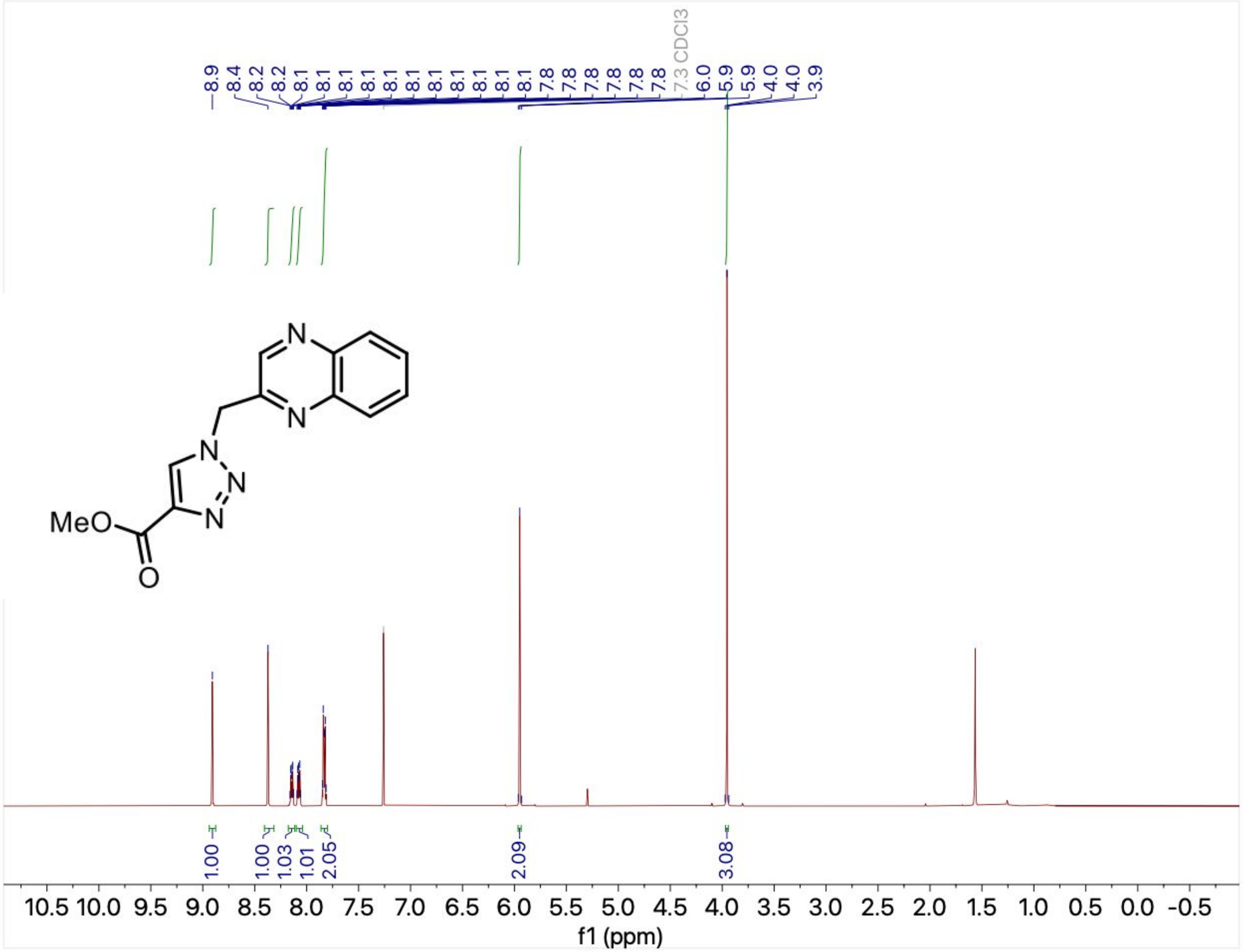

$^{13}C\{^{1}H\}$ 500 MHz NMR for Compound 11.

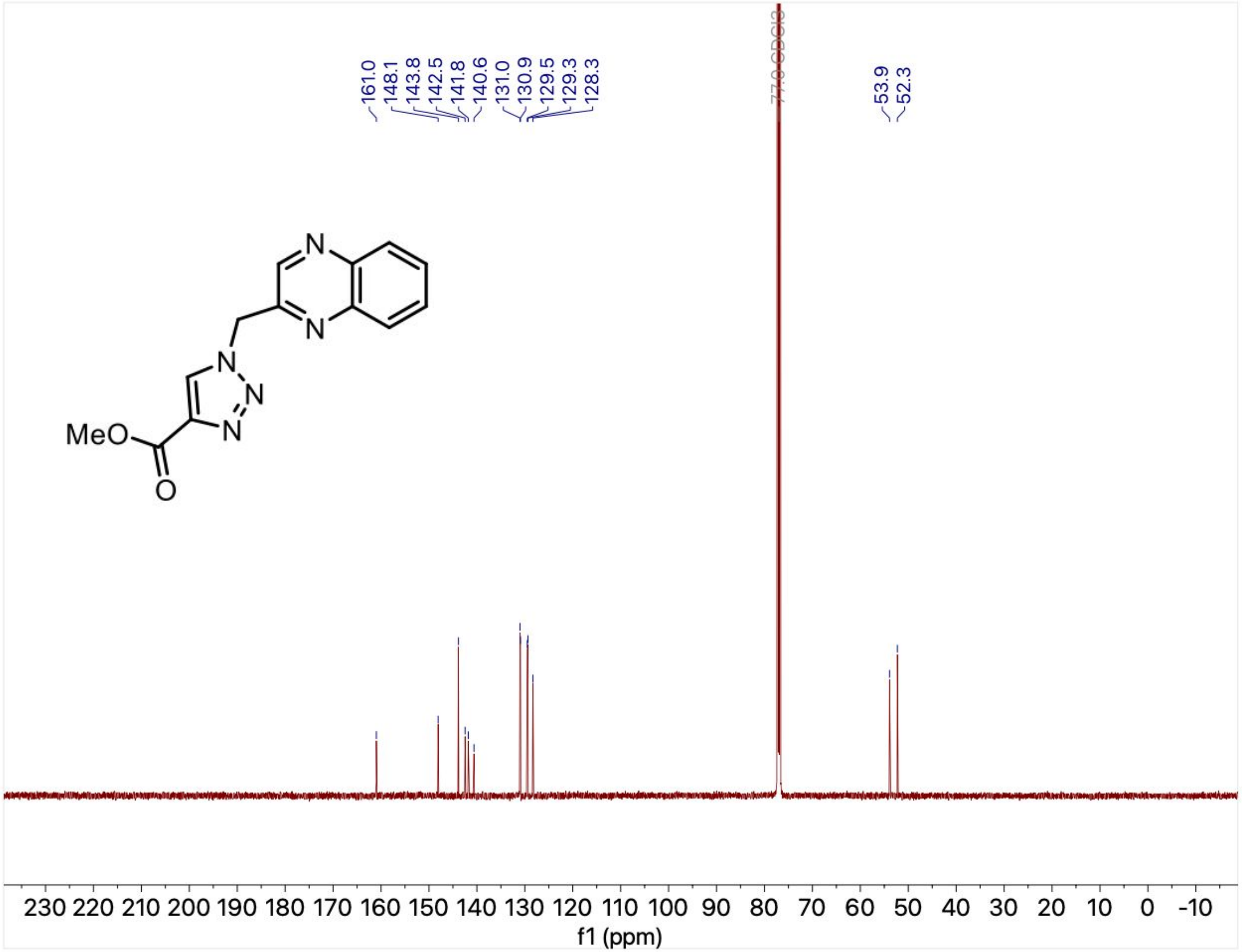

$^{1}H\{^{13}C\}$ 500 MHz NMR for Compound 12.

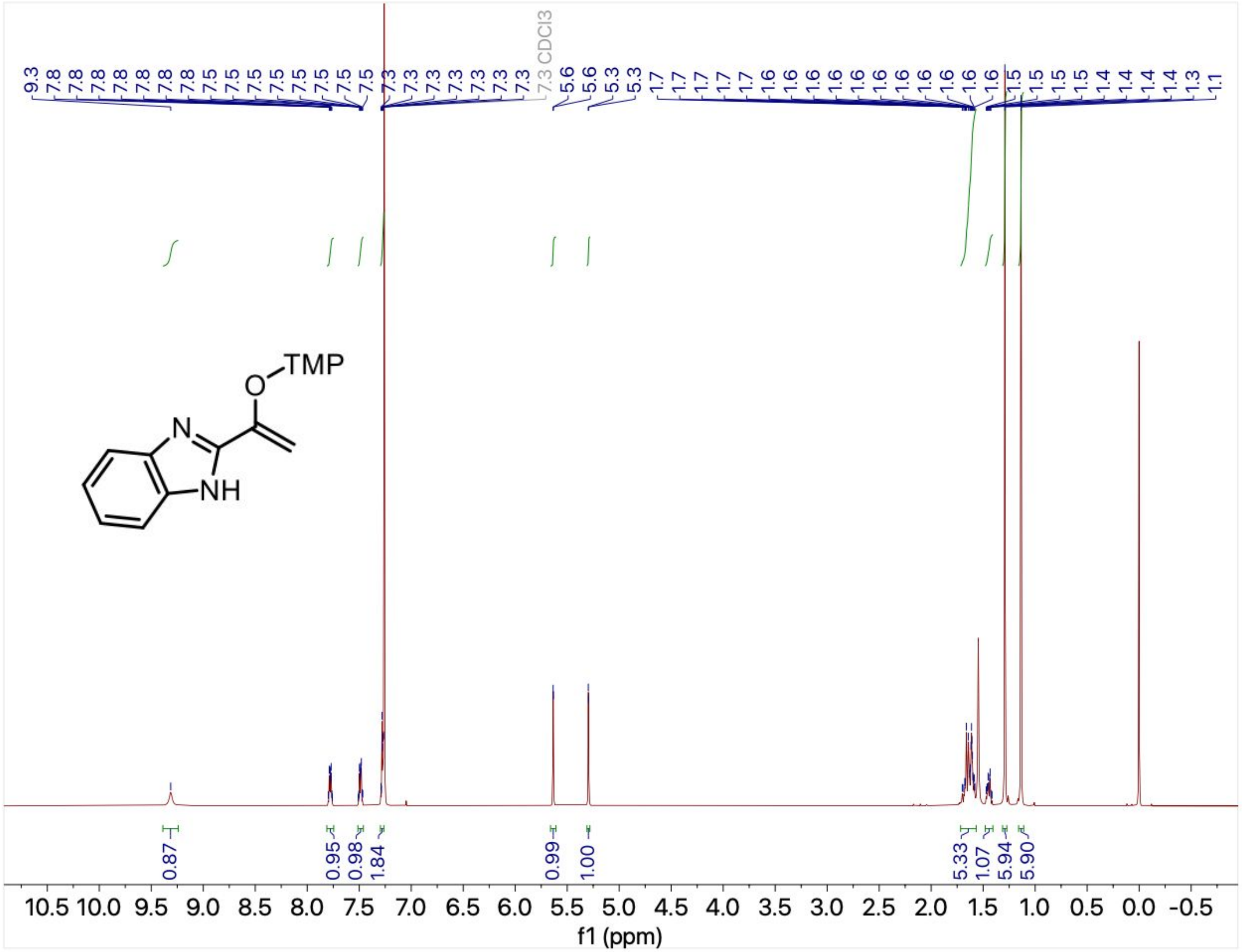

$^{13}C\{^1H\}$ **500 MHz NMR for Compound 12.**

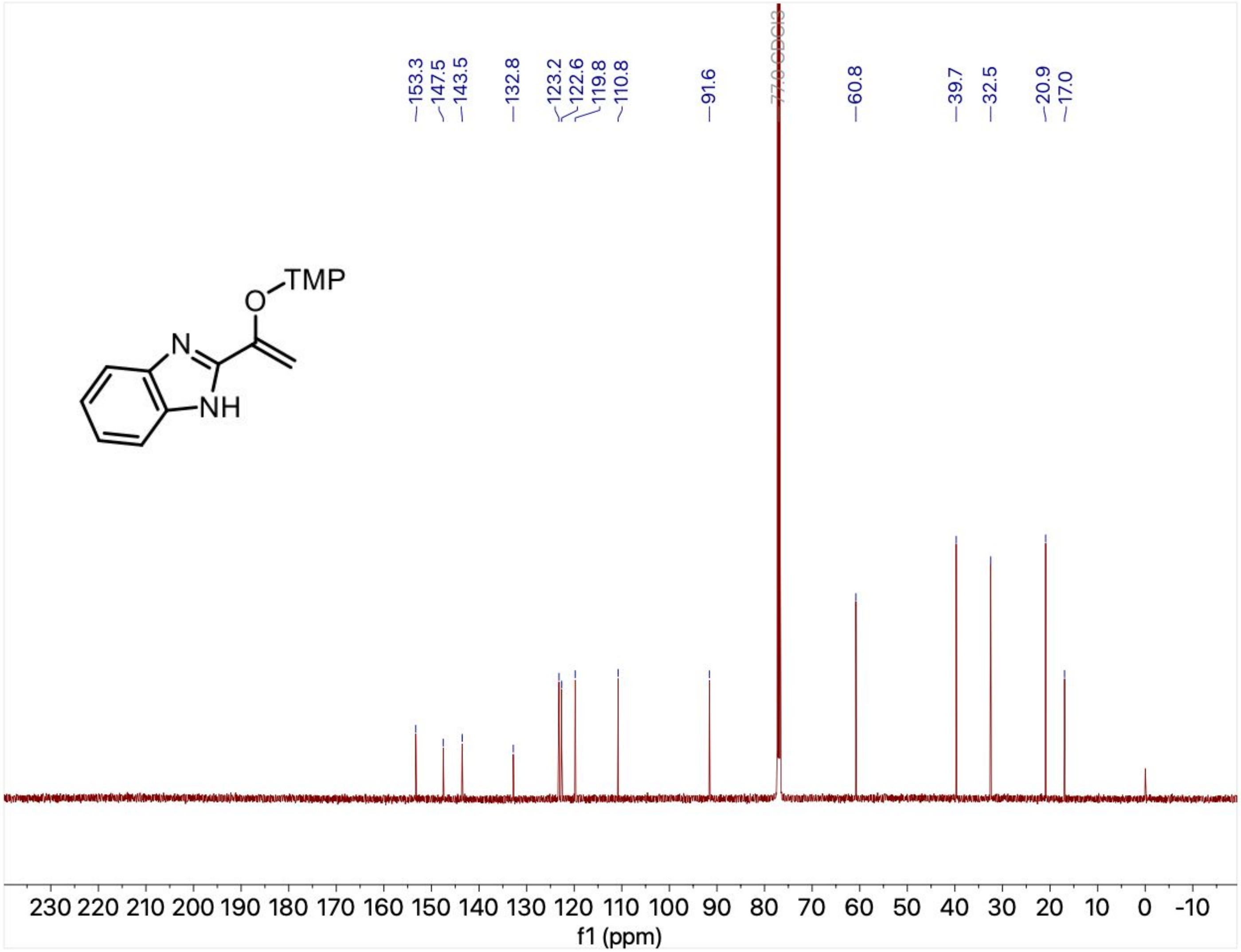

$^{1}H\{^{13}C\}$ **500 MHz NMR for Compound 13.**

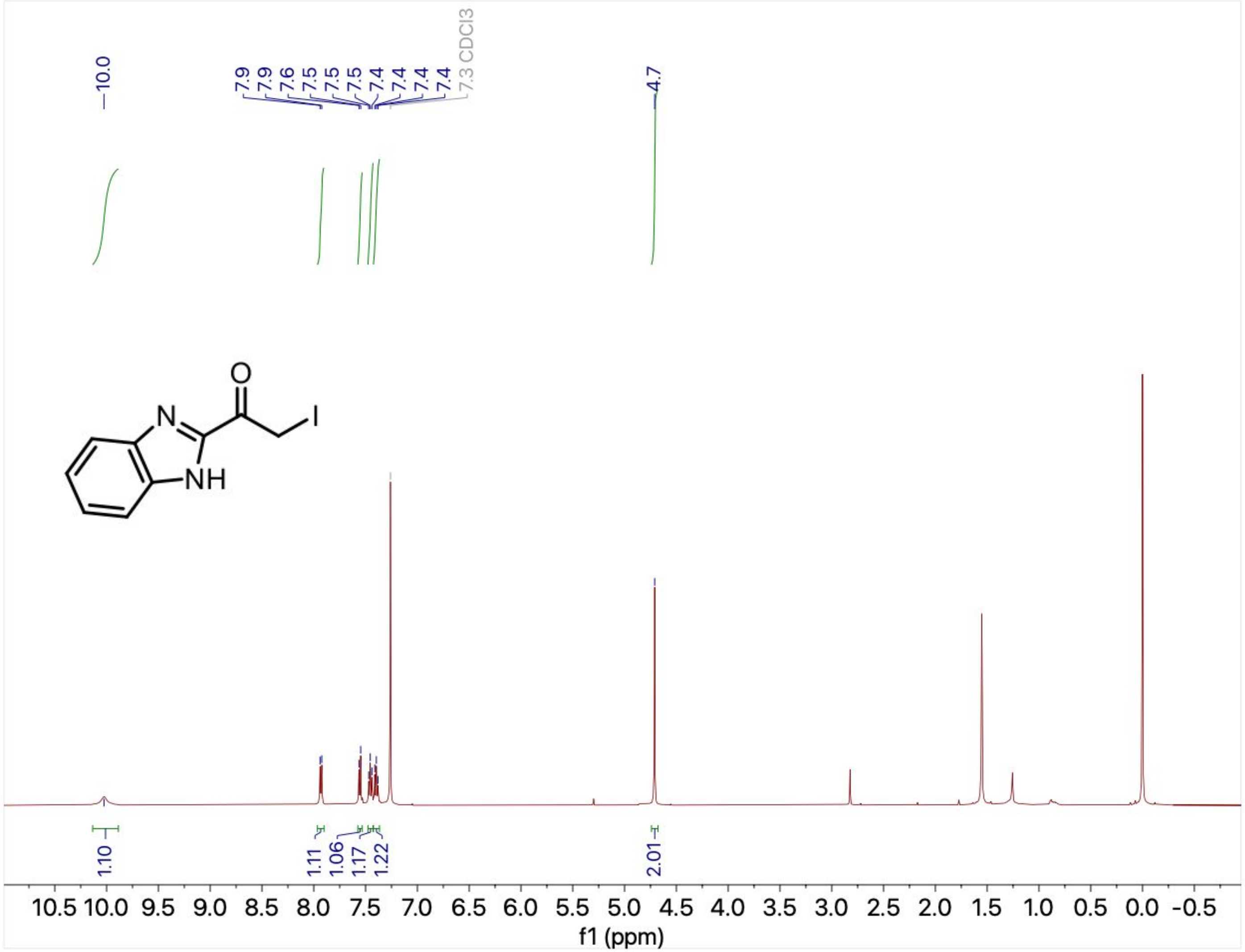

$^{13}C\{^{1}H\}$ 500 MHz NMR for Compound 13.

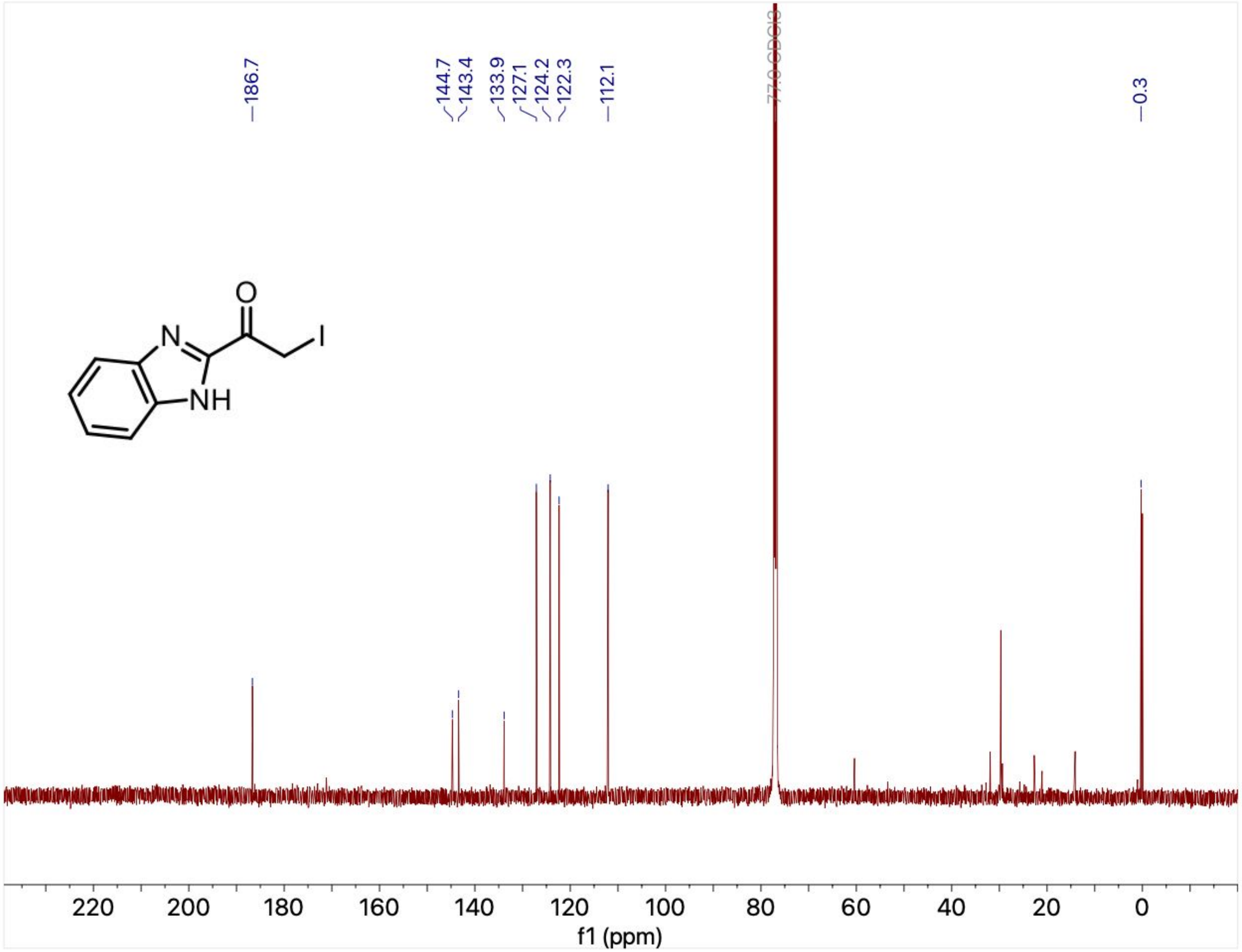

$^{1}H\{^{13}C\}$ 500 MHz NMR for Compound 14.

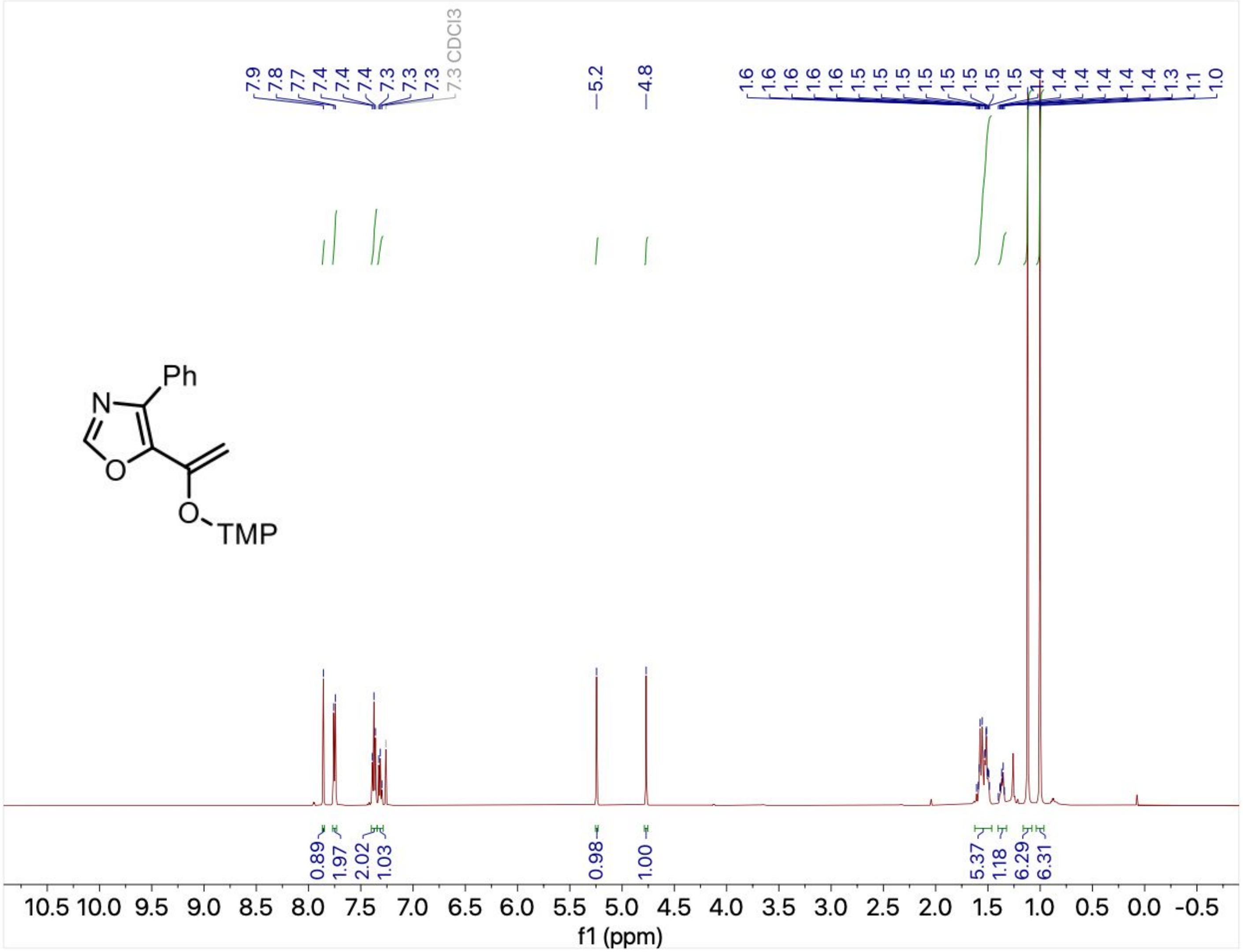

$^{13}C\{^{1}H\}$ **500 MHz NMR for Compound 14.**

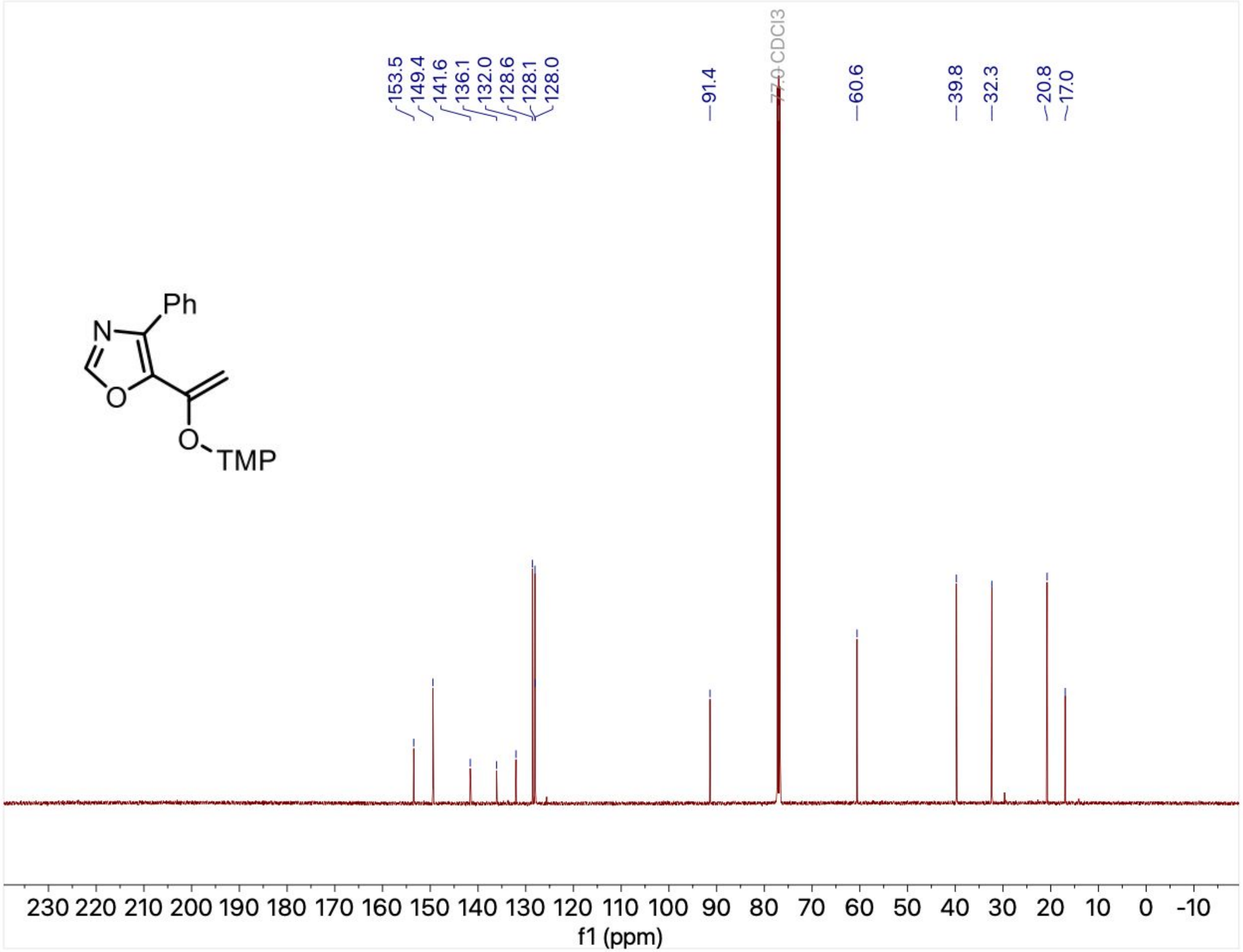

$^{1}H\{^{13}C\}$ **500 MHz NMR for Compound 15.**

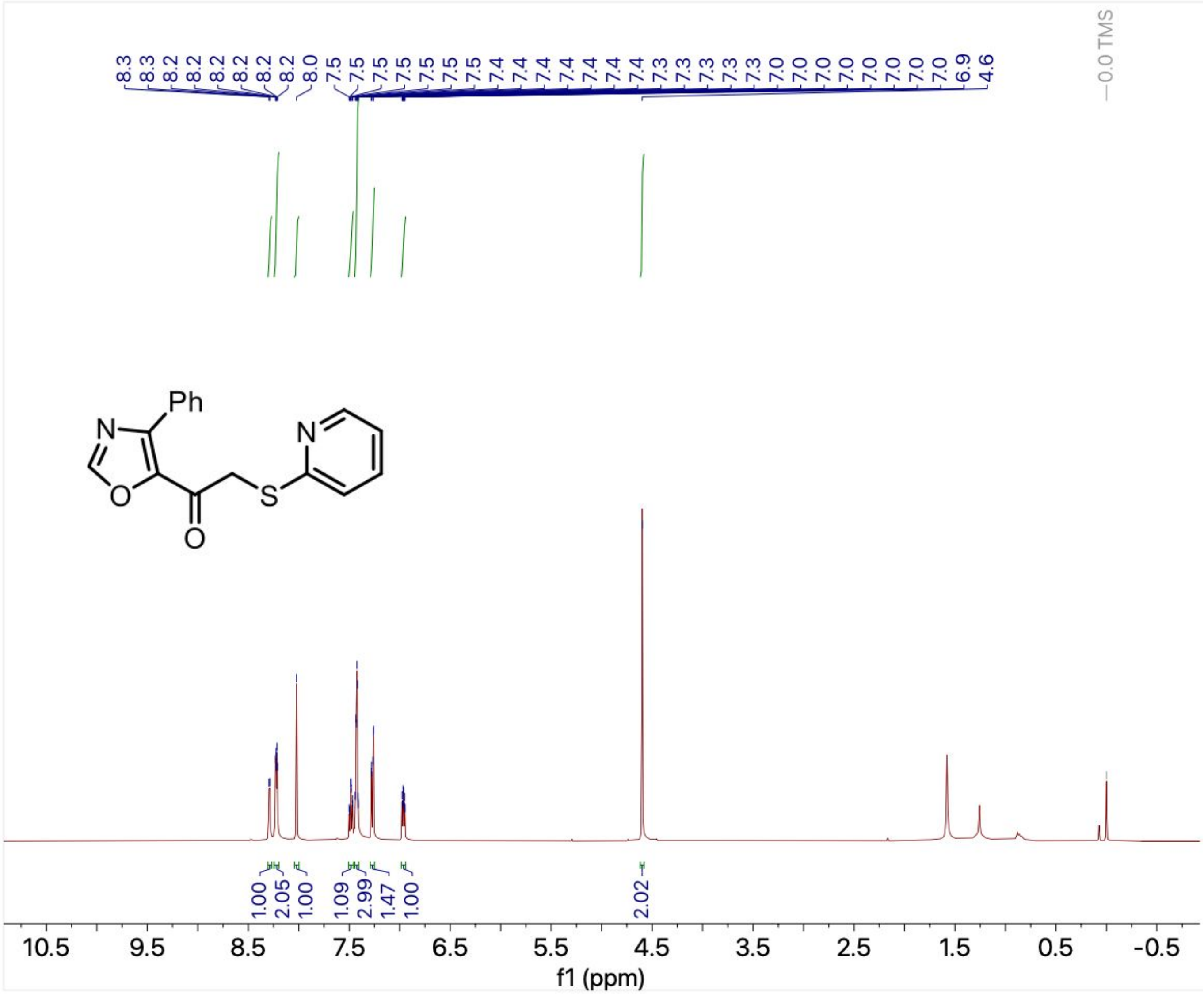

$^{13}C\{^{1}H\}$ 500 MHz NMR for Compound 15.

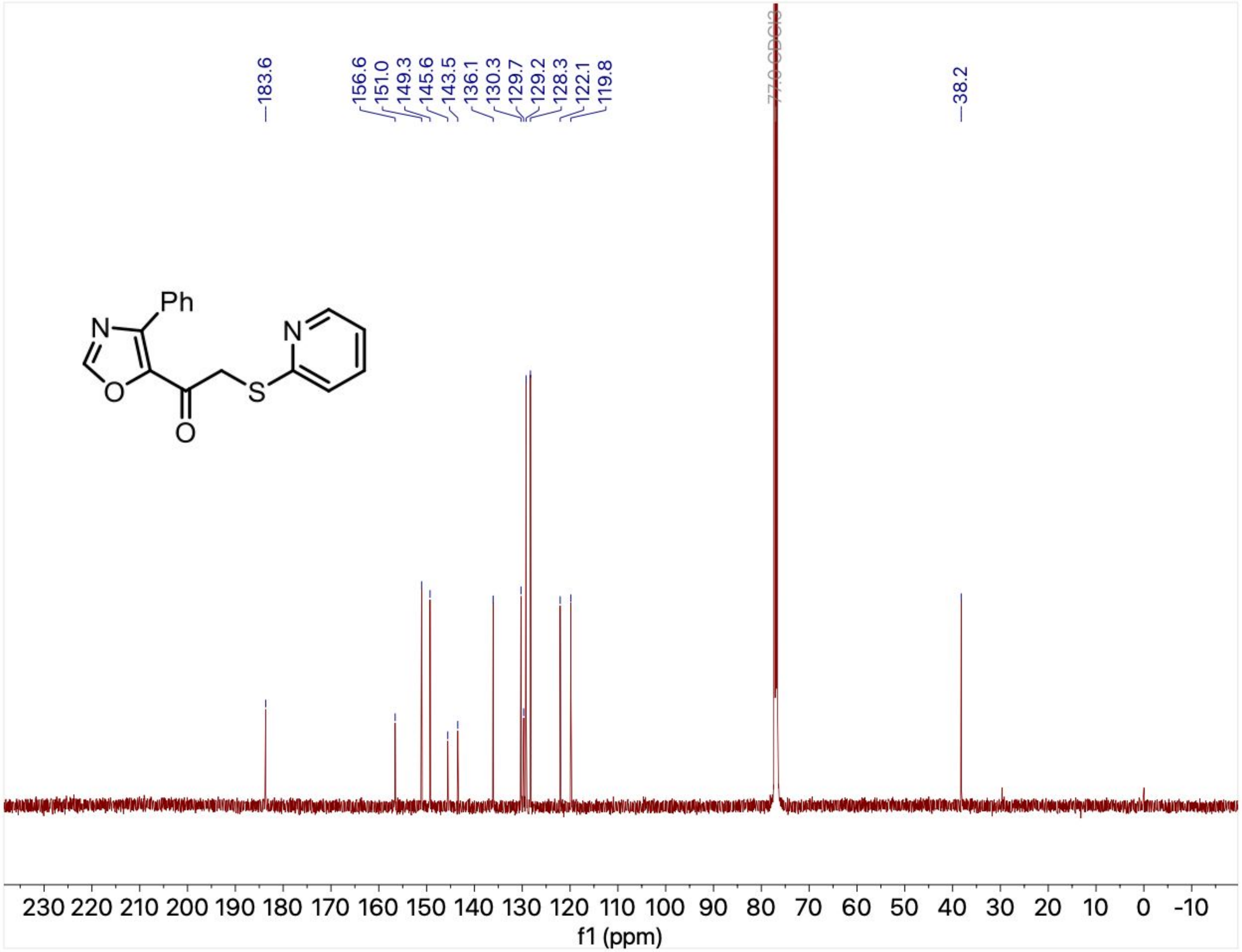

^{1}H{^{13}C} 500 MHz NMR for Compound 16.

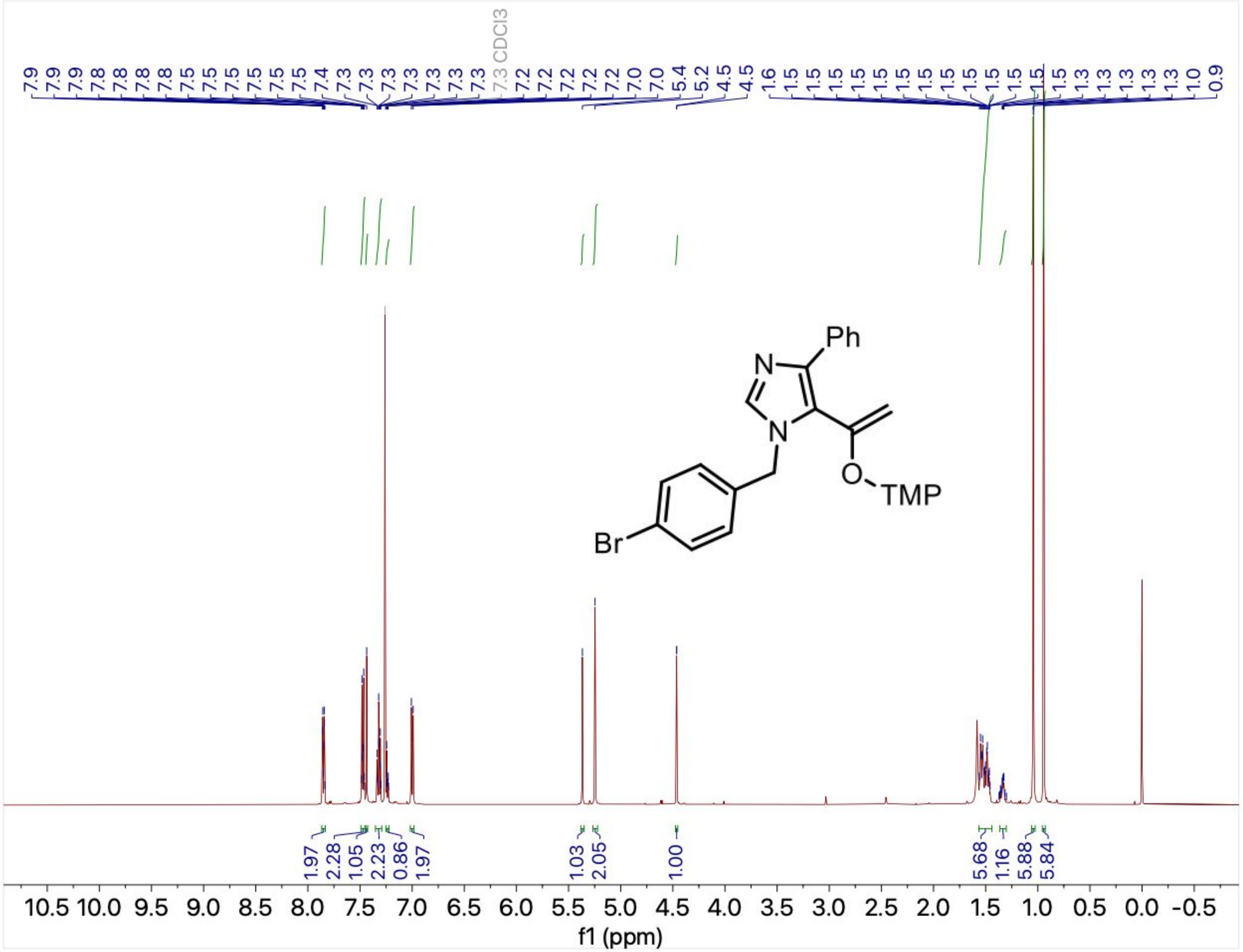

$^{13}C\{^{1}H\}$ 500 MHz NMR for Compound 16.

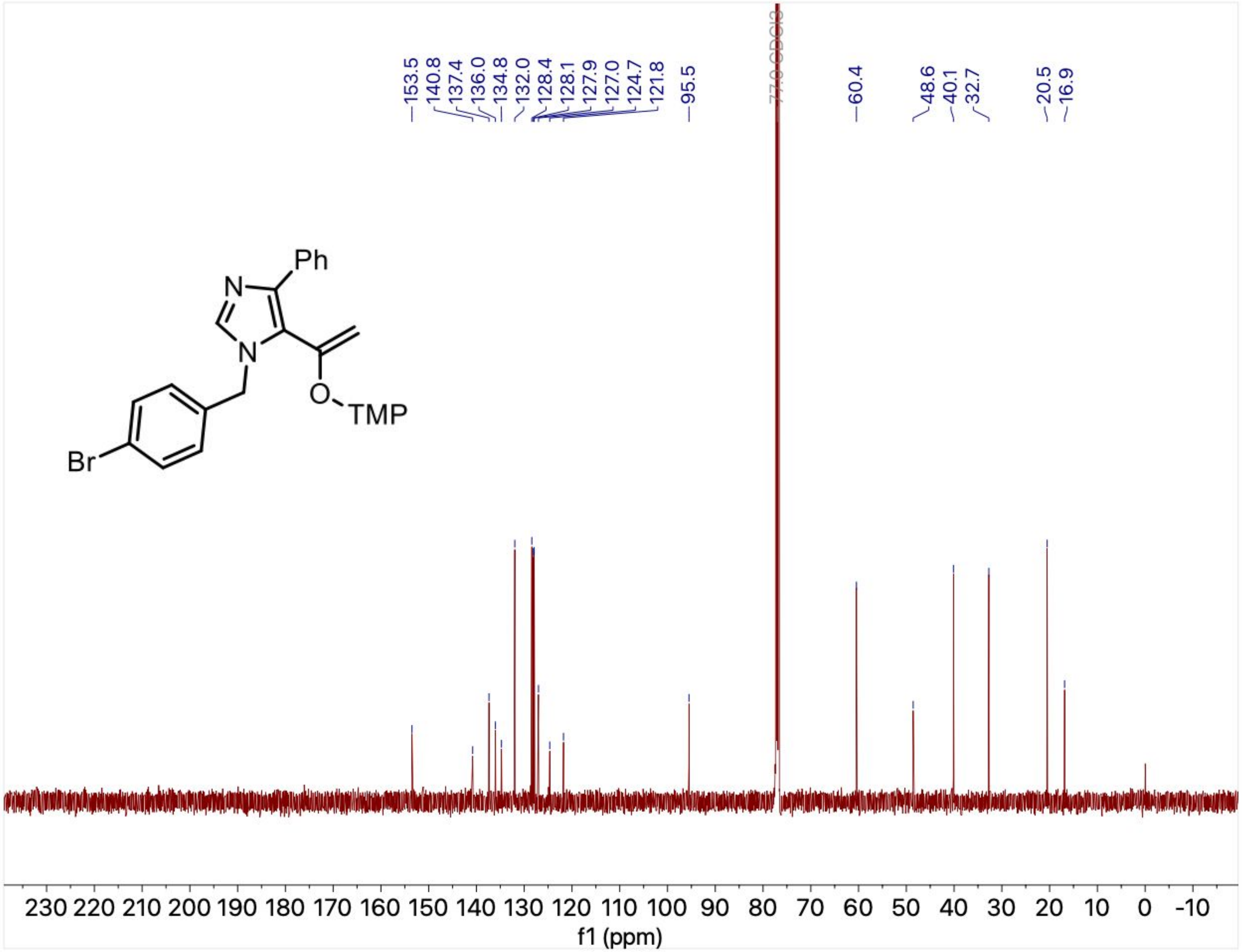

$^{1}H\{^{13}C\}$ **500 MHz NMR for Compound 17.**

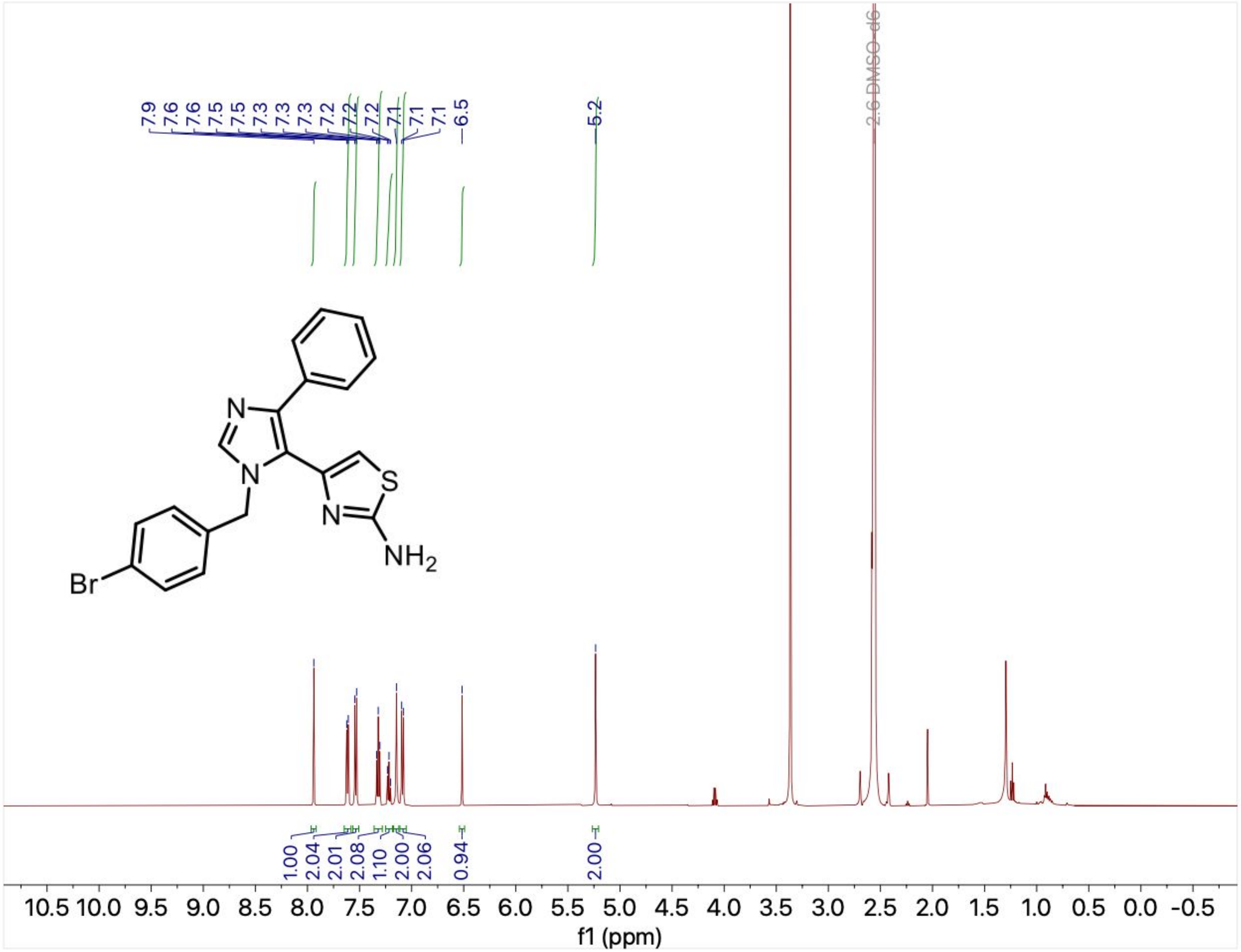

$^{13}C\{^1H\}$ 500 MHz NMR for Compound 17.

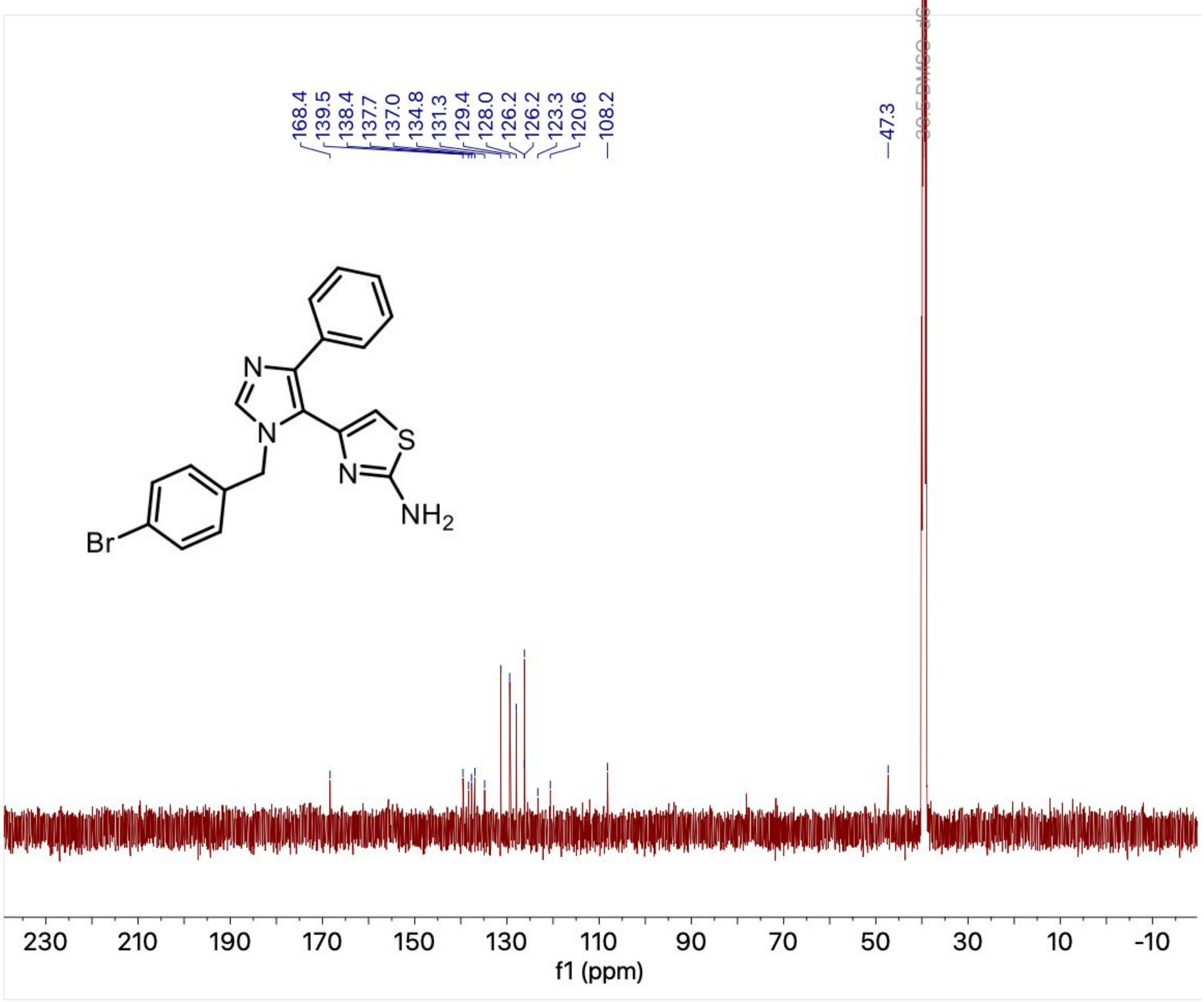

$^{1}H\{^{13}C\}$ **500 MHz NMR for Compound 19.**

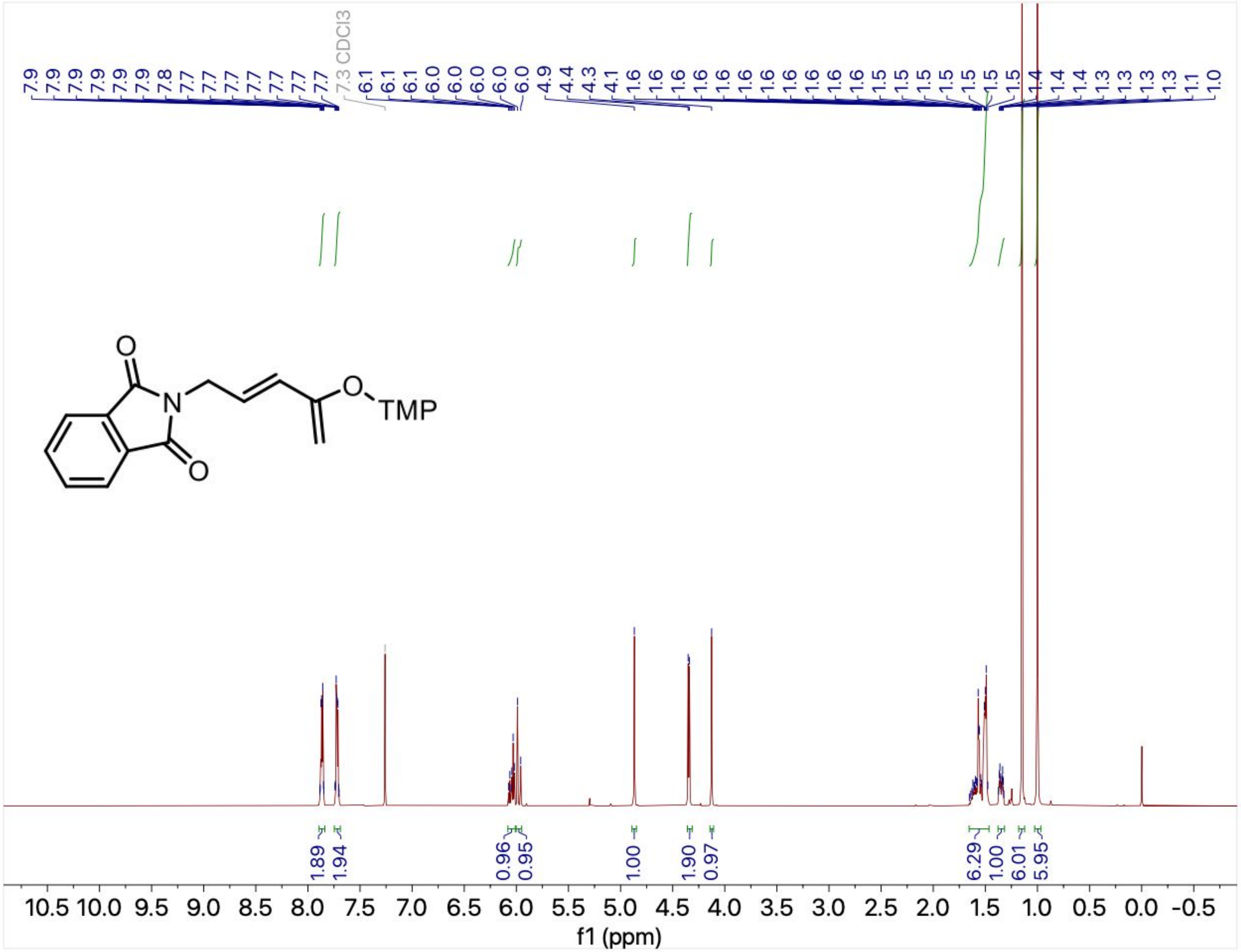

$^{13}C\{^{1}H\}$ 500 MHz NMR for Compound 19.

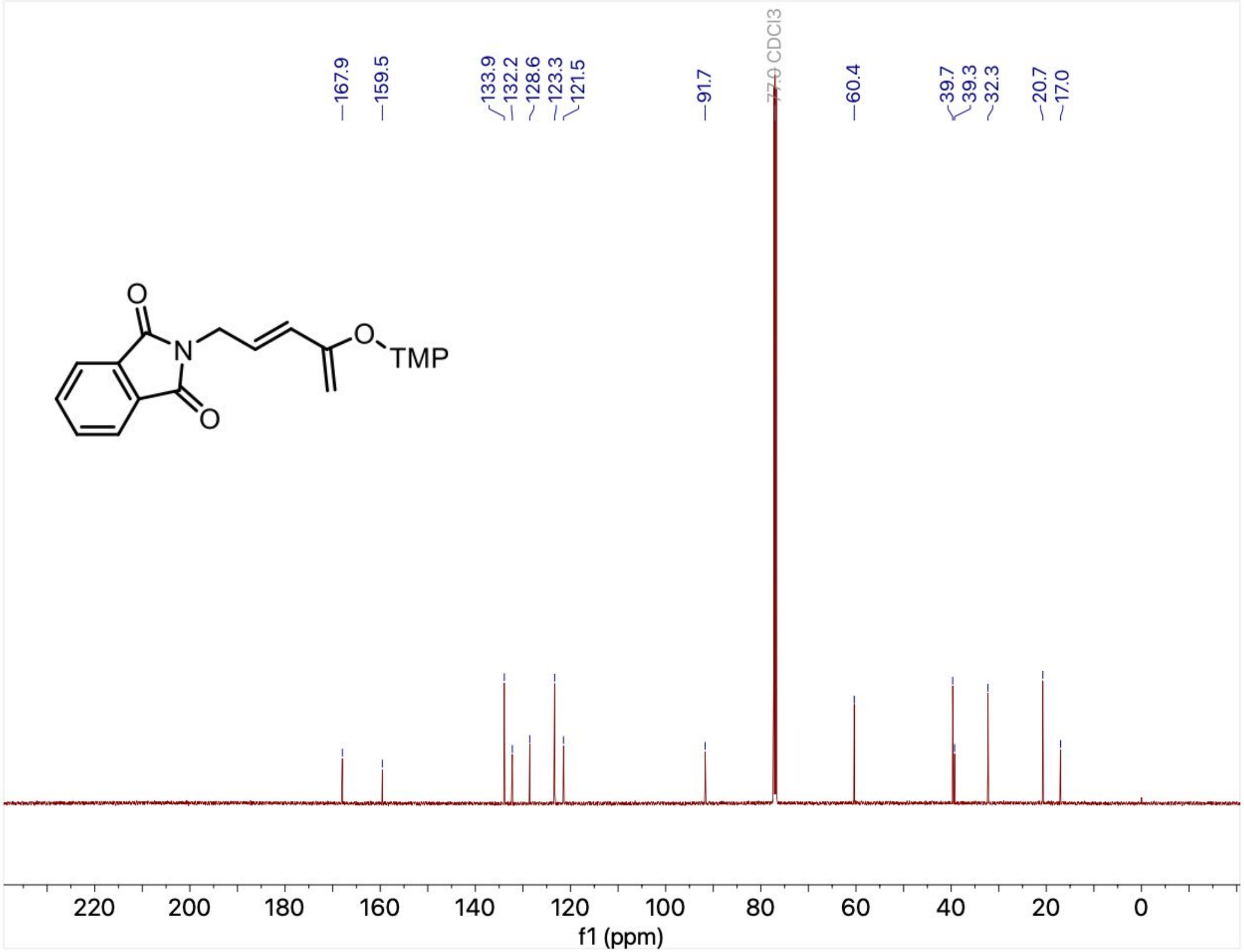

$^{1}H\{^{13}C\}$ 500 MHz NMR for Compound 20.

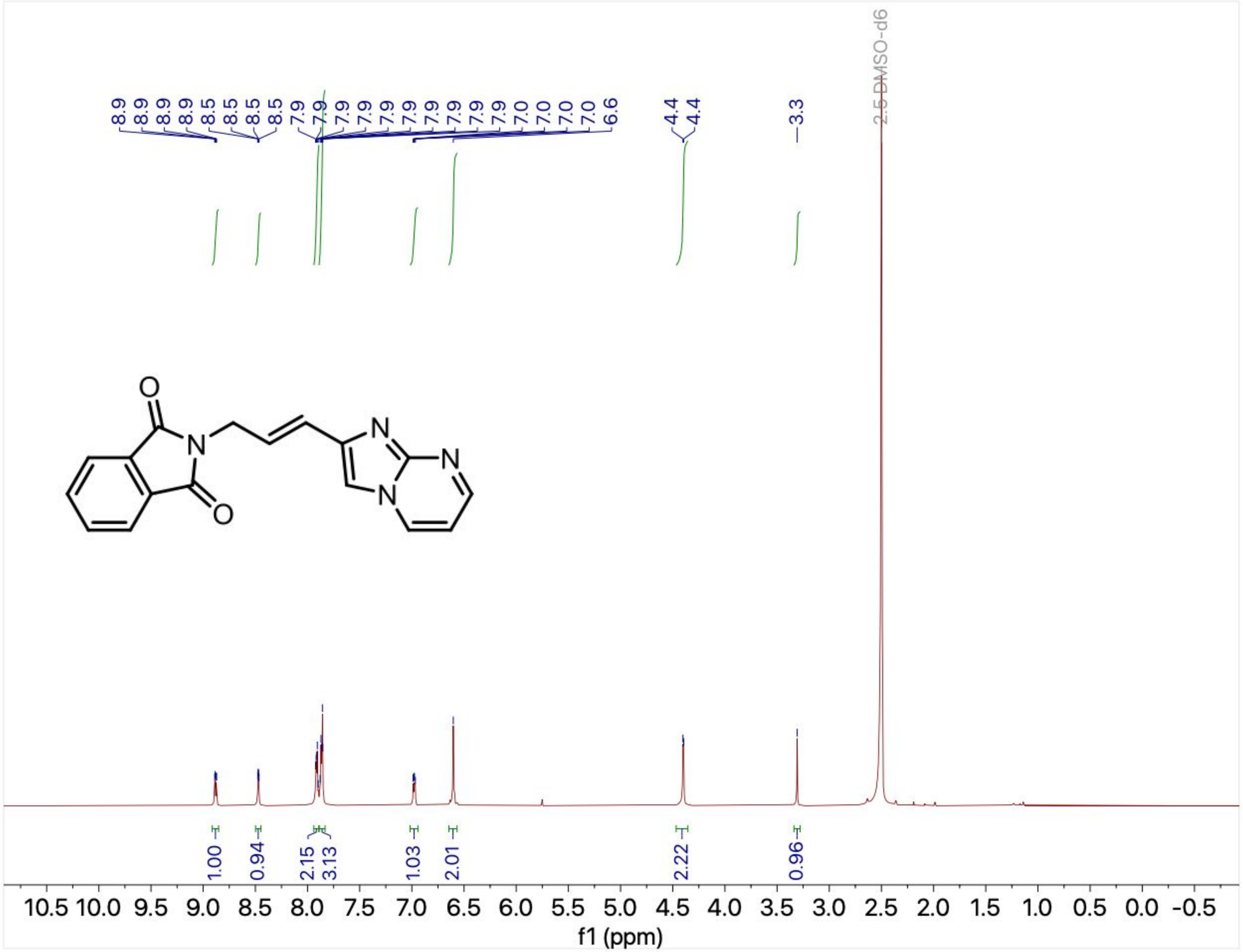

$^{13}C\{^{1}H\}$ 500 MHz NMR for Compound 20.

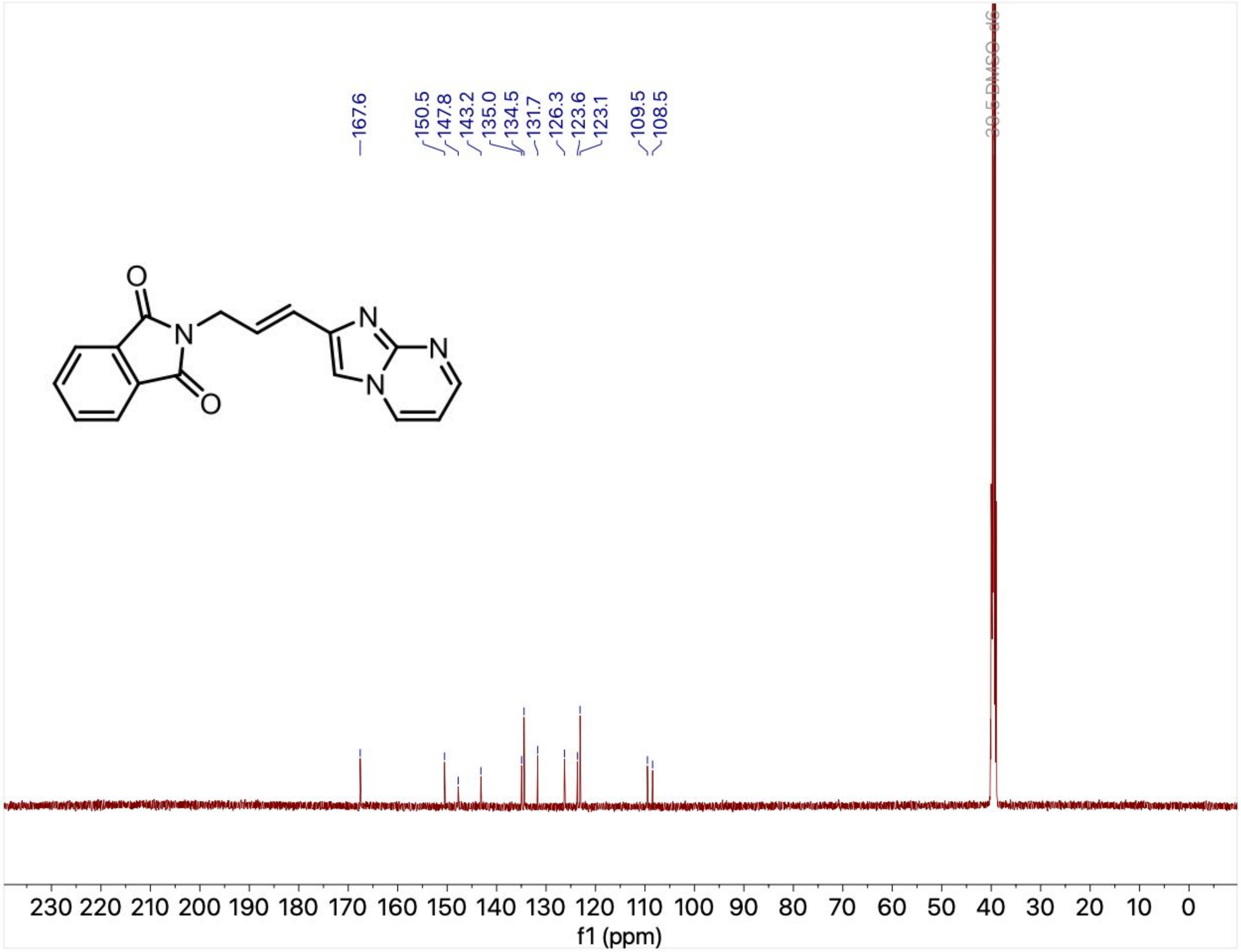

$^1H\{^{13}C\}$ 500 MHz NMR for Compound 21.

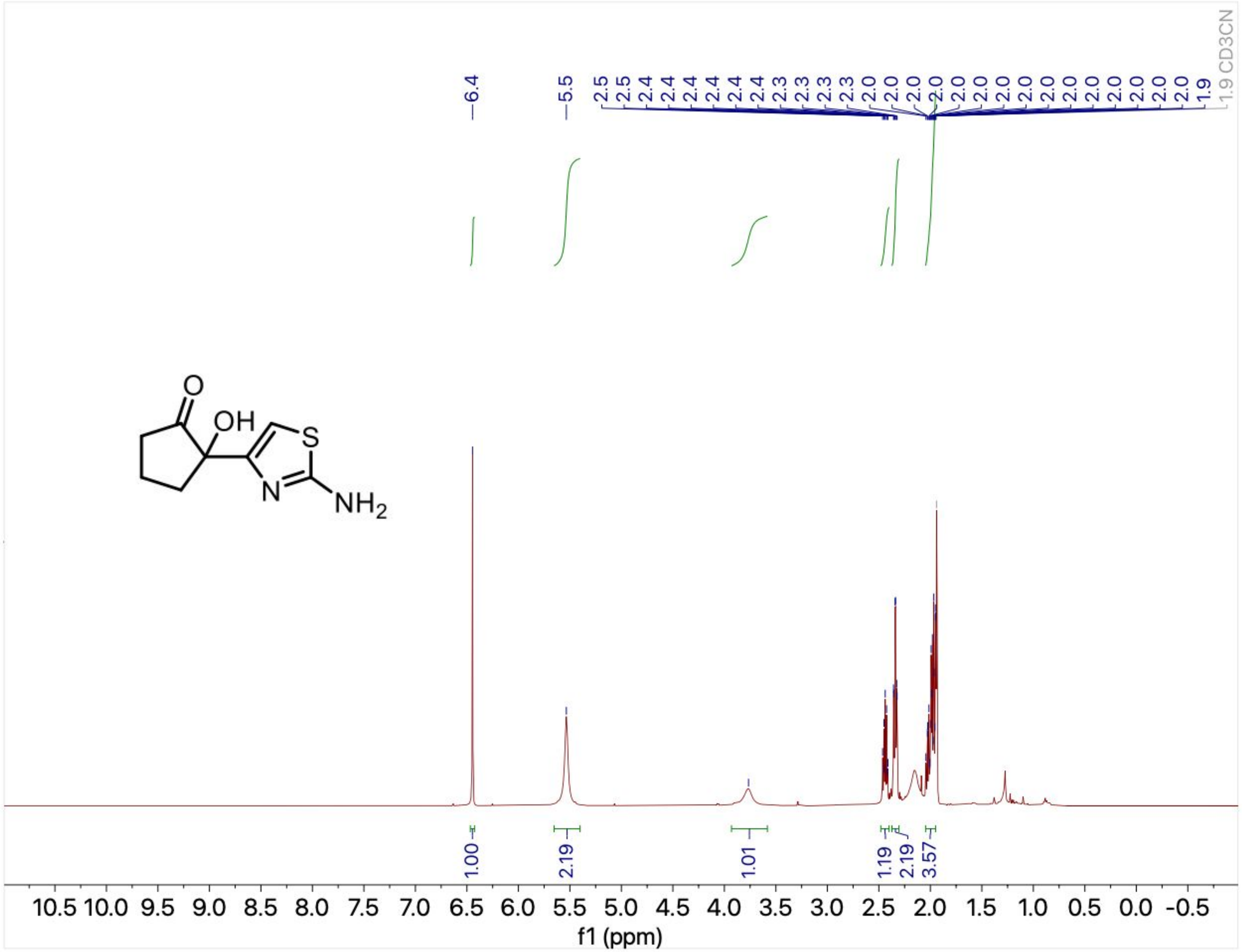

^{13}C{^{1}H} 500 MHz NMR for Compound 21.

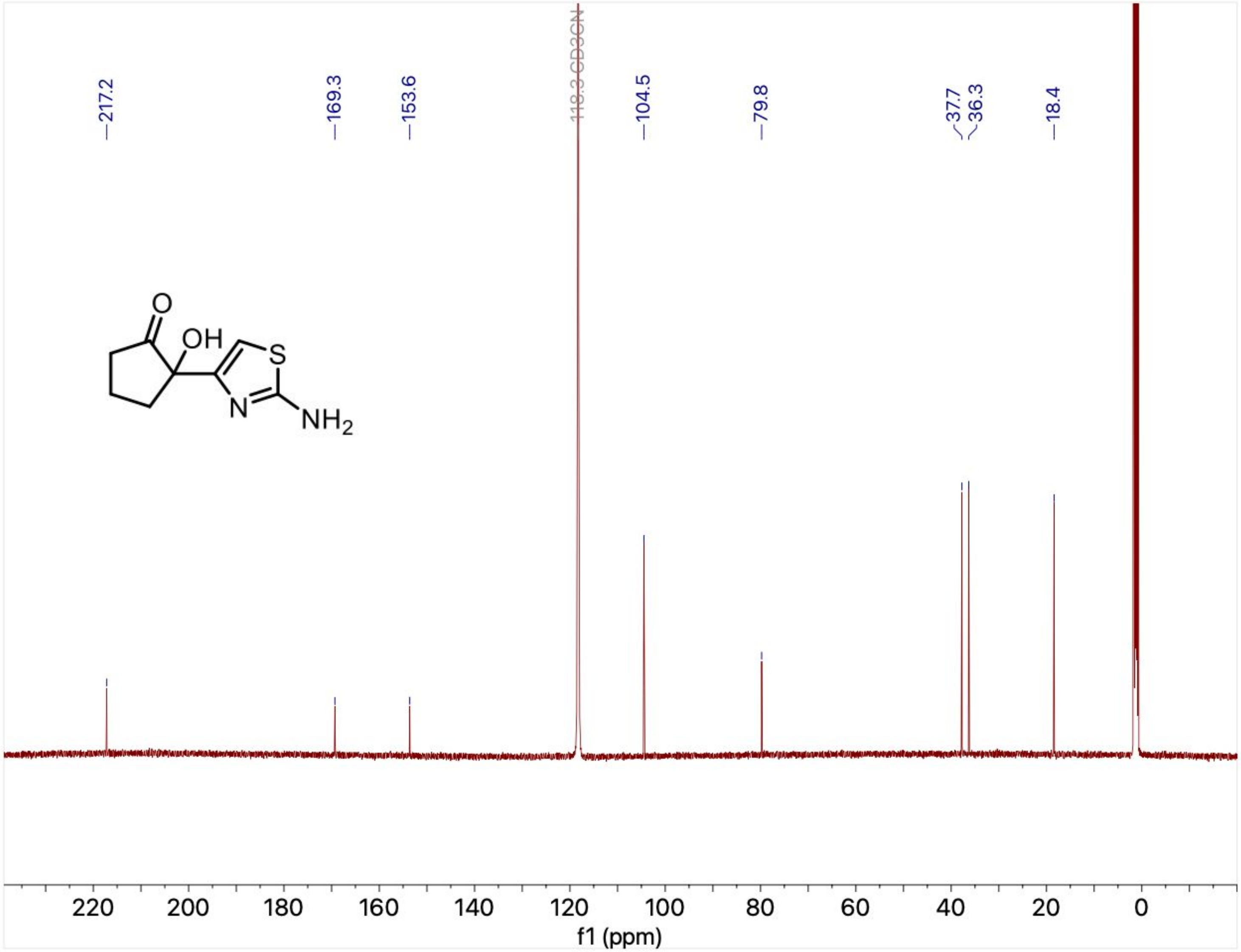

^{1}H{^{13}C} 500 MHz NMR for Compound 22.

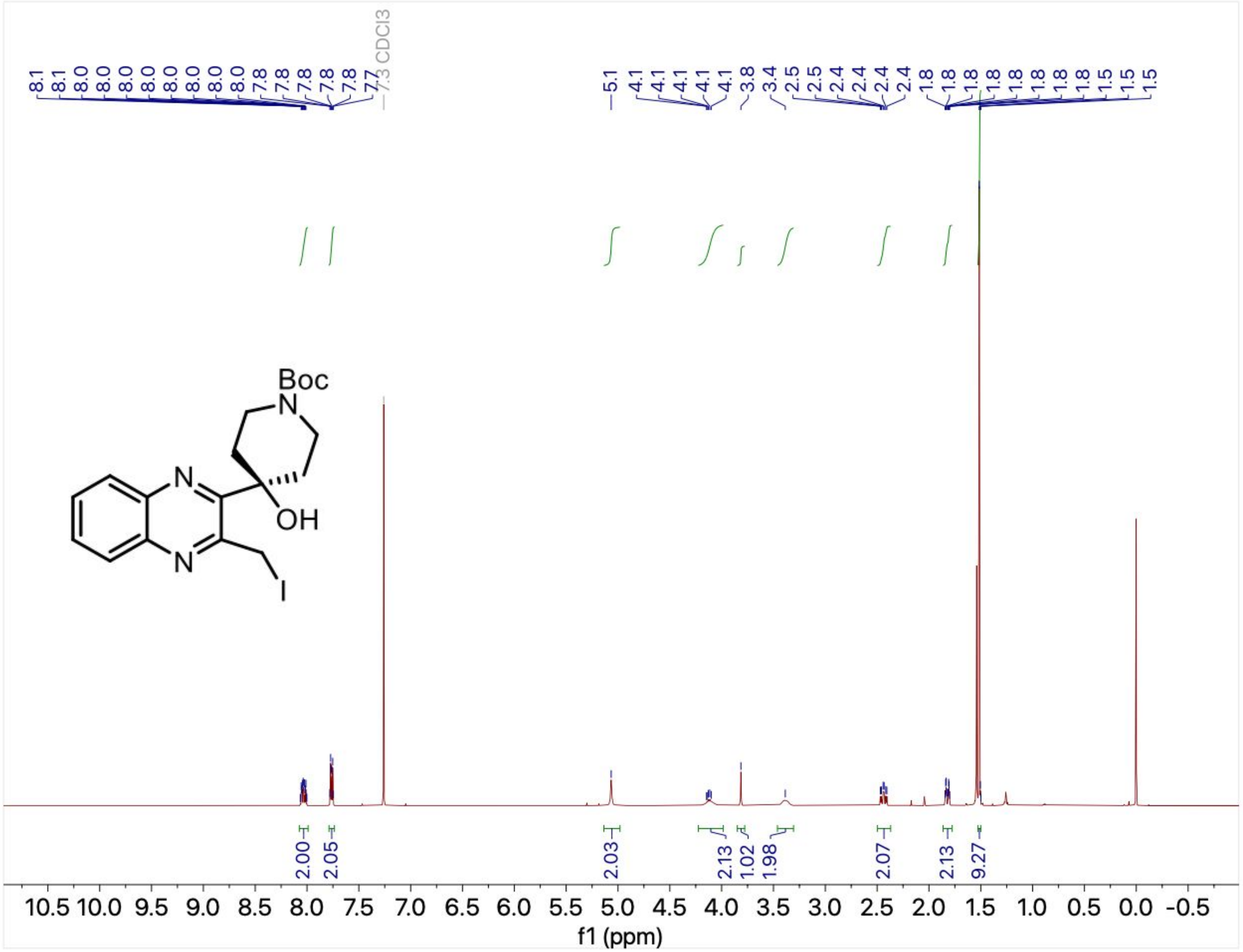

$^{13}C\{^1H\}$ 500 MHz NMR for Compound 22.

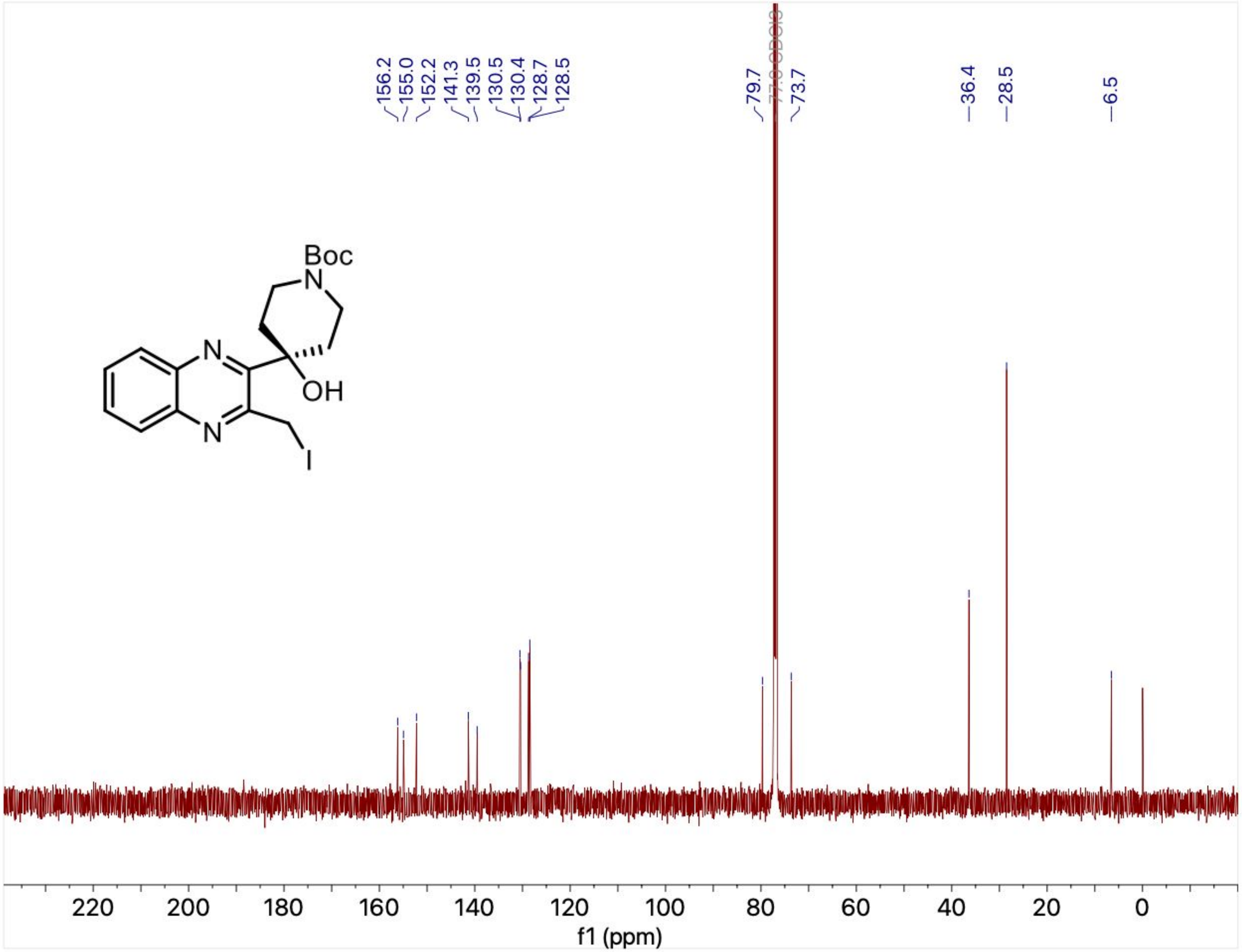

$^{1}H\{^{13}C\}$ **500 MHz NMR for Compound 23.**

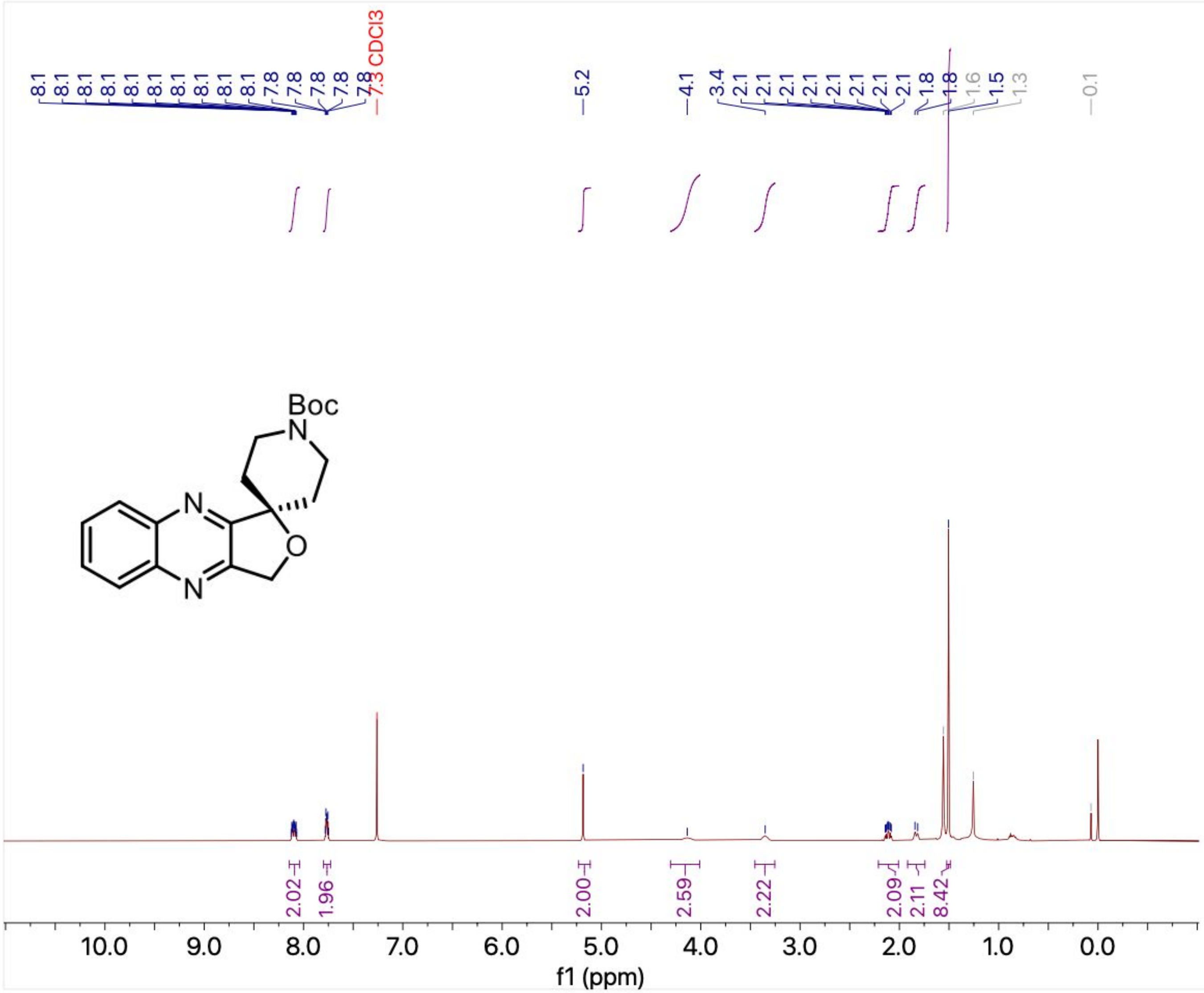

$^{13}C\{^{1}H\}$ 500 MHz NMR for Compound 23.

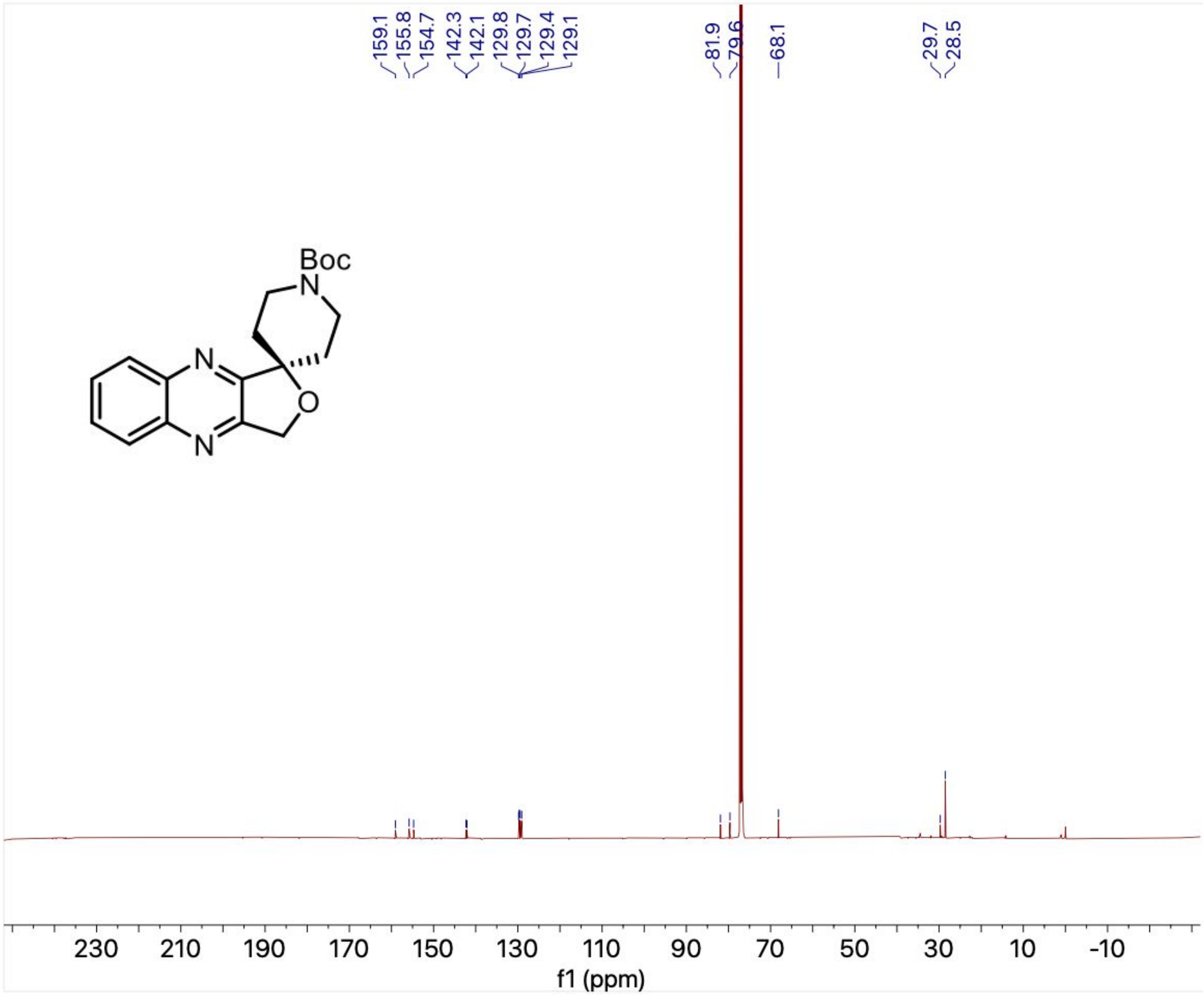

$^{1}H\{^{13}C\}$ 500 MHz NMR for Compound 25.

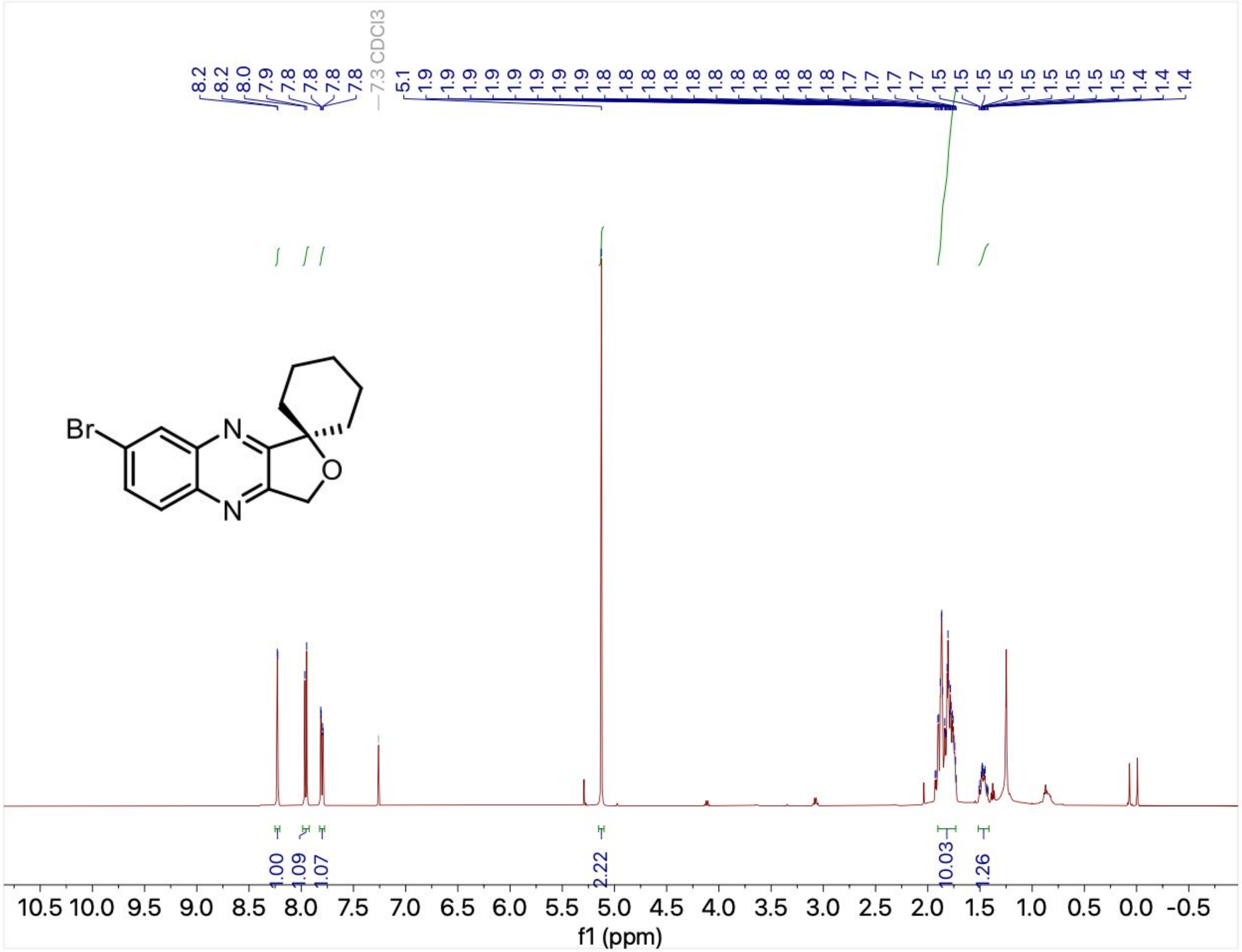

$^{13}C\{^{1}H\}$ 500 MHz NMR for Compound 25.

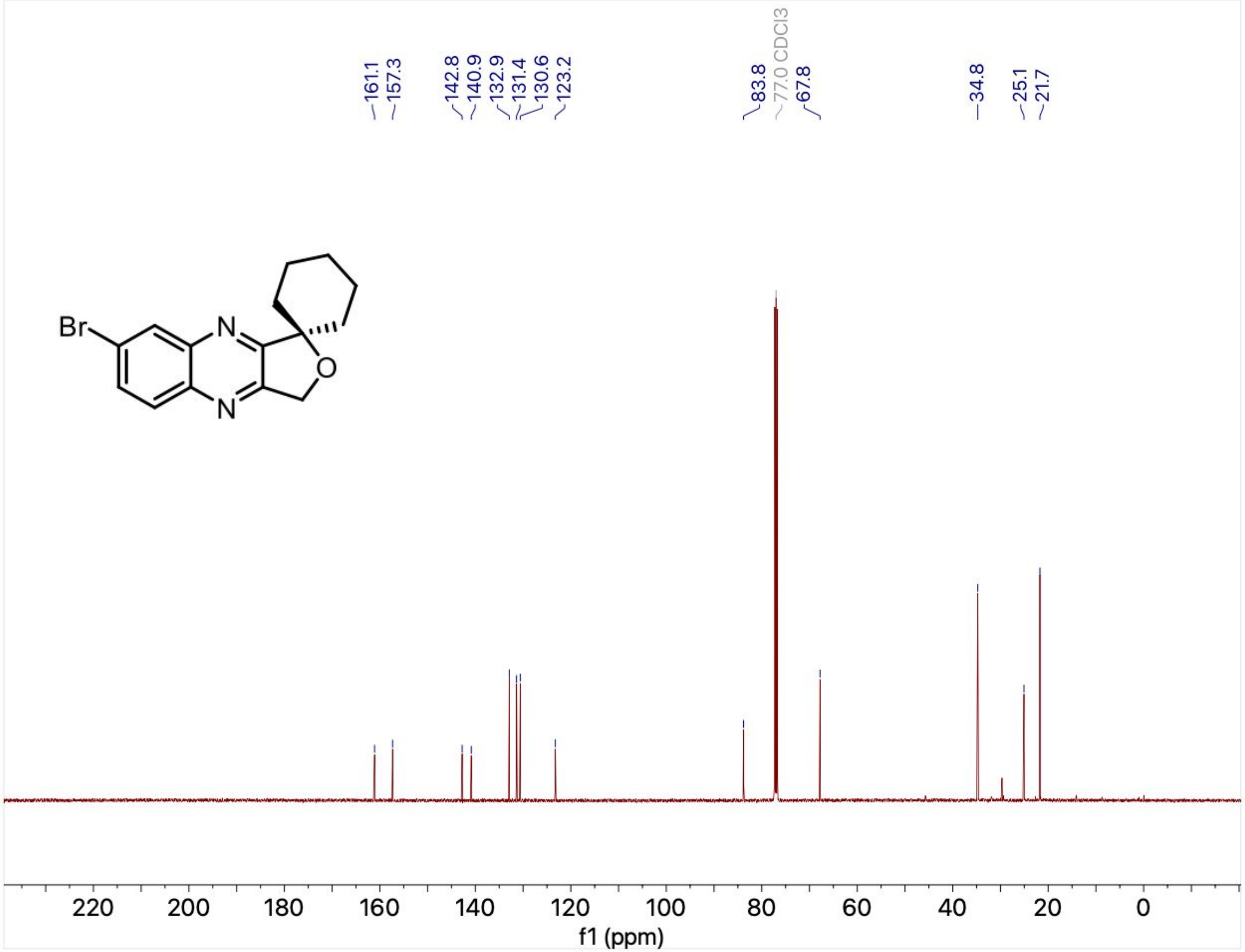

$^{1}H\{^{13}C\}$ 500 MHz NMR for Compound 26.

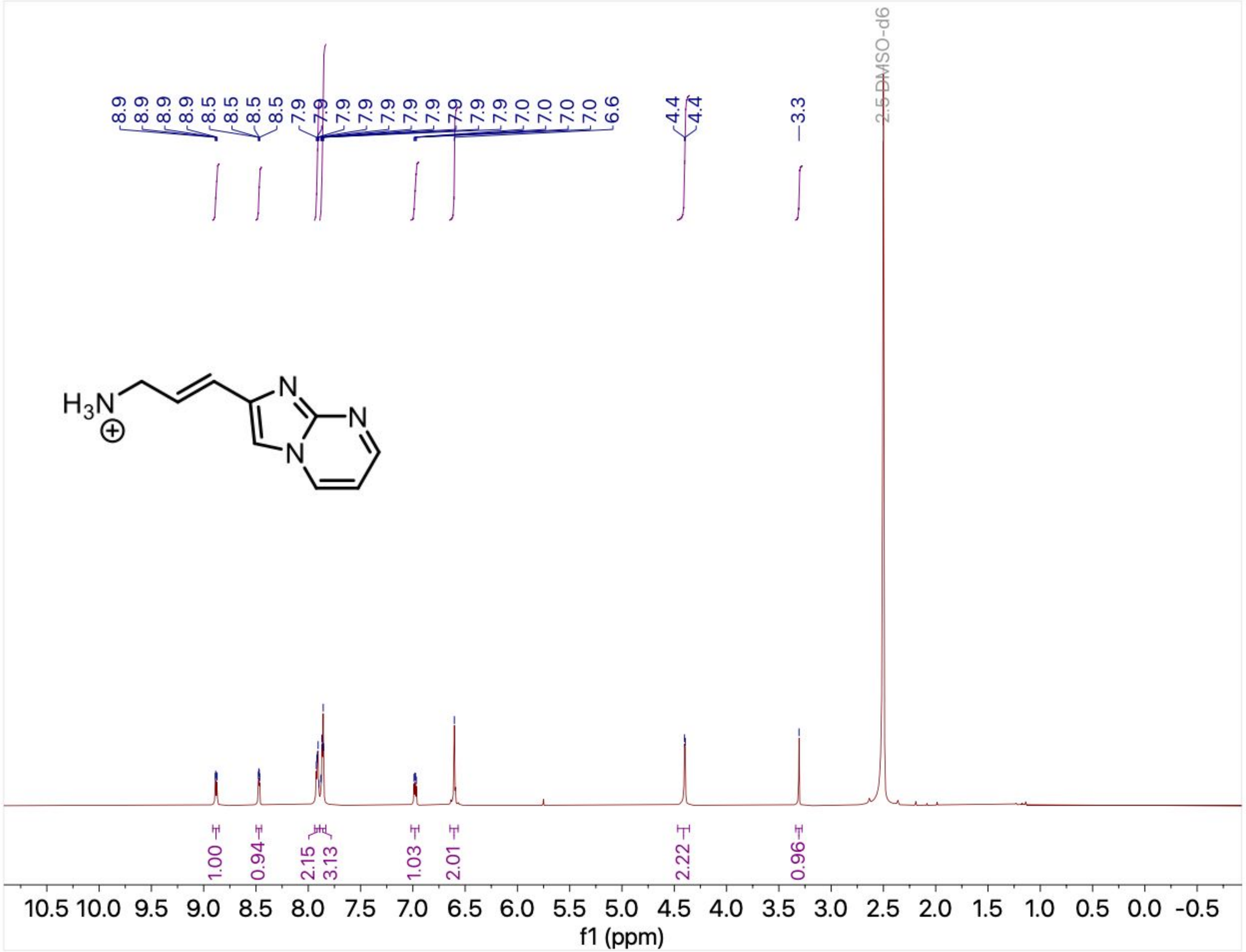

^{1}H–^{13}C HSQC 500 MHz NMR for Compound 26.

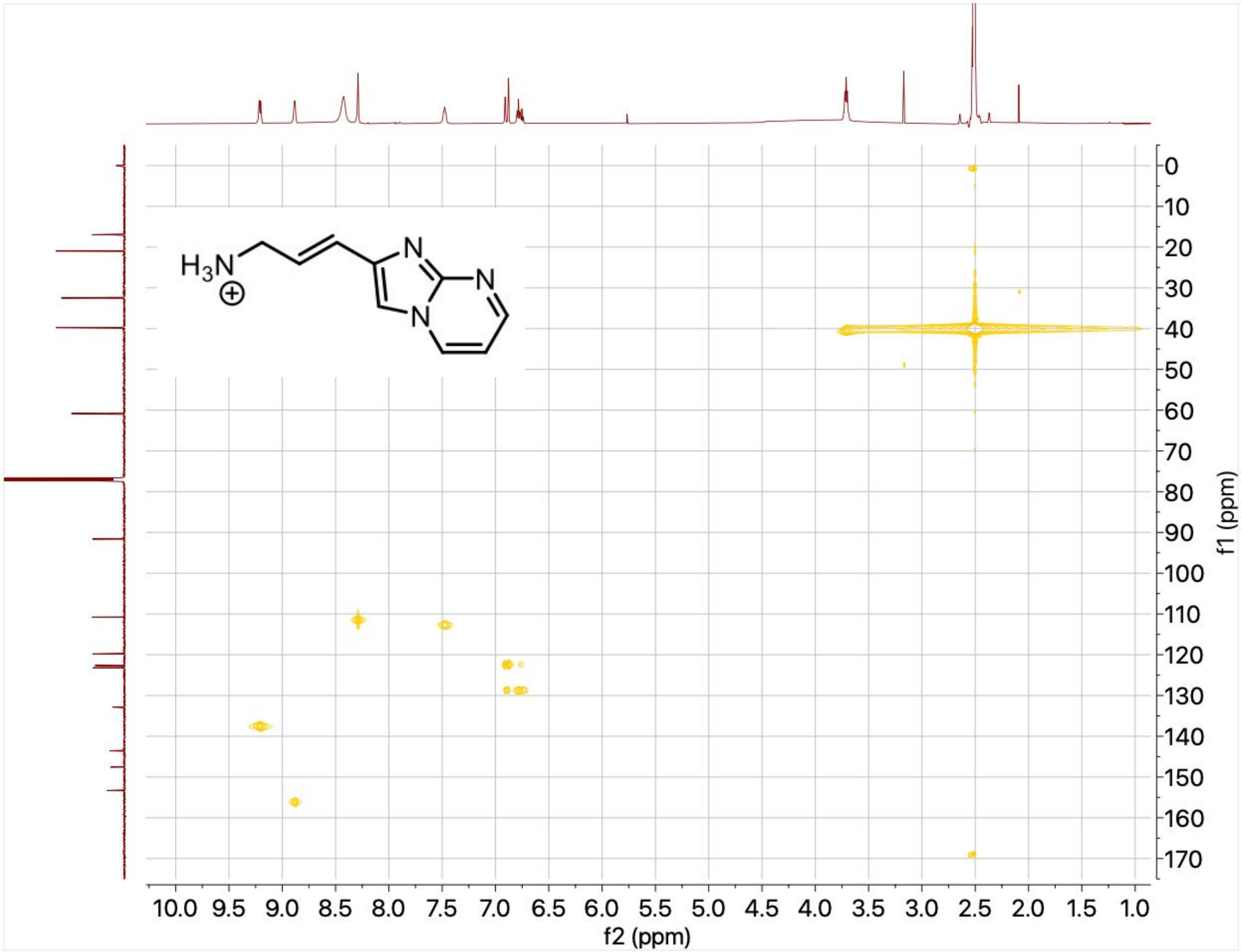

^{1}H{^{13}C} 500 MHz NMR for Compound 27.

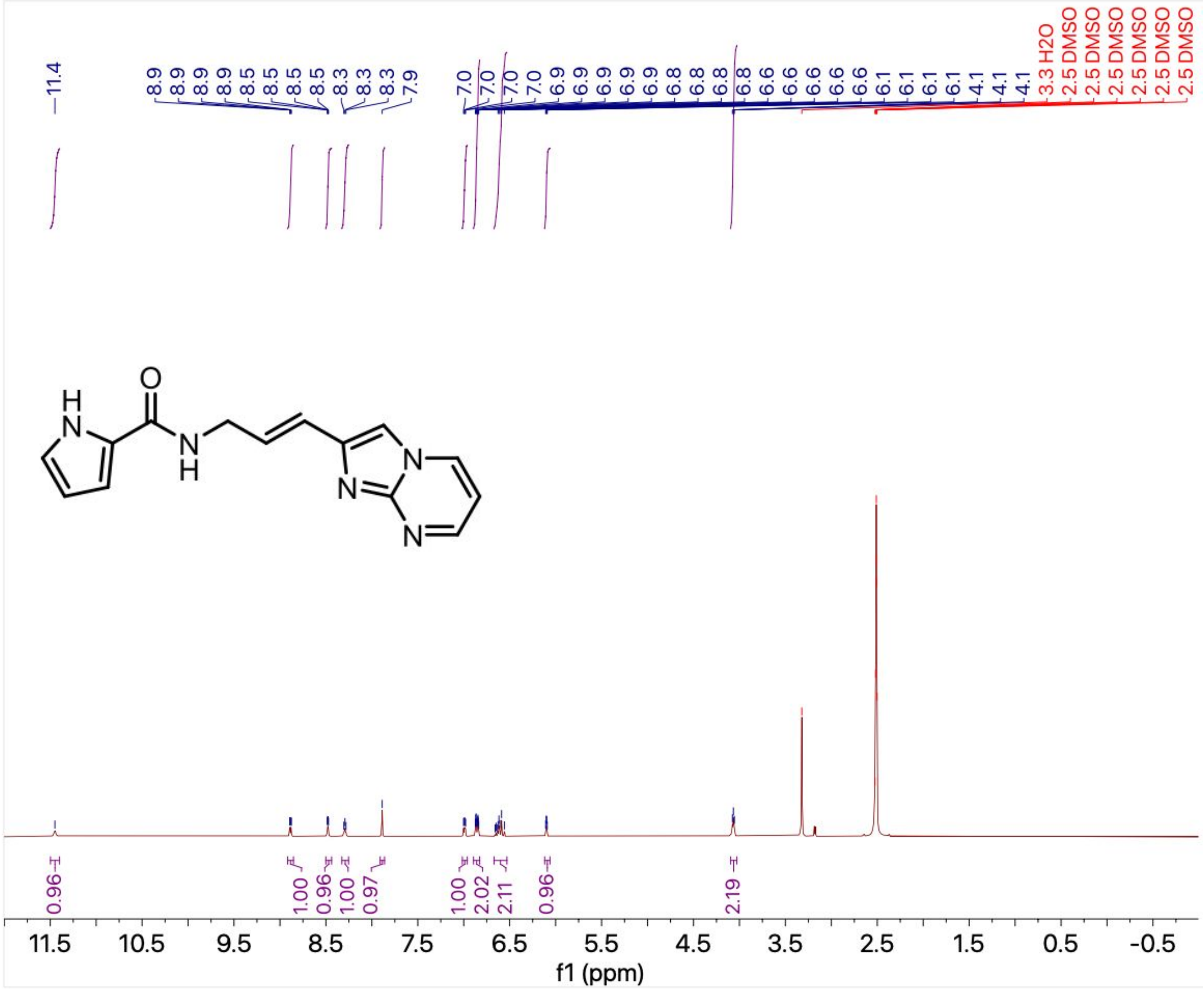

$^{13}C\{^1H\}$ 500 MHz NMR for Compound 27.

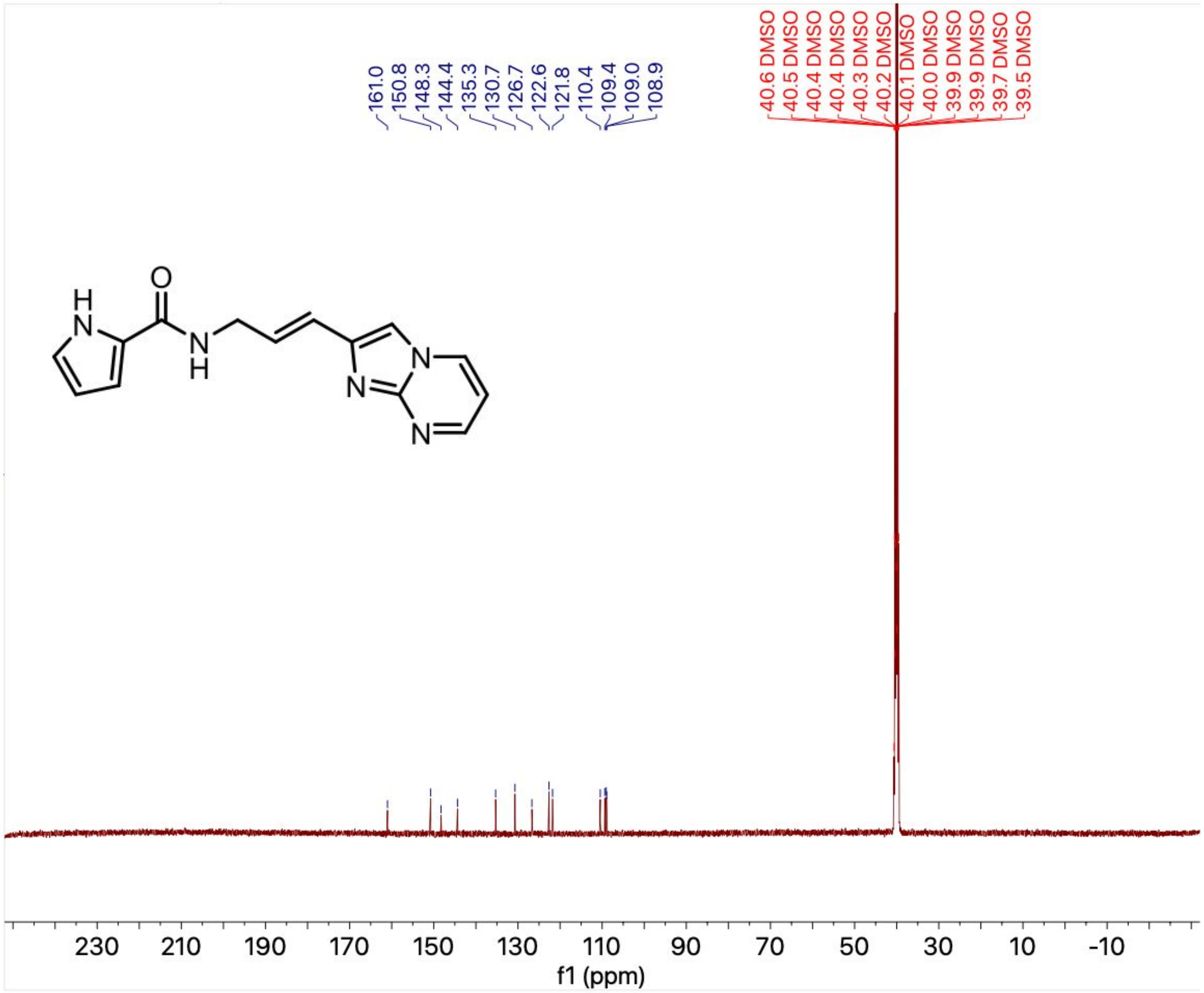

$^{1}H\{^{13}C\}$ 500 MHz NMR for Compound 28.

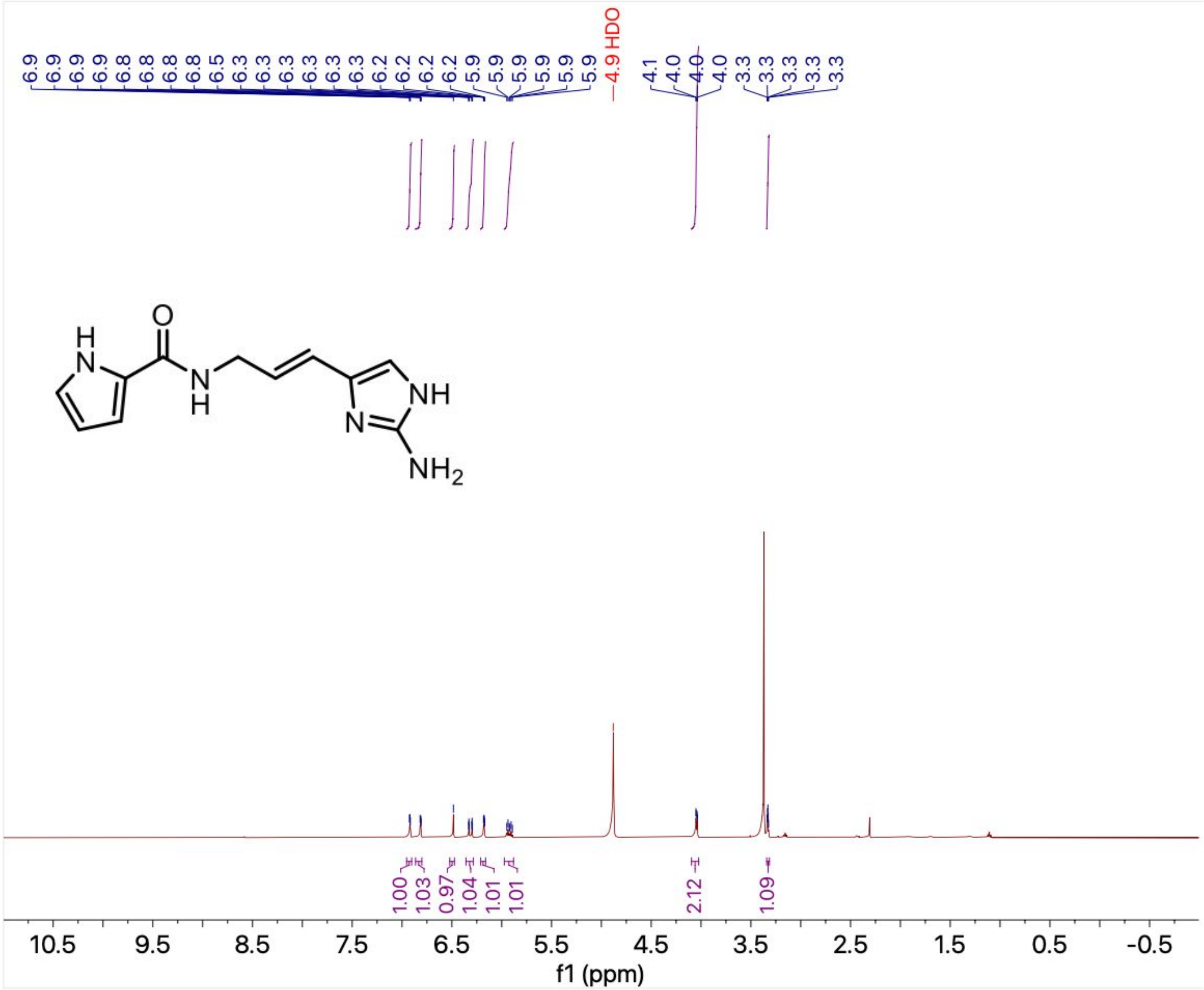

$^{13}C\{^{1}H\}$ 500 MHz NMR for Compound 28.

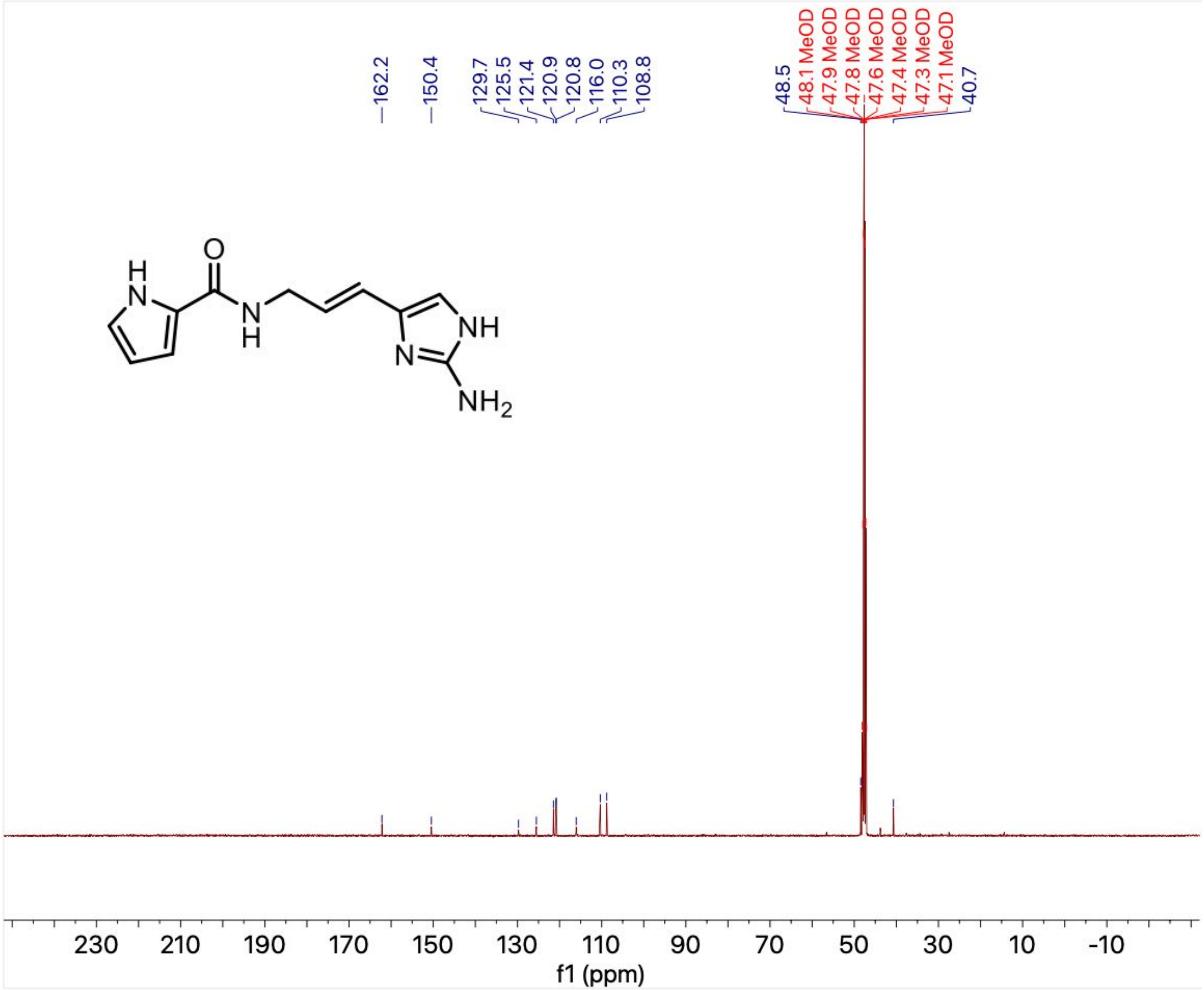

Supplement: Supplementary file 1 [file ja5c21371_si_001.pdf]
